# Long History of Queries about Bovine Paratuberculosis as a Risk Factor for Human Health

**DOI:** 10.3390/pathogens10111394

**Published:** 2021-10-28

**Authors:** Karel Hruska, Leonardo A. Sechi

**Affiliations:** 1 Veterinary Research Institute, 62100 Brno, Czech Republic; 2 Dipartimento di Scienze Biomediche, Sezione di Microbiologia Sperimentale e Clinica, Università degli Studi di Sassari, 07100 Sassari, Italy; sechila@uniss.it

Motto: *All truth passes through three stages. First, it is ridiculed. Second, it is violently opposed. Third, it is accepted as self-evident.* Arthur Schopenhauer (1788–1860)

The story began in 1895, with a description of chronic bacterial enteritis in cattle [1]. The discussion of the problem has been marked by many milestones since 2005, when the American Academy of Microbiology published the report “Microbial Triggers of Chronic Human Illness” [2], and several key publications were published in the journal *Science*.

The enemy, which cannot be destroyed, must be discovered, its positions investigated and drawn on the map. As evident, triggers can be derived from *Mycobacterium avium* subsp. *paratuberculosis* (MAP) or other species of mycobacteria from different sources in many quite unexpected places. Are you surprised that seven million mycobacterial cells can be found in one gram of house dust or 10 million per one gram of pork lymph node? Do you envy the blower operator their job in the fresh air, or do you enjoy using an air blower to clean your garden or backyard? Are you happy when the pavement under your windows is cleaned by an air blower? Is it cleaning at all? Did you expect contamination of bottled mineral water with mycobacteria? Thousands of scientific papers have addressed questions on the ecology of mycobacteria and their impact on animal and human health. Nevertheless, mycobacteria still require more attention from microbiologists, ecologists, biomedical scientists, clinicians, gastroenterologists, immunologists, surgeons, dentists, veterinarians, public health and food inspectors, farmers, technicians in the water industry, and many others. 

Mycobacteria were first suspected to be associated with Crohn’s disease more than 100 years ago, when T. K. Dalziel, a surgeon from the Western Infirmary, Glasgow, published his paper on chronic interstitial enteritis in *The British Medical Journal* in 1913 [1]. He made a possible diagnosis of tuberculosis, but also noted the uniform character of the infection. The lymph nodes were enlarged but not caseous, and bowel consistence and smoothness resembled “an eel in a state of rigor mortis”. Furthermore, Dalziel cited an earlier description of chronic bacterial enteritis in cattle, already observed in 1895 (Henny and Frothingham). The disease described by McFadyen in cattle in 1911 and by Dalziel in humans in 1913 were so similar with regard to their histological profiles as to justify the proposition that the diseases may be one and the same. McFadyen described “an acid-fast bacillus, similar to but demonstrably not the tubercle bacillus, differing in size and also not giving rise to tuberculosis in guinea-pigs”. This first hypothesis has run since that time like a thread through the history of Crohn’s disease for more than one hundred years. Mycobacteria, and specifically MAP, suggested as an etiological factor by McFadyen, are regarded as prominent candidates for participation in the etiology or pathogenesis of Crohn’s disease. Infection by listeria or by viruses in anamnesis, namely, measles, adherent invasive *Escherichia coli*, or imbalances in the intestinal microbiome are also suspected causes. There are several good reasons to consider mycobacteria as the most important, but not the only etiological factors in the development of Crohn’s disease [3,4,5,6,7,8,9,10]. Dead mycobacteria have been widely used as immunomodulators in Freund’s adjuvant since 1956. The mechanism is known and experimentally confirmed. 

In 2000, the European Commission attempted to demonstrate the involvement of mycobacteria in the development of Crohn’s disease, and an expert report stated that “the link cannot be confirmed or ruled out” [11]. Members of the European Parliament have repeatedly called for an update of this report. The European Commission has promised to reopen the problem when it has more information. However, this has not happened since 2000, although hundreds of new publications have been published. The EU 5th Framework Program project Pathogen Combat organized a discussion in 2009 entitled “Bacterial triggers in the etiology of Crohn’s disease and other autoimmune and autoinflammatory diseases” [5]. Twenty experts from Europe and overseas were invited [12]. Unfortunately, the participants’ statement that triggers of Crohn’s disease and other inflammatory diseases should be considered a serious risk was not accepted.

In 2015, the European Commission asked the European Food Safety Authority (EFSA) for a scientific opinion on the categorization of paratuberculosis in the light of the Animal Health Act. The result was published in 2017, but does not mention mycobacterial triggers or the risks they pose at all, although some publications are cited to draw attention to them [13].

We hope that this *Pathogens* Special Issue will be an important milestone in this disputation.

Selected Supplementary Materials are chronologically arranged.

## Supplementary Materials

The following are available online at https://www.mdpi.com/article/10.3390/pathogens10111394/s1, PDF1: DALZIEL, PDF2: EUROPEAN COMMISSION, PDF3: CARBONE, PDF4: NACY, PDF5: LIPTON, PDF6: KAZDA, PDF7: HRUSKA List of participants, PDF8: HRUSKA Publ excerpts, PDF9: SWEENEY, PDF10: AGRAWAL 2014, PDF11: EFSA PANEL, PDF12: KUENSTNER, PDF13: AGRAWAL 2021.
