# Supplementary material for: Long History of Queries about Bovine Paratuberculosis as a Risk Factor for Human Health
Source: Pathogens. 2021 Oct 28;10(11):1394. doi: 10.3390/pathogens10111394 (PMC8622788; doi:10.3390/pathogens10111394)
Supplement: Supplementary file 1 [file pathogens-10-01394-s001.zip › AGRAWAL 2014.pdf]

Future
MICROBIOLOGY

EDITORIAL

For reprint orders, please contact: reprints@futuremedicine.com

'Global warming' to *Mycobacterium avium* subspecies *paratuberculosis*

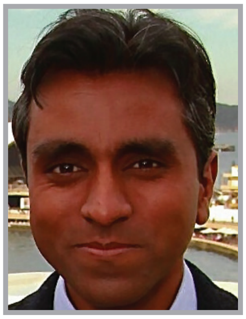
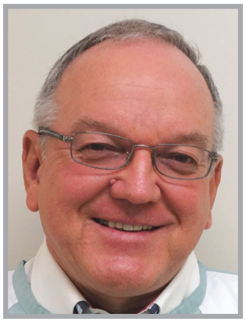
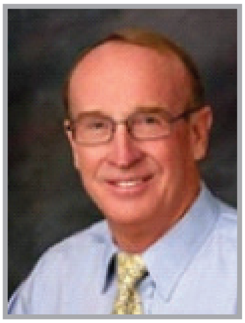

Gaurav Agrawal*[,1], Thomas J Borody[1] & William Chamberlin[2]

There is a growing worldwide movement to investigate the relationship between Crohn's disease (CD) and microorganisms, especially for causality. Scientists and doctors are warming to this historical idea again, particularly with the advent of discoveries involving the gut microbiome, metagenomics and the revelations of the deficiencies of the innate immune system and autophagy. Looking back, early CD reports were already concerned with finding an infectious cause, which is being revisited by researchers around the globe.

History

CD was described by the Scottish surgeon, TK Dalziel in 1913 [1], though it was probably described even earlier by a Polish surgeon Antoni Leśniowski in 1904 [1]. Subsequently CD was labeled as 'regional ileitis' by an American gastroenterologist, Burril Crohn [1] and so it became known as CD. Dr Burril Crohn commented initially upon its similarities to known mycobacterial infections of the gut, such as *Mycobacterium tuberculosis*, particularly in that it was of a 'granulomatous enteritis' nature. However, given mycobacteria were not cultured, alternative proposals were forwarded. Psychosomatic origins were discussed in the 1950s and a decade later the concept that CD was an autoimmune disorder came to the fore, and it included the involvement of dysfunctional T cells of the adaptive immune response. This explanation dominated the field for the next 40 years, but more recently, however, a newer concept has evolved which describes the inflammatory response occurring secondary to an aberrant reaction to the body's normal gut flora [2].

Recent work has led to the hypothesis that innate immune deficiencies are central to a dysregulated chronic inflammatory process. This proposal stresses the importance of dysfunctional macrophages and dendritic cells. These antigen-presenting cells are impaired in their ability to signal to the rest of the immune network and show diminished ability to kill intracellular infections. Along with other functional abnormalities, disturbances of cytokine signaling impair neutrophil chemotaxis resulting in significant aberrations throughout the innate and adaptive immune network.

KEYWORDS
• autophagy • Crohn's disease • Dietzia • fecal microbiota transplantation • microbiota • *Mycobacterium paratuberculosis*

"There is a growing worldwide movement to investigate the relationship between Crohn's disease and microorganisms, especially for causality."

[1]Centre for Digestive Diseases, Level 1, Five Dock, NSW 2046, Australia
[2]Montana State University, Billings, Saint Vincent Healthcare, Billings, MT 59101, USA
*Author for correspondence: Tel.: +61 2 9713 4011; Fax: +61 2 9713 1026; drgagrawal@gmail.com

Future Medicine part of fsg

The concept of CD being triggered by a *Mycobacterium* is gaining momentum with the advent of new molecular techniques and cytogenetics. *Mycobacterium avium* subspecies *paratuberculosis* (MAP) is once again coming under the microscope due to the increased ability to detect it's genetic signature. This is the best contender for a causative microorganism of CD which infects macrophages and disrupts the microbicidal and immune signaling function of these immune cells. Indeed it is the accepted cause of a similar chronic inflammatory bowel disease in cattle, known as Johne's disease. This postulated zoonosis and its controversial mode of transmission shares similarities (clinically and histopathologically) with *M. tuberculosis* enteritis and CD in humans. CD is becoming an epidemic and has risen almost exponentially, with large increases being seen across the globe. Traditionally 'low-incident' countries, for example, China, India and Latin America now have to deal increasingly with this problem.

Autophagy

Autophagy is essential for macrophages to eradicate intracellular pathogens, especially mycobacteria such as MAP. Dysfunctional macrophages are central players in the CD process characterized by intracellular infections or the converse where such successful intracellular pathogens downregulate autophagy as an evolved survival strategy, which pathogenic mycobacteria have been shown to adopt as a survival strategy. Genome-wide association studies identified over 70 single nucleotide polymorphisms associated with CD with the strongest linkages associated with genes involved in the recognition and response to intracellular infections – that is, *NOD2, IRGM* and *ATG16L1*. These peptides are all involved in macro-autophagy and implicated in the pathogenesis of CD. Even more striking was the discovery that these same mutant gene alleles are associated with leprosy, tuberculosis and 'other mycobacterial infections'. A weak autophagy response to microbial infections at the intracellular level results in all the findings of CD: innate immune deficiency, poor neutrophil chemotaxis, compromised macrophage microbicidal function impaired cytokine signaling, persistent intracellular infections, chronic inflammation and granulomas, among others [3].

> "Rational future therapies need to focus on reversing the ineffective autophagy and eradicating *Mycobacterium avium* subspecies *paratuberculosis.*"

MAP & CD

MAP primarily targets the human intestine and causes similar inflammatory bowel disease in a wide range of mammals, including non-human primates. It infects dendritic cells and macrophages and when these cells are infected their molecular signaling pathways are impaired. The homeostatic balance of the entire immune system is disrupted and chronic inflammation occurs. Ineffective attempts by the immune system to destroy MAP results in tissue damage and the broad spectrum of clinical disease that occurs in patients with CD. Indeed this is similar to leprosy (which is caused my *Mycobacterium leprae*, and to which MAP is phenotypically similar) [4]. Bovine- and human-derived MAP isolates evoke the same immune responses. Similarities evoked by early MAP infection in cattle during the latent and early clinical period to those seen in human CD are striking. These similarities are found at the molecular, cellular and tissue levels [5].

The different species of the Mycobacteria family have similar general features but different characteristics and behavior. Literature suggests a qualitatively different role for TNF-α during infection with *M. tuberculosis* as compared with *M. avium* [6]. MAP has been shown to be associated with increased levels of TNF-α – more than other types of mycobacteria and that it thrives on high levels, as shown by Bach *et al.* [7]. TNF-α induces apoptosis of MAP infected cells and so drugs, such as infliximab, act as an antibiotic as well as an anti-inflammatory agent. Apoptosis is induced in MAP-infected macrophages, that secrete higher amounts of TNF-α on their surface and so the survival of the white cell is reduced along with the intracellular organism. In addition, it reduces the antibody titers of two mycobacterial proteins associated with MAP [7]. The authors propose that the combination of infliximab and anti-MAP antibiotics is likely to be synergistic in healing CD lesions and have noted this in their clinical practice to date.

A 2-year trial of anti-MAP therapy carried out by Selby *et al.* initially stated that it does not "find evidence of a sustained benefit and does not support a significant role for MAP in the pathogenesis of CD in the majority of patients" [8]. However, corrected analysis of the data by scientific peers revealed widespread and serious flaws and misinterpretations that led to inaccurate conclusions [9,10] and re-analyzed data showed differences between the two groups with a highly significant treatment response to antibiotics compared with the immunosuppressive arm [11].

The future

Weak macro-autophagy response, either genetic, acquired or both, begets innate immune deficiency that predisposes to chronic intracellular macrophage infections and a dysregulated innate and adaptive immune response resulting in the CD. Rational future therapies need to focus on reversing the ineffective autophagy and eradicating MAP.

• Targeting MAP using antibiotics

MAP has fulfilled Koch's postulates as the cause of CD [12], a set criteria used to prove causality of a disease by a microorganism. As such anti-MAP treatment is increasingly prescribed as a therapy for CD using a combination of antibiotics that targets MAP and has been shown to be quite effective [13]. To prevent development of resistance during long term therapy a combination of antibiotics is required to target the bacterium at all stages of the life cycle including reproduction and dormancy. A randomized controlled trial in CD using such a combination is currently in progress and this could prove the effectiveness of a therapy that targets the MAP organism [14].

• Competitively inhibiting MAP

Dietzia subspecies C79793-74, previously known as *Mycobacterium gordonae*, is a potentially useful and novel step in treating MAP. Acting to displace MAP from the macrophage represents a novel therapeutic method of removing MAP from its niche so taking away its survival environment. By using an evolutionarily more 'adept and inert' member of the same family to replace its 'cousin', we could be utilizing a naturally occurring method in evolutionary competition. Data for this potential therapy are based on its effectiveness as a prophylactic therapy in cattle [15]. Dietzia is a nonpathogenic microorganism used to competitively displace and inhibit MAP infection. Some 40% of cattle with early Johne's disease – which is notoriously difficult to treat – were cured with this oral probiotic and the effect was long lasting compared with the use of antimycobacterial antibiotics. Hence, it could be used in the same manner for Crohn's patients [16].

• Targeting the microbiome & modifying immunity

There have been reports of fecal microbiota transplantation reversing active CD by the implantation of normal donor gut microbiota into the bowel of CD individual [17,18]. Partial and complete disappearance of CD has been achieved and the authors have now three patients with complete remission off therapy between 12 months and 13 years after fecal microbiota transplantation. This is a sporadic result and not the norm but indicates potential for further research in restoration of the bowel microbiota as a future cotherapy in CD [19]. Furthermore it argues against the cause of inflammatory bowel disease being an "inappropriate and ongoing activation of the mucosal immune system driven by the presence of normal luminal flora" [2]. The mechanism(s) of action is not well understood but clearly the microbiome influences mucosal immune networks.

• Developing therapeutic vaccination

One of the most exciting developments from John Hermon-Taylor's laboratory is the anti-MAP vaccine capable of driving MAP from infected tissues [20]. It is envisaged that the stimulation of immune responses in the CD host, contrary to current immune-suppression, will be another co-therapy in the eradication of the intracellular pathogen/s driving the chronic inflammation in CD.

Conclusion

Current 'global warming' of scientific thought toward a microbial role in CD and seemingly unrelated therapeutic developments listed above augur well for patients with this chronic condition. The focus of therapy will now shift away from control of inflammation and toward control and eradication of the underlying pathogen/s, particularly MAP with restoration of defective immunity.

"The focus of therapy will now shift away from control of inflammation and toward control and eradication of the underlying pathogen/s, particularly MAP with restoration of defective immunity."

Financial & competing interests disclosure

*G Agrawal has filed patents in anti-*Mycobacterium avium *subspecies* paratuberculosis *antibiotics and infliximab combination therapy. TJ Borody has a financial interest in the Centre for Digestive Diseases, where fecal microbiota transplantation is a treatment option. In addition, he has filed patent applications in the field of fecal transplantation and anti-*Mycobacterium avium *subspecies* paratuberculosis *therapies. The authors have no other relevant affiliations or financial involvement with any organization or entity with a financial interest in or financial conflict with the subject matter or materials discussed in the manuscript apart from those disclosed.*

No writing assistance was utilized in the production of this manuscript.

References

1 Campbell J, Borody TJ, Leis S. The many faces of Crohn's disease: latest concepts in etiology. *Open J. Int. Med.* 2(2), 107 (2012).

2 Podolsky DK. Inflammatory bowel disease. *N. Engl. J. Med.* 347(6), 417–429 (2002).

3 Deretic V. Autophagy in immunity and cell-autonomous defense against intracellular microbes *Immunol. Rev.* 240(1), 92–104 (2011).

4 Greenstein RJ. Is Crohn's disease caused by a mycobacterium? Comparisons with leprosy, tuberculosis, and Johne's disease. *Lancet Infect. Dis.* 3(8) 507–514 (2003).

5 Davis W, Madsen-Bouterse S. Crohn's disease and *Mycobacterium avium* subsp. *paratuberculosis*: the need for a study is long overdue. *Vet. Immunol. Immunopathol.* 145(1–2), 1–6 (2012).

6 Nakase H, Tamaki HH, Matsuura M, Chiba T, Okazaki K. Involvement of *Mycobacterium avium* subspecies *paratuberculosis* in TNF-α production from macrophage: possible link between MAP and immune response in Crohn's disease. *Inflamm. Bowel Dis.* 17(2), 140–142 (2011).

7 Bach H, Rosenfeld HG, Bressler B. Treatment of Crohn's disease patients with infliximab is detrimental for the survival of *Mycobacterium avium* ssp. *paratuberculosis* within macrophages and shows a remarkable decrease in the immunogenicity of mycobacterial proteins. *J. Crohns Colitis* 6(5), 628–629 (2012).

8 Selby W, Pavli WP, Crotty B *et al.* Two-year combination antibiotic therapy with clarithromycin, rifabutin, and clofazimine for Crohn's disease. *Gastroenterology* 132(7), 2313–2319 (2007).

9 Behr M, Kapur MV. The evidence for *Mycobacterium paratuberculosis* in Crohn's disease. *Curr. Opin. Gastroenterol.* 24(1), 17–21 (2008).

10 Barash JE, Lipton JE, Barash DP. Flawed Australian CD study does not end MAP controversy. *Gastroenterology* 133(5), 1742; author reply 1745–1746 (2007).

11 Gitlin L, Biesacker J. Australian Crohn's antibiotic study opens new horizons. *Gastroenterology* 133(5), 1743–1744; author reply 1745–1746 (2007).

12 Chamberlin W, Borody WT, Naser S. MAP-associated Crohn's disease: MAP, Koch's postulates, causality and Crohn's disease. *Digest. Liver Dis.* 39(8), 792–794 (2007).

13 Gui GP, Thomas PR, Tizard ML, Lake J, Sanderson JD, Hermon-Taylor J. Two-year-outcomes analysis of Crohn's disease treated with rifabutin and macrolide antibiotics. *J. Antimicrob. Chemother.* 39(3), 393–400 (1997).

14 Efficacy and Safety of Anti-MAP Therapy in Adult Crohn's Disease (MAPUS). www.clinicaltrials.gov/ct2/show/NCT01951326

15 Click RE. A 60-day probiotic protocol with Dietzia subsp C79793-74 prevents development of Johne's disease parameters after *in utero* and/or neonatal MAP infection. *Virulence* 2(4), 337–347 (2011).

16 Click RE. A potential 'curative' modality for Crohn's disease – modeled after prophylaxis of bovine Johne's disease. *J. Mycobact. Dis.* 2, 117 (2012).

17 Zhang F, WangFH, Wang M, Cui B, Fan Z, Ji G. Fecal microbiota transplantation for severe enterocolonic fistulizing Crohn's disease. *World J. Gastroenterol.* 19(41), 7213–7216 (2013).

18 Gordon H, HarbordHM. A patient with severe Crohn's colitis responds to faecal microbiota transplantation. *J. Crohns Colitis* 8(3), 256–257 (2014).

19 Anderson JL, Edney RJ, Whelan K. Systematic review: faecal microbiota transplantation in the management of inflammatory bowel disease. *Aliment. Pharmacol. Ther.* 36(6), 503–516 (2012).

20 Bull TJ, Gilbert SC, Sridhar S *et al.* A novel multi-antigen virally vectored vaccine against *Mycobacterium avium* subspecies *paratuberculosis*. *PLoS ONE* 2(11), e1229 (2007).
