# Supplementary material for: Long History of Queries about Bovine Paratuberculosis as a Risk Factor for Human Health
Source: Pathogens. 2021 Oct 28;10(11):1394. doi: 10.3390/pathogens10111394 (PMC8622788; doi:10.3390/pathogens10111394)
Supplement: Supplementary file 1 [file pathogens-10-01394-s001.zip › CARBONE.pdf]

AMERICAN ACADEMY OF MICROBIOLOGY

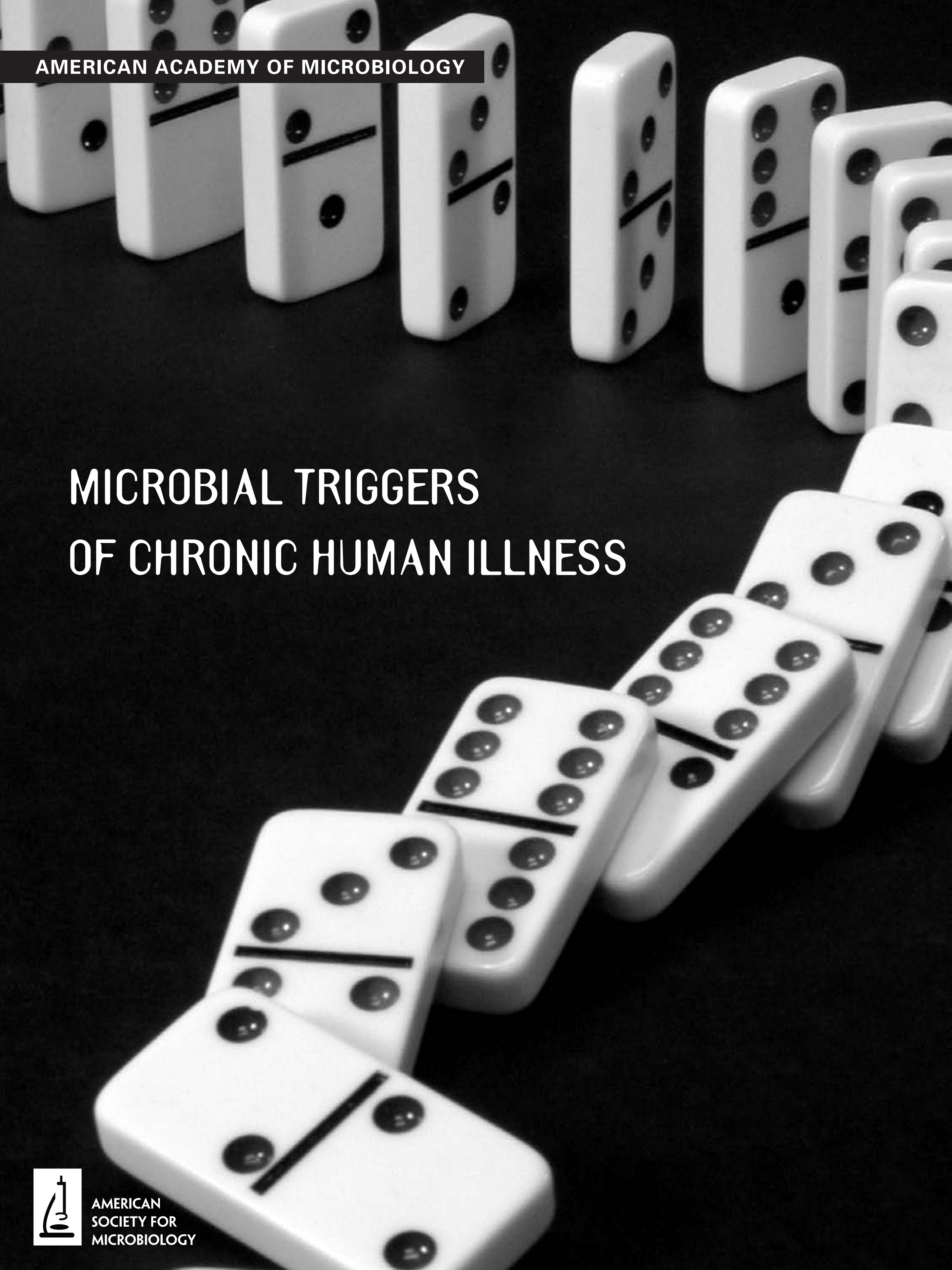

MICROBIAL TRIGGERS OF CHRONIC HUMAN ILLNESS

AMERICAN
SOCIETY FOR
MICROBIOLOGY

Copyright © 2005
American Academy of Microbiology

1752 N Street, N.W.
Washington, DC 20036
http://www.asm.org

This report is based on a colloquium, sponsored by the American Academy of Microbiology, held June 25-27, 2004, in New Orleans, Louisiana.

The American Academy of Microbiology is the honorific leadership group of the American Society for Microbiology. The mission of the American Academy of Microbiology is to recognize scientific excellence and foster knowledge and understanding in the microbiological sciences. The Academy strives to include underrepresented scientists in all its activities.

The American Academy of Microbiology is grateful for the generous support of NIAID, National Institutes of Health (Grant Number: 1 R13 AI059002-01).

The opinions expressed in this report are those solely of the colloquium participants and may not necessarily reflect the official positions of our supporters, the Food and Drug Administration and the Federal Government, or the American Society for Microbiology.

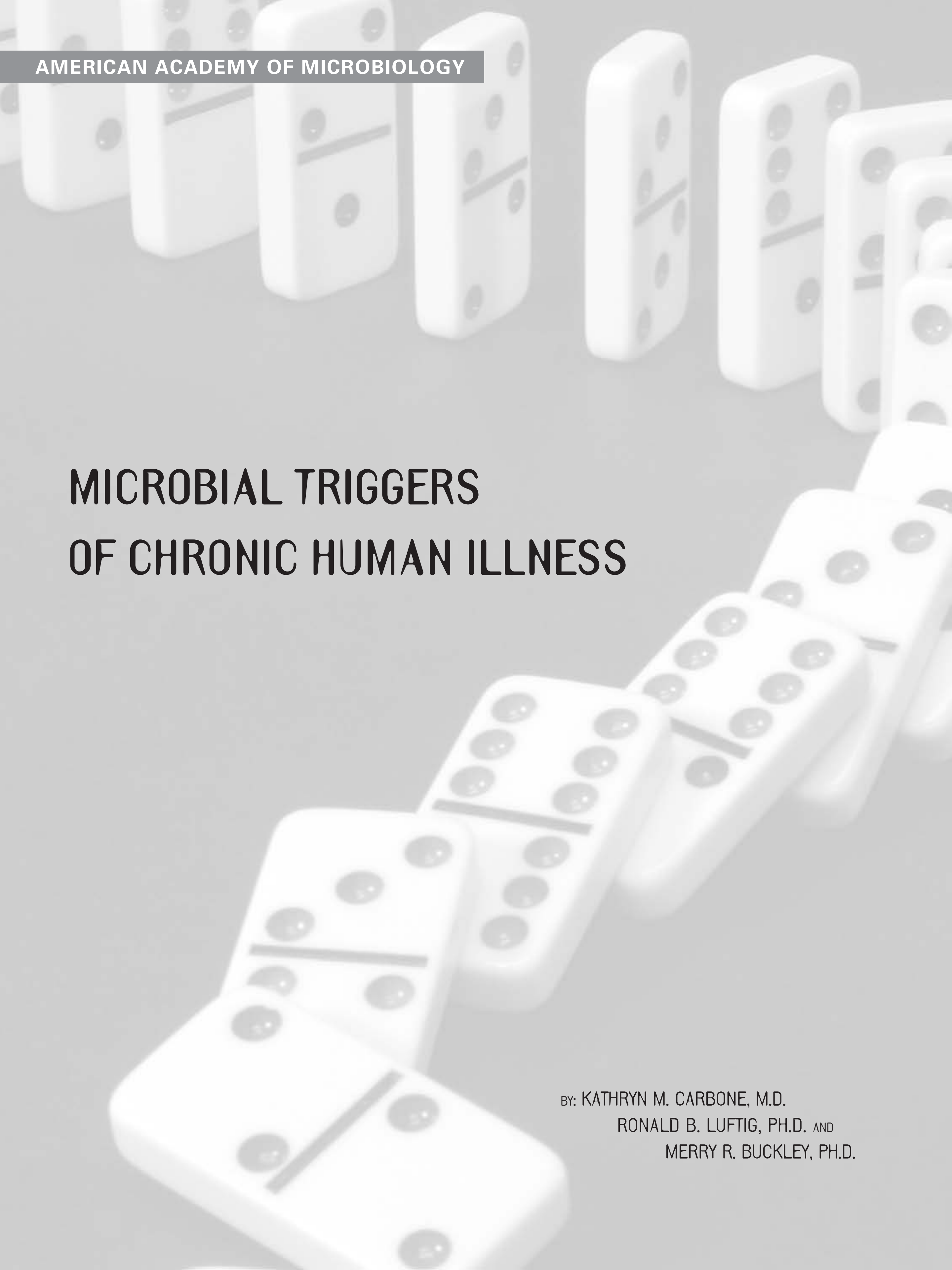

AMERICAN ACADEMY OF MICROBIOLOGY

MICROBIAL TRIGGERS OF CHRONIC HUMAN ILLNESS

BY: KATHRYN M. CARBONE, M.D.
RONALD B. LUFTIG, PH.D. AND
MERRY R. BUCKLEY, PH.D.

Board of Governors
American Academy of Microbiology

Eugene W. Nester, Ph.D. (Chair)
University of Washington

Kenneth I. Berns, M.D., Ph.D.
University of Florida

Arnold L. Demain, Ph.D.
Drew University

E. Peter Greenberg, Ph.D.
University of Washington

J. Michael Miller, Ph.D.
Centers for Disease Control and Prevention

Stephen A. Morse, Ph.D.
Centers for Disease Control and Prevention

Harriet L. Robinson, Ph.D.
Emory University

Abraham L. Sonenshein, Ph.D.
Tufts University Medical Center

George F. Sprague, Jr., Ph.D.
University of Oregon

David A. Stahl, Ph.D.
University of Washington

Judy A. Wall, Ph.D.
University of Missouri

Colloquium Steering Committee

Kathryn M. Carbone, M.D. (Co-Chair)
Center for Biologics Evaluation and Research
U.S. Food and Drug Administration

Ronald B. Luftig, Ph.D. (Co-Chair)
Louisiana State University Medical Center

Michael E. Hagensee, M.D., Ph.D.
Louisiana State University Medical Center

William S. Mason, Ph.D.
Fox Chase Cancer Center
Philadelphia, Pennsylvania

Seth H. Pincus, M.D.
Children's Hospital
Louisiana State University Health Sciences Center

Carol A. Colgan
American Academy of Microbiology

Colloquium Participants

Kathryn M. Carbone, M.D.
Center for Biologics Evaluation and Research
U.S. Food and Drug Administration

Paul Ewald, Ph.D.
University of Louisville

Michael E. Hagensee, M.D., Ph.D.
Louisiana State University Medical Center

William P. Halford, Ph.D.
Tulane University Medical School

Lisa Jackson, M.D., M.P.H.
University of Washington

Martin Kast, Ph.D.
University of Southern California

James W. Lillard, Ph.D.
Morehouse School of Medicine

Ronald B. Luftig, Ph.D.,
Louisiana State University Medical Center

William S. Mason, Ph.D.
Fox Chase Cancer Center
Philadelphia, Pennsylvania

Patrick S. Moore, M.D., M.P.H.
University of Pittsburgh Cancer Institute

Seth H. Pincus, M.D.
Research Institute of Children's Hospital
Louisiana State University Health Sciences Center

David Pisetsky, M.D., Ph.D.
Duke University

Glenn S. Rall, Ph.D.
Fox Chase Cancer Center
Philadelphia, Pennsylvania

Herbert Winkler, Ph.D.
University of South Alabama

EXECUTIVE SUMMARY

The American Academy of Microbiology convened a colloquium on June 25-27, 2004, in New Orleans, Louisiana, to deliberate the microbial causes of chronic diseases. Research professionals from the fields of microbiology, medicine, oncology, vaccine development, immunology, and other related fields participated, and a number of topics related to these pathogens and illnesses were covered. The participants compiled several recommendations for future research efforts.

A number of chronic human illnesses are triggered, either directly or indirectly, by microorganisms. Among these are diseases that many scientists never suspected of having an infectious etiology, including peptic ulcer disease, brought on by the bacterium *Helicobacter pylori*, and cervical cancer, a condition caused by a human papilloma virus (HPV). Scientists have had to apply multiple and different lines of evidence to convincingly link a given chronic disease to a causative agent, employing cultivation, molecular methods of detection, epidemiological studies, preventive intervention studies, transmission studies, animal models, and other methods to compile sufficient evidence. Numerous host factors and microbial factors have been found to come into play in determining the outcome of infections with the pathogens that trigger these diseases.

Other diseases, including some extremely common and devastating conditions, exhibit characteristics that indicate they may be infectious as well. Characteristics that can tip off investigators to a possible microbial cause range from pathogen presence in diseased tissues, to an increased risk of disease in the immune suppressed, to the response of the condition to antimicrobial therapy. A number of complicating factors can make it more difficult for researchers to investigate these putative microbial links. If an infection is ubiquitous in a population, for example, it can be challenging to draw a one-to-one relationship between infection and illness.

Because of the difficulty of applying Koch's postulates to identifying the microbial triggers of chronic diseases, new criteria are needed for establishing proof of associations of this kind.

In releasing the results of research that associates devastating illnesses with infectious causes, some thought should be given to the possible social consequences and impacts on risk-associated behaviors.

In investigating possible microbial links to chronic illnesses, it is extremely important to be able to detect the presence of any pathogens in affected tissues. Currently available technologies for pathogen detection have their strengths, but there is room for improvement. Other technological needs include better markers of early disease states and better information on disease occurrence to help to identify new syndromes and recognize patterns in old syndromes.

Research on chronic infectious diseases can incorporate many different elements, including medical, microbiological, epidemiological, and genetic components. Accordingly, the best research will be multidisciplinary, involving experts from multiple areas of experience to derive the most complete picture of the issues at hand.

KOCH'S POSTULATES

Three rules for experimental proof of the pathogenicity of an organism were presented in 1883 by the German bacteriologist, Robert Koch. A fourth was appended by E. F. Smith (1905). Briefly, these rules state:

1. The suspected causal organism must be constantly associated with the disease.

2. The suspected causal organism must be isolated from an infected plant and grown in pure culture.

3. When a healthy susceptible host is inoculated with the pathogen from pure culture, symptoms of the original disease must develop.

4. The same pathogen must be re-isolated from plants infected under experimental conditions.

These rules of proof are often referred to as Koch's Postulates

Atherosclerosis, diabetes, and Alzheimer's disease are all devastating chronic illnesses that cost millions of dollars in health care every year and exact incalculable tolls of pain and suffering. Atherosclerosis, the hardening and narrowing of the arteries, is responsible for approximately 42 percent of deaths in this country, and currently affects one of every four Americans (CDC). Over 13 million Americans are living with diabetes, the sixth leading cause of death (CDC). Alzheimer's disease is the eighth leading cause of death in the U.S. (CDC), a condition borne by an estimated four million Americans (http://www.alzheimers.org). Each of these diseases was once thought to be caused exclusively by environmental exposures or by genetic predisposition. Now, at least a subset of all of them are suspected of being infectious diseases—illnesses that are spread by viruses, bacteria, microbial parasites, or prions.

Although often difficult to recognize and even more difficult to substantiate, a number of chronic illnesses that are triggered by microbial pathogens have been identified (see Table 1). Some of the clearest, best-supported connections include human papilloma virus (HPV) and cervical cancer; *Helicobacter pylori* and peptic ulcers; hepatitis B and C viruses (HBV and HCV) and hepatocellular carcinoma; *Streptococcus* species and rheumatic fever; human immunodeficiency virus (HIV) and AIDS; and *Mycobacterium tuberculosis* and tuberculosis. Other chronic diseases exhibit characteristics that hint at infectious causes, but they have either not been investigated or have yet to be conclusively verified or refuted (see Table 2). These illnesses include many terrible conditions, like atherosclerosis, diabetes, and Alzheimer's disease, which long have been considered rooted in genetics or environmental factors.

MICROBIAL TRIGGERS

In studying possible links between pathogens and chronic illness, the concept of a microbial *trigger* of disease requires refinement. In this document, a microbial *trigger* is defined broadly to mean any organism that sets in motion or expedites a disease process. Hence, a microbial trigger can bring on disease in any of a number of ways, including persistence as a chronic infection and the induction of destructive host immune responses (see How Microbes Trigger Chronic Illnesses, below). The definition of a microbial *trigger* can play a role in the way illnesses are classified and how we think about disease. For example, in AIDS patients who develop lymphomas, what is the microbial trigger of the lymphoma HIV, which devastates a patient's immune system, or the Epstein-Barr virus, which takes advantage of immune weakness and inserts its DNA into human cells, causing them to form a tumor, or both?

HOW MICROBES TRIGGER CHRONIC ILLNESSES

Microbes are wildly diverse. Pathogens generally accepted as triggers of chronic illnesses reveal this diversity in the range of behaviors they use to cause disease. It is likely that many more unique ways of bringing about disease are employed by pathogens we have yet to recognize.

Some pathogens bring about chronic illness by **maintaining a persistent infection at the site of the disease**:

- HPV serves to illustrate how a pathogen can cause disease by adding its nucleic acids to host cells. Upon infection, the virus enters cervical cells and integrates genes into the host genome, leading to changes that alter the normal cycle of cell division, resulting in uncontrollable division, cellular transformation, and tumor formation.

- In the case of HBV, the host response to infection leads to development of disease. Chronic liver infection stimulates scarring, fibrosis, and cirrhosis, which can lead to liver failure. Mutagenic events, like oxidative damage to host DNA, viral DNA integration, and possibly environmental aggravators like aflatoxins, can contribute to cellular transformation. Replacement of hepatocytes killed by the adaptive immune response to the virus promotes outgrowth of the transformed cells into liver tumors.

- In the case of Kaposi's sarcoma-associated herpes virus (KSHV), infection drives sarcoma formation through the host's autocrine and paracrine responses. Infection induces abnormal cell multiplication that affects both infected cells and uninfected cells in the tissue.

Other pathogens create their respective chronic diseases by **triggering an immune response in genetically susceptible hosts.** Susceptible individuals stage responses that may be either overly vigorous (leading to self-destructive fallout) or insufficient to clear the organism. Crohn's disease, for example, does not result from infection alone, but from the confluence of infection and genetic susceptibility. Susceptible individuals, who carry the NOD2 or TNFR polymorphisms, may respond to certain commensal intestinal flora, stimulating acute inflammation that leads to chronic inflammation and colitis. Similarly, RANTES (ccl5) and CCR5 polymorphisms make certain individuals more susceptible or resistant to HIV-1 and AIDS progression.

Certain pathogens take a "hit and run" line of attack, in which the **disease occurs after the initial infection has cleared** and the agent is no longer present. One example of this type of approach is infection that leads to Reiter's syndrome. The initial gastrointestinal or sexually transmitted infection is relatively short-lived, and the onset of the reactive arthritis syndrome occurs weeks to months following clearance of the initial infection. Infection may trigger disease by inducing a host immune response among genetically predisposed persons, combining the "hit and run" approach with the triggering of responses in genetically susceptible individuals. Hence, pathogens can cause chronic disease by using any one of these strategies in

isolation or in combination. Multiple factors may act in any given relationship between infection and disease.

Other ways in which pathogens can trigger chronic illness include:

- Direct carcinogenesis, in which infection interferes with tumor suppressor mechanisms,
- Indirect carcinogenesis, in which infection induces inflammation that creates an environment leading to tumorigenesis,
- Inhibition of cell division,
- Induction of host protein structural changes,
- Immunosuppression, and
- Direct targeting and impact on cell activity or viability.

Over 90 million Americans live with chronic illnesses, conditions that account for 70 percent of all deaths in the U.S. (CDC). Researching the causes of chronic illnesses, infectious or otherwise, will lead to development of therapies, cures, and strategies for prevention that will affect the lives of millions of individuals every year.

In terms of the number of lives saved and improvement of quality of life, the biggest breakthroughs in medicine have been in grasping the causes of infectious disease and developing ways to prevent those diseases. Hence, it is imperative that research into putative links between microorganisms and chronic illness continue. The findings from that research can readily be applied in saving people's lives.

CONVICTED: CHRONIC ILLNESSES WITH INFECTIOUS CAUSES

In the few years since the discovery that microbes can trigger non-acute diseases, physicians and researchers have learned that a number of bacteria, viruses, and eukaryotic microbes are responsible for a range of well-known chronic human illnesses. Table 1 lists infections and the diseases they can initiate that have been linked to chronic disease by reasonably strong evidence.

MULTIPLE OFFENDERS: CHRONIC ILLNESSES TRIGGERED BY MULTIPLE PATHOGENS

Table 1 reveals that some pathogens can cause more than one chronic disorder. Conversely, more than one microbe can trigger certain chronic diseases. For example, HBV or HCV can induce chronic hepatitis; the histopathological signs of the diseases triggered by these two agents are indistinguishable. For these illnesses, a search for a single culprit will prove frustrating and uninformative.

Certain pathogens have also been observed to cause different diseases in different locations. EBV, for example, only seems to cause Burkitt's lymphoma in equatorial Africa, but not in other parts of the world.

PROVING THE MICROBIAL ORIGINS OF CHRONIC ILLNESSES

It can be extremely difficult to prove that a pathogen is the cause of a chronic disorder, particularly in those instances where the pathology occurs in tissues removed from the primary site of replication or in instances where the onset of disease begins some time after the duration of the infection. Oftentimes, it is not practical or even possible to use Koch's postulates to prove the infectious nature of chronic illnesses.

The first step along the pathway to proving an infectious origin for chronic disease is often tenuous, taken by clinicians or researchers who believe that some aspect of the disease conforms to an infectious model. In the case of Kaposi's sarcoma, for example, an infectious etiology was suspected because of the epidemiological patterns of the disease. It had an unusual geographic distribution, indicating to investigators that the disease could be spread from person to person. These early suspicions then had to be confirmed. It is rare that any single line of evidence is sufficient to prove that a chronic disease has an infectious nature. Proof that a microbial trigger exists for a given chronic illness can only be realized by summing the totality and consistency of many lines of evidence.

Techniques applied and type of evidence required to prove that a given microorganism is at the root of a chronic illness will depend largely on the tissues involved. For example, the gut is inhabited by hundreds of species of organisms; subtle shifts in the balance of these populations may lead to illness. On the other hand, the nervous system, which may be susceptible to microbes that may trigger such disorders as amyotrophic lateral sclerosis, is an otherwise sterile environment. The methods applied to substantiate microbe-disease links in these two types of tissues and the resulting data that can be considered "conclusive" would be fundamentally different.

(The criteria used to ascribe etiology to non-microbial causes like genetics and non-infectious environmental influences are far less rigorous than those applied to identify microbial causes. For example, the autism community has all but concluded that autism is a genetic disease, but the dizygotic twin and sibling concordance studies do not support a dominant role for genetic cause. These potential etiologies need to be held to the same experimental standards for causation as microbial etiologies.)

TABLE 1. CHRONIC DISEASES FOR WHICH THERE IS STRONG EVIDENCE OF AN INFECTIOUS ETIOLOGY.

CHRONIC DISEASE(S)	INFECTION
**Human T-cell Lymphotrophic virus type I**	Adult T cell leukemia
	Tropical spastic paraparesis
**Human papilloma virus (HPV)**	Cervical carcinoma
	Laryngінal papilloma
	Penile cancer
	Anal cancer
	Vulvar and vaginal intraepithelial neoplasia
	Venereal warts
	Common warts
	Head and neck cancer
**Epstein-Barr virus (EBV)**	Burkitt's lymphoma in Africa
	Nasopharyngeal carcinoma
	Hodgkin's disease
	Post-transplant lymphoproliferative disorders
	B cell lymphomas in AIDS patients
**Hepatitis B virus (HBV)**	Hepatocellular carcinoma, chronic hepatitis
**Hepatitis C virus (HCV)**	
**HBV and delta virus**	
**HBV**	Polyarteritis nodosa
**HCV**	Mixed cryoglobulinemia
**Measles**	Sub acute sclerosing panencephalitis
**Kaposi's sarcoma-associated herpes virus**	Multicentric Castleman's disease
	Lymphoma
	Kaposi's sarcoma
**Parvovirus B19**	Anemia; arthritis
**Rubella**	Post-rubella arthritis syndrome
	Congenital rubella syndrome
**Prions**	Creutzfeld Jacob disease
	Kuru
	Familial insomnia
Helicobacter pylori	Gastric lymphoma
	MALT lymphoma
	Peptic ulcer disease (PUD)
**Histoplasmosis**	Chronic pericarditis
**Syphilis**	Tertiary and neurosyphilis
Borellia burgdorferi	Lyme disease
**Group A *Streptococcus***	Post-streptococcal glomerulonephritis
	Rheumatic fever
Chlamydia trachomatis	Reiter's syndrome and reactive arthritis
Tropheryma whippleii	Whipple's disease
Mycobacterium leprae	Leprosy
Mycobacterium tuberculosis	Tuberculosis
Campylobacter jejuni	Guillan-Barre syndrome
Chlamydia trachomatis	Pelvic inflammatory disease
**Osteomyelitis**	Squamous cell carcinoma
***Escherichia coli* O157:H7**	Hemolytic-uremic syndrome
**Cytomegalovirus**	Post-transplant accelerated atherosclerosis

Detection of a Pathogen at the Site of Disease

In some cases, once researchers started looking for an infectious cause to a chronic disease, conclusive evidence for an infectious link was not difficult to find. The most straightforward evidence for an infectious origin to a chronic disease is obtained when the pathogen is detected at the site of disease. An example is Whipple's disease—a condition that was linked to the bacterium *Tropheryma whippleii* when bacilli were found in diseased tissue. The discovery of this pathogen in diseased tissue led to the realization that this illness could be managed using antibiotic therapies, and the effectiveness of this therapy, in turn, provided further evidence of the microbial origin of the disease.

Pathogens have also been identified by using molecular techniques, such as PCR, to look for signature microbial genetic sequences like ribosomal RNA genes in infected tissues. In some cases, it has been possible to evaluate the relative numbers of a pathogen within and outside the affected site, revealing a biological gradient between diseased tissue and normal tissue. Such was the case with investigations of Kaposi's sarcoma lesions—herpes virus particles occurred in greater numbers within affected tissues than in other areas of the body.

Detection of an organism in diseased tissue does not necessarily prove causality. There is a possibility that the organism may be an "innocent bystander," present at the site but not a factor in the disease. False positive results, in which the test indicates a given organism is present when it actually is not, can also mislead researchers. Conversely, failure to detect an organism using PCR-based methods does not necessarily mean that a microbial linkage does not exist; it is possible that the method applied is not sufficiently sensitive to detect viruses or bacteria present in very low numbers in the tissue. Alternatively, a causative organism may have initiated the illness and subsequently departed the target tissue, or initiated illness remotely, in a location other than the site of infection.

Connecting the Dots: Epidemiological Approaches

Epidemiological associations have also proven to be powerful tools in ascribing cause-and-effect relationships between pathogens and chronic illness. Studies comparing the risk of a given chronic disease between people who have been identified as infected and their uninfected counterparts have provided strong evidence in support of causation for many of the diseases that are now known to have a microbial trigger, like stomach cancer, which is often caused by *Helicobacter pylori*, and Kaposi's sarcoma, caused by HHV 8.

Epidemiological studies rely on a number of methods to determine the infection status of the study subjects. These methods include PCR-dependent approaches to detect pathogen DNA in target tissues and serologic methods, like measurements of blood antibody levels. Prospective and retrospective serologic studies have been particularly useful in investigating links between pathogens and chronic illness.

Limitations on the usefulness of serologic testing in epidemiological studies exist. Oftentimes, sensitive and specific serologic tests do not exist for the organism of interest. Moreover, serologic evidence of past exposure does not necessarily imply current infection or, for that matter, past infection of the target tissues. Antibody levels may also decline over time, so seronegative individuals may have had a past infection. Ubiquitous infections are also difficult to evaluate using epidemiological approaches, either in conjunction with serological methods or otherwise (see Features That Make a Microbial Diagnosis Problematic).

Preventive Interventions and Treatment Studies

Researchers can also evaluate the outcomes of preventive interventions and antibiotic treatments to determine whether a microbial cause for a chronic illness exists. These data can include the results of ecologic studies, randomized controlled trials, or cross sectional evaluations.

Antibiotic treatment studies are underway to evaluate the association between *C. pneumoniae* and atherosclerosis, including several randomized control trials of antibiotics for secondary prevention of coronary heart disease events. In this study, the treatment is not specific to Chlamydia, so an effect of treatment may not be due to an effect against Chlamydia. Conversely, the lack of an effect does not exclude the possibility that an organism is involved in the disease process, because a pathogen may not be equally susceptible to treatment at all stages of infection.

Transmission Studies

Transmission studies examine the effects of deliberate, experimental infection on study subjects. Although they do not meet necessary standards for safety and ethical acceptability, these studies occasionally have been employed in documenting the risk of disease following infection and in defining the temporal relationship between infection and disease. In evaluating the connection between *Helicobacter pylori* and peptic ulcer disease, a researcher associated with the project ingested a cocktail of the suspected pathogen. Hence, it was possible to document that disease followed infection and, importantly, that the disease could be resolved with antibiotic treatment.

Animal Models

Animal models can be very useful in studying chronic illnesses and their origins but because of basic differences in the biology of animals and humans, drawing conclusions about human disease from model data is not always possible. Given the problems in generalizing from animals

to humans, successfully proving a link to chronic illness in an animal model is not sufficient, on its own, to establish causality. However, we can use animal models to generate hypotheses, and they can be useful adjuvants to other sources of evidence in building an argument in favor of causality. Hamster models for simian virus 40 (SV40) infection and the development of mesothelioma, for example, have supported the hypothesis that infection can have a carcinogenic effect in humans and added to the totality of the evidence linking the virus to cancer.

HOST FACTORS THAT CONTRIBUTE TO ILLNESS

The outcome of confrontation between a human body and a pathogenic microorganism is determined by what each of the players brings to the fight. Humans can be made more or less susceptible to an initial infection or to the onset of the chronic illness by any of a number of intrinsic or extrinsic factors, including:

- Genetic factors. Certain diseases occur at a higher rate or exclusively in persons with defined genetic characteristics, while other genetic markers can confer resistance. For example, individuals with the leukocyte antigen HLAB27 are at a higher risk of developing Reiter's syndrome following infection with one of the triggering pathogens. Many other examples of the impact of host genetics on microbially-triggered illnesses also exist (see Box A). The role of host genetics in microbially-triggered chronic disease is absolutely interlinked with the geographic distribution of human populations. Co-evolution between hosts and pathogens can influence the incidence of the pathogen and the susceptibility of the population to chronic disease.

- Concomitant infections. Infection with one agent can increase the susceptibility of a host to infection by other pathogens that cause chronic disease. For example, infection with *Chlamydia trachomatis* can increase susceptibility to pelvic inflammatory disease.

- Age. The age at which an individual contracts an infection can play a role in the outcome. Young people who contract gastritis as a result of infection with *H. pylor*i are at a lower risk of duodenal PUD than people who acquire the infection later in life.

- Dose. The size of the inoculum or the extent of the infected tissue can determine whether a chronic illness follows infection.

- Gender. Men and women experience different risks for certain chronic illnesses with microbial etiologies. For example, the risk of developing hepatocellular carcinoma as a result of hepatitis C infection is believed to be elevated in men.

- Hormonal factors. Sex hormones, like estrogen, progesterone, testosterone, and androgen, and stress hormones, like cortisol, ghrelin, and leptin, have been shown to impact the immune response to a number of different pathogens.

- Immune status. Immune suppression increases the risk of infection by many of the organisms known to cause chronic diseases. Moreover, immunologic history can play a role in susceptibility; past exposure to an organism can be protective against subsequent infection.

- Nutritional status. Malnutrition is a decisive factor in host susceptibility.

- Behavioral factors and exposure to non-infectious agents. Choosing to engage in certain activities can greatly increase the likelihood of infection by certain agents. Tobacco, for example, can dampen the body's immune reactions, making an individual more susceptible to any of a number of different kinds of infection. Intravenous injection of illicit drugs can expose an individual to numerous different pathogens if needles and supplies are shared. Moreover, exposure to ultraviolet radiation can make the eye more susceptible to conjunctival carcinoma, a condition that may be triggered by human papilloma virus.

Box A. Malaria and susceptibility to infections that cause chronic illness.

The evolutionary pressure exerted by malaria has resulted in several genetic abnormalities in humans: sickle cell trait, thalassemia, glucose 6 phosphate dehydrogenase (G6PD) variance/deficiencies, and diminished RANTES (Regulated on Activation, Normal T-cell Expressed and Secreted) expression. In an interesting circular interplay of pathogens and human evolution, these genetic aberrations offer a modicum of protection from malaria, but, insidiously, they also make carriers more susceptible to other microorganisms, many of which can cause chronic disease.

The sickle cell gene, HbSS, is particularly problematic. Extremely common among people of African descent, a single copy of HbSS confers a certain amount of resistance to malaria. However, individuals who carry two copies of the gene, in addition to suffering from the crippling effects of sickle cell anemia, suffer from excess iron in the bloodstream. Hepatitis C, B, D, and E viruses can take advantage of this surplus iron, infecting sickle-cell patients at higher rates than the general population. Sickle-cell patients are also more susceptible to Chlamydia, Mycoplasma, Hemophilus, Pneumocystis, and Streptococcus, all of which have been implicated in triggering chronic diseases.

In managing these factors to reduce the incidence of a given chronic illness, researchers must first determine the factors that are the primary drivers in the development of disease. These factors must then be linked logically to the pathogen that is suspected of causing the disease.

In linking a chronic illness to a microbial infection, researchers should make full use of the substantial pool of individuals who consume daily regimens of pharmaceuticals. These drugs, including statins, anti-inflammatories, immune suppressants, and others, may substantially alter the outcome of exposure or infection, and the medical histories of their users can serve to indicate the underlying mechanisms of a disease.

As medicine becomes more and more tailored, both behaviorally and genetically, to the individual, the information technology infrastructure to acquire and interpret information related to disease outcomes must also emerge. The information associated with applying tailored drug regimens could be valuable in assessing host factor-mediated susceptibility to chronic diseases.

The timescale over which host-related factors are significant to the outcome of an exposure or infection remains unclear. Could an event at age 10 play into a disease that becomes manifest at age 20, 40, or 60? Research in this field must begin to establish connections over long temporal intervals.

Defining the roles of genetic markers and microbial triggers in cases of chronic illness

Clearly, host genetics can make all the difference in determining whether an individual becomes ill or not when confronted with a pathogen that causes chronic illness. Epidemiological studies have proven to be indispensable to uncovering instances where a host's genetic susceptibility to a given pathogen is particularly critical. In cases in which a large number of individuals are infected by a pathogen but only some develop a chronic illness, HLA mapping, which identifies histocompatibility antigens, can also identify susceptibility alleles in the population. Resolving the respective roles of microorganisms and genetic markers is critical to managing disease at the public health level as well as to developing treatment strategies for individual patients.

MICROBIAL FACTORS THAT CONTRIBUTE TO ILLNESS

Host factors are not the only consideration in the outcome of a conflict between a human and a chronic disease-causing microbe; the profile of the microbe is the other key determinant. The most critical characteristics include:

- Viral integration. The ability of a virus to integrate its genetic information into the host's genome can have an impact on whether the virus will have a lasting, chronic effect on the host.
- Latency factors. The ability to establish latency, a lag period after infection and prior to the development of pathogenesis and disease, appears to enable some microbes to cause a chronic disease.
- Ability to bind to mucosal surfaces or other tissues. The likelihood that a microbe will infect a given individual is proportional to its ability to persist in the body.
- Characteristics of the target organ. Some organs are apparently more susceptible to chronic disease than others are. For example, the liver and brain may have immunosuppressive activities that prevent these organs from directing immune defenses to microbial offenders.
- High mutation rate. A microbe's ability to transform and disguise itself under the pressure of the immune system can be a great asset in escaping from a host's defenses.
- Immune evasion. Certainly, the ability of the microbe to elude the host's immune defenses determines whether that organism will persist long enough to cause disease. The ability to engage in molecular mimicry of the host is one quality that influences immune evasion. Certain organisms are capable of imitating the molecular signature of the host, allowing them to "hide out" right under the watchful gaze of the immune system.

Certain pathogens, like negative strand RNA viruses, are known to mutate readily within the host. Others, like adenoviruses and papillomaviruses, can have multiple genotypes. The distinct characteristics of mutants and alternate serotypes should be considered when determining the microbial attributes that impact disease.

Thorough knowledge of the lifestyle of a given pathogen, its mode of infection and ecology, is crucial to understanding how these organisms bring about disease. The exploration of pathogens through rigorous basic science is highly recommended.

PRIME SUSPECTS: CHRONIC ILLNESSES SUSPECTED OF HAVING AN INFECTIOUS ETIOLOGY

Investigations that have convincingly linked chronic diseases with microbial triggers all began with the same step: someone grew suspicious about the circumstances surrounding the disease and investigated further. A number of different types of observations point to a possible microbial etiology and elicit this type of suspicion. They include:

- The presence of pathogens or pathogen genes in diseased tissues. Finding evidence of a pathogen at the site of disease is a significant suggestion of causation.
- Response of the condition to antimicrobial therapy. A partial response to antibiotic therapy is evidence for a microbial cause, but it could also be due to the modification of disease cofactors, including concomitant infections.
- The disease sets in after a persistent infection. Infection in the target organ or another part of the body when followed by a chronic disease is a key indicator of a microbial trigger.
- An increased risk of disease in immune suppressed individuals. This indicates that the immune system can play a role in protection, and therefore, that the causative agent is likely external, and possibly microbial.
- Host responses suggest infection. A host may exhibit adaptive immunity, necrosis, inflammation, neoplasia, or other pathological characteristics consistent with infection. For example, some diabetes cases are suspected of being brought on by infectious disease because the immunological characteristics of the patients' pancreases are consistent with infection.
- The disease is multifocal, involving more than one organ. Parvovirus B19 can infect reticulocytes, bone marrow, and the liver. *Trypanosoma* cruizi can lead to chronic megacolon, megacardia, and megaesophagus.
- Epidemiological patterns suggest an infectious cause. The incidence of the chronic disease may be greater in certain groups or geographical locations, suggesting that the illness can be passed from person to person. Alternatively, the incidence may increase without known explanation (as is the case in recent years with asthma) or may have some other temporal attribute.
- The disease is associated with inflammation. Inflammation is often a sign of infection.
- Similar diseases are known to be infectious. Rheumatoid arthritis, for example, has many features in common with Lyme disease, a condition caused by a bacterial pathogen suggesting that it too may have a microbial origin.
- Animal models suggest an infectious origin. Crohn's disease is suspected of having an infectious etiology because of inflammatory conditions observed in animal models. *Mycobacterium avium* subspecies *paratuberculosis* triggers Johne's disease in cows and *Clostridium* species trigger ileitis in chickens, both of which are similar to Crohn's disease.
- The disease is idiopathic. If there is no other likely explanation for a chronic disease, the possibility of a microbial cause is often investigated.
- The disease is associated with granuloma formation. Granulomas often form in response to microbial insults.
- Unexplained fever coincident with chronic illness. Fever is often a feature of infection.
- The occurrence of illness flare-ups or a pattern of relapse and remission.

There are caveats in interpreting these signals; none is absolutely reliable on its own. For example, instead of denoting the influence of a microbial trigger, many of these characteristics can instead indicate that a pathogen is exacerbating a preexisting, noninfectious condition.

SIGNATURES OF MICROBES THAT CAN TRIGGER CHRONIC ILLNESS

Among the pathogens that cause acute illness, certain characteristics indicate the capacity to establish the kind of persistent infections that can lead to chronic illness. These features include:

- The ability of the organism to trigger apoptosis (cell death),
- The ability of the organism to elicit innate immunity,
- The ability to trigger acute inflammation and adaptive immunity, and
- The ability of the organism to transform host cells, inserting the microbe's genes into the host's genome.

CHRONIC ILLNESSES SUSPECTED OF HAVING AN INFECTIOUS ETIOLOGY

A number of chronic illnesses exhibit one or more of the possible signs of an infectious cause listed above. Some of these putative links have been investigated, but sufficient evidence to support or disprove an infectious etiology for them has not been amassed.

FEATURES THAT MAKE A MICROBIAL DIAGNOSIS PROBLEMATIC

Investigating the possible infectious causes of chronic conditions is almost always a complicated endeavor, but certain features can make demonstrating an organism-disease connection particularly problematic:

- **The infection is ubiquitous**. Many people are infected with the Epstein-Barr virus, for example, but very few of these individuals develop lymphoma as a result. Hence, a one-to-one relationship of infection to illness is lacking, complicating efforts to correlate the two. In cases like this, it may be that disease results from a combination of infection and downstream factors, such as genetics or environmental exposures.

- **The chronic disease is temporally disconnected from the infection**. Pathogens with a long latency period, like HIV (AIDS) or *Borellia burgdorferi* (Lyme disease), are more difficult to link to an illness than pathogens that initiate disease more rapidly.

- **The disease is a complicated syndrome or lacks an accurate biomarker.** If it is difficult to accurately identify the disease in a patient then it can be difficult to draw ties to a causative microbe. Examples of such complex conditions include chronic fatigue syndrome and schizophrenia.

- **The infection can be linked to the disease only in the presence of an associated condition**. The existence of another condition can significantly complicate analyses.

- **The target tissues are relatively inaccessible**. In cases where the brain or coronary arteries are the target of disease, for example, efforts to identify infected and uninfected individuals are difficult.

- **The causative organism is not detectable by current methods.**

- **The disease is not due to persistent infection**. In some cases, the illness can set in after the organism has apparently been cleared, so the organism is not present in diseased tissues at the time of analysis.

- **The disease occurs at a nonsterile site**. Disease in the gut, for example, makes detecting an association with a single organism (or group of organisms) very difficult.

TABLE 2. CHRONIC DISEASES FOR WHICH THERE IS SUSPICION OF AN INFECTIOUS ETIOLOGY

DISEASE	SUSPECTED AGENT(S), IF ANY
**Primary biliary cirrhosis**	*Helicobacter pylori*, retrovirus
**Mesothelioma**	Simian virus 40
**Multiple sclerosis**	Epstein-Barr Virus
**Tics and Obsessive Compulsive Disorder**	Group A *Streptococcus agalactiae*
**Obsessive compulsive disorder**	Group A *S. agalactiae*
**Crohn's disease**	*Mycobacterium paratuberculosis* and others*
**Alzheimer's disease**	*Chlamydia pneumoniae*
**Diabetes**	Enteroviruses
**Sjogren's disease**	*H. pylori*
**Sarcoidosis**	*Mycobacterium* species
**Atherosclerosis**	*C. pneumoniae,* CMV
**Bell's palsy**	Herpes Simplex Virus
**Schizophrenia**	Intrauterine exposure to Influenza
**ALS**	Prions
**Chronic fatigue**	HTLV-1; EBV
**Prostate cancer**	BK virus

** Clostridium, Campylobacter jejuni, Campylobacter faecalis, Listeria monocytogenes, Brucella abortus, Yersinia pseudotuberculosis, Yersinia enterocolitica, Klebsiella spp., Chlamydia spp., Eubacterium spp., Peptostreptococcus spp., Bacteroides fragilis, Enterococcus faecalis, and Escherichia coli*

APPEALING TARGETS FOR DISCOVERY

Many chronic diseases lack dominant causal hypotheses. Where a hypothesis does exist, it is often so generic that it is uninformative regarding mechanism and treatment and offers little substance on which to base inquiries. Hence, in order to develop useful hypotheses, rigorous, active consideration of all possible etiological explanations must be considered in a process that involves input from investigators of many disciplines.

Research to identify the possible microbial triggers of chronic illness should focus on those diseases that have the greatest impact on quality of life, like cancer, atherosclerosis, diabetes, and autism, in order to produce the greatest increase in high quality days for treated patients.

It would benefit researchers to agree upon a definitive list of the patterns that indicate the influence of a microbe in a given chronic illness. Based on the amount of existing supporting evidence, new hypotheses about infectious agents and disease can be placed in categories that describe the anticipated difficulty of resolving the possible linkage. The amount of data and stringency of proof required to qualify for a category would increase as the categories go from "difficult" to "easy" and the linkages become easier to establish conclusively. Placing microbes and chronic diseases of unknown etiology on such a spectrum will help to define the outstanding questions that need to be addressed to confirm or exclude causality. Those conditions that are most likely to have a microbial etiology should be high priorities for research.

It is strongly recommended, for the safety of patients and for the sake of data analysis, that experimental application of antibiotic therapies to cure chronic conditions be attempted only in clinical trial settings. Physicians offering nontraditional therapies are encouraged to report their observations so the data can be subjected to independent evaluation and comparison to outcomes of similar efforts can be made. (In investigating putative links between microbes and chronic illness, it should be remembered that the desirable endpoint is not necessarily to determine the causative organism, but to cure or prevent the disease. A therapy that works, even if its mechanism of action is not clear, has obvious value.)

The "low hanging fruit"—chronic diseases with readily apparent links to pathogenic microorganisms—have already been thoroughly investigated. The large number of remaining putative associations are vaguer and will be more difficult to prove or disprove. New approaches that move beyond a strict adherence to Koch's postulates are needed to investigate these potential links.

BEYOND THE KOCH APPROACH: NEW METHODS FOR PROVING OR DISPROVING CAUSATION

Koch's postulates, still seen by many as a necessary tool for proving causative links between microbes and disease, is not equal to some of the particular problems of chronic illnesses. Many of the causative organisms involved in chronic illness cannot be cultivated using recognized techniques, and others position themselves in tissues that are difficult to access for sampling, making them hard to detect in the afflicted. In some cases, the span of time between infection and disease onset is too long to carry out meaningful studies of the effect of reintroduction. New tools must be developed to circumvent these problems and criteria must be established against which evidence of causality can be measured in cases where Koch's postulates are not appropriate.

A database for compiling anecdotal evidence of linkages between pathogens and chronic illness could be used to build a convincing case. For example, nontraditional treatment of a chronic disease with antimicrobials that has a beneficial effect on the condition would be a powerful (if nonspecific) proof of causation. The database would provide an opportunity to report positive outcomes; information from a single case in which a benefit was observed could be captured and added to the totality of the current evidence. The process of submitting data to the database would require formalization, possibly by a strategy

Epidemiologic criteria, such as Hill's criteria, established to measure the evidence that smoking causes lung cancer, could be employed for proving causative links between microbes and chronic illnesses as well. These criteria include (1) consistency of the association, (2) strength of the association, (3) presence of a dose response relationship, (4) specificity of the association, (5) plausibility, and (6) exposure preceding disease. Modern revisions of Hill's criteria have been proposed to incorporate molecular approaches to pathogen identification. A similar adaptation may be applicable to the problem of linking chronic illness and pathogens.

Reproducibility of results obtained by different investigators in different labs could be used as a criterion for proving causative links between microbes and illness, as could the **effectiveness of preventive interventions** like vaccines or antibiotics. The **strength of epidemiological links**, such as correlations between infection rates and risk of chronic disease in different populations or geographical locations or over time, can also be a criterion.

similar to that used in the AVERSE (Vaccine Adverse Events Reporting System) database, in which MDs report adverse reactions to vaccines.

POSSIBLE CONSEQUENCES OF UNEARTHING AN INFECTIOUS ETIOLOGY FOR A CHRONIC ILLNESS

Although there are no disadvantages to acquiring more knowledge about those diseases triggered by pathogens, there may be disadvantages to conveying that information to the public if not handled carefully. If a feared disease is found to have an infectious etiology, then there may be negative repercussions for the afflicted, as is the case with victims of leprosy, for example.

Complacence can also pose a problem. If the disease is perceived as easily curable with a round of antibiotics, members of the public may engage more in risk-taking behaviors, reasoning that a cure is readily available. Or, in the case of diseases for which microbial infections account for only a portion of the total number of cases, there may be an inclination among the public and health professionals to ignore other, noninfectious bases of the disease. This could be perilous; if a particular case of disease is brought about by another cause, it may be incurable.

Like any endeavor in biology today, progress in determining the causes of chronic human illness is governed by technology. The existing methods for pathogen detection have their own assorted strengths and weaknesses, and a number of outstanding technological needs exist.

CURRENTLY AVAILABLE TECHNOLOGIES FOR PATHOGEN DETECTION

Although a number of powerful assays exist for the detection of pathogens in human tissues (see **Table 3**), the limitations of these methods must be taken into account. Data from assays that identify pathogen proteins or nucleic acids, in particular, must be interpreted with care. It should be remembered that failure to detect these signals is not necessarily proof that the pathogen of interest is not involved.

High throughput assays are continually being developed and refined, but their usefulness is constrained by the tools and approaches available for data analysis. Exploration of the infectious nature of chronic illnesses often requires a combination of traditional and high throughput approaches. Combining these approaches, and coordinating and interpreting the data streams they provide, will often require effective collaboration between teams of researchers with different sets of skills.

Due to the challenges associated with establishing pathogens as the cause of chronic disease, mechanisms should be developed to handle conflicting theories and evidence likely to arise on the path to truth. When different laboratories obtain conflicting results, the source of the inconsistencies must be discovered. Methods should be compared and potential sources of error should be explored. Shared reagents, blinded samples, and independent validation by third-party investigators will help to resolve differences in data interpretation; however, as yet unrecognized geographic differences in pathogen and host populations or in environmental factors could be the true underlying cause of such inconsistencies.

In light of continual advancements in technology, researchers must take care to regularly revisit putative microbe-disease links, particularly if a reasonable etiology is not found in the interim. As laboratory techniques change and become progressively more sensitive, it is likely that formerly intractable areas of study will become amenable to research.

TECHNOLOGICAL NEEDS

Detection of microorganisms in affected human tissues poses the greatest technical stumbling block to progress in linking microbes to the chronic illnesses they cause. One tool that can be envisioned is a "pathogen chip," a microarray that holds conserved genes belonging to many of the pathogens that are thought to initiate chronic disease.

Although detection of a pathogen does not prove causality, evidence gained using an array could be an important first-pass filter for supporting the need for further research. Microarrays of host factors, pathogen polymorphisms, and gene expression would also be helpful.

Other technical advancements that would improve the ability to detect pathogens in tissues include increased sensitivity of microbial culture methods and increased ability to culture diverse viruses. Non-invasive, high-resolution imaging techniques for detecting microbes or for detecting abnormalities that are indicative of chronic infection, and more specific serological methods for identifying chronic infections are also needed.

In general, more bioinformatics tools for syndrome surveillance of chronic illnesses that may have an infectious etiology are needed, as are computational biology solutions.

Better markers of early disease states and better information on disease occurrence would help to identify new syndromes and recognize patterns in old syndromes.

Access to well-characterized sets of tissues would also prove useful to researchers, particularly tissues from patients who died during, but not because of, chronic illness. Autopsy specimens could serve as an important resource of the appropriate tissues.

TABLE 3. EXPERIMENTAL METHODS FOR DETECTING PATHOGENS IN HUMAN TISSUES.

METHOD	COMMENTS
**Consensus PCR**	**Advantages:** Can detect a wide variety of viruses and bacteria
***In situ* PCR**	**Disadvantage:** May be poorly reproducible
**PCR detection and characterization of conserved sequences**	**Advantage:** The specific organism does not need to be known, although one needs to suspect bacteria
**Representational difference analysis**	**Advantage:** No need to hypothesize about the type of virus
**PCR detection using organism specific primers**	**Advantages:** Highly sensitive, requires small amounts of tissue<br>**Disadvantages:** Need to know the organism to target
**Microbial culture**	**Disadvantage:** Many organisms are not cultivatable<br>**Advantage:** If the organism can be cultured, this method allows further characterization
**Microarray assays, e.g. Virochip**	**Advantage:** Allows rapid testing for many prospective pathogens
**Serologic testing**	Cross-reactive serologic testing can be used if the specific organism has not been identified. A pattern of reactivity in the serum is used to identify the class of agent that may be responsible
**Labeled viruses**	To follow tissue profiles
**Immunohistochemistry**	To locate microbes in tissue
**Histology**	Followed by *in situ* hybridization
**Animal transmission studies**	Important for prion diseases and cancer models for chronic diseases

COLLABORATION, FUNDING, AND THE ROLE OF PROFESSIONAL SOCIETIES

COLLABORATION

The issues surrounding research into possible microbial links to chronic disease are complex, and professionals from many different fields can participate meaningfully in these projects (see Box B). In resolving causal links between chronic disease and microbes, it is important to build evidence from multiple layers of techniques, including epidemiological, histopathological, clinical, and molecular tools. The evidence must be sufficiently compelling that scientists of diverse backgrounds can agree on its finality. Establishing these convincing links is best accomplished by collaborative research that brings together scientists of diverse specialties. However, the most critical collaborative relationships are between research scientists and clinicians, who can combine knowledge about pathogenicity and public health with lessons learned at the front lines of medicine, in hospitals and doctors' offices. These collaborations need to become routine.

Effective collaborations will require careful cultivation in the form of basic cross-education to promote appreciation of the powers of each knowledge set, both clinical and research-based. Exposing Ph.D. candidates to clinical settings could help to cross-fertilize research efforts and medical practices. Inviting junior scientists, like post-docs and senior graduate students, to participate in meetings and discussions about collaboration could jump-start research activities and promote the professional development of the next generation of scientists.

International collaborations are important as chronic diseases do not respect international boundaries, and unique insights can arise from observations made by employing a diversity of approaches and by observing a diversity of clinical settings. Also, a number of the chronic diseases that are due to microbial infection occur only in specific regions of the world, but progress made in understanding one microbe-disease link could generate breakthroughs in the study of others, so scientists of all nations should be encouraged to stay in touch. International collaborations would also facilitate access to genetically diverse and geographically distinct populations. Medical recordkeeping in other countries (including many European nations) is better than in the United States, providing resources for tracking disease that cannot be obtained domestically.

Box B. Other professionals who can contribute to the exploration of links between microbes and chronic illness:

- Bioinformaticists,
- Imaging specialists,
- Geneticists,
- Epidemiologists,
- Physicians,
- Statisticians,
- Immunologists,
- Public health officials, and
- Pathologists.

FUNDING ISSUES

Financial incentives are the key to promoting effective research, but the competition for federal research dollars is fierce. Money often follows good science, so supporting and promoting the excellent (and funded) research of high-profile individuals in the field would help to focus attention on the pressing needs.

Major revolutions in the allocation of research funding have come about when an alarmed public has applied legislative pressure. HIV, biodefense, and autism are three recent examples of concerns that have caught the public's imagination and inspired change. Chronic illnesses are also of significant importance to public health. Finding the microbial triggers of certain of these diseases could save lives and countless years of pain and suffering. The power of public opinion should be brought to bear on addressing the causes of these illnesses.

One obstacle to funding research on microbial links to chronic illness is the fact that the best work in this field is multidisciplinary and does not produce quick answers. Because of the diversity of the contributing fields, this research lacks an ideological "home" among the U.S. federal funding agencies. The key funding agencies need to be made aware of the importance of supporting this work, even if it is outside the strict confines of their expressed priorities. This may pave the way for the establishment of study sections equipped to evaluate the proposed work.

Also a problem is the current funding climate, in which studying acute pathogens of limited clinical impact (in terms of number of patients affected) is more easily funded than studying the role of infections in chronic diseases, which may have a much larger impact on public health. Investigations of the causes of chronic illnesses are often dismissed as "fishing expeditions."

THE ROLE OF PROFESSIONAL SOCIETIES

Medical professionals who deal with patients on a day-to-day basis must be kept informed about progress in linking chronic illness to microbial triggers. Communicating these issues to clinicians is one way that professional societies can contribute to improving the treatment of these diseases.

Societies can also convene colloquia, like this one, to review the evidence in controversial areas, like possible links between SV40 and mesothelioma. Finally, professional societies can have a very powerful voice in promoting awareness, acceptance, and, most importantly, action,

when an association between chronic disease and pathogens appears to be overwhelming.

- Many thousands or millions of people are administered ongoing regimens of pharmaceuticals that can alter their metabolic profiles, and hence, their susceptibility to pathogens and chronic illnesses, substantially. The medical experiences of these "host modified" individuals could serve as an important resource for researchers exploring the underlying mechanisms of the chronic illnesses that may have microbial origins. These records should be put to better use in research.

- In order to produce the greatest increase in high quality days for treated patients, research on the putative microbial causes of chronic disease should focus on those conditions that pose the greatest threat to the quality of human life.

- Interested parties, including researchers, clinicians, and public health officials, should develop a system for classifying chronic diseases of unknown etiology according to the strength of the existing association between the disease and microbial influences. A spectrum of categories will help to identify those diseases that are most likely to have a microbial etiology, and hence, would yield experimental results most easily.

- The challenges of identifying causal links between microorganisms and chronic illnesses requires close teamwork between researchers and clinicians. In order to foster effective collaborative relationships, clinicians need to be made aware of the possibility that pathogens can be involved in more diverse diseases than previously thought. This will facilitate more incisive clinical observations and, in turn, help to build causative arguments.

- Koch’s postulates are still seen by many as a necessary tool for proving causative links between microbes and disease, but they are often inappropriate for exploring the roots of chronic illnesses. New criteria for evaluating the strength of association between microbes and chronic illnesses need to be developed.

- The limitations of the currently available methods of pathogen detection must be taken into account in drawing connections between pathogens and chronic diseases. Assays that rely on the detection of pathogen proteins or nucleic acids, must be interpreted with particular care. Failure to detect these signals is not necessarily proof that the pathogen of interest is not involved.

- Technology is continually advancing, improving the ability to effectively investigate putative links between microorganisms and chronic disease. If they remain unproven, these putative links should be revisited repeatedly to determine whether more refined tools can uncover relationships that older tools could not.

- Some attempt should be made to reconcile conflicting results from different laboratories researching microbial links to chronic illness. Potential sources of error and inconsistencies should be explored using shared reagents, blinded samples, and independent validation by third-party investigators. Alternative outcomes may reflect real differences in populations.

- Conclusive evidence of causal relationships between microbes and chronic disease can only be constructed from the results of multiple layers of techniques, including epidemiological, histopathological, clinical, and molecular tools. The application of multiple techniques is best accomplished by participation in collaborative research that brings together scientists of diverse expertise.
