# Supplementary material for: Long History of Queries about Bovine Paratuberculosis as a Risk Factor for Human Health
Source: Pathogens. 2021 Oct 28;10(11):1394. doi: 10.3390/pathogens10111394 (PMC8622788; doi:10.3390/pathogens10111394)
Supplement: Supplementary file 1 [file pathogens-10-01394-s001.zip › DALZIEL.pdf]

EIGHTY-FIRST ANNUAL MEETING

OF THE

British Medical Association.

Held in Brighton on July 23rd, 24th, and 25th.

PROCEEDINGS OF SECTIONS.

SECTION OF SURGERY.

W. THELWALL THOMAS, F.R.C.S., President.

DISCUSSION ON THE DIAGNOSIS AND TREATMENT OF PRIMARY CARCINOMA OF THE STOMACH.

OPENING PAPER.

By G. H. MAKINS, C.B., F.R.C.S.,
Vice-President, Royal College of Surgeons.

IT is a matter to me of some interest to recall the fact that some thirty odd years ago, when Billroth's first stomach operations were performed, the late Sir John Erichsen in speaking to me expressed his regret that the operations should have been performed and published, as he considered their publication would entail a great deal of useless suffering on patients who were likely to be operated upon by surgeons attracted by Billroth's proposition. At the present day a great advance has been made on that position, yet it is still an undoubted fact that far fewer subjects of gastric carcinoma come into the hands of the surgeon than should be the case; again, that they do so less frequently than do patients with carcinoma of the remaining portion of the alimentary tract, although operative treatment of the disease has been ably advocated in this country by Robson, Moynihan, Paterson, and others.

The disease occurs with great frequency, since it is computed that carcinoma of the stomach accounts for 21 per cent. of all cancers, the incidence thus following closely on carcinoma of the uterus.

The fact that surgical means are not more generally resorted to in the treatment of carcinoma of the stomach depends on two circumstances: (1) The difficulty of early diagnosis of the disease; (2) the want of general appreciation of the results which can be attained.

Incidence of Carcinoma in Various Parts of the Intestinal Tract.

Year.	Stomach.	Small Intestine.	Large Intestine.	Rectum.	Total.
1901 ...	29	4	21	22	76
1902 ...	26	1	19	27	73
1903 ...	31	0	24	32	87
1904 ...	30	1	25	32	88
1905 ...	38	0	15	35	88
1906 ...	40	0	25	32	97
1907 ...	45	0	47	17	109
1908 ...	53	1	38	29	121
1909 ...	44	1	21	30	96
1910 ...	49	1	24	43	117
1911 ...	32	1	28	43	104
1912 ...	41	0	40	35	116
	458	10	327	377	1,172
	39 %	0.8 %	27.9 %	32 %	

Total number of patients for the years 1903-12, 70,234, with 403 cases of malignant disease of the stomach. Incidence, 0.57 per cent.

The above table shows:

1. The incidence of carcinoma of the stomach in patients undergoing treatment in a general hospital. Thus, of 70,234 cases treated in the medical and surgical wards of St. Thomas's Hospital, 403 were the subjects of gastric carcinoma (0.57 per cent.).

2. The incidence of carcinoma in the various portions of the alimentary tract from the stomach downwards.

It may be noted that the table shows a slight continuous increase in the number of patients coming under treatment, which probably corresponds with the slowly growing appreciation of the fact that such patients may be successfully dealt with by surgical methods. It is of some interest to compare the numbers in this table with a similar compilation made by Dr. Mayo at St. Mary's Hospital, Rochester, in which an exact reversal of the proportionate occurrence of cases of carcinoma of the stomach and carcinoma of the large intestine respectively is seen.

	St. Thomas's (12 years).		St. Mary's, Rochester (14 years).	
	Number.	Per Cent.	Number.	Per Cent.
Stomach	458	39.0	863	68.2
Small intestine	10	0.8	14	1.1
Large intestine	327	27.9	219	17.3
Rectum	377	32.0	168	13.2
Rectum and large intestine together	704	60.0	387	30.6
Total	1,172		1,264	

Operable Cases.—St. Thomas's, 31.4 per cent.; St. Mary's, Rochester, 35.5 per cent.

I believe this to depend on the fact that where operations on carcinoma of the stomach are freely performed their success is demonstrated and the supply of cases increased. Conversely, the surgical treatment of carcinoma of the bowel has acquired an earlier recognition than that of the same disease of the stomach. This is no doubt in part due to the fact that growths of the colon and rectum are more easy of diagnosis, but chiefly because the medical attendant has always been ready to resort to surgery for the relief of intestinal obstruction, which for a long period he has known could be relieved by a simple colostomy, and for a shorter period he has become familiar with the results of partial colectomy. Thus, of the 704 cases in the table, 45 per cent. were admitted for intestinal obstruction. On the other hand, the distressing results of pyloric obstruction are not to be relieved by the simple expedient of establishing a fistula, a step which for many years has acquired a definite position in the treatment of the less hopeful conditions of carcinoma of the gullet. The introduction of gastro-enterostomy made the first step in carrying the conviction that surgery could aid, and from that the possibilities of gastrectomy are only slowly gaining the notice they deserve.

THE DIAGNOSIS OF GASTRIC CARCINOMA.

The early diagnosis of carcinomata of the intestinal tract is difficult throughout, and that of carcinoma of the stomach is perhaps the most difficult of all. Yet in early diagnosis lies the whole crux of dealing with this disease on an adequate scale.

The mere persistence of symptoms of indigestion, which differ little or not at all from those accompanying simple conditions of the stomach, except, perhaps, in their intractability may be all that exists to arouse suspicion. Such symptoms occurring in any patient of middle age, especially in a man, should lead to special investigation, particularly if they commence suddenly with a previously good digestive history, or if they supervene after a long interval of health following earlier signs of a gastric ulcer.

METHODS OF INVESTIGATION.

A determination of the functional capacity of the stomach by a test meal. From this we learn that the passage of the contents of the stomach is delayed either as a result of deficient motor power, or of mechanical obstruction, either by noting the delay in digestion of certain food elements, or by the presence of an undue amount of the ingested food after an interval which should

that the only officially recognized treatment of cancer is the knife, and the knife as early as possible.

Now it seems to me that this phrase—that a cancer is inoperable—requires consideration, and perhaps some qualification. What is an inoperable cancer? Does the surgeon mean merely that in his opinion an operation is not advisable because likely to be followed by speedy recurrence, or does he mean that it is literally impossible, without undue risk to life, to perform any operation? From the patient's point of view the term "inoperable" may have a different meaning, for he may decline to submit to the vast mutilation that some surgeons still—and with good results in many cases to support them—feel themselves justified in recommending. For example, a patient has a small buccal or lingual cancer. The surgeon consulted thinks it quite operable, and suggests excision of the upper jaw or complete removal of the tongue. The patient declines, for he prefers, if necessary, death to such deformity. In other words, his cancer, to him, is inoperable—nor, indeed, can we blame his decision.

The term, therefore, does not seem to admit of strict definition, but must vary in its sense with the judgement and experience of each surgeon. Perhaps the simplest and least objectionable meaning to be attached to it would be that a cancer must be considered inoperable or operable in unfavourable circumstances when there is extensive glandular infection. Wide extension to glands of a cancerous process implies conditions of time and distribution that make it of very grave import.

If the cancer, then, be considered inoperable, is there any resource other than surgery, or is the outlook altogether hopeless?

Five years' experience has shown me that radium is worthy of recognition by every surgeon and physician who may be called to deal with an "inoperable" cancer. I ask the consultant surgeon not to confound inoperable with hopeless conditions, but to remember that radium-therapy, although in its infancy, has already proved its value in such cases. No reasonable man who takes the trouble to follow the records that have been published in different parts of the world can doubt that radium has won—next, perhaps, to the bistoury—the highest place in our therapeutic armoury.

In Paris, my friend Dr. Chéron and Dr. Rubens-Duval published last year a case of inoperable uterine cancer cured by radium. I use the word "cured" advisedly, for fifteen months later the patient died of a disease of the central nervous system, and the autopsy verified the clinical evidence of the complete disappearance of all cancerous tissue. Some details of this case were given in the *Lancet* of November 16th, 1912.

Ten further cases of uterine or vaginal cancer have been reported by these authors in which there has been, clinically, complete disappearance of the growth. Eight of these patients are alive and well one, two, and four years after the cessation of all treatment. These results are confirmed by Léjars, the well-known surgeon, and others.

I have had under my care a number of cases of advanced cancer—all so proved by microscopic examination—that have been known to exist for periods varying from nine to eighteen months, and that, accompanied by extensive glandular involvement, have rightly been considered inoperable. In almost every case the use of radium, alone or combined with surgical intervention on the glands, has resulted in the complete disappearance of the original tumour. Sometimes there has been no recurrence; the longest case in my mind, to which I shall refer more fully in a moment, is a squamous epithelioma of the mucous membrane of the lip under the care of Professor Gaucher and Dr. Dominici, inoperable, but well under radium treatment since February, 1908—that is, over five years later. Sometimes, although the initial lesion disappears, the glands can only be incompletely dealt with, and the patient succumbs to generalization. Sometimes, when a local cure has apparently been effected a metastatic growth in the vertebrae, or in an abdominal organ, makes its appearance. Sometimes there is recurrence, so-called, some months later, in contiguous tissue.

Considering that in these cases the radium has been applied after the cancer cells have been circulating in the lymphatic system for from nine to eighteen months, such results can scarcely be laid to the discredit of the therapeutic agent. It is possible no blame can be placed anywhere; but if there is any to be apportioned it is surely to the delay in the recognition of the cancer as finally inoperable, and in reaching the decision that the case is at last suitable for the radium expert.

It is surely evident that such reasoning is neither logical nor just. The squamous epithelioma of the mucous membrane of the lip, to which I refer above, occurred in a patient shown by Dr. Dominici and myself at a demonstration given before the Radiology Section of the British Medical Association in London in July, 1910. This patient (see photograph and special plate, BRITISH MEDICAL JOURNAL, August 27th, 1910) was treated in December, 1907, and was well in February, 1908, and now, in the spring of 1913, or over five years later, remains perfectly well with the mucous membrane of the lip completely restored and normal in appearance.

As I have referred summarily to what may fairly be termed a definite cure of cancer by radium, let me also give, as briefly as possible, notes of what might be termed a failure, in that, within the year, there has been "recurrence" or rather a fresh outbreak, close to the original lesion.

In May, 1912, a patient was sent to me to Paris by a distinguished London surgeon. He first had sore-throat with a definite pharyngeal lesion, not, however, then recognized as cancer, in March, 1911.

In October, 1911, glands enlarged on the left side of the neck, and, six months later, suppurated, were opened, examined histologically and found to be squamous epithelioma. The primary lesion, an ulcer situated between the epiglottis and the side of the pharynx, was then detected and other glands noted in the neck. On his arrival in Paris, therefore, we had to deal with a carcinomatous lesion of at least fifteen months' standing, inaccessible to surgery and complicated by extensive lymphatic infection.

In consultation with Drs. de Martel and Dominici our treatment was by a combination of radium-therapy and surgery. A platinum tube containing 40 mg. of radium was introduced into the base of the tongue through a small submental incision, and, after the anterior triangle of the neck had been cleared of the glands and cancerous tissue including part of the sterno-mastoid muscle, three other tubes were left to irradiate the tissues of the neck. These four tubes were removed thirty-six hours later. The wounds healed rapidly and, a month later, the complete disappearance of the tumour was certified by three eminent throat specialists, among them the authority who had originally discovered the lesion.

Nine months later the patient's condition remained excellent, and hopes of complete recovery were held by all familiar with the details of the case.

In April, 1913, that is, ten months after the treatment, further malignant processes were found in the throat—namely, ulceration the size of a sixpence, at the base of the tongue and along the left border of the epiglottis, an inch distant from the original lesion. Under cocaine the epiglottis was removed, and the microscope showed the cancer cells extending deeply into its cartilage. The lingual ulcer was dealt with by the introduction into the base of the tongue, through small lateral incisions in the neck, of two tubes of radium of 45 and 25 mg. A month later the most careful search again failed to detect any sign of malignant disease, and the patient is to-day in full enjoyment of his usual strength.

Even admitting such a case to be, however, a failure in that recurrence took place, it may justly be claimed for radium:

1. That the original tumour disappeared.
2. That the neck remains free from evidence of lymphatic infection; and
3. That the patient is still enjoying healthy activity a year after a hopeless verdict had been pronounced, and more than two years after the first evidence of cancer.

CHRONIC INTERSTITIAL ENTERITIS.

By T. K. DALZIEL, M.B., C.M., F.R.F.P.S.G.,
Surgeon, Western Infirmary, Glasgow.

I HAVE pleasure in drawing your attention to this condition, which, I think, has not yet been fully described.

Twelve years ago I saw a professional colleague, suffering from obstruction of the bowels of a fortnight's duration.

previous to which he had had for several weeks numerous attacks of colic, slight attacks of diarrhoea with no tenderness over the abdomen, and very slight rise in temperature, with no appreciable alteration in the pulse-rate. When seen by me the abdomen was not distended nor were the muscles rigid, but to the hand gave a sense of putty-like resistance. As vomiting was persistent, I concluded that there might be an obstruction high up, and so opened the abdomen, to find the whole of the intestines, large and small alike, contracted, rigidly fixed, so that when a loop was lifted from the abdomen it sprang back into its sulcus. That the wall of the whole intestine was chronically inflamed there was no doubt. In parts the peritoneum seemed oedematous, as was also the omentum and mesentery, in which the glands could be felt enlarged. Nothing could be done to restore the function of the canal, and the patient died a few days afterwards.

We were not then familiar with the condition, and it was supposed to be tuberculous, though this was negatived by microscopic examination, the only information we obtained from the pathologist being that the condition was a chronic and inflammatory one. A few years later, with Dr. Gibb of Paisley, I saw an exactly similar condition in a young man of 32. His symptoms were somewhat more acute than the previous case, but practically the same. He also died. No examination was allowed.

In these two fatal cases the disease involved the whole of the small and large intestines. The following cases being localized, and therefore excisable, permitted operation, and excision of the affected portion was in all cases followed by complete restoration to health.

The first of these cases I saw with the late Professor Gemmell in 1905.

Mrs. T. was admitted to the Western Infirmary with symptoms of partial obstruction, and one could palpate a coil of intestine, rigid and thickened. Treatment was of no avail, attacks of pain becoming more frequent; progressive emaciation and general malaise led to operative interference, when a portion of jejunum over 2 ft. in length was found to be affected and was excised, with perfect recovery. I removed in two cases the caput coli and adjoining portion of the ileum.

In another case the sigmoid, and in another the transverse colon, and, lastly, from a child of 10 a specimen which well now indicates the great thickening of the bowel wall. This specimen was from the middle of the ileum.

The following is the pathological report of the condition from the laboratory in the Western Infirmary, on the specimens obtained from the ileum, jejunum, and colon.

Pathology.

Histologically there is much in common in the three cases, indeed they form a graded series in which all the stages from acute to chronic may be traced. The most acute lesions are found in Master W. G., and the most chronic in Mrs. N. The following description is based upon a study of numerous sections from each case.

The earliest change in the bowel appears to be that of acute congestion. The vessels throughout are dilated, and there is much oedema of the submucosa. As evidences of the acute inflammation, the vessels are seen to be rich in polymorphs, and there is considerable infiltration of all the coats with similar cells. Here and there, too, in mucosa and submucosa irregular haemorrhages have occurred. These changes also implicate the mesentery in a lesser degree. It is noteworthy that the lymphoid aggregations are singularly free from pathological change.

With increasing infiltration the next phase arises, namely, cellular and fibrinous exudation within the gut lumen (bile-stained naked eye). Still later the mucous membrane is denuded of epithelium, and, the muscularis mucosae being obscured by infiltration and necrosis, the appearance is that of a few islets of glandular tissue lying in a semipurulent collection which abuts upon the much altered submucosa. There is, however, no great sloughing of the bowel wall, and the muscle is not laid bare, indeed it is in a way protected, as shown by a new formation of capillaries in the more superficial layers of the submucosa.

In the specimen from Mrs. T. the regenerative process is in the ascendancy, although the condition is still fairly acute. The serous and muscular coats are slightly oedematous, markedly congested, even slightly haemorrhagic, and considerably infiltrated with both polymorphs and mononuclear cells. The submucosa is also oedematous and infiltrated, mononuclear cells, however, predominating. The muscularis mucosae is definable as the outer limit of a broad zone of young granulation tissue which is evidently replacing the now thin layer of purulent exudate within the gut lumen.

A still further advance in the healing process is seen in the sections from Mrs. N. There is scarcely any purulent exudate within the lumen, it and the mucosa having been replaced by granulation tissue in which the vessels are numerous and well formed, and fibroblastic transformation is well marked. There is less oedema of the tissues than in the two previous cases, and though leucocytic infiltration of all the coats is still great, it is definitely a mononuclear one. Further, there is a notable number of eosinophiles throughout, and a few giant cells are also present in the granulation tissue.

From the acute case, Master W. G., coliform bacilli were isolated in pure culture from the depths of the affected bowel wall under circumstances which suggest an etiological relationship. They are also demonstrable in suitably stained sections.

A careful search has failed to reveal micro-organisms of any kind in the depths of the other two cases—the ordinary bacterial flora of the gut alone visible in the most superficial part of the exudates. The symptoms in all the cases were similar; the characteristic and most striking feature being most violent colic, causing vomiting and occasionally an escape of some blood, also constant mucus from the bowel. The bowel becoming exhausted, or the contents being forced through the rigid portion, the patient then would be at rest, quite comfortable and cheerful for a time. In the case of the child even ten or twelve hours might elapse between the attacks of pain, which were truly distressing in their intensity. In the young one would naturally suspect intussusception, except that the obstruction was not complete, while the intensity of the pain put a chronic intussusception out of the question. Above the affected portion of the bowel peristalsis could be observed. During a painful attack the inability to retain food and the constant suffering leads to steady emaciation, the temperature only occasionally rises and during the intervals of pain, and the pulse is quiet. In all the cases one could determine an area of resistance in the colon and sigmoid, naturally giving rise to the supposition that we might have to deal with a diffused and malignant growth. As far as I am aware, the prognosis is bad except in cases where the disease is localized, and even there seems rather hopeless unless operation be had recourse to.

Etiology.

In regard to etiology, we have no direct clue by histological or pathological examination. The cases gave the impression that they were probably tuberculous, and yet from the uniform character of the affection it evidently is not so. The affected bowel gives the consistence and smoothness of an eel in a state of rigor mortis, and the glands, though enlarged, are evidently not caseous.

In vol. xx of the *Journal of Comparative Pathology and Therapeutics*, McFadyen draws attention to Johne's disease, a chronic bacterial enteritis of cattle which was called pseudo-tuberculous, in which the histological characters and naked-eye appearances are as similar as may be to those we have found in man. The condition was first described by Henny and Frothingham in 1895, since which time numerous observers have noted its course in various parts of the Continent, and McFadyen examined 6 cases found in England in 1911. McFadyen, however, describes an acid-fast bacillus similar to but demonstrably not the tubercle bacillus, differing in size, and also as not giving rise to tuberculosis in guinea-pigs. This bacillus is found not only in the tissues but also on the surface of the mucous membrane, which in animals seems to be more affected, presumably because they die earlier, than in man, so that the disease is not so advanced. In my cases the absence of the acid-fast bacillus would suggest a clear distinction, but the histological characters are so similar as to justify a proposition that the diseases may be the same. As far as I know the disease has not been previously described, but it seems probable that many cases must have been seen and have been diagnosed as tuberculous, and possibly nothing done for their relief.

Treatment.

In regard to treatment, these cases which have come under observation have pursued their course uninfluenced by dietetic or medicinal treatment, and apparently only operation can afford relief, and then only if the disease be limited. Seven out of the nine made a perfect recovery after the operation, and one does not hesitate in resecting large portions of the intestine. The subject has been one of great interest to me for some years. My friends the pathologists prefer to call it hyperplastic enteritis, and I can only regret that the etiology of the condition remains in obscurity, but I trust that ere long further consideration will clear up the difficulty.

Another specimen I obtained recently from a patient of Dr. Revie of Kilmarnock.

A lady on whom I had performed colostomy on the right side a year previously, with the object of arresting the intestinal current to enable us to freely flush the diseased colon. The symptoms were those already described with an exaggerated degree of pain, and persistent, most painful diarrhoea with blood and mucus. Distinct improvement ensued from the colostomy and lavage, though during the year she had on two occasions exacerbations. When seen at the end of the year she had been extremely ill again for one month, and was so evidently losing ground that I advised complete removal of the colon, which colon shrunk to its present dimensions as I now show you. The histological characters are similar to those found in the previous specimens. The patient has made an uninterrupted recovery so far, and I hope at no distant date to transplant her caput coli (which alone was unaffected and was left) to her rectum.

An interesting fact in this case is that on the removal of the colon it was immediately sent in a sealed vessel to the pathologist, and he failed to discover micro-organisms either on the surface of the mucous membrane or in the tissues, indicating that lavage had been thoroughly effective in purifying the canal, in spite of which lavage, however, the disease had steadily progressed. Indeed, from the first operation the disease had extended upwards from below the hepatic flexure to near the caput coli.

DISCUSSION ON THE DIAGNOSIS AND TREATMENT OF INJURIES OF THE KNEE-JOINT OTHER THAN FRACTURES AND DISLOCATIONS.

OPENING PAPERS.

I.—A. M. MARTIN, M.B., B.S.Durh.,
Surgeon, Royal Victoria Infirmary, Newcastle-on-Tyne.

IN the first place I must thank the President and Council of the Surgical Section for the honour they have done me in asking me to introduce the discussion on the diagnosis and treatment of injuries to the knee-joint other than fractures and dislocations. I can assure you it is an honour I greatly appreciate, and I sincerely trust that the subsequent discussion will prove both interesting and profitable.

It has been somewhat difficult for me to decide upon the form in which I should treat the subject, but, after considerable thought, I have come to the conclusion that the clearest and best method will be to exclude such conditions as perforating wounds and their effects, and confine myself to what one might term subcutaneous injuries. Among these are simple traumatic synovitis and arthritis, more or less severe lacerations of important ligaments, haemophilia, some forms of loose bodies, injuries, sometimes trivial, to joints already affected with disease, such as osteo-arthritis and torn semilunar cartilage.

Anatomy.

Before considering these injuries seriatim, I think it would make the subject clearer if I briefly mentioned some of the more important features in the anatomy of the knee-joint, particularly those which have a bearing on my subsequent remarks. This joint, besides being the largest, is the most complicated articulation in the body, and possesses important extrinsic and intrinsic ligaments. Included in the former are the anterior or ligamentum patellae; the internal lateral, which is a broad, flat structure; the cord-like external, and lastly, the posterior. The chief intrinsic ligaments are the anterior and posterior crucial. Also contained in the joint, and in close relationship to the articular surface of the upper end of the tibia, are the two important semilunar cartilages. These are two crescent-shaped pieces of fibro-cartilage, the internal being applied to the upper surface of the internal and the external to the upper surface of the external tuberosity of the tibia. The internal forms a larger segment of a circle, and is less movable than the external. Its posterior end is attached to the back part of the intermediate rough area on the upper end of the tibia in front of the attachment of the posterior crucial ligament, and the anterior end is attached to the front of the intermediate rough area just in front of the anterior crucial ligament. Its circumference has a firm attachment to the deep surface of the internal lateral ligament, and a somewhat weak connexion, by means of the coronary ligament, to the upper end of the tibia. The external cartilage forms a smaller segment of a circle than the internal, and takes up a smaller space. Its anterior extremity is fixed to the intermediate rough area in front of the tibial spine, while its posterior end is connected to the tibial spine between the two tubercles, giving a strong slip to the posterior crucial ligament. It has no attachment to the external lateral ligament, being separated from this by the tendon of the popliteus muscle, but it has a weak connexion with the upper end of the tibia by means of the coronary ligament. The external semilunar cartilage therefore has a decidedly looser attachment than the internal, and this circumstance, as I will show later, is the cause of the greater frequency of tears or splits of the internal semilunar cartilage.

The knee is not a simple hinge-joint, as at the end of extension and the beginning of flexion there is a distinct twisting movement of the femur on the tibia. This is brought about by the fact that the articular surface of the internal condyle is longer by a third than the corresponding surface of the external. Thus, in the movement of extension, following upon acute flexion, after the articular surfaces on the external and posterior two-thirds of the internal condyles have glided over the corresponding parts of the articular surfaces of the tibia, any further movement must occur in the anterior oblique third of the internal condyle. In consequence, this condyle twists or screws inwards, and at the end of extension the joint is securely locked. Thus, the femur and tibia become practically one continuous rigid support; no lateral or rotatory movements being possible, and the following ligaments—namely, the internal lateral, anterior crucial, and posterior—become taut and tense. When, however, flexion takes place, a certain amount of rotation and lateral mobility is possible, varying in degree with the amount of flexion. Whereas, therefore, in the case of a fully extended knee-joint, no rotation is possible, except there be tearing or stretching of important ligaments, in the case of the flexed position a certain amount of rotation and lateral movement is possible, and hence there is greater insecurity. It is in this flexed position that tearing or splitting of the semilunar cartilage takes place.

In a paper which I read on October 8th, 1912, before the Royal Society of Medicine, after stating that the occupation of coal-mining far outnumbered all others, in the district of Northumberland and Durham, in supplying the vast majority of sufferers from this accident, I mentioned that the coal-miner, owing to the lowness of the seam at which he is hewing, performs his work with his knees more or less flexed—in a position, therefore, in which a certain amount of rotation and lateral mobility, between the upper end of the tibia and the lower end of the femur, is permitted. If, while the knee is in this position, a forcible twist or wrench takes place, the semilunar cartilage, nipped between the condyle of the femur and the upper end of the tibia, may be forcibly dragged towards the centre of the joint, and either split more or less in a longitudinal manner, or have its free edge torn or frayed.

This conclusion, which was based upon a personal experience of 449 cases in which operation had been performed, and in which great care had been exercised in taking the history of the patients, is, I am aware, directly contrary to that of Mr. Albert Walton, who is of opinion that forcible extension is the cause. I may mention, however, that his opinion is based mainly upon anatomical grounds and experiments on the cadaver.

I would here urge that in all cases of injury to the knee joint accurate inquiry should be made into the story of the accident and the subsequent history, as, when the
