# Supplementary material for: Long History of Queries about Bovine Paratuberculosis as a Risk Factor for Human Health
Source: Pathogens. 2021 Oct 28;10(11):1394. doi: 10.3390/pathogens10111394 (PMC8622788; doi:10.3390/pathogens10111394)
Supplement: Supplementary file 1 [file pathogens-10-01394-s001.zip › EU_COMMSION.pdf]

SANCO/B3/R16/2000

EUROPEAN COMMISSION
DIRECTORATE-GENERAL HEALTH & CONSUMER PROTECTION

Directorate B - Scientific Health Opinions
Unit B3 – Management of scientific committees II

Possible links between Crohn's disease and Paratuberculosis

Report of the Scientific Committee on Animal Health and Animal Welfare

Adopted 21 March 2000

Possible links between Crohn's disease and Paratuberculosis.

Contents

1. REQUEST FOR AN OPINION 4
1.1. BACKGROUND 4
2. *M. AVIUM.* SUBSP. *PARATUBERCULOSIS* AND RELATED STRAINS 6
2.1 DESCRIPTION OF M. AVIUM 6
2.2 DESCRIPTION OF M. AVIUM SUBSP. PARATUBERCULOSIS 6
2.3 DIVERSITY OF M. AVIUM SUBSP PARATUBERCULOSIS AND STRAIN DIFFERENTIATION 7
3. DESCRIPTION OF PARATUBERCULOSIS 9
3.1 HISTORY 9
3.2 AETIOLOGY 9
3.3 PATHOGENESIS 10
3.3.1 Genetic Susceptibility 10
3.3.2 Immunopathology 10
3.4 PATHOLOGY 12
3.5 CLINICAL FEATURES 13
4. DESCRIPTION OF CROHN'S DISEASE 14
4.1 HISTORY 14
4.2 AETIOLOGY 14
4.3 PATHOGENESIS 15
4.3.1 Disease mechanisms 15
4.3.2 Genetic susceptibility 15
4.3.3. Immunopathology 16
4.4 PATHOLOGY 17
4.5 CLINICAL FEATURES 18
5. EPIDEMIOLOGY AND GEOGRAPHICAL DISTRIBUTION OF BOTH DISEASES ... 22
5.1 EPIDEMIOLOGY AND GEOGRAPHICAL DISTRIBUTION OF PARATUBERCULOSIS IN ANIMALS 22
5.2. EPIDEMIOLOGY AND GEOGRAPHICAL DISTRIBUTION OF CROHN'S DISEASE 23
5.3 IS THE INCIDENCE OF BOTH DISEASES CHANGING? 26
5.4 IS THERE A CAUSAL ASSOCIATION BETWEEN THE GEOGRAPHICAL INCIDENCE/PREVALENCE OF CROHN'S DISEASE AND THAT OF PARATUBERCULOSIS? 26
6. DIAGNOSIS AND CONTROL OF PARATUBERCULOSIS 28
6.1 INTRODUCTION 28
6.2 DETECTION OF THE CAUSATIVE AGENT 29
6.2.1 Microscopical examination 29
6.2.2. Cultural methods 29
6.2.3. Polymerase Chain Reaction (P.C.R.) 30
6.3 DETECTION OF AN IMMUNE RESPONSE TO M. AVIUM SUBSP. PARATUBERCULOSIS 31
6.3.1 The complement fixation test 31
6.3.2 The absorbed ELISA 32
6.3.3. Agar-gel-immuno-diffusion test (AGID) 32
6.3.4 Cellular Immunity Tests 33
6.4 TREATMENT OF PARATUBERCULOSIS IN ANIMALS 33
6.5 VACCINATION AGAINST PARATUBERCULOSIS 34
6.6 CONTROL AND ERADICATION PROGRAMMES 36

7. *M. AVIUM* SSP *PARATUBERCULOSIS* INFECTION IN HUMANS. 39

7.1 DETECTION OF MAP IN CROHN'S DISEASE BY CULTURE AND PCR. 39
7.2 IMMUNOLOGICAL RESPONSES TO MAP IN HUMANS WITH CROHN'S DISEASE. 43
7.3 TREATMENT OF CROHN'S DISEASE WITH ANTIMYCOBACTERIAL DRUGS. 46

8. EXPOSURE OF HUMAN POPULATIONS TO *M.AVIUM* SSP.*PARATUBERCULOSIS* 49

8.1 NON-DIETARY EXPOSURE 49
8.2 DIETARY EXPOSURE. 49
8.2.1 Raw milk 49
8.2.2 Heat treated milk 50
8.2.3 Other foods 51
8.2.4 Water supplies... 51

9. SUMMARY 52

10. RESEARCH 54

10.1 CROHN'S DISEASE 54
10.2 PARATUBERCULOSIS 54

11. CONCLUSIONS 56

12. REFERENCES.. 57

13. ACKNOWLEDGEMENTS 74

1. Request for an opinion

In the light of speculation that Crohn's disease in man and paratuberculosis in animals (also known as Johnes disease) is caused by the same organism *Mycobacterium avium* subsp. *paratuberculosis*, the Scientific Committee on Animal Health and Animal Welfare is asked to examine and comment on the evidence for and against a causative link between the two diseases.

1.1. Background

Paratuberculosis is a chronic inflammation of the intestine in animals caused by a slow growing and difficult to culture bacterium called *Mycobacterium avium subsp. paratuberculosis* (*Map*). Paratuberculosis can affect many different animals including primates. It is particularly prevalent and apparently increasing in European dairy herds and other domestic livestock such as sheep, goats and farmed deer[1],[2].

Paratuberculosis is a serious disease with considerable economic consequences. The net cost to farmers of subclinical infection with *Map* in their animals, was estimated in Europe in 1987 to be £209 per infected dairy cow mostly due to reduced milk production.[3] Recent work in the US[4] has estimated that paratuberculosis costs dairy farmers there around $100 per cow in moderately infected herds rising to over $200 per cow in heavily infected herds. These losses were mainly due to decreased milk production and increased cow replacement costs. The study estimated the costs in terms of reduced productivity alone to be $200 to $250 million per annum which would be a considerable underestimate of the total costs due to this disease. In Spain, losses in sheep have been estimated to be €120 and €60 for clinical cases in dairy and meat sheep respectively[5].

Besides losses due to emaciation and death, infected animals show decreased productivity, increased infertility and susceptibility to other infections[6]. Other causes of culling like mastitis or infertility were significantly higher in cows infected with *Map* than in non-infected animals. In milk production, a decrease of 16% was observed between the current lactation and the lactation two seasons previously in clinically affected dairy cows[3]. As in other diseases, poor health leads to poor welfare.

Crohn's disease is a chronic inflammation of the intestine in humans. A major multi-centre European study[7] has calculated an incidence rate for Crohn's disease of 5.6 per 100,000 per year, though there were considerable local differences. The disease is lifelong, though considerable periods of remission may occur. It is estimated that over 200,000 people are affected by this disease in the European Union.

Data of the cost of Crohn's disease to society in Europe are not available. However, a Swedish study[8] has shown that in 1994 Sweden spent over €40 million on patients with this condition, 29% of which were direct costs i.e. costs for in-patient care, ambulatory care and drugs. Indirect costs i.e. sickness leave and early retirement constituted 71% .

Crohn's disease results in substantial morbidity and high use of health services. Mortality is not usually a feature of the disease though patients, particularly the young, have double the risk of dying compared with a matched group drawn from the general population, mainly because of complications associated with the disease[9]. A US[10] study found that for a representative patient, projected lifetime costs were $125,404 using mean charges. Overall, the total annual medical costs in the US were estimated in 1992 at $1 billion-$1.2 billion[11]. These costs were unevenly distributed with the top 2% of patients accounting for around 29% of the costs. If disability payments and the cost of early retirement are taken into account the total cost of this disease is much higher.

This report has confined itself to an examination of the possibility that Crohn’s disease is caused by *Map*. It does not examine other possible causes of Crohn’s disease in detail. The examination has focussed on a number of aspects, a description of the organism, a comparison of both diseases, a discussion of the geographical prevalences of both diseases, diagnosis and control of paratuberculosis in animals, detection of the organism in Crohn’s patients, immunological responses to the organism in Crohn’s patients, a discussion on the effects of various treatments and finally a consideration of the possibility of humans being exposed to the organism from animal sources.

2. *M. avium.* subsp. *paratuberculosis* and related strains

2.1 Description of M. avium

Mycobacterium avium subsp. *paratuberculosis* belongs to the Family *Mycobacteriacea,* Chester, 1897. The main attributes of these bacteria is their slow growth rate and resistance to treatments with acid and alcoholic compounds. This is due to their strong cellular wall, with a high lipid composition.
Mycobacterium avium form a large group of closely related mycobacteria which can be subclassified into *Mycobacterium avium* subspecies *avium* (*Maa*), *Mycobacterium avium* subspecies *silvaticum* (*Mas*), and *Mycobacterium avium* subspecies *paratuberculosis* (*Map*)[12] . *Mas* (originally called the woodpigeon strain) contains the specific insertion sequences IS*901* and IS*902*[13,14] and has been also reported to produce a disease resembling paratuberculosis in calves and deer[15,16,17,18]. *Maa* are becoming better defined by molecular methods including rDNA sequencing and restriction fragment length polymorphism (RFLP) genotyping exploiting the increasing number of insertion elements (IS) identified in this group of organisms. *Maa* are very widely distributed in the natural environment including soil and apparently healthy animals, birds and humans. The insertion elements they contain include IS*1245*[19,] IS*110*[20], IS*1311*[21] and IS*1626*[22]. These have been used to type *Maa* causing infections in animals and humans[23,24]. *Maa* are generally opportunist pathogens and only cause infection and disease in debilitated hosts.

In culture, colonies of *Maa* may be smooth or rough and the switch in colony type may occur in repeated sub-cultures following the first isolation from the sample. Smooth to rough transition in *Maa* serovar 2 is associated with specific genetic deletions and irreversible loss of pathogenicity[25] . Growth in culture is usually visible after more than ten days at 37°C, which is the optimal temperature, and may require mycobactin and/or special media other than egg based media. Growth may be stimulated by pH 5.5 or pyruvate. Strains do not produce niacin, peroxidase, nitrate reductase, urease, arylsulfatase, penicillinase, or beta-glucosidase and do not hydrolise Tween 80 in 10 days. Strains produce small amounts of thermoresistant catalase. All strains have the same mycolic acid pattern and are resistant to thiophene-2-carboxylic acid hydrazide and isoniazid. Strains may be susceptible to p-nitrobenzoate, ethambutol, pyrazinamide, rifampin, and streptomycin. Most strains are susceptible to ansamycin. The reference strain is strain ATCC 25291. The species includes both potential and obligate pathogens and is divided into subspecies on the basis of pathogenicity and host range[12].

2.2 Description of M. avium subsp. *paratuberculosis*

M. avium subsp. *paratuberculosis* possesses the properties of *M. avium* along with some additional features. The organism causes paratuberculosis, a chronic enteric disease in animals. The reference strain is strain ATCC 19698[12].

The main distinguishing feature of *Map* is its slow growth and the dependency on exogenous mycobactin for in vitro growth. Mycobactin is an iron-chelating agent produced by all other mycobacteria. However, *Map* does not produce, or produces an insufficient amount of mycobactin. It is closely related to other *M. avium* bacteria, sharing some antigenic determinants. Different strains of this bacterium have been reported: a bovine strain, a pigmented strain and strains isolated from small ruminants that seem to grow slower than bovine ones[26]. Strains obtained from sheep and goats seem to be more difficult to culture[27]. A specific insertion sequence of *Map,* IS900, has also been reported[28].

2.3 *Diversity of M. avium* subsp paratuberculosis *and strain differentiation*

The development of reliable techniques that can be used to examine diversity in the *M.avium* complex has evolved over a number of years and encompasses a range of techniques directed at phenotypic and genetic characteristics[29]. For example, numerical taxonomy that analyses a panel of phenotypic properties can distinguish three subspecies of *M. avium*, viz *M. a. avium*, *M. a.* subsp. *silvaticum* and *M. a.* subsp. *paratuberculosis* [12]. However, this is a complex approach and a more common way of differentiating closely related species is to exploit differences in the composition of their genomes. Molecular techniques targeting rRNA genes have been used extensively for establishing taxonomic and evolutionary relationships between closely related organisms. Unfortunately, each of the rRNA genes (16S, 23S and 5S) as well as their intergenic spacer regions are highly conserved within the *M. avium* complex [30],[31],[32]. Analysis of these regions has demonstrated the high genetic homology of these organisms and failed to discriminate *M. avium* and *Map*. Fortunately, identification of a specific insertion element, IS900, in *Map* has greatly simplified the detection of this organism and its differentiation from *M. avium* [28].
Most research on diversity and strain differentiation within the *M. avium* complex has focused on molecular techniques to detect RFLPs, which have been exploited successfully to reveal genetic diversity in many organisms. Conventional restriction endonuclease analysis and field inversion gel electrophoresis can discriminate *Map* from other *M. avium* species, but are unable to differentiate different strains of *Map* [33],[34],[35]. RFLP analysis can be improved by hybridising the DNA fragments with specific probes. Such analysis of *M. avium* isolates, using IS1245 and IS1311, has revealed a high degree of diversity among human and porcine strains[36],[37],[23]. Similar analyses of *Map* have employed both IS900 and IS1311. Using the IS900 insertion sequence as a hybridisation probe, around 20 different RFLP types have been identified, reflecting some differences between strains [38],[39],[40],[41],[42],[43],[44]. In general, the observed polymorphisms have not been associated with the animal of origin [25],[29]. However, this insertion element has specific integration targets, which place constraints on the possible number of polymorphisms. RFLP analysis using IS1311 as a probe detects fewer polymorphisms than IS900, which probably reflects the lower copy number of IS1311 elements[21],[45]. Whether the IS1311 polymorphisms overlap the IS900 polymorphisms and provide additional epidemiological information has not been

assessed. Interestingly, point mutations in IS1311 have allowed differentiation of isolates originating from sheep and cattle in Australia[45],[46] . Unless new repeated DNA sequences that are specific for *Map* can be identified, the use of RFLP analysis in epidemiological studies of *Map* will be limited.

Pulsed field gel electrophoresis (PFGE) is useful for strain differentiation of human isolates of *M. avium*, particularly those from AIDS patients[47],[48],[49],[50]. This technique has not been applied extensively to strains of *Map* and more work is needed to determine its potential [51],[52],[53].

Although IS900 has 14-16 specific integration sites, six appear to be variably inserted or rearranged, allowing the discrimination of eight genotypes. This feature has permitted the development of a multiplex PCR to identify which integration sites are present in any genome and has been able to discriminate isolates from different host species e.g., cattle, sheep and man[54]. This technique, therefore, warrants fuller assessment and may be a useful tool in epidemiological investigations.

There is a great need to develop non-targeted techniques, which may reveal greater diversity. Randomly amplified polymorphic DNA (RAPD) patterns produced by PCR have been shown to be highly discriminatory for many bacteria and have been used successfully to type clinical isolates of *M. tuberculosis* and *M. avium* [55],[56],[57]. Analyses of *Map* by this technique have produced conflicting results, although some diversity between the isolates was reported [58],[59].

In summary, the currently available evidence points to a high degree of genetic homogeneity within *Map*, and current characterisation techniques are not yet sufficiently developed to support detailed epidemiological studies by differentiating between mycobacterial strains.

3. Description of Paratuberculosis

3.1 History

Paratuberculosis was first described in Germany in 1895 by Johne and Frothingham[60]. They demonstrated the presence of acid-fast bacilli in affected animals and thought that the disease was an atypical form of tuberculosis. Twort isolated the causative organism in 1910 and named it *Mycobacterium enteriditis chronicae pseudotuberculosae bovis johne*. The disease later became known as paratuberculosis or Johne's disease and the causative agent *Mycobacterium avium* subsp. *paratuberculosis* (*Map*). The name *Mycobacterium johnei* has also been used in the past.

3.2 Aetiology

Paratuberculosis is a chronic digestive disorder of both wild and domestic ruminants. The disease is characterised by a granulomatous enteritis that provokes a progressive weight loss and ends with the death of affected animals. Paratuberculosis is a contagious and enzootic disease of ruminants caused by the multiplication of a specific bacterium, *Map* in the mucous membrane of the intestine. The organism, which is described in detail in Chapter 2, can also infect a wide range of other domestic and wild (free-living or captive) species including primates. This mycobacterium has recently been isolated in wild rabbits suffering a chronic granulomatous enteropathy[61] and also from their predators, i.e. foxes, weasels and stoats[62].

The infection can be transmitted by either direct or indirect contact of infected animals with susceptible animals. Transmission occurs mainly by the faecal-oral route. Bacilli are ingested most commonly in large numbers when young animals nurse on teats which have been contaminated by faeces of shedding animals[63] . These faeces may contaminate pastures, feed and water. The ability of this mycobacterium to resist destruction in the natural environment (it can survive nine months or longer outside an animal) makes prevention and control of paratuberculosis more difficult[64].

Map has also been recovered from the uterus and placenta of infected cows and intrauterine infection of the foetus sometimes occurs. However, classical lesions of paratuberculosis have not been recognised in infected foetuses. Although *Map* has been isolated from the semen of infected bulls, venereal transmission by artificial insemination or natural service is unlikely[1,65]. There appears to be an age-related resistance to the development of new paratuberculosis infections. Animals infected in the first few months of life are the most susceptible[66,67]. Adult animals are quite resistant. Although infections are usually initiated during calfhood, clinical disease does not appear until adulthood. Whether or not the exposed calf becomes infected depends on the number of bacilli ingested and the defence mechanisms of the host[68]. The infection persists in breeding stocks after the introduction of infected animals.

The long incubation period of the disease allows mycobacterial shedding in faeces by animals for up to 18 months before clinical signs become apparent, but shedding is particularly high (up to 5 x 10^{12} mycobacteria per day) during clinical expression of the infection[1].

Risk factors include intensive farming systems, acid soils, low dietary intake, stress related to transport, lactation and parturition and immunosuppression by agents such as bovine virus diarrhoea virus (BVDV). Close contact of susceptible animals with contaminated faeces is an important influence and may, because of the different husbandry methods, account for the reports of a higher prevalence in dairy cattle than in beef cattle[1].

3.3 Pathogenesis

3.3.1 Genetic Susceptibility

Some breeds of cattle are commonly regarded as being particularly susceptible to paratuberculosis (Jersey, Limousin) but this aspect has not been studied in detail[1].

3.3.2 Immunopathology

Once ingested, the bacilli immediately penetrate the mucosal surfaces of the gastrointestinal tract and are phagocytised by macrophages[69] . The portal of entry is the gut associated lymphoid tissue (Peyers patches), both in the ileum and in the jejunum. These patches reach their maximum development about the time of birth and progressively disappear afterwards, though patches in the jejunum and ileocaecal valve region can persist in adult animals[70]. This could be one of the explanations for the highest susceptibility to the infection occurring in young animals. Once within the macrophages, the bacilli remain viable and protected from humoral factors. Generally, a granulomatous lesion first starts to develop in the jejunal and ileocaecal Peyers patches area[71]. At this stage lesions can persist as latent for long periods of time[67]. Later, as the infection progresses, lesions will affect different parts of the Peyers patches related mucosa and finally will spread to wide areas of the intestine, causing the typical granulomatous enteritis[72]. In the normal course of the infection, a cell-mediated response is seen initially[63,73]. Later, as the disease progresses, there is a humoral response initiated by the release of bacilli from dying macrophages.

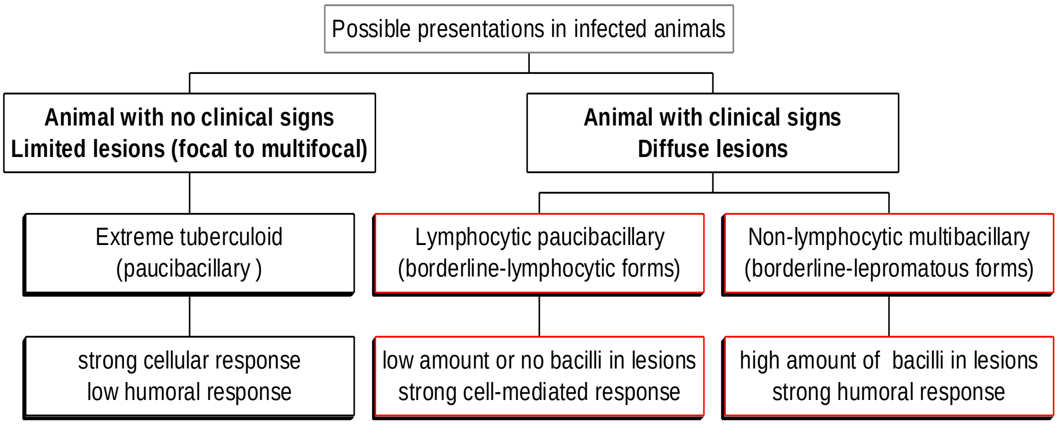

Figure 1: Immunological and histopathological spectrum of paratuberculosis

The existence of a wide immunological and histopathological spectrum, dependant on the host response, which was proposed initially in the case of leprosy[74] has subsequently been applied to different mycobacterioses such as paratuberculosis. At one end of this spectrum, the tuberculoid extreme is located where the host offers a strong cellular immune response with a very low humoral response. At the opposite extreme of the spectrum one finds the lepromatous forms associated with a weak cellular immune response but with a strong humoral response. Between both extremes one finds the so-called borderline forms (borderline-lymphocytic and borderline-lepromatous forms). Animals showing these borderline forms exhibit the most severe clinical signs.

When culled animals from an infected sheep flock are studied, a range of lesions may be found[5],[75],[76], which may be delimited or diffuse. The former are not usually considered in pathological studies since they are only observed in apparently healthy sheep. However they represent about two thirds of the total number of infected animals and can be further divided into focal-tuberculoid and multifocal tuberculoid types.

Among clinically affected animals, different pathological pictures can be found with varying amounts of bacilli in the lesions, depending on the immune status of the host Some can show the diffuse non-lymphocytic multibacillary forms (borderline-lepromatous), with high amounts of bacilli and a strong humoral response whereas others could present diffuse lymphocytic paucibacillary forms (borderline-tuberculoid), with few or no bacilli and a strong cell-mediated immune responses. In summary, animals from a paratuberculosis infected flock can present varying immunological and pathological pictures and be located in different positions in the spectrum. All of these different immunological responses may appear at any time and the disease presentation can change from the limited to diffuse forms during the course of the infection. An understanding of this feature is especially important because the response of the animals to diagnostic tests is associated with their location in the spectrum[77],[75].

3.4 Pathology

The principal pathological changes centre on the intestine and the related lymphatic and lymphoid tissues although, in advanced cases, pathology associated with cachexia may be present. It must be emphasised that gross changes in sheep and goats with paratuberculosis are often lacking or difficult to detect, and may not resemble those of the disease in cattle[1].

In advanced cases there is wasting with gelatinous atrophy of fat depots and serous effusion into body cavities. In the intestine, macroscopic changes principally affect the terminal ileum, but may extend to involve the jejunum and colon. The ileum may be thickened and may feel doughy when handled, but more usually the only visible change in the lining is a slight fleshy or velvety thickening, or a faint granularity of the surface, perhaps with slight congestion. These subtle changes may be overlooked in a cursory examination. Occasionally, there may be a tendency for the mucosa to form fissures when bent over the fingers. Where infection with pigmented strains occurs, the mucosal lining takes on a pathognomonic bright yellow colour, due to the presence of pigmented *Maps* in the lamina propria.

The afferent lymphatic vessels in the intestinal peritoneum and mesentery may be thickened and convoluted, and contain numerous small (1-4mm) whitish nodules, which may be caseous or even calcified. Similar nodules or white flecks may be seen on the peritoneal surface of the ileum, or the cut surface of the intestinal wall or the mesenteric lymph nodes. The latter are almost invariably enlarged and prominent at necropsy.

Although all clinical cases present as an afebrile, chronic wasting, the severity of the signs is unrelated to the extent of the pathology. In cattle, sheep and goats, two distinct types of pathology are apparent, based on the abundance of mycobacteria and cellular infiltrate[76],[78]. The more common form, known as borderline-lepromatous or multibacillary, is characterised by numerous acid-fast *Map* packing the cytoplasm of the many large macrophages that infiltrate the mucosa in all cases, forming extensive, diffuse sheets. Lymphocytes and granulocytes are present in much lower numbers. Occasional multinucleate, Langhans-type giant cells may be seen. These changes cause marked thickening of the intestine.

The less common form, known as lymphocytic or paucibacillary, comprises approximately 30% of cases in sheep and goats[79]. It is characterised by a more marked lymphocytic infiltrate with scattered, small focal granulomata and giant cells. Lesions may exhibit caseation, calcification or fibrosis, the resultant nodular lesions being visible macroscopically. This tendency for lesions in sheep to undergo caseation or calcification is an important point of differentiation from the lesions of Johne's disease in cattle. Acid-fast *Map* are sparse or undetectable in lymphocytic lesions, and are usually absent from caseous or calcified foci.

The two types of pathology in paratuberculosis correlate with different host responses to the bacterium. Sheep with multibacillary disease have a strong antibody response but a weak, or absent, cell-mediated immunity (CMI) as indicated by poor skin hypersensitivity and predominant Th2-like cytokines (IL-4 and IL-10)[80]. Electron microscopy indicates that *Map* appears to be able to multiply in epitheloid cells in these lesions[81]. Animals with paucibacillary disease show a strong CMI response and strong skin hypersensitivity, poor or absent antibody response and predominant Th1-like cytokines (IL-2 and IFN-γ). In these lesions, the bacteria appear to degenerate in

epitheloid macrophages. Thus, this form of paratuberculosis appears to reflect the ability of the host to reduce the bacterial load in the intestine.

3.5 Clinical Features

It is important to recognise that paratuberculosis has a protracted incubation period and that clinical disease is the terminal stage of this process. The onset of clinical disease is unpredictable, but may be precipitated by parturition, lactation or by stressful events.

In all ruminants, clinical disease is characterised by a progressive, afebrile weight loss that leads to emaciation, brisket or submandibular oedema and poor coat quality (roughness, loss of pigmentation, alopecia, 'wool-slip'), despite maintaining a good appetite. A drop in milk yield has been reported to occur in the lactation preceding that in which clinical disease became apparent[3]. A major feature of the illness in cattle is chronic, intractable diarrhoea, although in some animals this may be intermittent. In contrast to cattle, diarrhoea is not a feature in small ruminants. This is probably due to their greater ability to reabsorb water in the large intestine, though in advanced cases the faeces may become soft and unformed.

Decreased serum concentrations of calcium, total protein and albumin have been reported in both cattle and sheep with clinical paratuberculosis[82],[83].

Although the disease is always chronic, there is considerable variation in the course. In some instances, the disease progresses relatively rapidly with the interval between the appearance of wasting and death measured in months. In other cases, after the initial loss of condition, there may be no clinical deterioration for long periods.

Since the first signs of clinical disease are progressive weight loss and drop in milk production, most farmers will note this and may cull the animal without requesting further diagnosis. For this reason the actual number of clinical cases (and the prevalence) of paratuberculosis is greatly underestimated[63].

4. Description of Crohn's Disease

Crohn's disease is a non-specific chronic transmural inflammatory disease of humans that most commonly affects the distal ileum and colon but may also occur in any part of the gastrointestinal tract from the mouth to the anus and perianal area. It is a life long disease and has no cure. Quality of life is compromised[84],[85] and patients afflicted with the disorder have to learn to live with the threat of significant morbidity throughout their lives. It generally affects persons during the prime of life, with highest incidence being in the age group 15-24, often from homes where early hygiene has been good[86],[87]. The disease is characterised by intermittent episodes of relapse, remission and recurrence requiring one or more surgical intervention in a large proportion of patients[88]. However, with the advent of new therapeutic agents such as aminosalicylates (5-ASA), budesonide and immunosuppressive drugs, medical management has become more common.

4.1 History

Crohn's disease derives its name from the description of eight cases of regional ileitis described by Crohn, Ginsburg and Oppenheimer in 1932 at the Mount Sinai Hospital in New York[89] . However, the first clear description of the disease was made by Dalziel in 1913 at the Western Infirmary in Glasgow[90]. The earlier term of regional ileitis was replaced by regional enteritis in l960 when it was recognised by Lockhart-Mummery and Morson that the disease was not confined to the ileum when they described primary Crohn's disease of the colon[91]. This entity was subsequently termed granulomatous colitis by American clinicians. Together with ulcerative colitis and unclassified chronic colitis, Crohn's disease belongs to a spectrum of diseases more generally designated as "Chronic inflammatory bowel diseases".

4.2 Aetiology

The aetiology of this disease is unknown. Immunological factors have been extensively examined, as have possible infectious agents. The list of bacteria examined is long and has been reviewed by Thompson[92] (see table 1).

Table 1. Some bacteria investigated as possible causes of Crohn's disease

Klebsiella spp.	*Campylobacter jejuni*
Chlamydia spp.	*Campylobacter faecalis*
Eubacterium spp	*Listeria monocytogenes*
Peptostreptococcus spp	*Brucella abortus*
Bacteroides fragilis	*Yersinia pseudotuberculosis*
Enterococcus faecalis	*Yersinia enterocolitica*
Escherichia coli	*Mycobacteria* spp.

Several viruses have also been investigated, the most prominent being the measles virus. However, publications from the Chief Medical Officer Department of Health in the UK and the World Health Organisation have indicated a consensus conclusion that measles viruses are not associated with Crohn's disease [93],[94].

With increasing concern about the transmission of infectious diseases from animal to man, attention has refocused on *Mycobacterium paratuberculosis* as a candidate organism in the aetiology of Crohn’s disease (see Chapter 7).

4.3 Pathogenesis

4.3.1 Disease mechanisms

The pathogenesis of Crohn’s disease probably involves an interaction of genetic and environmental factors, but the precise mechanism responsible for initiating chronic intestinal inflammation remains unclear. Irrespective of the aetiologic agent, three theories of disease mechanisms in Crohn’s disease are currently under consideration: 1) reaction to a persistent intestinal infection, 2) existence of a defective mucosal barrier to luminal antigens, and 3) a dysregulated host immune response to ubiquitous antigens[95].

Another attractive hypothesis which fits well with the currently recognised role of cytokin imbalance and with observations in experimental colitis, is the breakdown of tolerance against microbial flora of the gut[96]. Cigarette smoking has also been linked to the long-term course of Crohn’s disease and as well as to the recurrence of the disease after surgery[97],[98].

4.3.2 Genetic susceptibility

Whether Crohn’s disease is primarily an inherited or acquired disorder has been explored in recent studies of familial aggregation in Belgium[99] , France[100] , the United States[101],[102] and the United Kingdom[103]. The investigators found greater than expected concordance for site and clinical type of Crohn’s disease within individual families as well as pattern of young age at disease onset and more complicated disease among familial cases as compared with sporadic cases. These studies were criticised for their statistical approach to risk estimates, but they provide strong evidence that genetic factors are important determinants of susceptibility and disease behaviour in Crohn’s disease. Concordance rates in siblings and in monozygotic twin pairs in Crohn’s disease suggest that the genetic contribution to disease pathogenesis is at least equivalent to that in other common immune-mediated diseases (multiple sclerosis, insulin-dependent diabetes mellitus). A simple Mendelian pattern of disease inheritance is not evident. The epidemiological data are most consistent with Crohn’s disease and ulcerative colitis being polygenic diseases, sharing some, but not all, susceptibility loci[104],[105].

Systematic screening of the human genome for susceptibility loci is now practicable in polygenic disorders, and has been successfully applied in Crohn's disease. Four independent studies[106],[107],[108],[109],[110] have all demonstrated linkage between susceptibility to Crohn's disease and a region on chromosome 16; in addition, two studies[103],[111], have also implicated an area on chromosome 12 as involved in the pathogenesis of both Crohn's disease and ulcerative colitis.

These data promise real advances in the understanding of the pathogenesis of Crohn's disease and may also have direct clinical applications in both diagnosis and therapy.

4.3.3. Immunopathology

Whatever the precise aetiology of the disease, numerous studies have confirmed the role of a constant stimulation of the mucosal and systemic immune systems to perpetuate the inflammatory cascade and lead to the gut lesions. Chronicity of inflammation seems to result from an interaction of the persistent stimulus of microbial antigens with genetically determined host susceptibility factors that determine the individual's immune response, and/or mucosal barrier function[95]. The pathogenesis could involve a series of steps, beginning with the breach of the mucosal barrier by infectious agents, or toxins. The defective barrier could expose lamina propria immune cells to the continuous presence of specific micro-organisms and/or resident luminal bacteria of the normal flora, bacterial products, or dietary antigens, which perpetuates the inflammatory cascade. Abnormal antigen presentation, with abnormal expression of HLA molecules by various cells, including epithelial intestinal cells, has been evoked. Immunological studies in the recent years have focused, however, on abnormalities in the cytokine secretion profile of T-cells, that contribute to differentiate Crohn's disease from other inflammatory bowel diseases, such as ulcerative colitis. In Crohn's disease, Th1 cells triggered by Interleukin-12 (IL-12) and producing IL-2 and Interferon γ (IFN-γ) are predominant and induce pro-inflammatory cytokine production, chemokine secretion and thus, activate cellular immunity through the recruitment and activation of macrophages[112],[113],[114],[115]. Tumor necrosis factor α (TNF- α) has been shown to be a key proinflammatory cytokine in the development of the lesions and in the persistence of IFN-γ production by Th1 cells. IL-6 levels are correlated with the severity of the disease and best predict relapses[116]. Conversely, production of "anti-inflammatory" cytokines, such as IL-10, IL-4 and IL-13, seems deficient, or at least insufficient to counteract the pro-inflammatory loop of the immune response[114],[117]. These observations have served as rationale for new therapeutic attempts: IL-10 has been shown to suppress chronic granulomatous inflammation induced experimentally by bacterial cell wall polymers, and pilot clinical trials have shown the efficacy of chimeric monoclonal antibodies to TNF–α, and of IL-10, in patients with Crohn's disease[118],[119],[120],[121],[122],[123]. It must be noted that Th-1-related mechanisms are those usually considered as efficient against mycobacterial agents. The continuous Th-1 stimulation could be an inappropriate but chronic response to mycobacteriae, unable to eradicate the offending microorganism, but leading to mucosal damage; alternatively, a relative success in killing the microorganism could be associated with genetically induced failure in producing those anti-inflammatory cytokines that could stop the

cellular immune response. Similarly to the observations of "lymphocytic" versus "lepromatous" forms of leprosy, it has been hypothesised that different presentations of Crohn's disease, an aggressive fistulising form and an indolent obstructive form, could represent differing host immune responses to a common antigenic stimulus (possibly mycobacterial) and could provide a new and key insight into the pathogenesis of the disease[124] .

4.4 Pathology

The earliest macroscopic lesions of Crohn's disease appear to be tiny focal "aphthoid" ulcerations of the mucosa, usually with underlying nodules of lymphoid tissue. Sometimes these lesions regress; in other cases, the inflammatory process progresses to involve all layers of the intestinal wall, which becomes greatly thickened. Changes are most marked in the submucosa, with lymphoedema and lymphocytic infiltration occurring first, and extensive fibrosis later. Patchy ulcerations develop on the mucosa, and the combination of longtitudinal and transverse ulcers with intervening mucosal oedema frequently creates a characteristic "cobblestone" appearance. The attached mesentery is thickened and lymphoedematous; mesenteric fat typically extends onto the serosal surface of the bowel. Mesenteric lymph nodes are often enlarged. The transmural inflammation, deep ulceration, oedema, and fibrosis are responsible for obstruction, deep sinus tracts and fistulas, and mesenteric abscesses, which are the major local complications.

Segments of diseased bowel are characteristically sharply demarcated from adjacent normal bowel – thus the name "regional" enteritis. Segmental lesions may be separated by normal areas (skip lesions). With reference to disease distribution in the gut a recent European collaborative study[125] has shown that ileum alone is involved in about 10% of cases (ileitis); both ileum and colon are affected in about 60% (ileocolitis); and the colon alone is involved in about 30% (granulomatous colitis). Occasionally the entire small bowel (jejunoileitis) is involved, and rarely also the stomach, duodenum, or oesophagus.

Sarcoid-type epithelioid granulomas in the intestinal wall and occasionally in the involved mesenteric nodes are pathognomonic, but since they are absent in up to 50% of patients, they are not essential to diagnose Crohn's disease. Although they may represent a hidden clue to pathogenesis, they appear to have no definitive bearing on the clinical course.

While granulomas are helpful in distinguishing Crohn's disease from other forms of inflammatory bowel diseases, when present, it is the chronic inflammation involving all layers of the intestinal wall which is most characteristic of Crohn's disease. Both lymphocytes and macrophages are present in the granulomas and/or the cellular infiltrate. In active Crohn's disease, mucosal T lymphocyte numbers appear reduced but the subset distribution is normal; activated macrophages and infiltrative monocytes, associated with giant cells and epithelioid cells in the granulomas, seem to be much involved in the pathogenesis of the lesions through the production of pro-inflammatory cytokines, lysosomal enzymes, oxygen free radicals, nitric oxide and extra-cellular

matrix components. Mesenteric lymph nodes show extensive infiltration of T-dependent areas with activated macrophages. Inflammatory cell turnover correlates closely with clinical and histological indices of disease activity[126],[127].

4.5 Clinical Features

Chronic diarrhoea associated with abdominal pain, fever, anorexia, weight loss, and a right lower quadrant mass or fullness are the most common presenting features[128]. However, many patients are first seen with an “acute abdomen” simulating acute appendicitis or intestinal obstruction, both of which must be ruled out. Four patterns of regional enteritis occur most often:

(1) inflammation, characterised by right lower quadrant abdominal pain and tenderness, mimicking appendicitis when acute;

(2) obstruction, in which intestinal stenosis causes recurrent partial obstruction with severe colic, abdominal distention, constipation, and vomiting;

(3) diffuse jejunoileitis, with both inflammation and obstruction resulting in malnutrition and chronic debility;

(4) abdominal fistulas and abscesses, usually late developments, often causing fever, painful abdominal masses, and generalised wasting. Fistulas may be enteroenteric, enterovesical, retroperitoneal, or enterocutaneous.

Obstruction, fistulation, and abscess formation are common complications of inflammation; intestinal bleeding, perforation, and small bowel cancer develop rarely. A history of perianal disease, especially fissures and fistulas, can be elicited in about one third of patients. When colon alone is affected, the clinical picture may be indistinguishable from ulcerative colitis raising questions of differential diagnosis.

Extraintestinal complications of Crohn’s disease fall into three principal categories:

(1) Complications that often parallel the activity of the intestinal disease and possibly represent acute immunological or microbiological concomitants of the bowel inflammation include peripheral arthritis, episcleritis, aphthous stomatitis, erythema nodosum, and pyoderma gangrenosum. These manifestations may be reported by over 1/3 of patients hospitalised with inflammatory bowel disease. They are twice as common when colitis is present as when disease is confined to the small intestine. When extraintestinal manifestations occur they are multiple in about one third of patients.

(2) Disorders associated with inflammatory bowel disease but running an independent course include ankylosing spondylitis, sacroilitis, uveitis, and primary sclerosing cholangitis.

(3) Complications that relate directly to the disrupted physiology of the bowel itself are chiefly renal problems. Kidney stones result from disorders of uric acid metabolism, impairment of urinary dilution and alkalinisation, and excessive dietary oxalate absorption; urinary tract infections occur especially with fistulation into the urinary tract; and hydroureter and hydronephrosis may result from ureteral compression by retroperitoneal extention of the intestinal inflammatory process.

Other bowel related complications include malabsorption, especially in the face of extensive ileal resection or bacterial overgrowth from chronic small bowel obstruction or fistulation; gallstones, related to impaired ileal reabsorption of bile salts; and amyloidosis, secondary to long standing inflammatory and suppurative disease.

Table 2. Comparative Summary

Pathological features in Crohn's disease and paratuberculosis

	**Crohn's Disease**	**Paratuberculosis**
**Lesion Location**		
- oesophagus and oral cavity	Yes	No
- ileum and colon	Yes	Yes[1]
- mesenteric lymph nodes	Yes	Yes
- rectum, anus	Yes	Advanced cases[2]
- segmental	Yes	Yes
**Macroscopic Features**		
- macroscopic appearance	Oedema of affected bowel wall, "garden hose" like appearance	Thickened bowel wall[2]
- parietal oedema	Yes	Yes
- stenosis	Yes	Rare
- perforation	Yes	Rare
- fistula	Yes	No
- pseudopolyps	Yes	No
- mucosal aspect	cobble stone appearance	Corrugated[2,3]
**Microscopic appearance**		
- transmural involvement	Yes	Yes
- fibrosis	Yes	No
- lymphoid aggregates	Yes	Yes[3]
- granuloma	Yes (50% -70% of cases)	Yes
- caseation	No	Usually not[4]
- fissures	Yes	No
- visible acid fast bacilli	No	Yes[5]

[1] Ileum and jejunum are the initial and most frequent locations
[2] not always in sheep
[3] predominant feature in lymphocytic/paucimicrobial form
[4] Varies with species
[5] Scarce or absent in lymphocytic/paucimicrobial form

Table 3. Comparative Summary

Clinical Features of Crohn's disease and paratuberculosis

	**Crohn's Disease**	**Paratuberculosis**
**Preclinical Stage**		
- symptoms and signs	Not known	Decreased milk yield
- incubation period	Not known	Minimum 6 months
**Clinical Stage**		
- presenting symptoms and signs	Chronic diarrhoea	Chronic diarrhoea[1]
	Abdominal pain	Dull hair
	Weight loss	Weight loss
		Decrease in lactation
**Gastro-intestinal symptoms and signs**		
- diarrhoea	Chronic (3 weeks +)	Chronic[6]
- blood in stools	Rare	Rare
- vomiting	Rare	No
- abdominal pain	Yes	No evidence
- obstruction	Yes	No
**Extra-intestinal manifestations**		
- polyarthritis	Yes	No
- uveitis	Yes	No
- skin lesions	Yes	No
- amyloidosis	Yes	No
- hepatic granulomatosis	Yes	Yes
- renal involvement	Yes	No
**Clinical Course**		
- remission and relapse	Yes	Yes

[6] Not seen in sheep

5. Epidemiology and geographical distribution of both diseases

5.1 Epidemiology and geographical distribution of paratuberculosis in animals

Since its first description, paratuberculosis has been recognised world-wide[129]. However, the difficulty in its diagnosis means that accurate studies concerning its actual distribution are not available[63]. In Europe, studies were made in France in 1934 with a prevalence of 0.8% of bovines[130] . Various surveys of abattoir cattle in England during the 1950's suggested prevalence rates between 11-17%[131],[132]. Jorgensen (1965)[64] found that the 2.3% of adult cattle slaughtered in Denmark were positive in culture and this figure increased to 9.8% in 1972[133]. More recently, a survey was carried out in Belgium on 300 serum samples obtained from cattle that are representative of the bovine population of the Walloon part of the country. This survey made with an ELISA based on a recombinant peptide (a362) endowed with B-cell epitopes specific to *Map* indicated that the seroprevalence of paratuberculosis in that part of Belgium was 12%[134].
In the USA, a national survey was performed in the 1980's, by a bacteriological examination of over 7000 samples from different bovine abattoirs around the country. The infection was considered as present in 1.6% of all cattle and 2.9% of cull cows[135]. In Spain, prevalence in cattle was estimated to be 1% of adult cattle in a slaughterhouse bacteriological survey and at 67% of herds by ELISA in the Basque country[136].

More recent data are available from prevalence studies in Belgium[137] and the Netherlands[138]. In both countries prevalence studies were conducted using the absorbed ELISA as the diagnostic tool. In Belgium the seroprevalence was found to be 17.4% of herds and 1.2% of individual animals. In the Netherlands the seroprevalence was 54.7% at the herd level and 2.5% at the individual animal level. In this latter study the test characteristics were taken into account when the true prevalences were calculated. The uncertainties in the characteristics with respect to sensitivity and specificity are responsible for large ranges in the calculated true prevalence: for example using a sensitivity and specificity of 30% and 99.5%, respectively, the estimated true prevalence at herd level is 70.6% and at animal level 6.9%. Using a sensitivity and specificity of 40% and 98.5%, respectively, the prevalences are 31.3% at the herd level and 2.7% at the animal level. Nevertheless these results show that paratuberculosis is a widespread problem. The differences in results between both studies can at least be explained in part by the differences in cattle populations tested in both countries: in Belgium the vast majority of herds tested were beef cattle herds whereas in the Netherlands the herds tested were dairy cattle herds.

Some studies have also been carried out in sheep. In Greece, 9.8% of ovines tested were positive to delayed-type hypersensitivity tests[139]. In Spain, a study in the Region of Aragon by means of serological, bacteriological and pathological techniques

identified 46.7% of flocks as infected[140]. Within flocks, prevalence levels of affected animals ranging from 33%[141] to 46%[76] have been reported. In the Basque Country, prevalence in sheep was estimated by ELISA to be 32% of flocks[142].

5.2. Epidemiology and geographical distribution of Crohn's disease

A review of the literature suggests that Crohn's disease is more prevalent in Western populations with northern European and Anglo-Saxon ethnic derivation, than in populations of southern Europe, Asia and Africa. However, when the Asian people migrate to urban-industrial societies of the West, they become as susceptible to the disease as the population of their host countries, suggesting environmental factors in the aetiology of Crohn's disease[143].

There are many epidemiological studies of the incidence and prevalence of Crohn's disease from Europe and America. The problem, however, is that most of the early studies were retrospective. They used different study designs (for example criteria of disease definition, case ascertainment, or the time period of investigation) and, with few exceptions (Aberdeen, Copenhagen, Leiden, Stockholm, Uppsala) were based upon the experience of specialised medical centres. The data they provided on the risk of disease in the population were confusing, and in some respects unreliable.

To overcome this problem, a group of clinicians and epidemiologists in Europe carried out an epidemiological survey of the incidence of inflammatory bowel disease (EC-IBD study) in 20 centres across Europe prospectively and simultaneously between 1991 and 1994[144]. The project was a Concerted Action project of the Commission of the European Communities with the aim of investigating the north-south gradient in the risk of disease (incidence) using a standard protocol for disease definition, case ascertainment and data analysis. The study was conducted in the population of defined geographical areas ranging from Reykjavic, Iceland in the north to Heraklion, Greece in the south, and included regional case reviews and site visits for the quality control of data.

The results of this study are presented in Table 5. They show that the highest age and sex adjusted incidence rate for Crohn's disease (per 100,000) was in Amiens (NW France) and Maastricht (the Netherlands), followed by Oslo (Norway), Iceland, and Copenhagen (Denmark). The lowest reported incidence was from Ioannina (NW Greece). The incidence was broadly similar for men and women.

However, recent epidemiological studies conducted in different regions of France, using a common methodology, showed that the prevalence and incidence of Crohn's disease tended to be as high in southern parts of that country as in the previously considered "high incidence areas" of the North[145] (see table 4 below).

Table 4. : Standardised incidence of CIBD in Europe and in France

Registry<br><br>Region	Year	CIBD<br><br>n	CD<br><br>n	UC<br><br>n	UCC<br><br>n	AC	Standardised Incidence*°		Estimated Incidence*	
							UC	CD	UC	CD
**Europe**	83-96	2085	706	1379	-	-	5.0	9.8	-	-
**Northern Europe**	83-96	1346	477	869	-	-	6.3	11.4	-	-
**Southern Europe**[144]	83-96	739	229	510	-	-	3.6	8.0	-	-
**Northern France** [146]	88	576	281	207	88	104	6.0	4.4	5.77	3.9
**Northern France**[147]	88-90	1291	674	466	151	-	4.9	3.2	5.1	3.6
**Central France**[148]	93-94	167	79	29	4	55	5.7	1.9	6.4	2.8
**Western France**[149]	94-95	657	205	165	42	245	2.8	2.7	3.5	4.0

* n/100000/year ° incidence standardised for age and sex.
CIBD : chronic inflammation bowel diseases ; CD : Crohn's disease ; UC : ulcerative colitis ; UCC : unclassified chronic colitis ; AC : acute colitis.

In summary, the EC-IBD study has produced an overall picture of the incidence of Crohn's disease and its geographical distribution across Europe. It has further shown that the incidence of Crohn's disease is 80% higher in the north than that in the south. The observed excess of incidence in the north did not seem to be explained by differences in tobacco consumption and education. The age-specific incidence rates per 100,000 show that the age group 15-24 were most at risk of coming down with the disease and the risk of disease in both sexes was broadly similar.

Similar North-South gradients in incidence have also been documented in North America. For example, the highest reported incidence of Crohn's disease in the world is in Manitoba, Canada of 14.6 per 100,000 population per year[150],[151], followed by an incidence rate of 10 in Northern Alberta[152] and of 7 cases per 100,000 population in Olmsted County, Minnesota[153], 400 miles to the South of Manitoba. These are substantial differences and suggest that climate, diet, water supply or other environmental factors may be involved in the risk of this disease in different geographical areas of the world.

Table 5 Incidence rates (/100,000/year) and numbers of cases (in parentheses) aged 15-64 years for Crohn's disease reported in the 20 centres included in the EC-IBD study 1991-94[144].

	**Men**		**Women**		**Total**	
**Centre**	**Crude rate**	**Age Adjusted rate (95% CI)**	**Crude rate**	**Age Adjusted rate (95% CI)**	**Crude rate**	**Age Adjusted rate (95% CI)**
Northern Centres						
Reykjavic (Iceland)	8.8 (15)	8.4 (5.5-11.3)	7.9(13)	7.2 (4.6-9.8)	8.4 (28)	7.8 (4.0-11.7)
Oslo (Norway)	7.6 (48)	7.6 (6.1-9.1)	8.2 (52)	8.3 (6.8-9.7)	7.9 (100)	7.9 (5.9-9.9)
Copenhagen (Denmark)	5.3 (20)	5.4 (3.8-7.1)	8.9 (34)	9.3 (7.3-11.4)	7.2 (54)	7.3 (4.7-10.0)
Dublin (Ireland)	4.8 (19)	4.5 (3.1-5.9)	6.5 (27)	5.9 (4.5- 7.4)	5.7 (46)	5.2 (3.2-7.2)
Leicester (UK)						
Non Immigrants	1.7 (2)	1.6 (0.1-3.1)	6.6 (8)	6.1 (3.3-8.9)	4.2 (10)	3.8 (0.7-6.9)
Immigrants	8.3 (4)	9.4 (2.9-15.9)	2.0 (1)	1.7 (0.0-3.8)	5.0 (5)	5.6 (0.0-12.5)
Maastricht (the Netherlands)	8.1 (37)	8.3 (6.5-10.2)	10.1 (45)	10.1 (8.2-12.0)	9.1 (82)	9.2 (6.5-11.8)
Essen (Germany)	3.1 (17)	3.2 (2.2-4.2)	5.3 (29)	5.6 (4.2-6.9)	4.2 (46)	4.4 (2.7-6.1)
Amiens (NW France)	8.1 (29)	8.0 (6.0-9.9)	10.8 (39)	10.6 (8.4-12.7)	9.5 (68)	9.2 (6.3-12.2)
**All Northern Centres**	**6.2 (191)**	**6.2 (4.2-8.1)**	**7.9 (248)**	**7.9 (5.9-9.8)**	**7.0 (439)**	**7.0 (4.2-9.8)**
Southern Centres						
Milan-Varese (Italy)	1.4 (2)	1.4 (0.1-2.6)	3.5 (5)	3.6 (1.5-5.6)	2.5 (7)	2.5 (0.1-4.9)
Crema-Cremona (Italy)	2.2 (5)	2.3 (0.9-3.6)	2.9 (6)	3.1 (1.5-4.7)	2.6 (11)	2.7 (0.6-4.8)
Reggio Emilia (Italy)	4.8 (10)	4.8 (3.1-6.5)	3.9 (11)	4.1 (2.5-5.7)	4.3 (25)	4.4 (2.1-6.7)
Florence (Italy)	2.4 (10)	2.6 (1.5-3.7)	4.0 (17)	4.1 (2.8-5.3)	3.2 (27)	3.3 (1.7-5.0)
Vigo (N.W. Spain)	7.8 (27)	7.3 (5.4-9.2)	3.1 (11)	2.8 (1.7-3.9)	5.4 (38)	5.1 (2.9-7.3)
Sabadell (N.E. Spain)	4.0 (10)	3.8 (2.2-5.4)	6.8 (17)	6.6 (4.6-8.7)	5.4 (27)	5.2 (2.6-7.7)
Braga (N. Portugal)	3.0 (5)	2.7 (1.1-4.3)	5.7 (10)	5.7 (3.4-8.0)	4.4 (15)	4.2 (1.3-7.0)
Ioannina (N.W. Greece)	1.2 (2)	1.1 (0.1-2.2)	0.6 (1)	0.7 (0.0-1.5)	0.9 (3)	0.9 (0.0-2.2)
Almada (S. Portugal)	1.9 (4)	1.9 (0.7-3.2)	3.3 (7)	3.3 (1.7-4.9)	2.6 (11)	2.6 (0.6-4.6)
Palermo, Sicily (Italy)	7.4 (6)	7.1 (3.2-11.0)	6.0 (6)	6.1 (2.9-9.3)	6.6 (12)	6.6 (1.6-11.5)
Heraklion, Crete (Greece)	7.6 (11)	7.5 (4.5-10.4)	2.0 (3)	2.1 (0.5-3.6)	4. 7 (14)	4.8 (1.5-8.1)
Beer Sheva (Israel)	2.3 (5)	2.4 (0.9-3.8)	6.5 (14)	6.5 (4.2-8.9)	4.4 (19)	4.4 (1.7-7.2)
**All southern centres**	**3.9 (101)**	**3.8 (1.9-5.8)**	**4.0 (108)**	**4.0 (2.0-6.0)**	**3.9 (209)**	**3.9 (1.1-6.7)**
**All Centres**	**5.1 (292)**	**5.1 (4.7-5.5)**	**6.1 (356)**	**6.1 (5.7-6.5)**	**5.6 (648)**	**5.6 (2.8-8.3)**

5.3 Is the incidence of both diseases changing?

From 1950s to 1980s several centres reported considerable increases in the incidence of Crohn's disease. Whereas, the latest studies have suggested that the increase in the incidence of Crohn's disease in the north has slowed or reached a plateau [154],[155],[156],[157]. The southern European centres have, however, continued to show an increase in annual incidence over the last several years [158],[159]. This may reflect more sensitive diagnostic methods, greater case ascertainment or increasing urbanisation and industrialisation of southern European societies.

Data are not available on which to base a comparison of the incidence of paratuberculosis over time.

5.4 Is there a causal association between the geographical incidence/prevalence of Crohn's disease and that of paratuberculosis?

If Crohn's disease in humans is caused by simple contact and infection with *Map* strains originating from paratuberculosis infection in animals one would expect a causal association between the geographical incidence and prevalence of both diseases.

There are a number of problems with this assumption which merit caution and render conclusions difficult to interpret.

1. Lack of reliable data on incidence and prevalence of both diseases especially paratuberculosis

While information has recently become available on the incidence rates of Crohn's disease in various population centres in Europe (Table 5) corresponding information on the incidence and prevalence of paratuberculosis in the regions of these centres is not available. Indeed, there are considerable difficulties in obtaining comparable data. It does seem clear however that paratuberculosis is extremely common in most parts of Europe (and the world) in both the cattle, sheep and goat populations. Those areas which are reputed to have a low prevalence of paratuberculosis have in general not been systematically examined to determine the prevalence of the disease. There are no areas where the disease has been conclusively shown to be absent in animals. The reported incidence of the disease, in all areas, are likely to be a fraction of the number of animals infected and shedding the organism.

2. Long incubation period.

If *Map* were to be involved in the aetiology of Crohn's disease, it is possible, as in animals, that the exposure to the organism would have taken place many years before the appearance of clinical signs. Comparisons, therefore, would need to be made between current incidence in man and the incidence of paratuberculosis in the animal population ten or fifteen years ago. These data are simply not available.

In short, there are considerable problems in assessing a causal association between the two diseases based on the geographical incidence and prevalence of both diseases. These issues include the evident time lag before clinical signs, the multifactorial nature of both diseases, the use of vaccine for paratuberculosis in some regions, dietary habits in humans and the influence of migrations.

6. Diagnosis and control of paratuberculosis

6.1 Introduction

The diagnostic techniques that can be used to detect the disease are influenced by the stage of the disease as it has developed in the affected host and the prevalence in the herd[160],[161],[162]. Control of the disease on the farm as well as to stop further spread of the disease by trading of infected animals largely depends on the recognition of young infected animals as early as possible. Detection of the infection of the host during this sub-clinical stage is complicated by the fact that the bacterium does not multiply rapidly and is therefore difficult to detect in faeces. There is little or no immune response at this stage. Serum antibodies are absent and even though a delayed type hypersensitivity response (DTH) occurs early in infection, it is not as significant as in (bovine) tuberculosis and the response wanes as the infection progresses.
In the clinical stage, serum antibodies are detectable in an ELISA assay and the bacterium can be cultured from faecal samples, though the organism may also be cultured before clinical signs appear. The sensitivity of both tests increases rapidly at this stage. Fig. 2 (adapted from Collins, 1998[163]) gives an illustration of the usefulness of the respective tests in cattle infected in early life at different stages of the disease. Even though this picture is valid in general, there is a great variety of different responses among individual animals, especially in the case of the paucibacillary form of the disease in sheep.

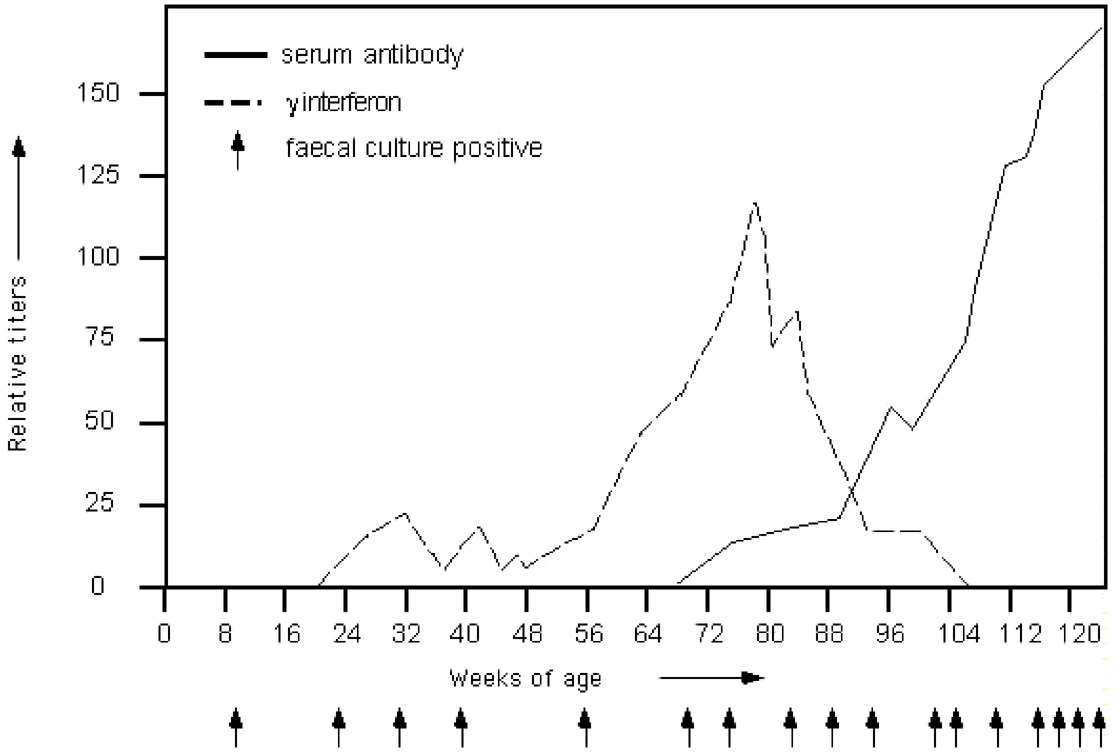

Figure 2: Typical responses to diagnostic tests in animals infected in early life -

The available tests can be divided into two groups. The first group of tests is aimed at the detection of the causative agent. The tests are: microscopic examination of faecal smears (Ziehl-Neelsen stain) though this is subject to the limits stated below, culture of the organism from faecal samples and the detection by polymerase chain reaction (PCR). Both these tests depend on the presence of the organism in faeces which is more likely as the disease progresses. The second group consists of the immunological tests. These are: the (absorbed) ELISA, the complement fixation test (CFT), the agar gel immunodiffusion test (AGID) and the delayed type hypersensitivity test (intradermal or interferon γ test).

6.2 Detection of the causative agent

6.2.1 Microscopical examination

The microscopic examination of (Ziehl-Neelsen stained) faecal smears is a rapid, economical way of obtaining a diagnosis. The diagnosis depends on the presence of clumps of small acid fast bacteria, as these can be shed intermittently. The presence of single acid fast bacilli from environmental origin in faecal samples is quite common and makes a diagnosis sometimes difficult and not completely specific. The test has a low sensitivity in early stages of the disease, but when the animal reaches the clinical stage, the test reaches virtually 100% sensitivity.

6.2.2. Cultural methods.

The traditional culture of *Map* from faeces is, as the disease progresses, the most sensitive test available and has a 100 % specificity and is therefore regarded as the "gold standard". However, the test is very labour-intensive, time consuming and therefore expensive. The test itself is not standardised and the laboratory proficiency varies considerably. The decontamination procedure is one of the variables in the procedure but an essential step in all methods used[164]. Since culturing can take up to six months to complete, it is crucial to eliminate the fast growing bacteria from the samples being tested. At present there are two basic methods in use for the culture of *Map* from clinical specimens: the method using oxalic acid and NaOH for decontamination and Löwenstein-Jenssen medium for growth and the method using hexadecylpyridinium (HPC) for decontamination in combination with Herrold's Egg Yolk Medium (HEYM) for growth. Both media contain mycobactin. Colonies can be recovered after 4-24 weeks of culture. Confirmation of the identity of the colonies was usually done by transferring them onto media with and without mycobactin. Incubation would take another 4 weeks and the mycobactin dependant colonies would be regarded as being *Map*. Nowadays the confirmation can be done within a day by the PCR amplification of the IS900 element, which is specific for *Map* [165].

Because culture is often used as a gold standard to estimate the sensitivity of other tests, its lack of standardisation makes the interpretation of results obtained (e.g. ELISA sensitivity, prevalence studies) by different laboratories extremely difficult.
Another, more rapid technique for the isolation of *Map* employs the use of a radiometric based detection system, the Bactec 460[166]. Growth of mycobacteria is measured by the release of $^{14}CO_2$ from palmitate by bacterial metabolism. However as this system is radiometrically based, it is not feasible to use in some laboratories and is phased out in others.
Recently three other, fluorescence-based rapid methods were introduced, the Bactec 9000 and the MGIT system (Becton Dickinson) and the MBBact system (Organon Technika). Initial experiments in which these methods were tested on faecal samples, suffered from severe problems with overgrowth by other bacteria (spore forms and fungi). Thusfar, an evaluation of these methods could not be made.

6.2.3. Polymerase Chain Reaction (P.C.R.).

The PCR for the detection of *Map* uses the IS900 insertion sequence as target sequence. This insertion sequence is specific for the bacterium and is present in 14 to 18 copies in the genome, making it an abundant target[167].

The technique of the polymerase chain reaction (PCR) offers an attractive alternative for the traditional cultural methods. It is not only a more rapid test (several days as opposed to six months) it can also offer high levels of specificity. However, great care has to be taken with regard to the specificity of the primer sets used in the PCR and the identity of the amplified product needs to be confirmed not only by size but also by DNA hybridisation.

However a high level of sensitivity is rarely attained due to problems encountered in sample preparation. Reduced sensitivity can result from inefficient extraction of the mycobacteria from the sample, particularly when small numbers of organisms are involved, or as a consequence of the presence of inhibitors of PCR. Different inhibitors are present in different types of sample but examples include large amounts of irrelevant DNA, polysaccharides, bile salts, etc.. Different types of samples pose different barriers to extraction and PCR. Unfortunately, faecal samples belong to the samples that are very difficult to process. Recent improvements in the method, indicate that it is possible to reach a sensitivity of 50 bacteria per gram of faeces which is similar to that of culture[168]. The extraction procedures are time consuming and require well trained technicians. As is the case with faecal culture only a limited number of samples can be processed. This makes implementing the PCR as a routine operation in a diagnostic laboratory rather difficult. Recently, the application of the IS900 PCR assay to blood and other tissues from sheep with clinical paratuberculosis raise some hope for the development of PCR as a routine technique. This PCR has particular advantages for the diagnosis of paratuberculosis because it can detect, for example, strains that are refractory to culture, for example certain types of sheep strains[169]. PCR can also be used for the analysis of tissue samples from Crohn's disease patients. In addition, it is a very rapid method for the identification of strains isolated from suspected samples.

Recent communications[170] mention the occurrence of false positive reactions with other mycobacteria. Therefore the specificity of the PCR and/or the primer sets being used will have to be established for the different types of samples to be tested.

6.3 Detection of an immune response to M. avium subsp. paratuberculosis

Despite the fact that microbial culture is regarded as the most sensitive test for the detection of paratuberculosis, because the test is so labour intensive and requires an extremely long incubation time, its usefulness as a test in a certification and/or eradication programme is limited.
For such purposes a serological test such as an ELISA, would be more suitable. These tests are fast, economical and, in fact, the only kind of tests that have the capacity to support a large scale testing programme. However, as can be seen from Figure 2, the antibody response (ELISA, CFT) rises at a late stage of the disease and is not detectable in the early stages. The ELISA will have a sensitivity rising from almost zero at the early stages of disease to virtually 100% when the animal reaches the clinical stage. The test is therefore only suitable for use on older cattle and is normally used on animals older than 2 years or sometimes only on animals older than 3 years. Furthermore, not all infected animals will give a measurable antibody response at an older age, but may be shedding the bacterium and be infectious to younger animals. As is the case for all mycobacterial infections, the overall sensitivity of the ELISA is rather low. Estimates for the sensitivity of the ELISA are highly variable, due to the testing of different age groups in the various studies. In addition, local differences in the load of environmental mycobacterial can contribute to differences in specificities obtained with the ELISA. This implies that using an ELISA at a certain time point will not identify all infected animals within the herd or guarantee the status of an individual animal.

6.3.1 The complement fixation test

The complement fixation test has been the most widely used standard test for paratuberculosis in cattle. The test allows the processing of a large number of samples and has a sufficiently high sensitivity to be used on clinical cases. A large number of different test protocols and different antigens are used world-wide and unfortunately the outcome of the test depends also on the interpretation of the person performing it. In general, the test lacks a sufficient sensitivity (and in many cases specificity) to be used as a reliable tool for the identification of infected animals [160],[171],[172].
Despite the fact that the outcome of the test gives no guarantee about the status of the individual animal, the CFT test is often the only test demanded for import/export of cattle within Europe.

6.3.2 The absorbed ELISA

The convenience of sample collection, rapid laboratory turnaround time and low cost have make an ELISA the preferred test for herd analysis. In general, the detection of serum antibodies against *Map* is achieved using different crude fractions of the bacterium; lipoarabinomannan (LAM) or PPD's. False positive results usually occur as a result of cross reactions with related bacteria of the *Corynebacterium-Mycobacterium-Nocardia* group, or in countries where bovine tuberculosis is endemic. The occurrence of false positives is therefore also geographically dependant. Another problem arises from the use of vaccination against paratuberculosis, often needed to aid affected farms in their survival, as this results in serologically positive animals.

The specificity of the test is increased in the so-called absorbed ELISA by absorption with sonicates from other (environmental) mycobacteria, like *M.phlei* or *M.vaccae*. Since the commercial absorbed ELISA has become available, it has become the most widely used serological test for paratuberculosis in cattle. Another approach to avoid the occurrence of ELISA false positive results would be the use of *Map* specific recombinant antigens. To date only one of these antigens (peptide a362) has been described and used in an ELISA. Whereas this a362-ELISA seems promising, it needs further validations on a larger scale[134],[173],[174], [175].

Reports on the sensitivity of the absorbed ELISA are contradictory[160],[162],[176]. As mentioned above, they depend on the standard used (faecal culture, pathology etc.) to calculate the relative sensitivity and on the animals that are tested. Since serum antibodies become more abundant in the later stages of the disease, using the ELISA in a herd or group of animals where the disease has been present for a longer period will result in a reasonable sensitivity. However the test gives a very low sensitivity in young, recently infected animals and can therefore not be used to detect the disease on a farm where spread of the disease has just commenced. Therefore, the test would only be useful in herd certification programmes where there are a sufficient number of older animals present.

Diagnosis of paratuberculosis is usually based on the combined results of faecal culture and the absorbed ELISA. In the Netherlands and other countries, experimental certification protocols based on alternate faecal culturing and testing by an absorbed ELISA are being discussed. Both these tests, however, have their own problems: faecal culturing is slow, labour intensive and expensive and the ELISA lacks sufficient sensitivity, especially for the final stage of eradication.

6.3.3. Agar-gel-immuno-diffusion test (AGID)

The agar gel immuno-diffusion test can be used for the detection of paratuberculosis in a variety of different animals[177] such as cattle, sheep, goats and deer. The methods employed in the different laboratories are variable and the antigens used are not specific for *Map*.
The relatively low cost of the AGID make this test a very attractive test for use in small ruminants. The AGID-test is, for example, the approved test for use in small ruminants, in fact the only approved test in goats, in the Australian National Johne's

Disease Program. Like the ELISA, the AGID is based on the detection of antibodies against *Map*. Therefore it has a relatively low sensitivity in the early stages of the disease, but becomes more useful in later stages.

The AGID has been used mainly in small ruminants (sheep and goats). Its sensitivity is lower than ELISA test and, as in the former, is closely related to the immunopathological forms of the infection. Sheep and goats with focal and tuberculoid forms (non-clinical infections) are usually negative[178]. Of clinical cases, sheep and goats with multibacillary forms are positive to AGID whereas those showing lymphocytic forms are usually negative[75]. Due to these features, in general, the sensitivity of the AGID test is variable, depending on the group of investigated animals[179], [180], [181]

6.3.4 Cellular Immunity Tests

As in leprosy and other mycobacterial diseases, a spectrum of immune responses occurs in *Map* infection (see also Fig 2). Early, or subclinical cases, have immune responses dominated by T cell-driven cellular responses, while more chronic, clinical cases are dominated by antibody responses. Therefore an optimal diagnosis of paratuberculosis would require a combination of cellular and antibody tests in order to identify both early and late cases - either of which may be missed by one of these tests used independently. However, the cellular response is less pronounced than in the case of bovine tuberculosis, where the intradermal test is still the most reliable test.

Cellular immunity tests can be performed in vivo or in vitro, using respectively the intradermal test or the gamma-interferon assay. Both tests are performed using avian purified protein derivative (PPD) or johnin (the paratuberculosis equivalent) to stimulate the response. In the intradermal test, skin thickness is measured before and 72 hours after injection with PPD. An increase in thickness of more than 2mm is regarded as a positive reaction. The gamma-interferon assay is performed by stimulating aliquots of heparinised blood with PPD and the subsequent release of gama-interferon is measured in an ELISA[182] . However as PPDs are crude steam-sterilized mycobacterial culture filtrate extracts containing many cross-reacting antigens with related bacteria of the *Corynebacteria-Mycobacteria-Nocardia* group[183], sensitisation to avian-PPD or johnin is very common in animals and therefore interferes with the specificity of both tests and makes interpretation of the results more difficult[184]. Moreover, as it has been mentioned above, there is a correlation between the positivity to cellular tests and animals showing focal-tuberculoid pathological forms. These are non clinical and non-shedding animals[73]. This results in a decreased sensitivity of the tests and reduces their value compared to the much cheaper ELISA.

6.4 Treatment of paratuberculosis in animals

Treatment of paratuberculosis in animals is not a feasible option. However, since the first description of the use of streptomycin in the treatment of 4 cows with Johne's

disease[185], there have been at least 14 reports of the use of conventional anti-tuberculous and anti-leprosy drugs in the treatment of *Map* infection in animals[186],[187],[188],[189],[190],[191],[192],[193],[194],[195],[196],[197],[198]. The animals tested included adult cattle and calves, sheep, goats and experimentally infected rabbits. The drugs used were streptomycin, isoniazid, clofazimine, rifampicin, ethambutol, pyrazinamide and dapsone, either as single agents or in combination[199]. In general, the number of animals in these studies was small and the scope of the work was limited by the cost of the drugs. Overall, the results of treatment were very similar. Where single agent therapy was used, either no effect or a transient clinical improvement with a reduction in faecal shedding was seen. Clinical improvement, if it occurred, usually lasted only a few weeks and was inevitably followed by relapse, either on treatment or after stopping the drug. The clinical and microbiological responses to drugs used in combinations such as streptomycin, isoniazid and rifampicin, were more marked and more prolonged than with single agent therapy, but cessation of faecal shedding of *Map* and the eradication of the infection were never convincingly achieved, and persistence of disease and relapse occurred in the majority of these studies.
None of the above reports referred to randomised controlled trials of these agents in experimentally or naturally infected animals so the results should be treated with caution.

6.5 Vaccination against paratuberculosis

Vaccination against paratuberculosis in cattle was first introduced in 1926 by Vallee and Rinjard[200]. The vaccination was performed by the subcutaneous injection of living, unattenuated, paratuberculosis bacilli and was administered at 1-30 days of age, subcutaneously in the brisket, where the vaccinal nodule is less obtrusive. Revaccination was not recommended. Since then vaccines have been used in other species, mainly sheep[3],[201],[202],[203] involving subcutaneous administration in both young (15 days to several months old) and adult animals. Many different formulations of the vaccine have been evaluated, using living bacteria of both unattenuated and attenuated strains, heat-killed organisms as well as disrupted fragments of *Map*.

At present, according to the guidelines of the specifications of the Office International des Epizooties (O.I.E.) the vaccines may contain live attenuated or killed bacteria incorporated with an adjuvant or freeze dried and adjuvanted on reconstitution[204]. However, because of the controversial reputation of *Map* and the unknown consequences of attenuated strains entering the food chain, the use of live vaccines is not allowed in several countries (e.g. the Netherlands).

The use of vaccination is still under debate. In the opinion of some authors[201],[203],[205] vaccination is the best way to control the infection, especially in sheep and goats though others conclude that vaccination alone is not sufficient to control paratuberculosis and that husbandry and other control measures are necessary to obtain maximum benefits. Since these changes in management coincide with the start of the vaccination programme, the effect of the vaccination itself is often difficult to assess. Clinical disease can still occur for several years after the start of vaccination on a farm[206] though several studies have noted the disappearance of clinical cases several months following the vaccination of adult animals[201],[207],[208]. However there is a

consensus that vaccination will reduce the number of animals with clinical disease, decrease the number of excretors and the level of excretion and therefore the number of animals with detectable intestinal infection[209].

In experimental studies[203], it has been observed that vaccination does not prevent infection but modifies the course of the disease and the pathology towards tuberculoid and regressive forms containing few (or undetectable) bacteria.

An important disadvantage is that vaccinated animals will respond positively to routine serodiagnostic tests for paratuberculosis[210],[211]. Thus, faecal culture remains the only available test to recognise infected animals though the PCR can be used in animals vaccinated using inactivated vaccines. However, because of the reduction in the excretion it will be more difficult to recognise the remaining carriers. Vaccinates must not be regarded as free of paratuberculosis and, in a certification programme, vaccinated herds should be treated as infected herds. A Dutch report states that vaccination does not reduce the overall prevalence of infection in a herd[212].

The sensitisation caused by vaccination not only affects the diagnosis of paratuberculosis. Vaccinated animals also become sensitised to bovine PPD used in the intradermal test in control and eradication programmes for bovine tuberculosis[213]. Comparative skin tests using bovine PPD and johnin or avian PPD can be used to differentiate between reactions to *M.bovis* infection and reactions caused by the vaccination[214]. However it was shown that in some cases in vaccinated animals, the bovine reaction was greater than the avian reaction[215], thus leading to false positive reactions for tuberculosis. This limits the large scale use of the available vaccines in regions where bovine tuberculosis is endemic.

Another undesirable feature of vaccination is the development of an inflammatory swelling and a subsequent fibrocaseous nodule at the site of inoculation. Similar effects are possible in the case of accidental self-injection by the practioner which could lead to severe local reactions, though these are not commonly reported.

In several countries vaccination has been used in order to control the disease. The adoption of a vaccination programme should be considered in heavily infected herds, in which the veterinary advisor considers adequate control will not be achieved by improved calf rearing hygiene alone. This will prevent considerable losses to the farmer and aid in his economical survival[216].

6.6 Control and eradication programmes

In the light of the high prevalence of paratuberculosis, the significant economic losses caused by the disease and the uneasiness caused by a possible link with Crohn's disease, attempts are being made in several countries to control paratuberculosis. In Australia, a large scale Market Assurance Programme focussed at cattle, sheep as well as goat herds is being set up and in other countries e.g. the Netherlands more experimental trials are being carried out to gain experience for a nationwide programme. The control programmes being developed not only differ from country to country but their design also heavily depends on the species the programmes are focussed on. For example, using faecal culture as a routine diagnostic test is for dairy cattle farmers an option, whereas the same test for the farmer with a goat or sheep flock (much larger number of animals, lower profits per animal and lower value of the animal itself) is not an economic option.

Indeed, for controlling the disease in small ruminants, vaccination, could be the only affordable alternative at present. Field trials using vaccination of small ruminants have been quite successful in removing clinical disease from the herds[217]. Vaccination has been used in small ruminants in Greece, Spain, UK and Iceland.

For control and eradication programmes, designed to be used in dairy cattle herds, to be successful, it is widely accepted that at the same time extensive husbandry measures should be taken in combination with intensive diagnostic testing. Husbandry measures have two objectives: first, to prevent further spread of a possible infection within a herd and second, to prevent introduction of the infection into a free herd. For the first objective, prevention of infection of young, newborn animals is very important. The introduction of clean calving pens, separate housing of young and older animals, feeding colostrum/milk from their own mother only etc. are very important rules. For the second objective it is very important to keep a closed herd. This involves not purchasing any animals from farms with an unknown history with respect to paratuberculosis but also not spreading manure as fertiliser coming from other farms. Methods to disinfect *Map* contaminated slurry by the addition of formalin or calcium cyanymide have also been described[218].

However, the diagnostic tests remain the weak link in any conceivable control programme. All available tests, as discussed above, lack sufficient sensitivity to give any warranty about the paratuberculosis status of an individual animal at a single time point and young infected animals, which are most often traded, can easily escape testing. Nevertheless, available diagnostic tests like the absorbed ELISA or faecal culture, can be used in certifying the paratuberculosis status of a herd, even though the test has limited value for certifying the individual animal. This can be done by testing only the older animals within a herd and when positives are found the herd can be classified as positive. Repetitive testing will give, with each round of testing, more certainty about the true status of the herd.

In a prototype control programme, diagnostic testing is aimed at identifying infected herds and at certifying herds to be free of paratuberculosis. Isolating positive herds

(e.g. ban from trading) will stop further spread of paratuberculosis. Subsequently a test-and-cull programme should be used to eradicate the disease and replacement stock can be bought from certified free herds. In order to certify herds free of paratuberculosis, numerous rounds of testing over a period of at least 7-8 years may be needed.

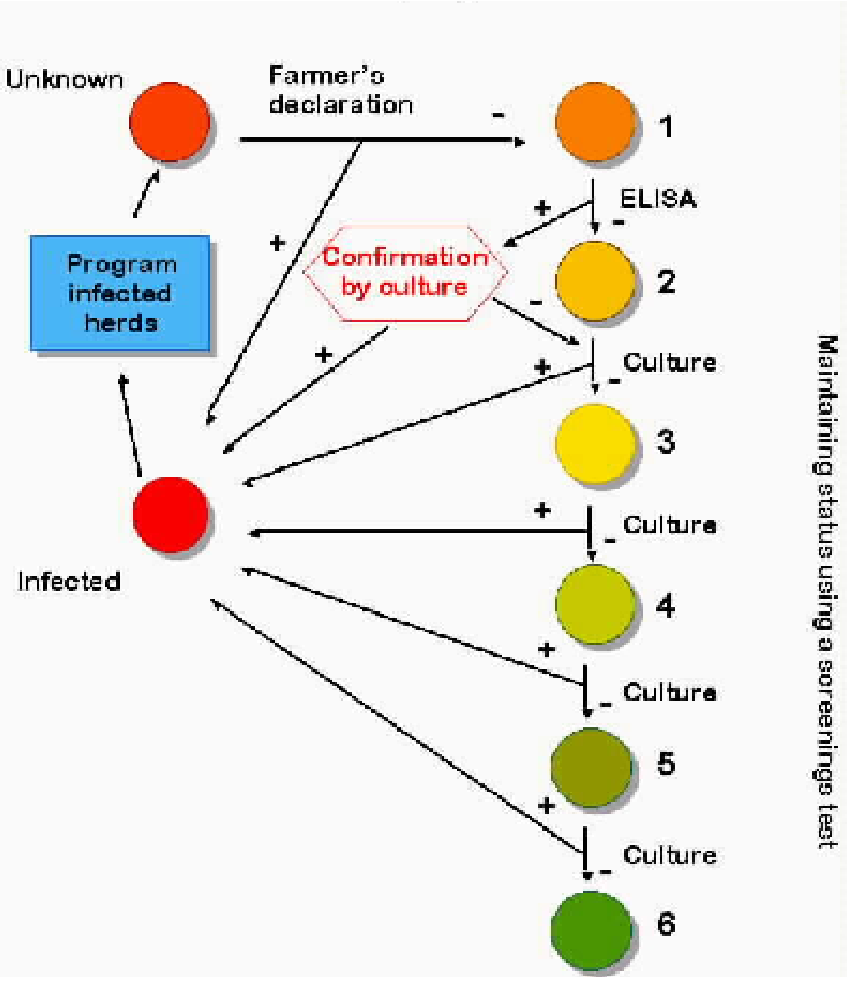

Figure 3: Schematic diagram of a paratuberculosis control programme

Recent experience in the Netherlands[219] with a small scale experimental certification programme showed, however, that even when herds with no known history of paratuberculosis were tested using faecal culture as the diagnostic test, positive farms were still being identified after five rounds of testing at six months intervals. This indicates that, with the test used, positive animals could be missed several times. In fact, fifty percent of the motiviated and enthusiastic farmers, who entered the voluntary programme three years earlier ended up in the 'positive' category. Such farms will have to invest in husbandry changes and expensive diagnostic tests to participate in a test-and-cull programme which will take at least ten years before they will be eligible to enter a new certification programme to obtain free status. Experiences like this explain why there is, at present, less optimism about the

feasability of rapid control programmes for paratuberculosis without the prior development of better diagnostic tests.

7. *M. avium* ssp *paratuberculosis* infection in humans.

7.1 Detection of Map in Crohn's disease by culture and PCR.

Mycobacteria and cell wall deficient organisms were first isolated from tissues from patients with Crohns disease in the 1970's[220]. In the following decade the isolation of *Map* from chronically inflamed intestine of human patients was reported[221]. In this context, culture means the ability eventually to isolate colonies in conventional solid or liquid media with the morphological, phenotypic and biochemical characteristics of very slow growing, mycobactin dependent, bacillary-form *Map*, which could then be subcultured and maintained in the laboratory. Three techniques have been used to detect the organism in patients with Crohn's disease; culture, culture followed by PCR, and PCR applied to resected bowel tissue.

Using culture, *Map* and several other species of mycobacteria, such as *M avium* complex, *M. kansasii*, *M. chelonei* and *M. fortuitum* were isolated by several research workers but only in up to 5% of people with Crohn's disease and often after incubation for many months or years[222],[223],[224],[225],[226],[227] (see Table 6). In many of these reports, spheroplastic forms were obtained, some of which appeared from subsequent studies to be mycobacteria. Innoculation of *Map* isolated from a patient with Crohn's disease has caused granulomas of the distal small intestine in goats following oral administration[228] and hepatic and splenic granulomas in mice[229] following intraperitoneal and intravenous injection.

The application of IS*900* PCR to long term cultures raised the detection rate of *Map* in Crohn's disease gut to 30%[230],[231] (Table 7).

IS*900* PCR applied directly to DNA extracts of full thickness surgically resected gut samples reported the presence of *Map* in about two thirds of people with Crohn's disease in 1992 [232]. This was the first study directly on intestinal tissues, since when there have been eighteen peer reviewed reports (see Tables 8 and 9) of similar studies using a wide variety of sample processing and PCR procedures, in which nine could identify *Map* in Crohn's disease some or most of the time[233],[234],[235],[236],[237],[238],[239],[240],[241], and nine could not[242],[243],[244],[245],[246],[247],[248],[249],[250], though these include more recent work. A recent study from Sweden[251] employed a broad range PCR to amplify 16S rDNA in eubacteria reported *MAC* but also *Helicobacter* spp. *Listeria monocytogenes* and *Escherichia coli* in three of five surgical samples from Crohn's disease patients.

Several reasons have been suggested to account for the conflicting results on the PCR detection of *Map* in Crohn's disease such as the low abundance of the primary specific pathogen. In addition, it is considerably more difficult to isolate chromosomal DNA from mycobacteria than it is from other bacteria in general[252]. It has also been proposed that *Map* in Crohn's disease (or in sheep with the paucibacillary form of paratuberculosis) may not be a conventional spheroplast and may exist as a tough protease-resistant phenotype[253]. However, this has not been tested experimentally. It has also been reported that optimal access to target *Map* DNA requires the inclusion of a mechanical disruption step by vibrating the sample lysate in a slurry of silica and

ceramic particles using the Hybaid Ribolyser system or bead beater[254]. The results of using these more recently optimised methods confirm the presence of *Map* in Crohn's disease tissue. *Map* has also been isolated from a single case of a patient with a non-tuberculous cervical lymphadenitis resistant to anti-TB treatment which preceded the onset of typical Crohn's disease by five years[255].

A system for the detection of *Map* has recently been developed which comprises a ten week incubation of a decontaminated tissue extract in an improved liquid culture medium (MGIT Becton Dickinson) followed by IS*900* PCR on the culture. The results to date have identified *Map* in 6 of 7 full thickness surgical samples of intestinal wall from patients with Crohn's disease[256], and not in normal intestine. Using the same system of MGIT liquid culture followed by IS*900* PCR on the culture, these researchers have also isolated *Map* from the centrifugal pellets (but not the cream fractions) of samples of breast milk obtained from two mothers with Crohn's disease who had recently given birth and not from the breast milk of five healthy women[257]. The isolation of *Map* from the breast milk of women with Crohn's disease suggests that in humans as in animals, *Map* infection can be systemic.

Discrepancies, experimental difficulties and laboratory to laboratory variation have also surrounded the PCR detection of other low abundance bacterial pathogens particularly in the chronically inflamed disease tissues of people with tuberculosis[258], Lyme disease[259], brucellosis[260] and lymphocytic leprosy[261]. It seems clear, however that *Map* is present in the intestines of at least a proportion of Crohn's disease patients but also, at a lower rate, in a proportion of apparently healthy controls.

Table 6. Isolations of Map on culture media

**Culture**					
**Reference**	**Total no examined**	**Crohns Patients**	**Controls**	**Method**	**Comments**
Ref. 222	14	3/14	Not included	Culture for 18 months	Isolations of mycobactin dependent mycobacteria
Ref. 223	30	1/30	Not included	Culture from colonic material for 16 weeks confirmed by biochemical characteristics	
Ref. 224	82	4/82	1/55	Culture from surgically resected tissue<br>1 confirmed as *Map*	4 other mycobacteria isolated
Ref. 225		1	Not included	Culture<br>*Map* confirmed	
Ref. 226	66	1/66	Not included	Culture<br>*Map* confirmed	3 other mycobacteria isolated (fortuitum and 2 unknown)
Ref. 240	86	8/24	5/22 UC<br>1/40 non IBD	Faecal culture	PCR was also performed (see table 8) but both PCR and culture were not performed on all samples
Ref. 227	5	1/5	Not included	Culture from surgically resected tissue for 2.5 months	

Table 7. Detection of Map by PCR on culture media

**PCR on Culture**					
**Reference**	**Total no examined**	**Crohns Patients**	**Controls**	**Method**	**Comments**
Ref. 230	29	6/18	1/11	IS900 PCR	Other mycobacteria were also detected
Ref. 231	30 cultures examined	6 /17 positive for *Map*	0/13	IS900 PCR	Mycobacterial DNA also detected in both CD and control tissues

Table 8. Detection of Map by PCR on tissue samples

**PCR on tissue**		**Map detected**			
**Reference**	**Total no examined**	**Crohns Patients**	**Controls**	**Method**	**Comments**
Ref. 232 above	103	26/40	6/63	PCR IS900 on surgically resected tissues	
Ref. 233	62	11/24	5/38	PCR IS900 on fresh and paraffin embedded surgically resected tissues	
Ref. 234	53	13/18	10/35	PCR IS900 on surgically resected tissues	Some controls were patients with undiagnosed intestinal disease
Ref. 235	61	4/31	0/30	PCR IS900 on surgically resected tissues	
Ref. 236	35	2/9	2/15 UC<br>0/11 non IBD	PCR IS900 on colon biopsy samples	Several biopsies tested from different parts of the colon for each subject. Two (1CD+1UC) were positive from more than one site..
Ref. 237	44	10/10	11/18 UC<br>14/16 controls	PCR IS900	
Ref. 238	61	10/26	4/35	PCR IS900 on paraffin wax embedded tissues	PCR Amplification was repeated – only slight reactions seen after the first amplification (40 cycles)
Ref. 239	74	17/36	2/18 UC<br>3/20 non IBD	PCR IS900	

Table 8 continued. Detection of Map by PCR on tissue samples

**PCR on tissue**		**Map detected**			
**Reference**	**Total no examined**	**Crohns Patients**	**Controls**	**Method**	**Comments**
Ref. 240	75	15/17	9/18 UC<br>1/40 non IBD	IS 900PCR-DEIA	Culture was also performed (see table 6) but both PCR and culture were not performed on all samples
Ref. 241	12	8/8	2/2 UC<br>0/2 colonic cancer	PCR IS900 on ileal mucosa	
Ref. 251	22	3/11	0/11	PCR	Mycobacteria (PCR was not specific for map). The 3 positive were 3 of 5 taken surgically. Others were removed by endoscope

Table 9. Failure to detect Map by PCR on tissue samples

**PCR on tissue**		**Map not detected**			
**Reference**	**Total no examined**	**Crohns Patients**	**Controls**	**Method**	**Comments**
Ref. 242	143	0/68	0/49 UC<br>1/26 non IBD controls	Fluorescent PCR IS900 on intestinal tissue samples	
Ref. 243	72	0/36	0/13<br>0/23	PCR on intestinal biopsies	Mycobacteria detected in approx. 50% of samples from all groups
Ref. 244	38	0/27	0/11	PCR	Numbers refer to colon specimens
Ref. 245	37	0/10	0/27	Multiplex PCR	Biopsy from terminal ileum
Ref. 246	59	0/21	0/14 UC<br>0/24 controls	PCR	Faeces, sera and intestinal tissue samples
Ref. 247	37	1/21	0/5 UC<br>0/11 controls	PCR<br>Culture	Intestinal mucosal biopsies and surgical resections. One PCR positive was obtained. All cultures were negative.
Ref. 248	51 specimens	0/34	0/17	PCR IS900	biopsies and surgical resections including lymph nodes was used
Ref. 249	94	0/47	0/27<br>0/20	PCR various primers	
Ref. 250	40	0/13	0/14 UC<br>0/13 controls	PCR	Samples of intestine

7.2 Immunological responses to Map in humans with Crohn's disease.

Map is very closely related to other MAC and organisms of the *M.intracellulare* complex. Analysis of the 5' region of 16S DNA used for taxonomy shows complete sequence identity between *Map* and *M.intracellulare* serovars 4, 5, 6, 8, 9, 10 and 11 and only a single base pair difference from other *M.avium* sp[262]. MAC are widely distributed in the environment and can be isolated by faecal culture from healthy persons[263,264]. Immunological recognition of MAC antigens is therefore widespread in the general population[265,266].

A serological study in 1980 attempted to identify agglutination of three strains of *Map* by Crohn's disease sera. No response was seen with two of the strains, and the agglutination observed with the third *Map* strain showed no difference between Crohn's disease and normal sera[267]. Between 1984 and 1994 five research groups in the USA, Italy, UK and Argentina used crude extracts of "*M.paratuberculosis* strain 18" in ELISAs to look for differences in antibody binding between Crohn's disease and control sera[268,269,270,271,272]. With one exception[268], no differences were reported. In the context of human infection specifically with *Map*, these studies are of doubtful validity because "*M.paratuberculosis* strain 18" is not *Map* at all, but an *M.avium* sp.[273]. Despite this, three out of the four negative studies were interpreted as providing evidence against a causal relationship between *Map* and Crohn's disease. Three further studies in 1988-1993 used crude extracts of human *Map* strain Linda or a *Map* of animal origin coated on ELISA plates, to look for differences in antibody binding between Crohn's disease and control sera. No differences were found[274,275,276]. A recent study from Japan[277] however, reported a significant increase in IgG binding to a crude protoplasmic extract of *Map* by Crohn's disease sera compared with normal controls ($p<0.05$). Three other research groups have tested for differences in peripheral blood or mucosal cell-mediated immune (CMI) responses to sonicates of *Map*, heat killed *Map*, or PPD preparations of *Map* between Crohn’s disease and control subjects; again no differences were found[278,279,280]. The authors of all these negative serological and CMI studies concluded that their data do not support a mycobacterial aetiology for Crohn's disease.

On the other hand, animal health care workers who may be repeatedly exposed to high dose bacilliary-form *Map* develop immunological responses to these organisms. These responses can be detected using crude antigen extracts of *in vitro* cultured bacillary-form *Map* and are significantly different from those of control subjects who do not have such a high level of exposure[281]. However the crude antigens used would also detect immune responses to many other members of the MAC complex to which these workers were likely to be exposed so these results cannot be regarded as specific for *Map*.

These studies and the various interpretations that have been placed on them suffer from the inherent difficulty that a considerable proportion of the population has been exposed and has immunity to MAC to which *Map* belongs and is very closely related. We might not expect to detect differences in serological or CMI reactivity between

people with Crohn's disease and other people with the crude antigenic preparations used.

More recent work (see Table 10) has focussed on antibody recognition of selected proteins and peptides of *Map*. Eighty four percent of Crohn's disease sera were found to recognise one or more of three proteins of 38-kDa, 24-kDa, and 18-kDa from *Map*[282], though these are known to have homologues in other mycobacteria. Research has demonstrated significant recognition by one third of Crohn's disease sera, of a specific B-cell epitope in the carboxyterminal 13.6-kDa portion of the 34-kDa component of the A36 immunodominant complex of *Map*. This target is also recognised by sera from cattle infected with *Map* [173],[175]. El-Zaatari *et al* identified *Map* proteins p35 (the rediscovered above cited 34-kDa protein) and p36 by screening a genomic expression library of *Map* with rabbit antisera[283],[284]. In one study the p36 antigen was recognised by 86% of Crohn's disease sera and by 11% of patients with UC, non IBD and also healthy (non BCG vaccinated) subjects. However 100% of BCG vaccinated subjects also reacted as did 89% of patients with tuberculosis or leprosy [285]. In a further study using both the p35 and p36 antigens, 74% of sera from patients with Crohn's disease reacted to both antigens whereas no healthy controls and only 1 out of 10 UC patients sera also reacted to both antigens[286].

Table 10. Immunological responses in patients with Crohn’s disease

Study Ref.	Antigen used	Total Subjects	Positive results in CD patients	Results in controls	Comments
Ref. 282	38kD	67	16/28	20 UC<br>18 healthy controls (all BCG vaccinated)	84% of CD patients positive to at least 1 of the 3 antigens<br><br>18% positive to all three<br><br>No correlation between responses to the three antigens
	24kD		15/28	0/20 UC	
	18kD		15/28	2/20 UC	
173<br>175	Polypeptide A362	122	73	50	36% of patients with CD had anti-A362 IgA titres significantly higher than the control group. Distribution in CD group was bimodal
Ref. 285	p36	199	77/89	16/18 with TB or Leprosy<br><br>5/42 UC and non IBD<br><br>10/10 BCG vaccinated controls<br><br>4/40 non BCG vaccinated controls	
Ref. 286	p36	98	40/53	1/10 UC<br>3/35 H	
	p35	166	79/89	4/27 UC<br>5/50 H	
	p35+p36	98	39/53	1/10 UC<br>0/35 H	
Ref. 289	Serum IgA binding to mycobact-erial HupB protein	30	9/10	0/10 UC<br>0/10 healthy	Cut off was mean+2sd of healthy control group
UC=Ulcerative Colitis H= Healthy control IBD Inflammatory Bowel disease					

A peptide epitope in the carboxyterminal 12-kDa portion of p43 encoded by IS*900* has been identified which is recognised by IgG from Crohn's disease sera but also sera from each of the other control groups[287],[288]. Recent work[289] has shown that anti-neutrophil cytoplasmic antibodies (pANCA) are a marker for the immune response in inflammatory bowel disease patients, including a subset of Crohn's disease patients. Histone H1 and the *M. tuberculosis* histone-like protein HupB were identified as a pANCA autoantigen and as a pANCA cross-reacting antigen, respectively.

Homologues of the *M. tuberculosis* HupB protein were also found to react with pANCA Igs in several other mycobacterial species, including *Map*. It was also shown that the sera of 9 out of 10 patients with Crohn's disease contained IgA directed against the *M. tuberculosis* HupB protein ($p<0.001$).

The results of these studies indicate a relatively weak recognition of *Map* in Crohn's disease which is only visible if certain immunological targets are selected. The specificity of antibodies to *Map* is not completely known and the possibility of cross reactions with other members of the MAC must be borne in mind. In addition, people suffering from Crohn's disease have a 'leaky' intestine and readily form antibodies to intestinal bacteria[290] and components of food such as *Saccharomyces cerevisiae* [290],[291],[292],[293]. The design of many, if not all, of the above cited studies fails to adequately take account of this factor and the effect it might have on the immune response of patients with Crohns disease. Thus weak immunological reactivity to specific *Map* components in Crohn's disease could be secondary to 'bystander' mycobacteria colonising the intestine as a result of Crohn's disease induced mucosal disruption, or an expression of the ability of *Map* to evade immune recognition, a strategy widely used by other pathogens[294].

7.3 Treatment of Crohn's disease with antimycobacterial drugs.

If *Map* is involved in the causation of Crohn's disease, it may be that drugs active against *Map* would produce clinical improvement in affected patients and in this sense the results of treatment may provide clues to the causation of the disease. However the results of such trials should be treated with caution for two reasons. Firstly, there is no guarantee that even if the disease were caused by mycobacteria that it would respond to treatment and secondly a clinical response could be caused by a non specific antibacterial or antimycobacterial response and may not be related specifically to *M avium* subsp. *paratuberculosis*. It must also be considered that human drug trials have, in many cases, been conducted on the most resistant cases of the disease i.e. on patients which have failed to respond to more conventional therapy. This section examines the various attempts to treat Crohn's disease with antimycobacterial drugs.

From 1975 to 1989 there were 11 anecdotal reports and open studies of the use of antimycobacterial drugs in the treatment of Crohn's disease. In 1975 Ward and McManus reported a marked clinical improvement in 4 of 6 patients with Crohn's disease treated with dapsone[295]. A more extensive study in 1977 reported that 40 of 52 patients with severe Crohn's disease treated with various combinations of rifampicin, isoniazid, streptomycin and ethambutol, showed clinical improvement, though the disease itself could not be fully cured [296]. A similar improvement was reported in Crohn's disease patients treated with rifampicin[297]. Schultz *et al.* described the complete remission of severe Crohn's disease in a 52 year old man treated with rifampin, isoniazid, pyrazinamide and ethambutol[298] . The same drug combination used in a 60 year old man with co-existing pulmonary tuberculosis and severe Crohn's disease, was followed by the cessation of diarrhoea of up to 6 times a day for the first time for 16 years, and weight gain from 43 to 51.5kgs [299]. Further examples of Crohn's

disease responding to anti-tuberculous drugs were also reported by various researchers[300],[301],[302],[303],[304]. Taken together, these case reports and open studies represent the cumulative experience of this treatment approach in a total of 107 selected Crohn's disease patients from 11 different centres throughout North America and Western Europe.

The message, which is consistent, is that there is a very small sub-group of people with Crohn's disease who do show clinical improvement which is occasionally dramatic, in response to treatment with conventional anti-tuberculous chemotherapy. With few exceptions however, clinical improvement is not lasting and complete cure has not been achieved.

A significant beneficial effect of antimycobacterial drugs to a larger proportion of people with Crohn's disease has not been substantiated in most randomised controlled trials. Shaffer *et al.* [305] found no subsequent difference in Crohn's disease activity index between 14 patients treated with rifampicin and ethambutol and 13 placebo control patients. A study in 28 patients found that clofazimine used as a single agent was ineffective in inducing remission in Crohn's disease[306]. Rutgeerts *et al*, reported that rifabutin and ethambutol did not prevent recurrent Crohn's disease in the neoterminal ileum after surgery for Crohn's disease[307]. In a further study[308], Prantera *et al.* randomised 40 patients with severe refractory steroid-dependent Crohn's disease to receive rifampicin, ethambutol, clofazimine and dapsone, or placebo. Significant improvement in biochemical and haematological parameters in the treatment group compared with controls occurred, together with a relief of symptoms. In a controlled trial of rifampicin, isoniazid and ethambutol versus placebo, Swift *et al.* [309] reported a significant reduction in abdominal pain, improvement in well-being score and reduction of abdominal mass at 2 months in the treated, compared with the control group. This apparent improvement was not however maintained, and no long term advantage in the course of the disease was subsequently seen[310]. These controlled trials involved a cumulative total of 245 patients.

Comparison of the results of treating *Map* infections in animals, and Crohn's disease in humans with antimycobacterial drugs need to be approached with care. Naturally occurring and experimental *Map* infection in animals almost always represents pluribacillary disease with the organisms having established mycobacterial cell walls. It has been theorised that the situation in Crohn's disease would be one in which *Map* would be present in very low abundance (see section 7.1), and with the organisms in a non-bacillary phenotype, so that differences in drug susceptibility between the animal and human disease, might occur. Despite this there are obvious similarities in the outcome of the treatment of *Map* infected animals and of humans with Crohn's disease using antimycobacterial drugs. The impression in both cases is that, whereas on some occasions clinicopathological improvement may follow the use of combinations of multiple agents, remission is unlikely to be sustained and a complete cure will not be achieved. This is consistent with what has long been known, that MAC in general are resistant to standard anti-tuberculous drugs[311],[312],[313]. MAC can prevent these agents from penetrating the myobacterial cell and can rapidly develop mutations which confer drug resistance[314],[315],[316],[317],[318]. MAC infections in immunocompetent hosts are difficult to eradicate; prolonged treatment is required and relapse either on treatment or off treatment is common.

An advance in the treatment of MAC infections in both HIV and non-HIV patients, as well as in the availability of candidate drugs for the treatment of Crohn's disease, came with the development of a new series of therapeutic agents which are chemical modifications of natural streptomyces antibiotics. Of particular relevance was rifabutin (ansamycin), a derivative of rifamycin-S[319,320], the macrolide clarithromycin, a derivative of natural erythromycins and the azalide azithromycin. These agents were found to have markedly improved activity against MAC in vitro[321,322,323,324,325] both alone and in combination with other agents. They also have the particular advantage of being concentrated within macrophages and other cells[326]. Furthermore rifabutin and clarithromycin demonstrated good activity in vitro against *Map*[327,328] and appear to synergise[329]. Early studies of the use of rifabutin in primates (macaques) naturally infected with *MAP* [330], and in 6 patients with Crohn's disease, were promising[331]. A preliminary report of a controlled trial of monotherapy with clarithromycin in 15 Crohn's disease patients, demonstrated sustained remission in the treatment group[332]; however a subsequent study of clarithromycin and ethambutol failed to show any benefit in Crohn's disease[333]. Monotherapy with clarithromycin may be followed by an initial 'honeymoon' response in active Crohn's disease, but it invites the development of drug resistance and should be avoided[334,335,336,337,338]. Both rifabutin and clarithromycin target microbial protein synthesis rather than inhibition of cell wall biosynthesis and were predicted to be applicable to the non-bacillary phenotype of *Map* in Crohn's disease. A two year outcomes analysis of the use of rifabutin together with clarithromycin or azithromycin in the treatment of 46 patients with active Crohn's disease was initiated in 1992. This study[339] demonstrated a highly significant improvement in the disease activity index in patients after 6 months treatment which was maintained at 2 years ($p<0.001$), and a significant improvement in inflammatory parameters. The efficacy of rifabutin and macrolide therapy in active Crohn's disease was further supported by work carried out independently in Australia[340]. A randomised double blind controlled multicenter trial of a combination of rifabutin, clarithromycin and clofazimine versus placebo on a background of standard prednisone immune suppression, was initiated in Australia in September 1999. The results of this study are not expected to be available before the middle of 2002.

8. Exposure of human populations to *M.avium* ssp.*paratuberculosis*

This chapter examines whether it is possible for the human population to be exposed to *Map* and therefore whether the opportunity exists for humans to become infected with the organism. In theory, humans could come in contact with *Map* by direct contact with other humans or animals or by indirect means such as diet.

8.1 Non-dietary exposure

Faeces of animals with the multibacillary form of paratuberculosis contain enormous numbers of *Map*. Humans coming in contact with either clinically or subclinically infected animals are therefore liable to be exposed to large numbers of the bacteria. Occupational groups affected would include farmers, veterinarians and abattoir workers. However a conspicuously high incidence of Crohn's disease has not been reported in these occupational groups though large scale epidemiological studies are required.

The possibility of spread from humans[257] has been mentioned earlier in Chapter 7, though the main possibility could be from faecal contamination. However, while *Map* has been cultured from the faeces of Crohn's disease patients and healthy people, the number of organisms involved appears to be very small. However, the recent report of the detection of *Map* in breast milk of mothers with Crohn's disease could provide a means by new generations could be exposed to *Map*. This finding is based on only two cases so more work is required to determine the extent of the issue. On the other hand, previous epidemiological work has indicated that patients with Crohn's disease were over represented among those who had received little or no breast feeding[341]. Higher levels of Crohns disease have not been reported in the spouses of patients with Crohn's disease

8.2 Dietary exposure.

8.2.1 Raw milk

It has long been known that *Map* can be cultured from raw milk of clinically infected cows with paratuberculosis[342],[343],[344]. It is probable that the organisms are present within the monocyte population abundant in milk, but they also occur extracellularly. More recent work has shown that *Map* can also be cultured from the milk of apparently healthy subclinically infected cows. Sweeney *et al*[345], cultured *Map* from the milk of 19% of healthy cows which were heavy faecal shedders of *Map*, and from the milk of 5% of healthy cows which were intermediate or light shedders of the organism. Streeter *et al* cultured *Map* from the colostrum and milk of 30% of faecal culture positive clinically normal animals[346]. Relying as they do on the ability of the *Map* from these animals to survive decontamination by overnight incubation in 0.75%

hexadecylpyridinium chloride (HPC) and then be culturable, such studies inevitably underestimate the true prevalence of these difficult-to-culture organisms.

Given the high prevalence of *Map* in the dairy herds and domestic livestock of western Europe and North America, it is inevitable that *Map* will from time to time be present in bulked tank milk being brought to pasteurisation plants. This raw milk is normally subjected to treatment either by pasteurisation, UHT treatment or sterilisation. The proportion of milk consumed in each of these categories varies considerably throughout Europe.

8.2.2 Heat treated milk

Because of the difficulty in culturing the organism, it has proved problematic to experimentally determine the efficiency of treatments such as pasteurisation [347],[348]. Since 1993 seven studies have shown that *Map* prepared in *in vitro* cultures, spiked into whole cows' milk at a range of microbial concentrations and then treated with experimental pasteurisation, remained culturable from some samples, after exposure to 65°C for 30 minutes (the Standard Holder method) or 72°C for 15 seconds (the High Temperature Short Time method) [349],[350],[351],[352],[353],[354],[355]. These studies have been criticised principally on the grounds that experimental pasteurisation does not accurately reproduce the conditions such as turbulent flow which occur in commercial pasteurisation units[356]. In addition, because of the presence of the organism in monocytes, spiking studies may not be representative. Two other studies reported the complete loss of culturability of *Map* spiked into milk and heated to 72°C for 15 seconds in either a laboratory scale pasteuriser unit, or in capillary tubes submerged in a circulating water bath[357],[358] though the procedures used have also been criticised[356].

An extensive survey carried out in England and Wales in 1990-1994 found that an overall 7% of cartons and bottles of retail whole pasteurised cows' milk tested positive for *Map* by IS*900* PCR[359]. The test detection limit was estimated to be about 200 *Map* ml^{-1}. Liquid cultures inoculated with *Map*-positive samples of cream or pellet, subsequently demonstrated the microscopic presence of sparse clumps of acid fast organisms when examined in the time frame 4 to 12 weeks of incubation. These cultures in multiple flasks were themselves strongly positive by IS*900* PCR and suggested the presence of residual *Map*. These cultures invariably went on to become overgrown by other organisms, and proof of live *Map* by subculture onto solid media was not obtained.

Subsequent research using raw milk spiked at 10^6 cfu ml^{-1}, has confirmed the ability of these organisms to survive pasteurisation conditions at 72°C for 15 seconds, as well as demonstrating a considerable range in the heat tolerance of different strains of *Map* right up to residual culturability after 90°C for 15 seconds. However, none of the strains investigated remained culturable after exposure to 72°C for 25 seconds, suggesting that extension of the holding time is more likely to achieve complete inactivation of *Map* in milk[360]. Ongoing work in the same laboratory, using improved sample processing procedures such as immunomagnetic capture of *Map* [361] and optimised decontamination prior to culture and IS*900* PCR, is currently demonstrating

Map in a small percentage of samples of retail pasteurised cows' milk. A recent interim report of ongoing work in the UK has reported the finding of viable *Map* in around 5% of raw milk samples examined and in about 3% of pasteurised samples examined[362], although no information is yet available on the numbers of organisms present.

8.2.3 Other foods

Because of its presence in raw milk, *Map* may be initially present in cheeses made from raw milk from infected animals, or in those made from milk exposed to pasteurisation at lower temperature prior to the cheese-making process. MAC are generally resistant to acid conditions and are known to be able to resist the acidic conditions created in intracellular phagolysosomes as part of their strategy for survival within the host cell. MAC may thus survive low pH conditions involved in cheese-making, although the recovery of MAC from cheese, by growth in conventional culture, may be unreliable. At the present time there are no publications from laboratory based research specifically addressing the question of whether *Map* is present in cheese, but epidemiological studies for environmental risk factors for MAC in patients with HIV disease, identified an increased risk associated with the consumption of hard cheeses[363].

Macrophages containing *Map* are known to be found throughout the body of animals with the advanced pluribacillary form of Johne's disease. Most tissues including lymph nodes, spleen, bone marrow, liver, kidney and lung are affected but titres of bacteria are low.

8.2.4 Water supplies.

Other *M.avium* complex (MAC) organisms, widely distributed in the environment and in natural waters, act as a source of nontuberculous mycobacterial disease in humans where infection is acquired by environmental exposure[364],[365],[366]. Drinking water is known to act as a source of *M. avium* superinfections in humans with AIDS and primates with SIV[50],[367],[368]. Both subclinically and clinically infected livestock shed abundant quantities of *Map* onto pastures. Unlike *M.bovis*, *Map* can survive in the environment for prolonged periods[369],[370],[371]. An unquantified further contribution to the environmental contamination by *Map* will be made by wildlife hosts such as infected deer, rabbits, foxes, stoats, and weasels[62],[372].

The possibility of Map entering lakes or rivers where considerable dilution would take place will depend on local topography and soil types. There is a general need to increase the volume and intensity of environmental research into *Map* within Europe.

9. Summary

This report has confined itself to an examination of the possibility that Crohn's disease is caused by *Mycobacterium avium* subsp. *paratuberculosis.* It has not examined other possible causes of the disease in detail.

1. *Mycobacterium avium* subsp. *paratuberculosis* (*Map*) is an organism which can cause chronic inflammation of the intestine (Johne's disease) in cattle, sheep and goats as well as in other animals including rabbits and macaques.

2. *Map* infections are widespread in domestic livestock, including cattle, sheep and goats. Rabbits, deer and other animals have been identified as wildlife hosts of *Map*.

3. Animals can harbour *Map* without showing signs of disease or reacting to serological tests.

4. *Map* is found in large quantities in the intestine of diseased animals and in lesser amounts in lymph nodes, liver, spleen and other tissues.

5. Clinically infected animals may shed up to 10^{12} *Map* per ml in their faeces. Subclinically infected animals also shed the organism though usually in lower amounts. Infected dairy cows and sheep shed *Map* in their milk.

6. *Map* can survive for prolonged periods (up to 9 months) on contaminated pastures and in the environment.

7. *Map* infections in animals are usually acquired at an early age from faeces contaminated environments and from milk, younger animals being more susceptible than adults.

8. Paratuberculosis in animals presents a range of histopathological types from multibacillary to lymphocytic/paucimicrobial forms.

9. *Map* is a very slow growing organism. Laboratory culture is a time consuming and insensitive means of detecting low levels of contamination by *Map* in environmental and food samples. In animals, faecal culture is regarded as the gold standard for diagnosis but it is slow and dependent on the organism being present in the faeces.

10. Immunological assays for *Map* infection in animals generally have poor sensitivity or poor specificity and can be unreliable, particularly for the early detection of infection.

11. Genetic analyses have revealed different strains of *Map*.

12. *Map* contains 14-18 copies of a specific DNA insertion element (IS900) which can be used to detect the presence of *Map* DNA. These PCR tests can be very sensitive when optimal sample processing procedures and reaction conditions are used.

13. *Map* is more robust than *M. tuberculosis* and *M. bovis*. The complete destruction of all viable *Map* in milk by pasteurisation at 65°C for 30 minutes or 72°C for 15 seconds, may not be assured. Viable *Map* has been identified in pasteurised milk supplies.

14. There is preliminary evidence that *Map* may be present in human milk. If these initial results are confirmed, this would be a significant finding.

15. Crohn's disease is a chronic inflammation of the intestine in humans which presents some clinical and pathological similarities to the lymphocytic/paucimicrobial form of paratuberculosis in animals.

16. Crohn's disease occurs in Western European countries with an overall incidence of 5.6 per 100,000 individuals per year.

17 Insufficient data are available to compare the incidence and prevalence of Crohn’s disease and paratuberculosis so as to determine epidemiological links between both diseases.

18. *Map* has been detected in approximately 30% of pathological samples from patients with Crohn's disease by IS900 PCR tests and also, at a lower level, in healthy people. *Map* can only be grown in conventional culture in about 5% of cases and then only after months or years of incubation.

19. The results of IS900 PCR tests on DNA extracts of Crohn's disease intestine carried out in many different laboratories have been conflicting. Several studies which succeeded in detecting *Map* in patients with Crohn’s disease also detected the organism in a smaller percentage of healthy subjects. Other studies were unable to detect the organism.

20. Crohn’s disease patients display an increased serological response to many intestinal micro-organisms including *Map*. This may simply reflect an increased immunological response in patients to many micro-organisms perhaps as a consequence of a ‘leaky intestine’.

21. A few Crohn's disease patients show clinical remission when treated with anti-tuberculosis drugs, but relapse usually occurs. New drugs such as rifabutin and macrolide antibiotics active against *M. avium* sp., are reported to be effective in higher numbers of Crohn's disease patients in open studies, though relapse still occurs. Results of large-scale well designed controlled therapeutic trials are not yet available.

10. Research

10.1 Crohn's disease

Further research is required to resolve this important issue raised in the request from the Commission. This research should be targeted on the following aspects;

1. Large scale epidemiological studies of Crohn's disease patients to examine risk factors, particularly in early life.

2. Attempts to localise the presence and pathology of *M avium* subsp. *paratuberculosis* in the human gut. The significance of the finding of spheroplasts in this disease needs to be determined

3. Follow up work to fully evaluate recent findings such as the finding of *M avium* subsp. *paratuberculosis* in breast milk and immunological recognition of mycobacterial and *Map* components in man.

4. Large scale multi-centre double blind drug trials using combination therapy of those drugs liable to be active against *Map*, preferably on patients in whom *M paratuberculosis* has been detected.

5. Experiments should be carried out both *in vitro* and *in vivo* to determine possible methods of transmission.

Facilitating the creation and maintenance of a network of researchers at EU level combining expertise in chronic inflammatory bowel diseases in humans and in *mycobacterium* sp. infections in animals, would greatly contribute to attaining these goals.

10.2 Paratuberculosis

Aside from any possible link with Crohn's disease, the development of the necessary tools to eradicate paratuberculosis from animals should also be a priority.

1. Improvement diagnostic methods are required and their development should be encouraged. There is a need to validate the methodology of currently used diagnostic methods, particularly sample preparation methods. Comparative testing between various laboratories should be carried out

2. The development of efficient vaccines and diagnostic tests to distinguish infected and vaccinated animals.

3. All these activities would be greatly accelerated by the availability of the complete genomic sequence of *Map*.

4. The distribution and levels of *Map* in infected animals should be determined.

5. Experiments with various time temperature combinations for pasteurisation should be carried out to determine optimum combinations to inactivate the *Map* bacterium.

6. The survival of *Map* in the environment and the role of natural water and water supplies in the transmission of paratuberculosis needs to be determined.

7. Statistically robust studies on the prevalence of Map in domestic and wild animals should be carried out. These will be of assistance for future eradication programmes and will also allow comparisons to be carried out with the geographical prevalence of Crohns disease.

11. Conclusions

The currently available evidence is insufficient to confirm or disprove that *Mycobacterium paratuberculosis* is a causative agent of at least some cases of Crohn's disease in man.

There are sufficient grounds for concern to warrant increased and urgent research activity to resolve the issue. This research is described in section 10.1 of this report.

Crohn's Disease is most likely a multifactorial condition. Its incidence is more common in the western world, in families where there have been other cases and in homes where hygiene in early life has been good. There are considerable clinical and pathological similarities, though also some significant differences between paratuberculosis in animals and Crohn's Disease in man.

The organism *Mycobacterium avium* subsp *paratuberculosis* (*Map*) is relatively common and it is likely that many people have come into contact with it. The detection of *Map* in a greater proportion of Crohn's disease patients than in controls suggests that the organism may have a role either as a causative agent, as a secondary invader which exacerbates the disease or as a non pathogenic coloniser because of changed bowel conditions.

Exposure to high levels of the organism can arise from direct contact with infected farm animals or by drinking raw milk from infected animals. Exposure to lower levels of the organism could possibly arise from heat treated milk or from sources such as wildlife hosts. A simple relationship between exposure to high (or low) levels of organisms and the development of Crohn's disease does not appear to exist.

If *Map* is involved in the causation of Crohn's disease, it would also require the presence of other susceptibility factors. It is also possible that its involvement may relate to a sub set of Crohn's disease cases.

The current tools available to control paratuberculosis in animals are inadequate. Using current methods, eradication programmes are likely to be long lasting, expensive and with low probability of success, particularly in endemic areas. Because paratuberculosis is an economically significant disease, the development of tools, such as efficient diagnostic tests, to enable eradication would be extremely beneficial and would encourage eradication programmes throughout the world.

Results from recent drug trials in humans with drugs likely to be active against *Map* have been encouraging but inconclusive. Larger scale double blind studies are in progress to obtain better data.

12. References

[1] Clarke, C.J. (1997). The pathology and pathogenesis of paratuberculosis in ruminants and other species. *J. Comp.Path*., 116, 217-261

[2] Cocito C; Gilot P; Coene M; de Kesel M; Poupart P; Vannuffel P (1994) Paratuberculosis. *Clin. Microbiol. Rev*.;7(3):328-45

[3] Benedictus G; Dijkhuizen AA; Stelwagen J.(1987) Economic losses due to paratuberculosis in dairy cattle. *Vet. Rec.;* 121(7):142-6

[4] Ott SL; Wells SJ; Wagner BA. (1999) Herd-level economic losses associated with Johne's disease on US dairy operations. *Prev. Vet. Med*.,11;40(3-4):179-92

[5] Juste, R.A. (1997). Johne’s disease: A review of current knowledge. IV International Congress for Sheep Veterinarians. pp. 140-150. 2-6 February, 1997. Armidale, NS Wales, Australia

[6] Merkal, R. S. ; Larsen, A. B. and Booth, G. D. (1975). Analysis of the effect of inapparent bovine paratuberculosis. *Am. J. Vet. Res.* 36: 837-838.

[7] Shivananda S; Lennard-Jones J; Logan R; Fear N; Price A, Carpenter L; van Blankenstein M. (1996) Incidence of Inflammatory bowel disease across Europe: is there a difference between north and south? Results of the European collaborative study on inflammatory bowel disease (EC-IBD). *Gut*, 39:690-697

[8] Ekbom A. Cost to Society in Crohn’s disease. Proceedings of the clinical symposium on IBD in Prague 1997; ASTRA-Lund-Sweden

[9] Prior P; Gyde S; Cooke WT; Waterhouse JA; Allan RN. (1981) Mortality in Crohn's disease. *Gastroenterology;* 80(2):307-12

[10] Silverstein MD; Loftus EV; Sandborn WJ; Tremaine WJ; Feagan BG; Nietert PJ; Harmsen WS; Zinsmeister AR (1999) Clinical course and costs of care for Crohn's disease: Markov model analysis of a population-based cohort. *Gastroenterology;* 117(1):49-57

[11] Hay JW; Hay AR (1992) Inflammatory bowel disease: costs-of-illness. *J Clin Gastroenterology;*14(4):309-17

[12] Thorel M-F; Krichevsky M; Levy-Frebault VV. (1990) Numerical Taxonomy of Mycobactin-Dependent Mycobacteria, emended description of *Mycobacterium avium*, and description of *Mycobacterium avium* subsp.*avium* subsp.nov., *Mycobacterium avium* subsp.*paratuberculosis* subsp.nov., and *Mycobacterium avium* subsp.*silvaticum* subsp.nov. *Int. J. System. Bacteriol*. 1990; 40:254-260.

[13] Kunze ZM, Wall S; Appelberg R; Silva MT; Portaels F; McFadden JJ. (1991) IS*901*, a new member of a widespread class of atypical insertion sequences, is associated with pathogenicity in *Mycobacterium avium. Mol. Microbiol;* 5:2265-2272.

[14] Moss MT; Malik ZP; Tizard ML; Green EP; Sanderson JD; Hermon-Taylor J (1992) IS*902*, an insertion element of the chronic-enteritis-causing *Mycobacterium avium* subsp *silvaticum. J. Gen. Microbiol.;*138: 139-145.

[15] Collins P; Matthews PRJ; McDiarmid; A Brown A. (1983) The pathogenicity of *Mycobacterium avium* and related mycobacteria for experimental animals. *J. Med. Micro*.; 16:27-35.

[16] Matthews, P. R; McDiarmid, A (1979). The production in bovine calves of a disease resembling paratuberculosis with a *Mycobacterium sp* isolated from a wood-pigeon (*Columba palumbus*). *Vet. Rec.* 104: 286.

[17] Nyange, (1991). Studies on paratuberculosis on red deer. PhD Thesis. University of Edinburgh.

[18] Thorel M.F.; Pardon P.; Irgens K.; Marly J.; Lechopier P.(1984) Paratuberculosis expérimentale : pouvoir pathogène chez le veau de souches de Mycobactéries mycobactine-dépendantes. *Ann. Rech. Vet.*,15:365-374.

[19] Ritacco V; Kremer K; van der Laan T; Pijnenburg JE; de Haas PE; van Soolingen D. (1998) Use of IS*901* and IS*1245* in RFLP typing of *Mycobacterium avium* complex: relatedness among serovar reference strains, human and animal isolates. *Int. J. Tuberc Lung Dis*; 2: 242-251

[20] Hernandez Perez M; Fomukong NG; Hellyer T; Brown IN; Dale JW. (1994) Characterization of IS*1110*, a highly mobile genetic element from *Mycobacterium avium. Mol. Microbiol*.; 12: 717-724

[21] Collins DM; Cavaignac S; de Lisle GW. (1997) Use of four DNA insertion sequences to characterise strains of the *Mycobacterium avium* complex isolated from animals. *Mol. Cell Probes*; 11: 373-380.

[22] Puyang X; Lee K; Pawlichuk C; Kunimoto D. (1999) IS*1626*, a new IS*900*-related *Mycobacterium avium* insertion sequence. *Microbiol.*;145:3163-3168.
[23] Roiz MP; Palenque E; Guerrero C; Garcia MJ. (1995). Use of restriction fragment length polymorphism as a genetic marker for typing *Mycobacterium avium* strains. *J. Clin. Microbiol.*; 33:1389-1391
[24] Picardeau M; Varnerot A; Lecompte T; Brel F; May T; Vincent V. (1997) Use of different molecular typing techniques for bacteriological follow-up in a clinical trial with AIDS patients with *Mycobacterium avium* bacteremia. *J. Clin. Microbiol.* 1997; 35:2503-2510.
[25] Belisle JT; Klaczkiewicz K; Brennan PJ; Jacobs WR Jr; Inamine JM (1993) Rough morphological variants of *Mycobacterium avium*. Characterisation of genomic deletions resulting in the loss of glycopeptidolipid expression. *J. Biol. Chem.* 1993;268: 10517-10523.
[26] Taylor, A. W. (1951):.Varieties of *Mycobacterium Johnei* isolated from sheep. *J. Path. Bacteriol.*, 63: 333-336
[27] Juste, RA; Marco, JC; Saez de Ocariz; C, Aduriz, JJ (1991): Comparison of different media for the isolation of small ruminant strains of Mycobacterium paratuberculosis. *Vet. Microbiol.* 28: 385-390
[28] Green, E.P.; Tizard, M.L.Y.; Moss, M.T.; Thompson, J.; Winterbourne, D.J.; McFadden, J.J.; Hermon-Taylor, J. (1989). Sequence and characteristics of IS900, an insertion element identified in a human Crohn's disease isolate of *Mycobacterium paratuberculosis. Nucleic Acid Res.* 17, 9063-9073.
[29] Stevenson K; Sharp JM, (1997) The contribution of molecular biology to *Mycobacterium avium* subspecies *Paratuberculosis* research *Vet. J;* 153(3):269-86
[30] Rogall T; Flohr, T; Bottger, E.C. (1990). Differentiation of *Mycobacterium* species by direct sequencing of amplified DNA. *J. Gen. Microbiol.* 136, 1915-1920.
[31] Stahl, D.A; Urbance, J.W. (1990). The division between fast- and slow-growing species corresponds to natural relationships among the mycobacteria. *J. Bacteriol.* 172, 116-1241990
[32] Van der Giessen, J.W.B.; Haring, R.; Van der Zeijst, B.A.M. (1994). Comparison of the 23S ribosomal RNA genes and the spacer region between the 16S and 23S rRNA genes of the closely related *Mycobacterium avium* and *Mycobacterium paratuberculosis* and the fast-growing *Mycobacterium phlei*. *Microbiology* 140, 1103-1108.
[33] Wards B.J.; Collins, D.M.; De Lisle, G.W. (1987). Restriction endonuclease analysis of members of the *Mycobacterium avium-M.intracellulare-M.scrofulaceum* serocomplex. *J. Clin. Microbiol.* 25, 2309-2313.
[34] Whipple, D.L.; Le Febvre, R.B.; Andrews, R.E. Jr.; Thiermann, A.B. (1987). Isolation and analysis of restriction endonuclease digestive patterns of chromosomal DNA from *Mycobacterium paratuberculosis* and other *Mycobacterium* species. *J. Clin. Microbiol.* 25, 1511-1515.
[35] Levy-Frebault, V.V.; Thorel, M-F.; Varnerot, A.; Gicquel, B. (1989). DNA polymorphism in *Mycobacterium paratuberculosis*, "wood pigeon mycobacteria," and related mycobacteria analysed by field inversion gel electrophoresis. *J. Clin. Microbiol.* **27**, 2823-2826.
[36] Bono, T.; Jemmi, T.; Bernascconi, C.; Burki, D.; Telenti, A.; Bodmer, T. (1995). Genotypic characterisation of *mycobacterium avium* strains recovered from animals and their comparison to human strains. *Appl. Environ. Microbiol.* **61**, 371-373.
[37] Guerrero, C.; Bernasconi, C.; Burki, D.; Bodmer, T.; Telenti, A. (1995). A novel insertion element from *Mycobacterium avium*, IS1245, is a specific target for analysis of strain relatedness. *J. Clin. Microbiol* **33**, 304-307
[38] Collins, D.M.; Gabric, D.M.; de Lisle, G.W. (1990). Identification of two groups of *Mycobacterium paratuberculosis* strains by restriction endonuclease analysis and DNA hybridisation. *J. Clin. Microbiol.* **28**, 1591-1596.
[39] Whipple, D.; Kapke, P.; Vary, C. (1990). Identification of Restriction Fragment Length Polymorphisms in DNA from *Mycobacterium paratuberculosis. J. Clin. Microbiol.* **28**, 2561-2564
[40] de Lisle, G.W.; Collins, D. M.; Huchzermeyer H.F.A.K. (1992) Characterisation of ovine strains of *Mycobacterium paratuberculosis* by restriction endonuclease analysis and DNA hybridisation. *Onderstepoort J. Vet. Res.* **59**, 163-165
[41] de Lisle, G.W.; Yates, G.F.; Collins, D.M. (1993). Paratuberculosis in farmed deer: case reports and DNA characterisation of isolates of *Mycobacterium paratuberculosis. Journal of Veterinary Diagnostic Investigations* **5**, 567-571
[42] Thoresen O.V.; Olsaker, I. (1994). Distribution and hybridisation patterns of the insertion element IS900 in clinical isolates of *Mycobacterium paratuberculosis. Vet. Microbiol.* **40**, 293-303.

[43] Pavlik, I.; Bejckova, L.; Pavlas, M.; Rozsypalova, Z.; Koskova, S. (1995). Characterisation by restriction endonuclease analysis and DNA hybridisation using IS900 of bovine, ovine, caprine and human dependent strains of *Mycobacterium paratuberculosis* isolated in various localities. *Vet. Microbiol.* **45**, 311-318

[44] Pavlik I; Horvathova A; Dvorska L; Bartl J; Svastova P; du Maine R; Rychlik I. (1999). Standardisation of restriction lenght polymorphism analysis for Mycobacterium avium subspecies paratuberculosis. *J. Microbiol Meth*. 38, 155-167

[45] Whittington R; Marsh I; Choy E; Cousins D (1998) Polymorphisms in IS1311, an insertion sequence common to *Mycobacterium avium* and *M. avium* subsp. *paratuberculosis*, can be used to distinguish between and within these species. *Mol. Cell. Probes*, 12, 349-358

[46] Marsh I; Whittington R; Cousins D. (1999) PCR-restriction endonuclease analysis for identification and strain typing of Mycobacterium avium subsp. paratuberculosis and Mycobacterium avium subsp. avium based on polymorphisms in IS1311. *Mol. Cell. Probes*, 13(2):115-26

[47] Arbeit, R.D.; Slutsky, A.; Barber, T.W.; Maslow JN; Niemczyk S; Falkinham JO; O'Connor GT; von Reyn CF. (1993). Genetic diversity among strains of *Mycobacterium avium* causing monoclonal and polyclonal bacteremia in patients with AIDS. *J. Infect. Dis.* **167**, 1384-1390

[48] Masurek, G.H.; Hartman, S.; Zhang, Y.; Brown, B.A.; Hector, J.S.R.; Murphy, D.; Wallace, R.J.Jr. (1993). Large DNA restriction fragment polymorphism in the *Mycobacterium avium - M. intracellulare* complex: a potential epidemiologic tool. *J. Clin. Microbiol.* **31**, 390-394.

[49] Slutsky, A.M.; Arbeit, R.D.; Barber, T.W.; Rich J; von Reyn CF; Pieciak W; Barlow MA; Maslow JN. (1994). Polyclonal infections due to *Mycobacterium avium* complex in patients with AIDS detected by pulsed-field gel electrophoresis of sequential clinical isolates. *J. Clin. Microbiol.* **32**, 1773-1778.

[50] von Reyn, C.F.; Maslow, J.N.; Barber, T.W.; Falkingham III, J.O.; Arbeit, R.D. (1994). Persistent colonisation of potable water as a source of *Mycobacterium avium* infection in AIDS. *Lancet* 343, 1137-1141

[51] Coffin, J.W.; Condon, C.; Compston, C.A.;Potter KN; Lamontagne LR; Shafiq J; Kunimoto DY. (1992). Use of restriction fragment length polymorphisms resolved by pulse-field gel electrophoresis for subspecies identification of mycobacteria in the *Mycobacterium avium* complex and for isolation of DNA probes. *J. Clin. Microbiol.* 30, 1829-1836.

[52] Feizabadi MM; Robertson ID; Hope A; Cousins DV; Hampson DJ (1997) Differentiation of Australian isolates of *Mycobacterium paratuberculosis* using pulsed-field gel electrophoresis. *Aust Vet J.*; 75(12):887-9

[53] Hughes, personal communication

[54] Bull, T.; Pavlik, I.; Hermon-Taylor, J.H.T.; El Zaatari, F.; and Tizard M.; Study of IS900 loci in Mycobacterium avium subsp. paratuberculosis by multiplex PCR screening In Proceedings of the Sixth International Colloquium on Paratuberculosis, Melbourne , Australia; Eds. Manning E.J.B. and Collins M.T.

[55] Lee, T-Y; Lee, T-J.; Kim, S-K. (1994). Differentiation of *Mycobacterium tuberculosis* strains by arbitrarily primed polymerase chain reaction-based DNA fingerprinting. *Yonsei Medical Journal* 35, 286-294.

[56] Linton , C.J.; Jalal, H.; Leeming, J.P.; Millar, M.R. (1994). Rapid discrimination of *Mycobacterium tuberculosis* by random amplified polymorphic DNA analysis. *J. Clin. Microbiol.* 32, 2169-2174.

[57] Matsiota-Bernard, P.; Waser, S.; Tassios, P.T.; Kyriakopulos, A.; Legakis, N.J. (1997). Rapid discrimination of *Mycobacterium avium* strains from AIDS patients by randomly amplified polymorphic DNA analysis. *J. Clin. Microbiol.* 35, 1585-1588

[58] Scheibl, P.; Gerlach, G-F. (1997). Differentiation of *Mycobacterium paratuberculosis* isolates by rDNA-spacer analysis and random amplified polymorphic DNA patterns. *Vet. Microbiol.* 51, 151-158.

[59] Francois B; Krishnamoorthy R; Elion J. (1997) Comparative study of *Mycobacterium paratuberculosis* strains isolated from Crohn's disease using restriction fragment length polymorphism and arbitrarily primed poplymerase chain reaction. *Epidemiol. Infect.*, 118, 227-23

[60] Johne, H.A. and Frothingham, L. (1895): Disch. Zeitschr. Tiermed. Vergl. Path, 21:438-454.

[61] Greig, A.; Stevenson; K. Pérez, V. Pirie AA; Grant JM; Sharp JM. (1997). Paratuberculosis in wild rabbits (Oryctolagus cuniculus) . *Vet. Rec.*, 140: 141-143. (1997).

[62] Beard PM; Henderson D; Daniels M; Pirie A; Buxton D; Greig A; Hutchings MR; McKendrick I; Rhind S; Stevenson K; Sharp M. (1999) Evidence for paratuberculosis in fox (*vulpes vulpes*) and stoat (*mustela erminea*). *Vet. Rec.*, 145, 612-613

[63] Chiodini, R. J.; Van Kruiningen, H. J. Merkal, R. S. (1984). Ruminant paratuberculosis (Johne's disease): the current status and future prospects. *Cornell Vet.* 74: 218-262.

[64] Jorgensen JB. (1977) Survival of Mycobacterium paratuberculosis in slurry. *Nord Vet Med.* 29(6):267-70

[65] Larsen, A. B.; Kopecky, K. E. (1970). *Mycobacterium paratuberculosis* in reproductive organs and semen of bulls. *Am. J. Vet. Res.* 31:255-258.

[66] Rankin, J. D. (1961) The experimental infection of cattle with *Mycobacterium johnei*. 3. Calves maintained in an infectious environment. *J. Comp. Pathol.Ther.* 71:10-15.

[67] Gilmour, N. J. L.; Angus, K. W. (1991): Paratuberculosis. In: Diseases of sheep. 2nd edition, Ed W.B. Martin and I.D. Aitken, Blackwell Scientific Publications.pp 95-99.

[68] Sweeney RW (1996) Transmission of paratuberculosis. *Vet Clin North Am Food Anim Pract.*; 12(2):305-12

[69] Momotani, E.; Whipple, D.L.; Thiermann, A.B. Cheville NF. (1988): Role of M cells and macrophages in the entrance of Mycobacterium paratuberculosis into domes of ileal Peyer's patches in calves. *Vet. Path.,* 25: 131-137.

[70] Landsverk, T.; Halleraker, M.; Aleksandersen, M.; McClure, S.; Hein, W.; Nicander, L. (1991): The intestinal habitat for organized lymphoid tissues in ruminants: comparative aspects of structure, function and development. *Vet. Immunol. Immunopathol.*, **28**: 1-16.

[71]. Nisbet, D. J.; Gilmour, N. J. L.; Brotherston, J. G (1962): Quantintative studies of *Mycobaterium johnei* in tissues of sheep. III.- Intestinal histopathology. *J. Comp. Path.*, **72**: 80-91

[72] Juste, R. A.; García Marín, J. F.; Peris, B.; Saez de Ocariz CS; Badiola JJ. (1994): Experimental infection of vaccinated and non-vaccinated lambs with Mycobacterium paratuberculosis. *J. Com. Path.* 110: 185-194

[73] Perez , V; Chavez, G.; Gutierrez, M., Tellechea, J.; Badiola, J. J., Garcia Marin, J. F. (1994): Evaluation of the response to AGID and gamma-interferon tests in lambs infected with *Mycobacterium avium* subsp *silvaticum* and *Mycobacterium avium* subsp. *paratuberculosis* and their relation with the diagnosis of ovine paratuberculosis. In: Chiodini, R. J.; Collins, M. T.; Bassey, E. O. E. (Eds.). Proceedings of the Fourth International Colloquium on Paratuberculosis. Ed. International Association for paratuberculosis. Providence. EE. UU. pp. 91-96

[74] Ridley, D. S. (1974). Histological classification and the immunological spectrum of leprosy. *Bull. W.H.O.*, 51: 451-465.

[75] Pérez, V.; Tellechea, J.; Badiola, J. J.; Gutierrez M; Garcia Marin JF. (1997). Relation between serologic response and pathologic findings in sheep with naturally acquired paratuberculosis. *Am. J. Vet. Res.* 58: 799-803.

[76] Perez, V., Garcia Marin, J.F. and Badiola, J.J. (1996). Description and classification of different types of lesion associated with natural paratuberculosis in sheep. *J. Comp. Path.*, 114, 107-122

[77] Clarke, C.; Little, D. (1996). The pathology of ovine paratuberculosis: gross and histological changes in the intestine and other tissues. *J. Comp. Path.*, 114: 419-437.

[78] Buergelt CD; Hall C; McEntee K; Duncan JR (1978) Pathological evaluation of paratuberculosis in naturally infected cattle. *Vet Pathol.;*15(2):196-207

[79] Corpa, J. M.; Garrido, J.; Garcia Marin, J. F.; Perez, V. (2000). Classification of lesions associated with natural paratuberculosis in goats. *J. Comp. Path.*, (In press)

[80] Burrells, C., Clarke, C.J., Colston, A., Kay, J.M., Porter, J., Little, D., Sharp, J.M. (1998) A study of immunological responses of sheep clinically-affected with paratuberculosis (Johne's disease). The relationship of blood, mesenteric lymph node and intestinal lymphocyte responses to gross and microscopic pathology. *Vet. Immunol Immunopathol.* 66(3-4):343-58.

[81] Gilmour N.J.L. (1976) The pathogenesis, diagnosis and control of Johne's disease. *Vet. Rec.*, 99, 433-434

[82] Patterson DS; Allen WM; Berrett S; Ivins LN; Sweasey D, (1968) Some biochemical aspects of clinical Johne's disease in cattle. *Res Vet Sci*, 9, 117

[83] Jones DG; Kay JM. (1996), Serum biochemistry and the diagnosis of Johne's disease (paratuberculosis) in sheep. *Vet Rec*,139, 498-499

[84] Irvine EJ. (1996) Quality of life in inflammatory bowel disease and other chronic diseases. Scand J *Gastroenterology*; 31 suppl 221: 990-995
[85] Shivananda S; van Blankenstein M; Schouten WR et al. (1993) Quality of life in Crohn's disease: results of a case-control study. Euro J *Gastroenterol &Hepatol*; 5: 919-925
[86] Gent AE; Hellier MD; Grace RH; Swarbrick ET; Coggon D. (1994) Inflammatory bowel disease and domestic hygiene in infancy. *Lancet*; 343: 766-767
[87] Duggan AE; Usmani I; Neal KR; Logan RFA. (1998) Appendicectomy, childhood hygiene, *Helicobacter pylori* status, and risk of inflammatory bowel disease: a case control study. *Gut*; 43: 494-498
[88] Shivananda S; Hordijk ML; Pena AS; Mayberry JF (1989). Crohn's disease: risk of recurrence and reoperation in a defined population. *Gut;* 30: 990-995
[89] Crohn BB; Ginzburg I; Oppenheimer GD. (1932) Regional ileitis: a pathologic and clinical entity. *JAMA*, 99:1323-1328
[90] Dalziel TK. Chronic intestinal enteritis. *B. M. J.* 1913,2:1068-1070
[91] Lockhart-Mummery HE; Morson BC. (1960) Crohn's disease (regional enteritis) of the large intestine and its distinction from ulcerative colitis. *Gut*; 1:87-105
[92] Thompson D.E., (1994) The Role of Mycobacteria in Crohn's Disease. J. Med Microbiol. 41, 71-74.
[93] Chief Medical Officer, (1998) Department of Health UK. Measles, MMR Vaccine, Crohn's disease and Autism. PL/CMO/98/2. 27th March 1998
[94] World Health Organisation concludes that measles viruses are not associated with Crohn's disease. *Commun Dis Rep CDR Wkly* 1998 Feb27;8(9):75,78
[95] Sartor RB, (1997) Pathogenesis and immune mechanisms of chronic inflammatory bowel diseases. *Am J Gastroenterol;* 92(12 Suppl):5S-11S
[96] Duchmann R; Schmitt E; Knolle P; Meyer zum Buschenfelde KH; Neurath M. (1996) Tolerance towards resident intestinal flora in mice is abrogated in experimental colitis and restored by treatment with interleukin-10 or antibodies to interleukin-12. *Clin. Exp. Immunol.;* 104: 483-490
[97] Cosnes J; Carbonnel F; Beugerie L; Le Quintrec Y; Gendre JP. (1996) Effect of cigarette smoking on the long-term course of Crohn's disease. *Gastroenterology*; 110: 424-431
[98] Sutherland JR; Ramcharan S; Bryant H; Fick G. (1990). Effect of cigarette smoking on recurrence of Crohn's disease. *Gastroenterology*; 98: 1123-1128
[99] Peeters M; Nevens H; Baert F; Hiele M; de Meyer AM, Vlietinck R; Rutgeerts P (1996). Familian aggregation in Crohn's disease: increased age-adjusted risk and concordance in clinical characteristics. *Gastroenterology*, 111:597-603
[100] Colombel JF; Grandbastein B; Gower-Rousseau C; Plegat S; Evrard JP; Dupas JL; Gendre JP; Modigliani R; Belaiche J; Hostein J; Hugot JP; van Kruiningen H; Cortot A. (1996) Clinical characteristics of Crohn's disease in 72 families. *Gastroenterology*, 111:604-607
[101] Bayless TM; Tokayer AZ; Polito JM 2nd; Quaskey SA; Mellits ED; Harris ML. (1996) Crohn's disease: concordance for site and clinical type in affected family members -potential heredity influences. *Gastroenterology* , 111:573-579
[102] Polito JM; Rees RC; Childs B; Mendeloff AI; Harris ML; Bayless TM. (1996) Preliminary evidence for genetic anticipation in Crohn's disease. *Lancet*, 347:798-800
[103] Satsangi J; Grootscholten C; Holt H; Jewell DP. (1996) Clinical pattern of familial inflammatory bowel disease. *Gut*, 38:738-741
[104] Satsangi J; Jewell DP; Rosenberg WMC, Bell JI. (1994) Genetics of Inflammatory Bowel Disease: A progress report. *Gut*; 35: 696-700
[105] Satsangi J; Jewell DP; Bell JI. (1997) Genetics of Crohn's disease. *Gut*; 41 (suppl 3) A35 (22.02)
[106] Hugot JP; Laurent-Puig P; Gower-Rousseau C; Olson JM; Lee JC; Beaugerie L; Naom I; Dupas JL; Van Gossum A, Orholm M; Bonaiti-Pellie C; Weissenbach J; Mathew CG; Lennard-Jones JE; Cortot A; Colombel JF; Thomas G. (1996) Mapping of a susceptibility locus for Crohn's disease on chromosome 16. *Nature*, 379:821-823
[107] Reinshagen M; Loelinger C; Kuehnl P; Weiss U; Manfras BJ; Adler G; Boehm BO. (1996) HLA class II genes with susceptibility to Crohn's disease. *Gut* 1996, 38:538-542
[108] Satsangi J; Welsh KI; Bunce M; Julier C; Farrant JM; Bell JI; Jewell DP. (1996) Contribution of genes of the major histocompatibility complex to susceptibility and disease phenotype in inflammatory bowel disease. *Lancet* 1996, 347:1212-1217

[109] Danze PM; Colombel JF; Jacquot S; Loste MN; Heresbach D; Ategbo S; Khamassi S; Perichon B; Semana G; Charron D; Cezard JP. (1996) Association of HLA class II genes with susceptibility to Crohn’s disease. *Gut*, 39:69-72

[110] Parkes M; Satsangi J; Jewell DP. (1997) Inflammatory bowel disease genetics: narrowing the region of linkage on chromosome 12. *Gut*, 41 (suppl 3) A35

[111] Mirza M, Lee J, Teare D; Hugot JP et al. The inflammatory bowel disease (IBD1) locus on chromosome 16: suggestive evidence of linkage to ulcerative colitis. *Gut* 1997, (suppl 3) A35

[112] Berrebi D; Besnard M; Fromont-Hankard G; Paris R; Mougenot JF; De Lagausie P; Emilie D; Cezard JP; Navarro J; Peuchmaur M. (1998) Interleukin-12 expression is focally enhanced in the gastric mucosa of pediatric patients with Crohn's disease. *Am J Pathol*;152(3):667-72

[113] MacDermott RP; Sanderson IR; Reinecker HC (1998) The central role of chemokines (chemotactic cytokines) in the immunopathogenesis of ulcerative colitis and Crohn's disease. *Inflamm. Bowel Dis.*; 4(1):54-67

[114] Lugering N; Kucharzik T; Stoll R; Domschke W. (1998) Current concept of the role of monocytes/macrophages in inflammatory bowel disease--balance of proinflammatory and immunosuppressive mediators. *Ital. J. Gastroenterol. Hepatol.;* 30(3):338-44

[115] Rogler G; Andus T. (1998) Cytokines in inflammatory bowel disease. World J Surg; 22(4):382-9

[116] Louis E; Belaiche J; van Kemseke C; Franchimont D; de Groote D; Gueenen V; Mary JY. (1997) A high serum concentration of interleukin-6 is predictive of relapse in quiescent Crohn's disease. *Eur. J. Gastroenterol. Hepatol.*; 9(10):939-44

[117] Narula SK; Cutler D; Grint P (1998) Immunomodulation of Crohn's disease by interleukin-10. *Agents Actions* Suppl;49:57-65

[118] Duchmann R; Marker-Hermann E; Meyer zum Buschenfelde KH. (1996) Bacteria-specific T-cell clones are selective in their reactivity towards different enterobacteria or *H. pylori* and increased in inflammatory bowel disease. *Scand J Immunol.;* 44(1):71-9

[119] Herfarth HH; Mohanty SP; Rath HC; Tonkonogy S; Sartor RB. (1996) Interleukin 10 suppresses experimental chronic, granulomatous inflammation induced by bacterial cell wall polymers. *Gut*; 39(96):836-45

[120] Plevy SE; Landers CJ; Prehn J; Carramanzana NM; Deem RL; Shealy D; Targan SR (1997) A role for TNF-alpha and mucosal T helper-1 cytokines in the pathogenesis of Crohn's disease. *J Immunol* 15;159(12):6276-82

[121] van Dullemen HM; Wolbink GJ; Wever PC; van der Poll T; Hack CE; Tytgat GN; van Deventer SJ. (1998) Reduction of circulating secretory phospholipase A2 levels by anti- tumor necrosis factor chimeric monoclonal antibody in patients with severe Crohn's disease. Relation between tumor necrosis factor and secretory phospholipase A2 in healthy humans and in active Crohn's disease. *Scand J Gastroenterol*; 33(10):1094-8

[122] van Deventer SJ; Camoglio L (1997) Monoclonal antibody therapy of inflammatory bowel disease. *Pharm World Sci.*;19(2):55-9

[123] Strober W; Ludviksson BR; Fuss IJ (1998) The pathogenesis of mucosal inflammation in murine models of inflammatory bowel disease and Crohn disease. *Ann Intern Med.* 128(10):848-56

[124] Greenstein RJ; Greenstein AJ. (1995) Is there clinical, epidemiological and molecular evidence for two forms of Crohn's disease? *Mol. Med. Today*, 1(7):343-8

[125] Lennard-Jones JE; Shivananda S. (1997) Clinical Uniformity of inflammatory bowel disease at presentation and during the first year of disease in the north and south of Europe. *Euro J Gastroenterol & Hepatol;* 9:353-359

[126] Hirata I; Berrebi G; Austin LL; Keren DF; Dobbins WO (1986) Immunohistological characterization of intraepithelial and lamina propria lymphocytes in control ileum and colon and in inflammatory bowel disease. *Dig Dis Sci.* 31(6):593-60

[127] Hume DA; Allan W; Hogan PG; Doe WF. (1987) Immunohistochemical characterisation of macrophages in human liver and gastrointestinal tract: expression of CD4, HLA-DR, OKM1, and the mature macrophage marker 25F9 in normal and diseased tissue. *J. Leukoc. Biol.;* 42(5):474-84

[128] The Merck Manual, 14th edition 1999, Merck Research laboratories, New Jersey, USA.

[129] Kátic, I. (1977) in: Bibliography of literature on Johne’s disease (paratuberculosis) 1895-1975. Arsskrift 1977: 110-154

[130] Doyle, T. M. (1956). Johne’s disease. *Vet. Rec.* 68: 869-896.

[131] Taylor, A. W. (1949). Observations on the incidence of infection with *M. johnei* in cattle *Vet. Rec.* 61: 539-540.
[132] Rankin, J. D. (1954). The presence of *Mycobacterium johnei* in apparently healthy cattle. *Vet. Rec.* 66: 550-551.
[133] Jorgensen, J. B. (1972). Studies on the occurrence of paratuberculosis in cattle in Denmark (in Danish) *Nord. Vet. Med.* 24: 297-308.
[134] Vannuffel P; Gilot P; Limbourg B; Naerhuyzen B; Dieterich C; (1994) Coene M; Machtelinckx L; Cocito C Development of species-specific enzyme-linked immunosorbent assay for diagnosis of Johne's disease in cattle. *J Clin Microbiol*; 32(5):1211-6
[135] Merkal RS; Whipple DL; Sacks JM; Snyder GR (1987) Prevalence of *Mycobacterium paratuberculosis* in ileocecal lymph nodes of cattle culled in the United States. *J. Am. Vet. Med. Assoc.*; 190(6):676-80
[136] Juste, R.A.; Garrido, J.; Aduriz, G. (1999). La paratuberculosis desde Neiker. IV Reunion sobre Paratuberculosis en España. Derio. Spain. October 26-27, 1999.
[137] Boelaert F.; Walravens K.; Vermeersch J.P.; Raskin A.; Dufey J.; J. Godfroid. Sample survey for estimating the herd and individual animal seroprevalence for bovine paratuberculosis in Belgium (submitted. Vet microbiol)
[138] Muskens J., H.W. Barkema, E. Russchen, K. van Maanen, Y.H. Schukken, D. Bakker (2000) Prevalence and regional distribution of bovine paratuberculosis in dairy herds in the Netherlands. *Vet. Microbiol.* In press
[139] Dimarelli, Z.; Sarris, K.; Xenos, G. et al.. (1992). *The Paratuberculosis Newsletter*. 3: 8-9.
[140] Juste, R. A., Badiola, J. J.; Arnal, M. C. et al. (1991). *The Paratuberculosis Newsletter*, 3: 3-4
[141] Huchzermeyer, H. F. and Bastianello, S. S. (1991). Proc. III Int. Coll. Ptbc. pp. 140-146.
[142] Juste Jordán, R.A.(1990) "III Reunión sobre paratuberculosis en España". *Informes Técnicos*, **34**. Dpto. de Agricultura y Pesca. Gobierno Vasco.
[143] Jayanthi V, Probert CSJ, Wicks AC, Mayberry JF. (1992) Epidemiology of Crohn's disease in Indian migrants and the indigenous population in Leicestershire. *Q. J. Med*, 298: 125-138
[144] Shivananda S; Lennard-Jones JE; Logan RF; Fear N; Price A; Carpenter L; Van Blankenstein M; and the EC-IBD Study Group.(1996) Incidence of Inflammatory bowel disease across Europe: is there a difference between north and south? Results of the European collaborative study on inflammatory bowel disease (EC-IBD). *Gut*, 39:690-697
[145] Gower-Rousseau C.; Grandbastien B.; Colombel J-F.; A Cortot. (1997) Incidences des maladies inflammatoires chroniques de l'intestin, en France: le tableau s'enrichit. *Gastroenterol Clin. Biol.*, 21 481-482.
[146] Colombel JF; Dupas JL; Cortot A; Salomez JL; Marti R; Gower-Rousseau C; Capron-Chivrac D; Lerebours E; Czernichow B; Paris JC. (1990) Incidence des maladies inflammatoires du tube digestif dans la région Nord-Pas de Calais et le département de la somme (1988). *Gastroenterol. Clin. Biol*, **14**, 614-8.
[147] Gower-Rousseau C; Salomez JL; Dupas JL; Marti R; Nuttens MC; Votte A; Lemahieu M; Lemaire B; Colombel JF; Cortot A. (1994) Incidence of inflammatory bowel disease in northern France (1988 - 1990). *Gut*, **35**, 1433-8.
[148] Flamenbaum M; Zénut M; Aublet-Cuvelier B; Larpent JL; Fabre P; Groupe EPIMICI; Abergel A; Dapoigny M; Bommelaer G. (1997) Incidence des maladies inflammatoires du tube digestif dans le département du Puy-de-Dôme en 1993 et 1994. *Gastroenterol Clin Biol*, 21, 491-6.
[149] Pagenault M; Tron I; Alexandre JL; Cruchant E; Dabadie A; Chaperon J; Rosbaszkiewics M; Bretagne JF et ABERMAD. (1997) Incidence des maladies inflammatoires du tube digestif en Bretagne (1994-1995). *Gastroenterol. Clin. Bio.*, **21**, 483-90.
[150] Bernstein CN; Blanchard JF; Rawsthorne P; Wajda A. (1999) Epidemiology of Crohn's disease and ulcerative colitis in a central Canadian province: A population based study. *Am. J. Epidemiol;* 149: 916-924
[151] Bernstein CN; Blanchard JF. (1999) The epidemiology of Crohn's disease: *Gastroenterol*; 116: 1503-1506
[152] Pinchbeck BR; Kirdeikis J; Thomson ABR. (1988) Inflammatory bowel disease in Northern Alberta: an epidemiologic study. *J. Clin. Gastroenterol.*; 10: 505-515

[153] Loftus EV; Silverstein MD;, Sandhorn WJ; Tremaine WJ; Harmsen WS; Zinsmeister AR. (1998) Crohn's disease in Olmsted County, Minnesota, 1940-1993: Incidence, Prevalence, and Survival. *Gastroenterology*; 114: 1161-1168

[154] Fellows IW; Freeman JG; Holmes GKT. (1990) Crohn's disease in the city of Derby, 1951-85. Gut, 31:1262-1265

[155] Lee FI; Nguyen-van-tam JS. (1994) Prospective study of incidence of Crohn's disease in northwest England: no increase since the late 1970s. *Euro. J. Gastroenterol Hepatol.*, 6:27-31

[156] Ekbom A; Helmick C; Zack M; Adami HO. (1991) The epidemiology of inflammatory bowel disease : a large population-based study in Sweden. *Gastroenterology*, 100:350-358

[157] Thomas GAO; Millar-Jones D; Rhodes J; Roberts GM; Williams GT; Mayberry JF. (1995) Incidence of Crohn's disease in Cardiff over 60 years: 1986-90 an update. *Euro. J. Gastroenterol. Hepatol.*, 7:401-405

[158] Munkholm P. (1997) Crohn's disease-occurrence, course and prognosis. An epidemiologic cohort study. Legeforeningens forlag Kobenhaven 1997, Ph.D thesis

[159] Trallori G; d'Albasio G; Palli D; Bardazzi G; Cipriani F; Frittelli G; Russo A; Vannozzi G; Morettini A. (1991) Epidemiology of inflammatory bowel disease over a 10 year period in Florence (1978-87) *Ital. J. Gastroenterol.* 1991, 23:559-63

[160] Ridge-SE; Morgan-IR; Sockett-DC; Collins-MT; Condron-RJ; Skilbeck-NW; Webber-JJ; (1991) Comparison of the Johne's absorbed EIA and the complement-fixation test for the diagnosis of Johne's disease in cattle : *Aust. Vet. J.;* 68(8): 253-7

[161] Sockett-DC;Heisey- DM; Collins-MT: (1995) Estimating prevalence from the results of a screening test when the sensitivity is a function of the prevalence In Chiodini-RJ;Collins-MT; Bassey-EOE (eds.): Proceedings of the Fourth International Colloquium on Paratuberculosis, 3-8.

[162] Sweeney-RW; Whitlock-RH; Buckley-CL; Spencer-PA ; (1995) Evaluation of a commercial enzyme-linked immunosorbent assay for the diagnosis of paratuberculosis in dairy cattle. *J. Vet. Diagn. Invest.*; 7(4): 488-93

[163] Collins-MT; http:// www.vetmed.wisc.edu/pbs/johnes/ 1998

[164] Whipple-DL; Callihan-DR; Jarnagin-JL: (1991) Cultivation of *Mycobacterium paratuberculosis* from bovine fecal specimens and a suggested standardized procedure. *J. Vet. Diagn. Invest.*; 3(4): 368-73

[165] Vary-PH; Andersen-PR; Green-E; Hermon-Taylor-J; McFadden-JJ (1990) Use of highly specific DNA probes and the polymerase chain reaction to detect *Mycobacterium paratuberculosis* in Johne's disease. *J. Clin. Microbiol.* 1990 May; 28(5): 933-7

[166] Collins-MT; Kenefick-KB; Sockett-DC; Lambrecht-RS; McDonald-J; Jorgensen-JB: (1990) Enhanced radiometric detection of *Mycobacterium paratuberculosis* by using filter-concentrated bovine fecal specimens. *J. Clin. Microbiol.;* 28(11): 2514-9

[167] Moss-MT; Green-EP; Tizard-ML; Malik-ZP; Hermon-Taylor-J. (1991) Specific detection of Mycobacterium paratuberculosis by DNA hybridisation with a fragment of the insertion element IS900. *Gut.*; 32(4): 395-8

[168] Garrido, J.M.; Cortabarria, N.; Aduriz, G.; Juste, R.A. Use of a PCR method on fecal samples for diagnosis of sheep paratuberculosis. Sixth International Colloquium on Paratuberculosis. Melbourne. Australia. 14-18th February, 1999.

[169] Gwozdz-JM; Reichel-MP; Murray-A; Manktelow-W; West-DM; Thompson-KG: (1997) Detection of *Mycobacterium avium* subsp. *paratuberculosis* in ovine tissues and blood by the polymerase chain reaction. *Vet. Microbiol.;* 57(2-3): 233-44

[170] Cousins D.; Whittington R.; Masters A.; Marsh I.; Evans R.; Kluver P. (1999) Investigation of false positives in the IS900 PCR for identification of *Mycobacterium avium* subsp. *paratuberculosis*. In: Proceedings of the Sixth International Colloquium on Paratuberculosis, Melbourne , Australia; Pages 259-264. Eds. Manning E.J.B. and Collins M.T.

[171] Sockett-DC; Conrad-TA; Thomas-CB; Collins-MT.L (1992) Evaluation of four serological tests for bovine paratuberculosis. *J. Clin. Microbiol.*; 30(5): 1134-9

[172] Yokomizo-Y; Kishima-M; Mori-Y; Nishimori-K; (1991) Evaluation of enzyme-linked immunosorbent assay in comparison with complement fixation test for the diagnosis of subclinical paratuberculosis in cattle. *J. Vet. Med. Sci.;* 53(4): 577-84

[173] Gilot P; De Kesel M; Machtelinckx L; Coene M; Cocito C (1993) Isolation and sequencing of the gene coding for an antigenic 34- kilodalton protein of *Mycobacterium paratuberculosis. J Bacteriol*; 175(15):4930-5

[174] De Kesel M; Gilot P; Misonne MC; Coene M; Cocito C. (1993) Cloning and expression of portions of the 34-kilodalton-protein gene of *Mycobacterium paratuberculosis*: its application to serological analysis of Johne's disease. *J. Clin. Microbiol*; 31(4):947-54

[175] Vannuffel P; Dieterich C; Naerhuyzen B; Gilot P; Coene M; Fiasse R; Cocito C (1994a) Occurrence, in Crohn's disease, of antibodies directed against a species-specific recombinant polypeptide of *Mycobacterium paratuberculosis. Clin. Diagn. Lab. Immunol.;*1(2):241-3

[176] Collins-MT; Sockett-DC; Ridge-S; Cox-JC: (1991) Evaluation of a commercial enzyme-linked immunosorbent assay for Johne's disease. *J. Clin. Microbiol*.; 29(2): 272-6

[177] Shermann D.M.; Markham R.J.F.; Bates F. (1984) Agar gel immuno-diffusion test for the detection of clinical paratuberculosis in cattle. *J.Am.Vet. Med. Assoc.* 185, 179-182.

[178] Garcia Marin, JF; Chavez, G.; Aduriz JJ.; Perez, V.; Juste RA.; Badiola JJ: (1993) Prevalence of paratuberculosis in infected goat flocks and comparison of different methods of diagnosis. Proceedings of the 3rd International Colloquium on Paratuberculosis. Orlando. Florida. pp. 157-167.

[179] Sherman, DM; Gezon HM. (1980) Comparison of agar gel immunodiffusion and fecal culture for identification of goats with paratuberculosis. *J. Am. Vet. Med. Assoc.* 177:1208-1211

[180] Sherman DM; Markham, JF; Bates, F. (1984) Agar gel immunodiffsion test for diagnosis of clinical paratuberculosis in cattle. *J. Am. Vet. Med. Assoc.* 185: 179-182 .

[181] Shulaw, WP; Bech-Nielsen S; Rings DM. (1993) Serodiagnosis of paratuberculosis in sheep by use of agar gel immunodiffusion. *Am. J. Vet. Res.* 54: 13-19.

[182] Wood-PR; Corner-LA; Rothel-JS (1991): Field comparison of the interferon –gamma assay and the intradermal tuberculin test for the diagnosis of bovine tuberculosis. *Aust. Vet. J.*; 68: 286-290.

[183] Gilot P.; Cocito C. (1993) Comparative analysis of three sensitins used in cutaneous testing for tuberculosis and paratuberculosis in cattle. *FEMS Microbiol Lett ;* 110: 307-312.

[184] Billman-Jacobe-H; Carrigan-M; Cockram-F; Corner-LA; Gill-IJ; Hill-JF; Jessep-T; Milner-AR; Wood-PR: (1992) A comparison of the interferon gamma assay with the absorbed ELISA for the diagnosis of Johne's disease in cattle.: *Aust. Vet. J.* 1992 Feb; 69(2): 25-8

[185] Larsen AB; Vardaman TH; Groth AH. (1950) Preliminary studies on the effect of streptomycin and other agents on *Mycobacterium paratuberculosis. Am. J. Vet. Res.;*11:374-377.

[186] Larsen AB; Vardaman TH. (1953) The effect of isonicotinic acid hydrazide on *Mycobacterium paratuberculosis. J. Am. Vet. Med. Assoc.* ;122:309-10.

[187] Rankin JD. (1953) Isoniazid: its effect on *Mycobacterium Johnei* in vitro and its failure to cure clinical Johne's disease in cattle. *Vet. Rec*.; 65:640-651.

[188] Rankin JD. (1955) An attempt to prevent the establishment of *Mycobacterium johnei* in calves by means of isoniazid alone and in combination with streptomycin. *Vet. Rec*. ; 67:1105-1107.

[189] Gilmour NJL. (1970) The failure of the rimino phenazine B663 (G30320) to reduce the level of experimental *Mycobacterium johnei* infection in calves. *Br. Vet. J.*;126:5-6.

[190] Gilmour NJL; Angus KW. (1971) Effect of the rimino phenazine B663 (G30320) on *Mycobacterium johnei* infection and reinfection in sheep. *J. Comp. Path*.;81:221-226

[191] Merkal RS; Larsen AB. (1973) Clofazimine treatment of cows naturally infected with *Mycobacterium paratuberculosis. Am. J. Vet. Res.*; 34:27-28.

[192] Baldwin EW. (1976) Isoniazid therapy in two cases of Johne's disease. *Vet. Med. Small Anim. Clin*.;71:1359-62

[193] Slocombe RF. (1982) Combined streptomycin-isoniazid-rifampin therapy in the treatment of Johne's disease in a goat. *Can. Vet. J.*; 23:160-163

[194] Gezon HM; Bither HD; Gibbs HC; Acker EJ; Hanson LA; Thompson JK; Jorgenson RD. (1988) Identification and control of paratuberculosis in a large goat herd. *Am J Vet Res*; 49:1817-1823.

[195] Hoffsis GF; Streeter RN; Rings DM; St.Jean G. (1990) Therapy for Johne's disease. *The Bovine Practitioner*; 25:55-58

[196] Belloli A; Arrigoni N; Belletti GL; Proverbio D; Greppi G; Vacirca G. (1991) First results of paratuberculosis therapy in calves intravenously infected with *Mycobacterium paratuberculosis.* In. R J Chiodini & JM Kreeger eds. Proceedings of the Third International Colloquium on Paratuberculosis. Publ. The Internation Assn. for Paratuberculosis RI. USA. pp144-146.

[197] Das SK, Sinha RP, Chauhan HVS. (1992) Chemotherapy of paratuberculosis in goats: streptomycin, rifampicin and levamisole versus streptomycin, rifampicin and dapsone. *Indian J. Anim. Sci.;* 62;8-13.
[198] Mondal D; Sinha RP; Gupta MK. (1994) Effect of combination therapy in *Mycobacterium paratuberculosis* infected rabbits. *Indian J. Exp. Biol.;* 32:318-323.
[199] St.Jean G. (1996) Treatment of clinical paratuberculosis in cattle. *Vet. Clin. of N.Am.: Food Animal Practice*;12:417-430.
[200] Vallee, H.; Rinjard, P.(1926), Etudes sur l'enterite paratuberculeuse des bovides (note preliminaire) *Rev. Gen. Med. Vet.* 35; 1-9.
[201] Sigurdsson, B. (1960): A killed vaccine against paratuberculosis (Johne´s disease) in sheep. *Am. J. Vet. Res.* **21**: 54-67.
[202] Perez, V.; Garcia Marin, J. F.; Bru, R.; Moreno, B.; Badiola, J. J. (1995): Resultados obtenidos en la vacunación de ovinos adultos frente a paratuberculosis. *Med. Vet.*, **12**: 196-201.
[203] Garcia Marin, J. F.; Tellechea, J.; Gutierrez, M.; Perez, V.; Juste, R. A. (1997): Paratuberculosis vaccination in sheep modifies and limits the development of lesions. In: Chiodini, R. J.; Hines, M. E.; Collins, M. T. (Eds.). Proceedings of the Fifth International Colloquium on Paratuberculosis. Ed. International Association for Paratuberculosis. pp. 223.
[204] Manual of standards for diagnostic tests and vaccines, O.I.E. Paris, 1996.
[205] Marly, J.; Thorel, M. F.; Perrin, G.; Pardon, P.; Guerrault, D. (1988): Suivi de vaccination de chevrettes contre le paratuberculose_ consequences cliniques, serologiques et allergiques et epreuve virulente. In: Thorel, M. F.; Merkal, R. S., (Eds.). Proceedings of the Second International Colloquium on Paratuberculosis. Ed. International Association for Paratuberculosis. pp. 99-109.
[206] Doyle T.M. (1960) Vaccination against Johne's disease. *Brit. Vet. J.* 116: 294-301.
[207] Crowther, R. W.; Polydorou, K.; Nitti, S.; Phyrilla, A. (1976): Johne´s disease in sheep in Cyprus. *Vet. Rec.* **98**: 463.
[208] Corpa, J. M.; Perez, V.; Sanchez, M. A. ; Garcia Marin, J. F. (1999). Control of paratuberculosis (Johne´s disease) in goats by vaccination of adult animals. *Vet. Rec.* (In press).
[209] Wentink G.H.; Bongers J.H.; Zeeuwen A.A.P.A.; Jaartsveld F.H.J. (1994) Incidence of paratuberculosis after vaccination against *M.paratuberculosis* in two infected dairy herds. *J. Vet. Med. B.* 41: 517-522.
[210] Kormendy B. (1994) The effect of vaccination on the prevalence of paratuberculosis in large dairy herds. *Vet Microbiol.* 41: 117-125.
[211] Spangler E.; Heider L.E.; Bech-Nielsen S.; Dorn C.R. (1991) Serologic ELISA responses of calves vaccinated with a killed *M.paratuberculosis* vaccine. *Am. J. Vet. Res.* 52:1197-1200.
[212] Van Weering H.J.; Breukink H.; van der Giessen J.; Haagsma J.; Kalis C.H.J.; de Lange D., Snoep J.J., de Vries G., and Wentink G.H. (1995) Intermediate report on the vaccination of Johne's disease in the Netherlands. *The Paratuberculosis Newsletter* 7: 4-8.
[213] Stuart P. (1965) Vaccination against Johne's disease in cattle exposed to experimental infection. *Brit. Vet. J.* 121:289-318.
[214] Ingliss J.S.S.; Weipers M. (1963) The effect of Johne's vaccination on the efficiency of the single intradermal comparative tuberculin test. *Brit. Vet. J.* 119: 426-429.
[215] Herbert, C.N.; Doyle, T.M.; Paterson, A.B. (1959) Tuberculin sensitivity in attested cattle vaccinated against Johne's disease. *Vet. Rec.* 71: 108-111.
[216] Dijkhuizen A.A.; Van Schaik G.; Huirne R.B.M.; Kalis C.H.J.; Benedictus G. (1994) A cost benefit analysis of vaccination against paratuberculosis in dairy cattle. *The Kenya Veterinarian* 18: 219-221
[217] Garcia Marin J.F.; Tellechea J.; Gutierrez M.; Corpa J.M.; Perez V. (2000) Evaluation of two vaccines (killed and attenuated) against small ruminant paratuberculosis. In: Proceedings of the Sixth International Colloquium on Paratuberculosis, Melbourne , Australia; Pages 234-241. Eds. Manning E.J.B. and Collins M.T.
[218] Bohm R; Ley T. (1994) Disinfection of Mycobacterium paratuberculosis in cattle slurry. Proc. 8th. Internat. Congress on Animal Hygiene, St. Paul, Minnesota, 12-16 Sept. 1994 pp10-16.
[219] Kalis C.H.J.; Barkema H.W.; Hesselink J.W. (2000) Herd certification for paratuberculosis in unsuspected dairy herds using cultures of strategically pooled faecal samples. In: Proceedings of the Sixth International Colloquium on paratuberculosis , Melbourne, Australia, (in press).

[220] Burnham WR; Lennard-Jones JE; Stanford JL; Bird RG (1978) Mycobacteria as a possible cause of inflammatory bowel disease. *Lancet*; 2 (8092 Pt 1):693-6

[221] Chiodini RJ. (1989) Crohn's disease and the mycobacterioses: a review and comparison of two disease entities. *Clin Microbiol;* 2:90-117.

[222] Chiodini RJ; Van Kruiningen HJ; Merkal RS; Thayer WR; Coutu JA. (1984) Characteristics of an unclassified *Mycobacterium* species isolated from patients with Crohn's disease. *J Clin Microbiol*; 20:966-971.

[223] Coloe P; Wilkes CR; Lightfoot D; Tosolini FA. (1986) Isolation of *Mycobacterium paratuberculosis* in Crohn's disease. *Aust Microbiol;* 7:188A.

[224] Gitnick G; Collins J; Beaman B; Brooks D; Arthur M; Imaeda T; Palieschesky M. (1989) Preliminary report on isolation of mycobacteria from patients with Crohn's disease. *Dig Dis Sci*; 34:925-932.

[225] Thorel M-F. (1989) Relationship between *Mycobacterium avium, M .paratuberculosis* and mycobacteria associated with Crohn's disease. *Ann Rech Vet;* 20:417-429.

[226] Haagsma J; Mulder CJJ; Eger A; Tytgat GNJ. (1991) *Mycobacterium paratuberculosis* isole chez des patients atteints de maladie de Crohn. Resultats preliminaires. *Acta endosc*; 21:255-260.

[227] Pavlik I; Bejckova L; Koskova S; Fixa B; Komarkova O; Bedrna J. (1994) DNA fingerprinting as a tool for epidemiological studies of paratuberculosis in ruminants and Crohn's disease. In: R J Chiodini, MR Collins & E. Bassey (eds), Proceedings of the Fourth International Colloquium on Paratuberculosis 1994. International Association for Paratuberculosis Inc. Rehoboth, Mass.

[228] Van Kruiningen, H. J., Chiodini, R. J.; Thayer, W. R.; Coutu, J. A.; Merkal, R. S.; Runnels, P. L. (1986): Experimental disease in infant goats induced by a *Mycobacterium* isolated from a patient with Crohn´s disease. *Dig. Dis. Sci.* **31**: 1351-1360.

[229] Chiodini RJ; Van Kruiningen HJ; Merkal RS; Thayer WR; Coutu JA. (1984) Possible role of mycobacteria in inflammatory bowel disease. I. An unclassified *Mycobacterium* species isolated from patients with Crohn's disease. *Dig. Dis. Sci*. 29(12): 1073-9

[230] Moss MT; Sanderson J; Tizard M; Hermon-Taylor J; El-Zaatari F; Markesich D; Graham D. (1992) PCR detection of *Mycobacterium paratuberculosis* in long term cultures from Crohn's disease tissues. *Gut*; 33:1209-1213.

[231] Wall S; Kunze ZM; Saboor S; Soufleri J; Seechurn P; Chiodini R; McFadden JJ. (1993) Identification of spheroplast-like agents isolated from tissues of patients with Crohn's disease and control tissues by polymerase chain reaction. *J Clin Microbiol;* 31:1241-1245.

[232] Sanderson JD; Moss MT; Tizard MLV; Hermon-Taylor J. (1992) *Mycobacterium paratuberculosis* DNA in Crohn's disease tissue. *Gut;* 33:890-896.

[233] Lisby G; Andersen J; Engbaek K; Binder V. (1994) *Mycobacterium paratuberculosis* in intestinal tissue from patients with Crohn's disease demonstrated by a nested primer polymerase chain reaction. *Scand J Gastroenterol* ; 29:923-929.

[234] Dell'Isola B; Poyart C; Goulet O; Mougenot J-F; Sadoun-Journo E; Brousse N; Schmitz J; Ricour C; Berche P. (1994) Detection of *Mycobacterium paratuberculosis* by polymerase chain reaction in children with Crohn's disease. *J Inf Dis,* 169:449-51.

[235] Fidler HM; Thurrell W; Johnson N McJ; Rook GAW; McFadden JJ. (1994) Specific detection of *Mycobacterium paratuberculosis* DNA associated with granulomatous tissue in Crohn's disease. *Gut;* 35:506-510.

[236] Murray A; Oliaro J; Schlup MMT; Chadwick VS. (1995) *Mycobacterium paratuberculosis* and inflammatory bowel disease: frequency distribution in serial colonoscopic biopsies using the polymerase chain reaction. *Microbios*; 83:217-228.

[237] Suenaga K, Yokoyama Y, Okazaki K, Yamamoto Y. (1995) Mycobacteria in the intestine of Japanese patients with inflammatory bowel disease. *Am. J; Gastroenterol;* 90:76-80.

[238] Erasmus DL; Victor TC; Van Eeden PJ; Falck V; Van Helden P. (1995) *Mycobacterium paratuberculosis* and Crohn's disease. Gut; 36:942.

[239] Gan H; Ouyang Q; Bu H. (1997) *Mycobacterium paratuberculosis* in the intestine of patients with Crohn's disease. *Chung Hua Nei Ko Tsa Chih;* 36:228-30.

[240] Del Prete R; Quaranta M; Lippolis A; Giannuzzi V; Mosca A; Jirillo E; Miragliotta G. (1998) Detection of *Mycobacterium paratuberculosis* in stool samples of patients with inflammatory bowel disease by IS*900*-based PCR and colorimetric detection of amplified DNA. *J Microbiol Methods*; 33:105-114.

[241] Mishina D; Katsel P; Brown ST; Gilberts ECAM; Greenstein RJ. (1996) On the etiology of Crohn disease. Proc Natl Acad Sci USA.; 93:9816-9820
[242] Rowbotham DS; Mapstone NP; Trejdosiewicz LK; Howdle PD; Quirke P. (1995) *Mycobacterium paratuberculosis* DNA not detected in Crohn's disease tissue by fluorescent polymerase chain reaction. *Gut*; 37:660-667
[243] Dumonceau J-M; Van Gossum A; Adler M; Fonteyne P-A; Van Vooren J-P; Deviere J; Portaels F. (1996) No *Mycobacterium paratuberculosis* found in Crohn's disease using the polymerase chain reaction. *Dig Dis & Sci;* 41:421-426
[244] Frank TS; Cook SM. (1996) Analysis of paraffin sections of Crohn's disease for *Mycobacterium paratuberculosis* using polymerase chain reaction. *Modern Path.*; 9:32-35
[245] Al-Shamali M; Khan I; Al-Nakib B; Al-Hassan F; Mustafa AS. (1997) A multiplex polymerase chain reaction assay for the detection of *Mycobacterium paratuberculosis* DNA in Crohn's disease tissue. *Scand. J. Gastroenterol;* 32:819-823
[246] Kallinowski F; Wassmer A; Hofmann MA; Harmsen D; Heesemann J; Karch H; Herfarth Ch; Buhr HJ. (1998) Prevalence of enteropathogenic bacteria in surgically treated chronic inflammatory bowel disease. *Hepato. Gastroenterol;* 45:1552-1558.
[247] Clarkson WK; Presti ME; Petersen PF; Zachary PE; Fan WX; Leonardi CL; Vernava AM; Longo WE; Kreeger JM. (1998) Role of *Mycobacterium paratuberculosis* in Crohn's disease. *Dis Colon Rectum*; 41:195-199
[248] Chiba M; Fukushima T; Horie Y; Iizuka M; Masamune O. (1998) No *Mycobacterium paratuberculosis* detected in intestinal tissue, including Peyer's patches and lymph follicles, of Crohn's disease. *J Gastroenterol* ;33:482-487
[249] Cellier C; De Beenhouwer H; Berger A; Penna C; Carbonnel F; Parc R; Cugnenc P-H; Le Quintrec Y; Gendre J-P; Barbier J-P; Portaels F. (1998) *Mycobacterium paratuberculosis* and *Mycobacterium avium* subsp *silvaticum* DNA cannot be detected by PCR in Crohn's disease tissue. *Gastroenterol. Clin Biol;* 22:675-678
[250] Kanazawa K; Haga Y; Funakoshi O; Nakajima H; Munakata A; Yoshida Y. (1999) Absence of *Mycobacterium paratuberculosis* DNA in intestinal tissues from Crohn's disease by nested polymerase chain reaction. *J Gastroenterol*; 34:200-206
[251] Tiveljung A; Soderholm JD; Olaison G; Jonasson J; Monstein H-J. (1999) Presence of eubacteria in biopsies from Crohn's disease inflammatory lesions as determined by 16S rRNA gene-based PCR. *J. Med. Microbiol*.; 48:263-268
[252] Hermon-Taylor J. (1998) The causation of Crohn's disease and treatment with antimicrobial drugs. *Ital J Gastroenterol & Hepatol;* 30:607-10
[253] Hermon-Taylor J; Bull TJ; Sheridan JM; Cheng J; Stellakis ML; Sumar N. (1999) The Causation of Crohn's Disease by *Mycobacterium avium* subspecies *paratuberculosis*. *Can. J. Gastroenterol.*; in press.
[254] Challans JA; Stevenson K; Reid HW; Sharp JM (1994) A rapid method for the extraction and detection of Mycobacterium avium subspecies paratuberculosis from clinical specimens. *Vet. Rec.*; 134(4):95-6
[255] Hermon-Taylor J; Barnes N; Clarke C; Finlayson C. (1998) *Mycobacterium paratuberculosis* cervical lymphadenitis, followed five years later by terminal ileitis similar to Crohn's disease. *Brit. Med. J.;* 316:449-453
[256] Naser S et al. Personal communication. 1999
[257] Naser S. (2000) Isolation of *Mycobacterium avium* subsp *paratuberculosis* from breast milk of Crohn’s disease patients. *Am. J. Gastroenterol.:* in press.
[258] Noordhoek GT; Kolk AHJ; Bjune G; Catty D; Dale JW; Fine PEM; Godfrey-Faussett P; Cho S-N; Shinnick T; Svenson SB; Wilson S; van Embden JDA. (1994) Sensitivity and specificity of PCR for detection of *Mycobacterium tuberculosis*: a blind comparison study among seven laboratories. *J. Clin. Microbiol.*; 32:277-284
[259] Schmidt BL. (1997) PCR in laboratory diagnosis of human *Borrelia burgdorferi* infections. *Clin. Microbiol. Revs*; 10:185-201.
[260] Navarro E; Fernandez JA; Escribano J; Solera J. (1999) PCR assay for diagnosis of human brucellosis. *J. Clin. Microbiol.;* 37:1654-1655

[261] de Wit MYL; Faber WR; Kreig SR; Douglas JT; Lucas SB; Montreewasuwat N; Pattyn SR; Hussain R; Ponnighaus JM; Hartskeerl RA; Klatser PR. (1991) Application of a polymerase chain reaction for the detection of *Mycobacterium leprae* in skin tissue. *J. Clin. Microbiol.;* 29:906-910

[262] Böddinghaus B; Wolters J; Heikens W; Bottger EC. (1990) Phylogenetic analysis and identification of different serovars of *Mycobacterium intracellulare* at the molecular level. *FEMS Microbiol.Letts.* 70; 197-203

[263] Edwards LB; Palmer CE. Isolation of atypical mycobacteria from healthy persons. (1959) *Am. Rev. Resp. Dis.* 80; 747-749

[264] Portaels F. Isolation of Mycobacteria from Healthy Persons' Stools. (1988) *Int.J.Leprosy*. 56; 469-471

[265] Horsburgh CR. (1998) Epidemiology of *Mycobacterium avium* Complex. In: *Mycobacterium avium*-Complex Infection. Korvick JS & Benson CA (eds) Lung Biology in Health and Disease. 87; 1-22

[266] Thayer WR; Bozic CM; Camphausen RT; McNeil M. (1990) Implications of Antibodies to Pyruvylated Glucose in Healthy Populations for Mycobacterioses and Other Infectious Diseases. *J. Clin. Microbiol.* 28; 714-718

[267] Matthews N; Mayberry JF; Rhodes J; Neale J; Munro J; Wensinck F; Lawson GHK; Rowland AC; Berkhoff GA; Barthold SW. (1980) Agglutinins to bacteria in Crohn's disease. *Gut*; 21:376-380.

[268] Thayer WR; Coutu JA; Chiodini RJ; Van Kruiningen HJ; Merkal RS. (1984) Possible role of mycobacteria in inflammatory bowel disease. *Dig Dis & Sci*; 29:1080-1085.

[269] Cho S-N; Brennan PJ; Yoshimura HH; Korelitz BI; Graham DY. (1986) Mycobacterial aetiology of Crohn's disease: serologic study using common mycobacterial antigens and a species-specific glycolipid antigen from *Mycobacterium paratuberculosis. Gut;* 27:1353-1356.

[270] Brunello F; Pera A; Martini S; Marino L; Astegiano M; Barletti C; Gastaldi P; Verme G; Emanuelli G. (1991) Antibodies to *Mycobacterium paratuberculosis* in patients with Crohn's disease. *Dig. Dis. & Sci.*; 36:1741-1745

[271] Tanaka K; Wilks M; Coates PJ; Farthing MJG; Walker-Smith, JA; Tabaqchali S. (1991) *Mycobacterium paratuberculosis* and Crohn's disease. *Gut*, 32:43-45.

[272] Morgante P; Lopez B; Barrera L; Ritacco V; de Kantor I N. (1994) Respuesta humoral a micobacterias en pacientes con enfermedad de Crohn. Medicina (Buenos Aires) ; 54:97-102

[273] Chiodini R J. (1993) Abolish *Mycobacterium paratuberculosis* Strain 18. *J. Clin. Microbiol.*;31:1956-7.

[274] Kobayashi K; Brown WR; Brennan PJ; Blaser MJ. (1988) Serum antibodies to mycobacterial antigens in active Crohn's disease. *Gastroenterol.;* 94:1404-1411.

[275] Markesich DC; Sawai ET; Butel JS; Graham DY. (1991) Investigations on etiology of Crohn's disease. Humoral response to stress (Heat shock) proteins. *Dig. Dis. & Sci.;* 36:454-460.

[276] Stainsby KJ; Lowes JR; Allan RN; Ibbotson JP. (1993) Antibodies to *Mycobacterium paratuberculosis* and nine species of environmental mycobacteria in Crohn's disease and control subjects. *Gut*; 34:371-374.

[277] Suenaga K; Yokoyama Y; Nishimori I; Sano S; Morita M; Okazaki K; Onishi S. (1999) Serum antibodies to *Mycobacterium paratuberculosis* in patients with Crohn's disease. *Dig. Dis. & Sci.;* 44:1202-1207

[278] Seldenrijk CA; Drexhage HA; Meuwissen SGM; Meijer CJLM. (1990) T-Cellular immune reactions (in macrophage inhibition factor assay) against *Mycobacterium paratuberculosis, Mycobacterium kansasii, Mycobacterium tuberculosis, Mycobacterium avium* in patients with chronic inflammatory bowel disease. *Gut*; 31:529-535.

[279] Ibbotson JP; Lowes JR; Chahal H; Gaston JSH; Life P; Kumararatne DS; Sharif H; Alexander-Williams J; Allan RN. (1992) Mucosal cell-mediated immunity to mycobacterial, enterobacterial and other microbial antigens in inflammatory bowel disease. *Clin. Exp. Immunol.;* 87:224-230.

[280] Rowbotham DS; Howdle PD; Trejdosiewicz LK. (1995) Peripheral cell-mediated immune response to mycobacterial antigens in inflammatory bowel disease. *Clin. Exp. Immunol.*; 102:456-461

[281] Chiodini RJ; Thayer WR; Coutu JA. Presence of *Mycobacterium paratuberculosis* in animal health care workers. Chiodini RJ, Hines ME and Collins MT eds. In: Proceedings of the Fifth International Colloquium on Paratuberculosis Int. Association for Paratuberculosis, Rehoboth MA 1996 pp324-328

[282] Elsaghier A; Prantera C; Moreno C; Ivanyi J. (1992) Antibodies to *Mycobacterium paratuberculosis*-specific protein antigens in Crohn's disease. *Clin. Exp. Immunol.;* 90:503-508

[283] El-Zaatari FAK; Naser SA; Engstrand L; Hachem CY; Graham DY. (1994) Identification and characterization of *Mycobacterium paratuberculosis* recombinant proteins expressed in *E.coli. Curr. Microbiol.*; 29:177-184.

[284] El-Zaatari FAK; Naser SA; Graham DY. (1997) Characterization of specific *Mycobacterium paratuberculosis* recombinant clone expressing 35,000-molecular weight antigen and reactivity with sera from animals with clinical and subclinical Johne's disease. *J. Clin. Microbiol.;* 35:1794-1799.

[285] El-Zaatari FAK; Naser SA; Hulten K; Burch P; Graham DY. (1999) Characterization of *mycobacterium paratuberculosis* p36 antigen and its seroreactivities in Crohn's disease. *Curr. Microbiol.*; 39:115-9

[286] Naser SA; Hulten K; Shafran I; Graham DY; El-Zaatari FAK. (1999) Specific seroreactivity of Crohn’s disease patients against p35 and p36 antigens of *M avium* subsp. *paratuberculosis*. In: Proceedings of the Sixth International Colloquium on Paratuberculosis, Melbourne , Australia; Pages 525-530. Eds. Manning E.J.B. and Collins M.T.

[287] Sumar N; Tizard MLV; Doran T; Austen BM; Hermon-Taylor J. (1994) Epitope mapping of IS*900* +ve strand encoded protein p43 using sera from humans with chronic enteritis Crohn's disease. In: Chiodini RJ, Collins MT, Bassey EO eds. Proceedings of the Fourth International Colloquium on Paratuberculosis. Publ. Int. Association for Paratuberculosis Inc. Rehoboth USA. 1994;pp273-278.

[288] Reddy M, Sumar N, Martin H, Bull T, Tizard MLV, Hermon-Taylor J. (1998) A peptide epitope in the C-terminus of p43 encoded by IS*900* in *Mycobacterium paratuberculosis* is recognised by Crohn's disease sera. *Digestion;* 59 (suppl3):114.

[289] Cohavy O; Harth G; Horwitz M; Eggena M; Landers C; Sutton C; Targan SR; Braun J. Identification of a novel mycobacterial histone H1 homologue (HupB) as an antigenic target of pANCA monoclonal antibody and serum immunoglobulin A from patients with Crohn's disease. *Infect. Immun*.; 67: 6510-17.

[290] Blaser MJ; Miller RA; Lacher J; Singleton JW (1984) Patients with active Crohn's disease have elevated serum antibodies to antigens of seven enteric bacterial pathogens. *Gastroenterology* 1984 Oct;87(4):888-94

[291] Tabaqchali S; O'Donoghue DP; Bettelheim KA. (1978) *Escherichia coli* antibodies in patients with inflammatory bowel disease. *Gut.;* 19:108-113

[292] Persson S; Danielsson D. (1979) On the occurrence of serum antibodies to *Bacteroides fragilis* and serogroups of *E.coli* in patients with Crohn's disease. *Scand. J. Infect. Dis*.; Suppl 19:61-67.

[293] Ruemmele FM; Targan SR; Levy G.;Dubinsky M; Braun J; Seidman EG. (1998) Diagnostic accuracy of serological assays in pediatric inflammatory bowel disease. *Gastroenterology*; 115:822-829

[294] Wurzner R. (1999) Evasion of pathogens by avoiding recognition or eradication by complement, in part via molecular mimicry. *Mol. Immunol*.; 36:249-60.

[295] Ward M; McManus JPA. (1975) Dapsone in Crohn's disease. *Lancet;* 1(7918):1236-37

[296] Paris JC; Simon V; Paris J. (1977) Etude critique des effets de la medication antituberculeuse dans une serie de 52 cas de formes severes de la maladie de Crohn. *Annal Gastroenterol & Hepatol;* 13:427-433.

[297] Toulet J; Rousselet J; Viteau J-M. (1979) La rifampicine dans le traitement de la maladie de Crohn. *Gastroenterol. Clin. Biol*.; 3:209-211

[298] Schultz MG; Rieder HL; Hersh T; Riepe S. (1987) Remission of Crohn's disease with antimycobacterial chemotherapy. *Lancet* 2(8572):1391-2.

[299] Warren JB; Rees HC; Cox TM. (1986) Remission of Crohn's disease with tuberculosis chemotherapy. *New Eng. J. Med.;* 314:182

[300] Wirostko E; Johnson L; Wirostko B. (1987) Crohn's disease: Rifampin treatment of the ocular and gut disease. *Hepato-Gastroenterol;* 34:90-93.

[301] Picciotto A; Gesu GP; Schito GC; Testa R; Varagona G; Celle G. (1988) Antimycobacterial chemotherapy in two cases of inflammatory bowel disease. *Lancet* 1(8584):536-7.

[302] Prantera C; Bothamley G; Levenstein S; Mangiarotti R; Argentieri R. (1989) Crohn's disease and mycobacteria: two cases of Crohn's disease with high anti-mycobacterial antibody levels cured by dapsone therapy. *Biomed. Pharmacother*; 43:295-299

[303] Hampson SJ; Parker MC; Saverymuttu SH; Joseph AE; McFadden J-J P; Hermon-Taylor J. (1989) Quadruple antimycobacterial chemotherapy in Crohn's disease: results at 9 months of a pilot study in 20 patients. *Aliment. Pharmacol. Therap.*; 3:343-352.

[304] Jarnerot G; Rolny P; Wickbom G; Alemayehu G. (1989) Antimycobacterial therapy ineffective in Crohn's disease after a year. *Lancet*; 1(8630):164-165.

[305] Shaffer JL; Hughes S; Linaker BD; Baker RD; Turnberg LA. (1984) Controlled trial of rifampicin and ethambutol in Crohn's disease. *Gut*; 25:203-205

[306] Afdhal NH; Long A; Lennon J; Crowe J; O'Donoghue DP. (1991) Controlled trial of antimycobacterial therapy in Crohn's disease. *Dig Dis & Sci;* 36:449-453.

[307] Rutgeerts P; Geboes K; Vantrappen G; Van Isveldt J; Peeters M; Penninckx F; Hiele M. (1992) Rifabutin and ethambutol do not help recurrent Crohn's disease in the neoterminal ileum. *J. Clin. Gastroenterol.*; 15:24-8.

[308] Prantera C; Kohn A; Mangiarotti R; Andreoli A; Luzi C. (1994) Antimycobacterial therapy in Crohn's disease: results of a controlled, double-blind trial with a multiple antibiotic regimen. *Am. J. Gastroenterol.*; 89:513-518.

[309] Swift GL; Srivastava ED; Stone R; Pullan RD; Newcombe RG; Rhodes J; Wilkinson S; Rhodes P; Roberts G; Lawrie BW; Evans KT; Jenkins PA; Williams GT; Strohmeyer G; Kreuzpaintner G; Thomas GAO; Calcraft B; Davies PS; Morris TJ; Morris J. (1994) Controlled trial of anti-tuberculous chemotherapy for two years in Crohn's disease. *Gut*; 35;363-368.

[310] Thomas GAO; Swift GL; Green JT; Newcombe RG; Braniff-Mathews C; Rhodes J; Wilkinson S; Strohmeyer G; Kreuzpainter G. (1998) Controlled trial of antituberculous chemotherapy in Crohn's disease: a five year follow up study. *Gut*; 42:497-500.

[311] Wolinsky E. (1979) Nontuberculous mycobacteria and associated diseases. *Am. Rev. Resp. Dis.*; 119:107-159

[312] Iseman MD; Corpe RF; O'Brien RJ; Rosenzwieg DY; Wolinsky E (1985) Disease due to *Mycobacterium avium intravellulare*. *Chest;* 87:139S-149S

[313] In: Korvick JA, Benson CA. eds. *Mycobacterium avium* -Complex infection. Lung Biology in Health and Disease. Marcel Dekker. New York vol 87 1996.

[314] Barry CE; Mdluli K. (1996) Drug sensitivity and environmental adaptation of mycobacterial cell wall components. *Trends Microbiol.;* 4:275-281.

[315] Bottger EC. (1994) Resistance to drugs targeting protein synthesis in mycobacteria. *Trends Microbiol.;* 2:416-421

[316] Rastogi N.; Falkinham JO. (1996) Solving the dilemma of antimycobacterial chemotherapy. *Res.Microbiol*;147: 7-10 and other papers in this journal.

[317] Lety MA; Nair S; Berche P; Escuyer V. (1997) A single point mutation in the *embB* gene is responsible for resistance to ethambutol in *Mycobacterium smegmatis.* *Antimicrob. Agents Chemother;* 41:2629-2633

[318] Musser JM. (1995) Antimicrobial agent resistance in Mycobacteria: molecular genetic insights. *Clin. Microbiol. Revs*; 8:496-514.

[319] O'Brien RJ; Lyle MA; Snider DE. (1987) Rifabutin(Ansamycin LM 427): A new rifamycin-S derivative for the treatment of mycobacterial diseases. *Rev. Inf. Dis.;* 9:519-530.

[320] Brogden RN; Fitton A. (1994) Rifabutin. A review of its antimicrobial activity, pharmacokinetic properties and therapeutic efficacy. *Drugs*; 47:983-1009.

[321] Saito J; Sato K; Tomioka H. (1988) Comparative in vitro and in vivo activity of rifabutin and rifampicin against *Mycobacterium avium* complex. *Tubercle*; 69:187-192.

[322] Inderlied CB; Kolonski PT; Wu M; Young MS. (1989). In vitro and in vivo activity of azithromycin (CP 62,993) against the *Mycobacterium avium* complex. *J. Inf. Dis.;* 159: 994-997.

[323] Rastogi N; Labrousse V. (1991) Extracellular and Intracellular activities of clarithromycin used alone and in association with ethambutol and rifampin against M*ycobacterium avium* complex. *Antimicrob. Agents Chemother.*; 35: 462-470.

[324] Piersimoni C; Tortoli E; Mascellino MT; Passerini Tosi C; Sbaraglia G; Mandler F; Bistoni F; Bornigia S; De Sio G; Goglio A et al.. (1995) Activity of seven antimicrobial agents, alone and in combination, against AIDS-associated isolates of *Mycobacterium avium* complex. *J. Antimicrob. Chemother.;* 36: 497-502

[325] Onyeji CO, Nightingale CH, Tessier PR, Nicolau DP; Bow LM. (1995) Activities of clarithromycin, azithromycin and ofloxacin in combination with liposomal or unencapsulated

granulocyte-macrophage colony-stimulating factor against intramacrophage *Mycobacterium avium-Mycobacterium intracellulare. J. Inf. Dis.;*172:810-16.

[326] Gladue RP; Snider ME. (1990) Intracellular accumulation of azithromycin by cultured human fibroblasts. *Antimicrob. Agents Chemother.*; 34:1056-1060

[327] Chiodini RJ. (1991) Antimicrobial activity of rifabutin in combination with two and three other antimicrobial agents against strains of *Mycobacterium paratuberculosis. J. Antimicrob. Chemother.*; 27:171-176.

[328] Rastogi N; Goh KS; Labrousse V. (1992) Activity of clarithromycin compared with those of other drugs against *Mycobacterium paratuberculosis* and further enhancement of its extracellular and intracellular activities by ethambutol. *Antimicrob. Agents Chemother.*; 36:2843-2846

[329] Ghebremichael S; Svenson SB; Kallenius G; Hoffner SE. (1996) Antimycobacterial synergism of clarithromycin and rifabutin. *Scand. J. Infect. Dis.*; 28:387-90

[330] McClure HM; Chiodini RJ; Anderson DC; Swenson RB; Thayer WR; Coutu JA. (1987) *Mycobacterium paratuberculosis* infection in a colony of Stumptail Macaques (*Macaca arctoides). J. Inf. Dis.*; 155:1011-1019.

[331] Thayer W; Coutu J; Chiodini R et al. Use of rifabutin and streptomycin in the therapy of Crohn's disease - preliminary results. In: R.P. MacDermott ed. Inflammatory Bowel Disease: current status and future approach. Publ. Elsevier Amsterdam. 1988. 565-568.

[332] Graham DY; Al-Assi MT; Robinson M. (1995) Prolonged remission in Crohn's disease following therapy for *Mycobacterium paratuberculosis* infection. *Gastroenterology*, 108:A826.

[333] Goodgame RW; Kimball K; Akram S; Graham DY, Ou, C-N. (1999) Randomized controlled trial of clarithromycin & ethambutol in the treatment of Crohn's disease. *Gasteroenterology;* 116: A725.

[334] Leiper K; Campbell BJ; Rhodes JM. (1999) Treatment of active Crohn's disease with clarithromycin. *Gut*; 44: 299.

[335] Meier A; Heifets L; Wallace RJ; Zhang Y; Brown BA; Sander P, Bottger EC. (1996) Molecular mechanisms of clarithromycin resistance in *Mycobacterium avium*: observation of multiple 23S rDNA mutations in a clonal population. *J. Inf. Dis.*; 174: 354-60.

[336] Ji B; Lounis N; Truffot-Pernot C; Grosset J (1992) Selection of resistant mutants of *Mycobacterium avium* in Beige mice by clarithromycin monotherapy. *Antimicrob. Agents and Chemo*; 36: 2839-2840.

[337] Doucet-Populaire F, Truffot-Pernot C, Grosset J, Jarlier V. (1995) Acquired resistance in *Mycobacterium avium* complex strains isolated from AIDS patients and beige mice during treatment with clarithromycin. *J. Antimicrob. Chemother.;* 36:129-136.

[338] Bermudez LE; Petrofsky M; Kolonoski P; Young LS. (1998) Emergence of *Mycobacterium avium* populations resistant to macrolides during experimental chemotherapy. *Antimicrob. Agents Chemother.*; 42:180-183

[339] Gui GPH; Thomas PRS; Tizard MLV; Lake J; Sanderson JD; Hermon-Taylor J. (1997) Two-year-outcomes analysis of Crohn's disease treated with rifabutin and macrolide antibiotics. *J. Antimicrob. Chemother.*; 39: 393-400.

[340] Borody TJ; Pearce L; Bampton PA et al. (1998) Treatment of severe Crohn's disease (CD) using rifabutin-macrolide-clofazimine combination: interim report. *Gastroenterology*; 114: A938

[341] Bergstrand O; Hellers G, (1983) Breast-feeding during infancy in patients who later develop Chron’s disease. *Scand. J Gastroenterol:* 18 903-906

[342] Doyle TM. (1954) Isolation of Johne's bacilli from the udders of clinically affected cows. *Br. Vet. J.*; 110: 218.

[343] Smith HW. (1960) The examination of milk for the presence of *Mycobacterium Johnei. J. Path. Bacteriol*; 80: 440-442

[344] Taylor TK; Wilks CR; McQueen DS. (1981) Isolation of *Mycobacterium paratuberculosis* from the milk of a cow with Johne's disease. *Vet. Rec.* 109(24): 532-533

[345] Sweeney RW; Whitlock RH; Rosenberger AE. (1992) *Mycobacterium paratuberculosis* cultured from milk and supramammary lymph nodes of infected asymptomatic cows. *J. Clin. Microbiol.*; 30: 166-171.

[346] Streeter RN; Hoffsis GF; Bech-Nielsen S; Shulaw WP; Rings DM. (1995) Isolation of *Mycobacterium paratuberculosis* from colostrum and milk of subclinically infected cows. *Am. J. Res.*; 56: 1322-1324

[347] Collins MT. (1997) *Mycobacterium paratuberculosis*: A potential food-borne pathogen ? *J. Dairy Sci.*; 80: 3445-3448
[348] Mason O; Rowe MT; Ball HJ. (1997) Is *Mycobacterium paratuberculosis* a possible agent in Crohn's disease ? Implications for the dairy industry. *Milchwissenschaft*; 52: 311-316.
[349] Chiodini RJ; Hermon-Taylor J. (1993) The thermal resistance of *Mycobacterium paratuberculosis* in raw milk under conditions simulating pasteurization. *J. Vet. Diagn. Invest.*; 5: 629-631
[350] Grant IR; Ball HJ; Neill SD; Rowe MT. (1996) Inactivation of *Mycobacterium paratuberculosis* in cows' milk at pasteurization temperatures. *Appl. Environ. Microbiol.;* 62: 631-636
[351] Meylan M; Rings DM; Shulaw WP, Kowalski JJ, Bech-Nielsen S, Hoffsis GF. (1996) Survival of *Mycobacterium paratuberculosis* and preservation of immunoglobulin G in bovine colostrum under experimental conditions simulating pasteurization. *Am. J. Vet. Res.;* 57: 1580-85.
[352] Hope AF; Tulk PA; Condron RJ. Pasteurization of *Mycobacterium paratuberculosis* in whole milk. In: RJ Chiodini, ME Hives, MT Colins. eds. Proceedings of the Fifth International Colloquium on Paratuberculosis 1996. Publ. Int Assn for Paratuberculosis Inc, Rehoboth MA USA. pp377-382.
[353] Grant IR; Ball HJ; Rowe MT. (1996) Thermal inactivation of several *Mycobacterium* spp. in milk by pasteurization. *Letts in Appl. Microbiol.;* 22: 253-256.
[354] Sung N; Collins MT. (1998) Thermal tolerance of *Mycobacterium paratuberculosis. Appl. Environ. Microbiol.*; 64: 999-1005
[355] Grant IR; Ball HJ; Rowe MT. (1998) Effect of high-temperature, short-time (HTST) pasteurization on milk containing low numbers of *Mycobacterium paratuberculosis. Letts in Appl. Microbiol.*; 26: 166-170.
[356] Grant IR. (1998) Does *Mycobacterium paratuberculosis* survive current pasteurization conditions ? *Appl. Environ. Microbiol.,* 64: 2760
[357] Stabel JR; Steadham EM; Bolin CA. (1997) Heat Inactivation of *Mycobacterium paratuberculosis* in raw milk: Are current pasteurization conditions effective? *Appl. Environ. Microbiol.*; 63: 4975-4977.
[358] Keswani J; Frank JF. (1998) Thermal inactivation of *Mycobacterium paratuberculosis* in milk. *J. Food Protect.*; 61: 974-978
[359] Millar D; Ford J; Sanderson J; Withey S; Tizard M; Doran T; Hermon-Taylor J. (1996) IS*900* PCR to detect *Mycobacterium paratuberculosis* in retail supplies of whole pasteurized cows' milk in England and Wales. *Appl. Environ. Microbiol.;* 62: 3446-3452.
[360] Grant IR; Ball HJ; Rowe MT. (1999) Effect of higher pasteurization temperatures, and longer holding times at 72°C, on the inactivation of *Mycobacterium paratuberculosis* in milk. *Letts in Appl. Microbiol.*; 28: 461-465
[361] Grant IR; Ball HJ; Rowe MT. (1998) Isolation of *Mycobacterium paratuberculosis* from milk by immunomagnetic separation. *Appl. Environ Microbiol.;* 64: 3153-3158.
[362] Advisory Committee on the Microbiological Safety of Food. (1999) National study on the Microbiological quality and heat processing of cows milk. Tabled Paper ACM/458 December 1999.
[363] Horsburgh CR, (1996). Epidemiology of *Mycobacterium avium* complex. In: J.A. Korvick and C.A.Benson eds.: *Mycobacterium avium* complex infection. Publ. Marcel Dekker Inc. , New York pp 1-22.
[364] Collins CH; Grange JM; Yates MD. (1984) Mycobacteria in water. *J. Appl. Bacteriol.*; 57: 193-211.
[365] Falkinham JO. (1996) Epidemiology of infection by nontuberculous mycobacteria. *Clin. Microbiol. Revs;* 9: 177-215.
[366] Covert TC; Rodgers MR; Reyes AL, Stelma GN. (1999) Occurrence of nontuberculous mycobacteria in environmental samples. *Appl. Environ Microbiol.;* 65: 2492-2496.
[367] Aronson T; Holtzman A; Glover N; Boian M; Froman S; Berlin OGW; Hill H; Stelma G. (1999) Comparison of large restriction fragments of *Mtcobacterium avium* isolates recovered from AIDS and non-AIDS patients with those of isolates from potable water. *J. Clin. Microbiol.;* 37: 1008-1012
[368] Mansfield KG; Lackner AA. (1997) Simian immunodeficiency virus-inoculated Macaques acquire *Mycobacterium avium* from potable water during AIDS. *J. Inf. Dis.*; 175: 184-7.
[369] Riemann HP; Abbas B. (1983) Diagnosis and control of bovine paratuberculosis (Johne's Disease). *Adv. Vet. Sci. & Comp. Med.;* 27: 481-506

[370] Jorgensen JB. (1977) Survival of *Mycobacterium paratuberculosis* in slurry. *Nord. Vet. Med.;* 6: 267-70

[371] Stehman SM; Rossiter CA; Shin SJ; Chang YF; Lein DH. Johne's disease in a fallow deer herd: Accuracy of fecal culture and results of environmental sampling. In: Chiodini RJ, Hines ME, Collins MT eds. Proceedings of the Fifth International Colloquium on Paratuberculosis 1996. Publ. Int.Assn for Paratuberculosis, Rehoboth, MA. USA. 1996 pp183-189.

[372] Greig A; Stevenson K; Henderson D; Perez V; Hughes V; Pavlik I; Hines ME; McKendrick I; Sharp JM. (1999) Epidemiological study of paratuberculosis in wild rabbits in Scotland. *J. Clin. Microbiol.;* 37: 1746-1751.

13. Acknowledgements

This report of the Scientific Committee on Animal Health and Animal Welfare is based on the work of a working group established by the Committee. The working group was chaired by Prof. J Badiola. The members of the group are listed below.

Prof. Juan José BADIOLA
Universidad de Zaragoza – Facultad de Veterinaria
C/. Miguel Servet 177
E – 50 013 Zaragoza Espana

Dr. Douwe BAKKER
Institute for Animal Science and Health
P.O.Box 65
NL – 8200 AB Lelystad Nederland

Dr. Juan Francisco GARCIA MARIN
Facultad de Veterinaria, Universidad de Leon
Campus de Vagazano
E – 24071 Leon Espana

Dr. Philippe GILOT
Institut Pasteur, Dépt.de Virologie
Rue Engeland 642
B – 1180 Brussels Belgium

Prof. John HERMON-TAYLOR
St George’s Hospital Medical School, Dept. of Surgery
University of London, Jenner Wing, Cranmer Terrace
UK – London SW1 7ORE United Kingdom

Dr. James Michael SHARP
Moredun Research Institute, Pentlands Science Park ,
Penicuik EH26 0PZ, Scotland, UK

Dr. Shiva SHIVANANDA
Pres. Kennedylaan 188

NL – 2343 GW Oegstgeest Nederland

Dr. Marie-Françoise THOREL
CNEVA - Alfort
BP 67 , rue Pierre Curie, 22
F – 94703 Maisons Alfort Cedex France

Prof. Dominique VUITTON
Université de Franche-Comté, Faculté de Médecine et de Pharmacie
Place Saint Jacques,
F – 25030 Besancon France

Reports of the Scientific Committee on Animal Health and Animal Welfare of the European Union are available at the Committee Website;

http://europa.eu.int/comm/dg24/health/sc/scah/index_en.html

Recent reports include;

No.	Title	Date Adopted
16	Possible links between Crohn's disease and paratuberculosis	21 March 2000
15	The welfare of Chickens kept for Meat Productiuon (broilers).	21 March 2000
14	Bacterial Kidney Disease	8 December 1999
13	Standards for the Microclimate inside animal Transport Road Vehicles	8 December 1999
12	Estimations of the Infective Period for Bluetongue in cattle	8 December 1999
11	Diagnostic Tests for Crimean Congo Haemorrhagic Fever in ratites	11 October 1999
10	Modification of Technical Annexes of Council Directive 64/432/EEC to take account of Scientific Developments regarding Tuberculosis, Brucellosis and Enzootic Bovine Leucosis	11 October 1999
9	Classical Swine Fever in Wild Boar	10 August 1999
8	Animal Welfare Aspects of the Use of Bovine Somatotrophin	10 March 1999

7	Strategy for the emergency vaccination against Foot and Mouth Disease	10 March 1999
6	Welfare Aspects of the production of Foie Gras in Ducks and geese.	16 December 1998
