# Supplementary material for: Long History of Queries about Bovine Paratuberculosis as a Risk Factor for Human Health
Source: Pathogens. 2021 Oct 28;10(11):1394. doi: 10.3390/pathogens10111394 (PMC8622788; doi:10.3390/pathogens10111394)
Supplement: Supplementary file 1 [file pathogens-10-01394-s001.zip › HRUSKA List of participants.pdf]

PathogenCombat
for safe food

Bacterial triggers in the etiology of Crohn's disease and other autoimmune and autoinflammatory diseases

WELCOME

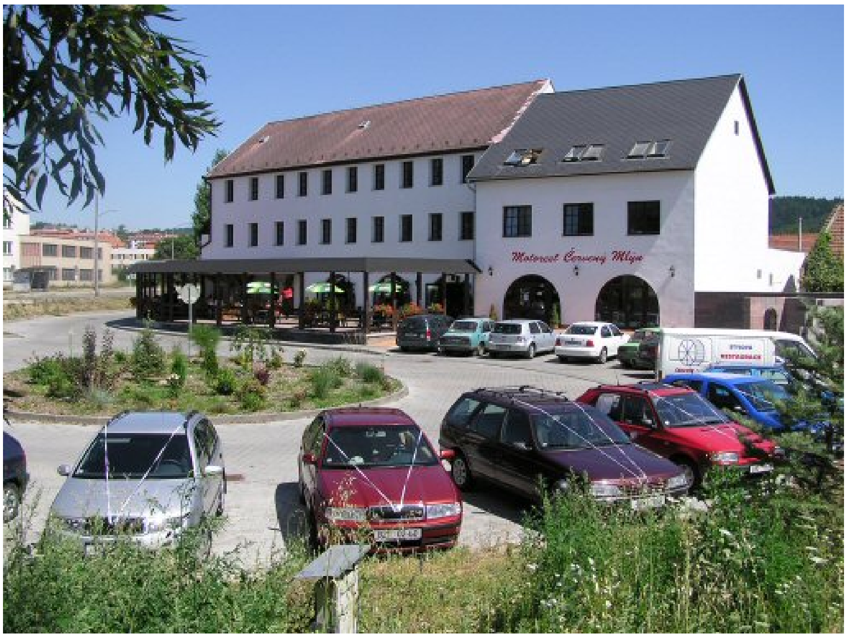

Brno (Tišnov), Czech Republic, 14-15 May, 2009

PathogenCombat integrated research project
of the European Union 6th Frame Programme

OIE Reference Laboratory for Paratuberculosis
Veterinary Research Institute, Brno

PROGRAMME

Wednesday 13 May, 2009
19:00 to 22:00 Get Together (Veterinary Research Institute, Hudcova 70, Brno)

Thursday 14 May 2009
08:30 Visit to the Department of Food and Feed Safety and OIE Reference Laboratory for Paratuberculosis (Meeting room)
10:30 Departure from Hotel IMOS
11:00 Registration
12:00 Lunch
13:15 Opening session (M. Toman, M. Jakobsen, E. Liebana)
13:30 **(1) Crohn's disease** (J. Hermon-Taylor)
14:30 **(2) Paratuberculosis** (M. Collins. R. Juste, I. Pavlík)
15:30 Coffee break
16:00 **(3) Mycobacteria in water and environment** (J. O. Falkinham III)
19:00 Dinner

Friday 15 May 2009
09:00 **(4) Mucosal immunity** (J. Mestecky, H. Tlaskalova-Hogenova)
10:00 **(5) Formula feeding** (K. Hruška)
10:30 Coffee break
11:00 **(6) Food industry and veterinary practice** (D. Bakker)
12:30 Lunch
13:30 **(7) Recommendations** (K. Hruška, R. Goethe, I. Pavlík)
15:00 Closing session
15:45 Porta Coeli
19:00 Dinner

Saturday 16 May 2009
Informal discussion continues, summaries of opinions should be submitted by e-mail before 20 May 2009
09:00 Departure
10:00 Pernštejn Castle
13:00 Lunch (Rustic Restaurant Formanka, Lipůvka)
15:00 Mendel Museum, Brno
16:00 Brno sightseeing
18:00 Accommodation (Hotel IMOS or Na Kytnerce)
19:30 Barbecue Party (Veterinary Research Institute)

GENERAL INFORMATION

The number of discussions will not be limited within the timetable. However one presentation should not exceed three minutes and no more than three slides can be presented during one comment.

Participants in the discussion are requested

- To assess the facts as acceptable or to suggest deletion from the list.
- To formulate more facts.
- To change a hypothesis to a fact if there is enough knowledge already available.
- To suggest new hypotheses.
- To recommend key research to assess the hypotheses.
- To recommend measures to decrease the health risk for consumers (knowledge dissemination should be directed to farmers, food industry, veterinary administration, veterinary practitioners, laboratory diagnosticians, consumers, health and environmental professionals).
- To recommend how to decrease the economic losses in the dairy industry.

The contributions presented during the discussion

- Will be requested by organizers immediately after the session as the written, authorized, concise text, to be published in the Proceedings
- Short summaries of the presented contributions can be written by hand, however a PC will be available in the meeting room and the electronic version is preferred (forms for contributions will be available: please mark the part of discussion according to the time table)

The Proceedings will be distributed before 15 July, 2009.

LIST OF PARTICIPANTS

Norma Arrigoni
Istituto Zooprofilattico Sperimentale della Lombardia e dell'Emilia Romagna
Piacenza, Italy
norma.arrigoni@bs.izs.it

Douwe Bakker
Central Veterinary Institute
Department of Bacteriology and TSE's
Lelystad, The Netherlands
douwe.bakker@wur.nl

Marcel A. Behr
McGill University Health Centre
Montreal, Canada
marcel.behr@mcgill.ca

Ignazio Castagliuolo
University of Padova
School of Pharmacy
Padova, Italy
ignazio.castagliuolo@unipd.it

Michael T. Collins
University of Wisconsin-Madison
School of Veterinary Medicine
2015 Linden Drive
Madison, Wisconsin, USA
mcollin5@wisc.edu

William C. Davis
Washington State University
College of Veterinary Medicine
Department of Veterinary Microbiology and Pathology
Pullman, Washingtom USA
davisw@vetmed.wsu.edu

Joe O. Falkinham III
Virginia Polytechnic Institute and State University
Department of Biological Sciences
Blacksburg, Virginia, USA
jofiii@vt.edu

Ralph Goethe
University of Veterinary Medicine
Institute for Microbiology
Department of Infectious Diseases
Hannover, Germany
ralph.goethe@tiho-hannover.de

Robert J. Greenstein
Veterans Affairs Medical Center
Bronx, New York, USA
BGAxis@aol.com

John Hermon-Taylor
King's College London
Division of Nutritional Sciences
London, U.K.
j.hermon@kcl.ac.uk

Ondřej Hradský
Charles University
University Hospital Motol and Second Faculty of Medicine
Department of Pediatrics
Prague, Czech Republic
ondrej.hradsky@lfmotol.cuni.cz

Karel Hruška
Veterinary Research Institute
Brno, Czech Republic
hruška@vri.cz

Mogens Jakobsen
University of Copenhagen, Faculty of Life Sciences
Department of Food Science
Frederiksberg C, Denmark
moj@life.ku.dk

Ramon Juste
NEIKER-Tecnalia-Instituto Vasco de Investigación y Desarrollo Agrario
Department of Production and Animal Health
Derio, Bizkaia, Spain
rjuste@neiker.net

Markéta Kabourková
Veterinary Research Institute
Brno, Czech Republic
kabourkova@vri.cz

Marija Kaevska
Veterinary Research Institute
Brno, Czech Republic
kaevska@vri.cz

Jindřich Kazda
University of Kiel
Kiel, Germany
j.kazda@t-online.de

Alan Kennedy
International Association for Paratuberculosis
Dublin, Ireland.
webmaster@paratuberculosis.org

David J. Kennedy
Ausvet Animal Health Services
Orange, NSW, Australia
david@ausvet.com.au

Heike U. Köhler
Friedrich-Loeffler-Institut
Institut of Molecular Pathogenesis
Jena, Germany
heike.koehler@fli.bund.de

Petr Králík
Veterinary Research Institute
Brno, Czech Republic
kralik@vri.cz

Petr Kříž
Veterinary Research Institute
Brno, Czech Republic
kriz@vri.cz

Ernesto Liebana
European Food Safety Authority (EFSA)
Scientific Panel on Biological Hazards
Largo N. Palli 5/A
Parma, Italy
ernesto.liebana@efsa.europa.eu

Katarina Logar
University of Ljubljana, Veterinary faculty
Institute of Microbiology and Parasitology
Ljubljana, Slovenia
Katarina.Logar@vf.uni-lj.si

Petr Maňásek
Veterinary Research Institute
Brno, Czech Republic
manasek@vri.cz

Jiří Městecký
The University of Alabama at Birmingham
Department of Microbiology and Medicine
Birmingham, Alabama, USA
mestecky@uab.edu

Wojtek P. Michalski
CSIRO Australian Animal Health Laboratory
Geelong, VIC, Australia
wojtek.michalski@csiro.au

Matjaz Ocepek
Veterinary Faculty
Institute of Microbiology and Parasitology
Ljubljana, Slovenia
matjaz.ocepek@vf.uni-lj.si

Ivo Pavlík
Veterinary Research Institute
Brno, Czech Republic
pavlik@vri.cz

Nicola Pozzato
Istituto zooprofilattico sperimentale delle Venezie
Verona, Italiy
npozzato@izsvenezie.it

Radka Přibylová
Veterinary Research Institute
Brno, Czech Republic
pribylova@vri.cz

Iva Slaná
Veterinary Research Institute
Brno, Czech Republic
slana@vri.cz

Michal Slaný
Veterinary Research Institute
Brno, Czech Republic
slany@vri.cz

Ulrich Sperling
International Forum for Transmissible Animal
Diseases and Food Safety
Bern, Switzerland
ulrich.sperling@tafsforum.org

Dagmar Štanclová
Veterinary Research Institute
Brno, Czech Republic
stanclova@vri.cz

Helena Tlaskalová-Hogenová
Institute of Microbiology
Academy of Sciences of the Czech Republic
Prague, Czech Republic
tlaskalo@biomed.cas.cz

Miroslav Toman
Veterinary Research Institute
Brno, Czech Republic
toman@vri.cz

PARTICIPANTS BY COUNTRIES AND INTERNATIONAL ORGANIZATIONS

Australia
David J. Kennedy
Wojtek P. Michalski

Canada
Marcel A. Behr

Czech Republic
Ondřej Hradský
Karel Hruška
Markéta Kabourková
Marija Kaevska
Petr Králík
Petr Kříž
Petr Maňásek
Ivo Pavlík
Radka Přibylová
Iva Slaná
Michal Slaný
Dagmar Štanclová
Helena Tlaskalová-Hogenová
Miroslav Toman

Denmark
Mogens Jakobsen

Germany
Ralph Goethe
Jindřich Kazda
Heike U. Köhler

Ireland
Alan Kennedy

Italy
Norma Arrigoni
Ignazio Castagliuolo
Nicola Pozzato

Slovenia
Katarina Logar
Matjaz Ocepek

Spain
Ramon Juste

Switzerland
Ulrich Sperling

The Netherlands
Douwe Bakker

United Kingdom
John Hermon-Taylor

USA
Michael T. Collins
William C. Davis
Joe O. Falkinham III
Robert J. Greenstein
Jiří Městecký

European Food Safety Authority
Ernesto Liebana

International Association for Paratuberculosis
Norma Arrigoni, Board Member
Douwe Bakker
Michael T. Collins, Past President
William C. Davis
Robert J. Greenstein
Ramon Juste, President
David J. Kennedy, Board Member
Wojtek P. Michalski
Matjaz Ocepek
Ivo Pavlík, Vice President

International Forum for Transmissible Animal Diseases and Food Safety
Douwe Bakker
Ulrich Sperling

PathogenCombat (EU 6th FP Research Project)
Karel Hruška, External Advisory Board
Mogens Jakobsen, Coordinator
Markéta Kabourková
Petr Králík
Ivo Pavlík
Iva Slaná

PathogenCombat

PathogenCombat is one of 13 FP6 research projects in which the Veterinary Research Institute, Brno, Czech Republic is participating. The project will contribute to food safety and consumer protection by generating more knowledge on food pathogens (bacteria and viruses) that can cause diseases in humans.

Mycobacteria, as food pathogens, are still considered less important than the more well-known salmonella or campylobacters. Mycobacteria are very resistant and may survive pasteurisation and insufficiently rigorous heat treatment of food. Those with compromised immune systems or people who have undergone transplantations may become ill not only after inhaling aerosol droplets containing mycobacteria or following skin injury, but also from contaminated food and water. Large quantities of *Mycobacterium avium* subspecies *paratuberculosis* may be found in the milk and meat of ruminants suffering from paratuberculosis which is a very common disease in cattle and sheep that causes great losses to farmers. Other mycobacterial species may also colonise biofilms in reservoirs and drinking water distribution systems.

Project investigators wish to contribute to the discussion concerning a role for mycobacteria in the etiology of Crohn's disease and certain other autoimmune and autoinflammatory diseases in which peptidoglycans and other components from bacterial cell walls play roles. Mycobacteria may constitute food allergens or triggers of different chronic diseases in humans. The PathogenCombat project contributes to knowledge dissemination in the area of food safety and consumer protection. The detection of mycobacteria in food and in the environment and the development of new diagnostic methods and procedures for the control of paratuberculosis in cattle and sheep are important tools for increasing consumer protection. The project also strives to mediate an exchange of opinions and experience between the experts from different fields and contributes greatly to international collaboration.

The researchers at the Veterinary Research Institute have established and maintain a database of published results for the project. They were also significant contributors to the book *The Ecology of Mycobacteria: Impact on Animal's and Human's Health*. A round-table discussion on 'Bacterial triggers in the etiology of Crohn's disease and other autoimmune and autoinflammatory diseases' is being organised by the PathogenCombat project, taking place in the Czech Republic in May 2009.

Partnership in an IP offers a unique opportunity for cooperation between project investigator teams, allows for communication of findings and the adoption of efficient ways of management and evaluation of research projects. The Czech representative on the PathogenCombat External Advisory Board uses the insights afforded by this position to contribute to the research management in the Czech Republic.

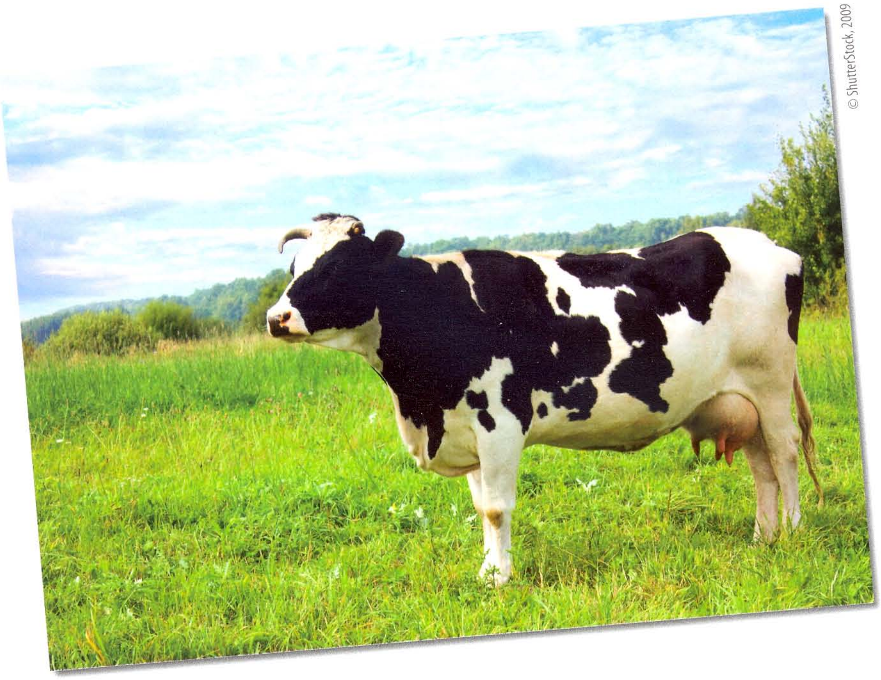

© ShutterStock, 2009

Project title

Control and prevention of emerging and future pathogens at cellular and molecular level throughout the food chain

Project acronym

PathogenCombat

Programme

FP6: 'Food quality and safety'

Project type

IP

Project duration

60 months

EC contribution

EUR 11.27 million

Project coordination

Professor Mogens Jakobsen, The Royal Veterinary and Agricultural University, Copenhagen, Denmark

Czech partner

Professor Ivo Pavlík, Veterinary Research Institute, Brno

Partner countries

16

Partner institutions

44

Project website

http://www.pathogencombat.com

Project on CORDIS

You can access the factsheet of the project on the CORDIS website. Using the Advanced Search function, click on 'Projects', then enter the project acronym in the acronym field.

http://cordis.europa.eu

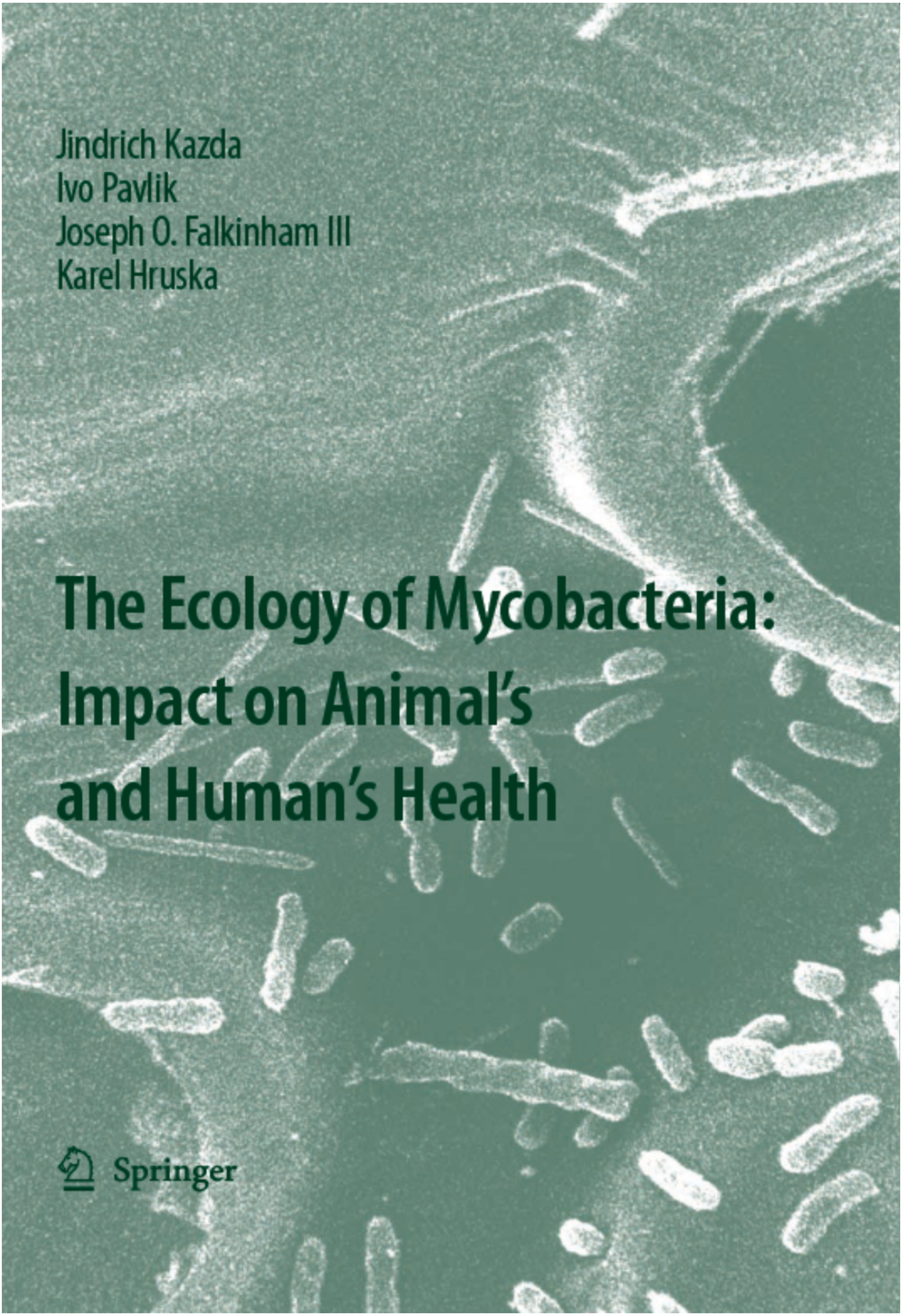

Jindrich Kazda
Ivo Pavlik
Joseph O. Falkinham III
Karel Hruska

The Ecology of Mycobacteria: Impact on Animal's and Human's Health

Springer

Jindřich Kazda · Ivo Pavlik · Joseph O. Falkinham III · Karel Hruska
The Ecology of Mycobacteria:
Impact on Animal's and Human's Health

The *Ecology of Mycobacteria* principally emphasizes the ecological characteristics of the environmental mycobacteria. It is now well understood that the incidence and prevalence of potentially pathogenic mycobacteria is increasing in humans and animals. Further, proof that mycobacteria are normal inhabitants of drinking water distribution systems and household water systems, indicates that humans and animals are surrounded by mycobacteria and thus at risk. It is anticipated that the emphasis on ecology and routes of infection will result in a text of widespread use for clinicians and for research scientists in medicine, academia, and industry. In addition to identifying habitats and thereby sources of mycobacteria infecting humans and animals, the text identifies those mycobacterial characteristics that determine its range of habitats. Additionally, the text comments critically on the available methods to identify those protocols with values in mycobacterial research. In that manner, although there are no chapters specifically devoted to methods, superior methods for mycobacteria will be identified.

A new text is needed for the mycobacteria because the prevalence of disease caused by the environmental potentially pathogenic mycobacteria is increasing. This increase is due to a number of factors. Host factors contribute to an increasing population of individuals more susceptible to mycobacterial infection. For example, the aging of the human population and the increasing frequency of immunosuppressed individuals as a result of infection (e.g. HIV), chemotherapy, and transplant-associated immunosuppression are all factors leading to increased susceptibility of infection with environment derived mycobacteria. Moreover, the role of mycobacteria as triggers in different autoimmune diseases is more and more evident. It is highly probable that peptidoglycans, lipoglycans, lipoproteins, heat shock proteins and some other structures from the mycobacterial cell wall, participate in different pathways of non-specific inflammatory reactions in humans, namely those with a specific genetic disposition. In such events mycobacteria in drinking water and food, even devitalized, have to be considered as a public health risk.

Second, human-engineered systems such as drinking water distribution systems are creating a habitat for the selection and proliferation of the potentially pathogenic mycobacteria. In as much as drinking water brings together overlapping habitats of both mycobacteria and humans and animals, a review of mycobacterial ecology is timely. The ecology of mycobacteria helps to understand the circulation of mycobacteria into the respective disciplines such as epidemiology, epizootology, immunology, environmental ecology, animal husbandry and environment conservation.

ISBN 978-1-4020-9412-5
9 781402 094125

springer.com

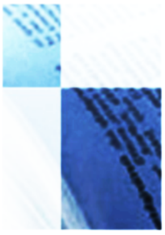

The Ecology of Mycobacteria: Impact on Animal's and Human's Health

Kazda, J., **Pavlik**, I., **Falkinham III**, J.O., **Hruska**, K.

2009, Approx. 260 p., Hardcover
ISBN: 978-1-4020-9412-5

Due: May 2009

approx. 119,95 €

Print
Recommend to others
Download Flyer

All books by these authors
Kazda, Jindrich
Pavlik, Ivo
Falkinham III, Joseph O.
Hruska, Karel

Related subjects
Ecology
Epidemiology
Internal Medicine
Medicine
Microbiology
Public Health

About this book | Table of contents

About this book

The Ecology of Mycobacteria principally emphasizes the ecological characteristics of the environmental mycobacteria. It is now well understood that the incidence and prevalence of potentially pathogenic mycobacteria is increasing in humans and animals. Further, proof that mycobacteria are normal inhabitants of drinking water distribution systems and household water systems, indicates that humans and animals are surrounded by mycobacteria and thus at risk. It is anticipated that the emphasis on ecology and routes of infection will result in a text of widespread use for clinicians and for research scientists in medicine, academia, and industry. In addition to identifying habitats and thereby sources of mycobacteria infecting humans and animals, the text identifies those mycobacterial characteristics that determine its range of habitats. Additionally, the text comments critically on the available methods to identify those protocols with values in mycobacterial research. In that manner, although there are no chapters specifically devoted to methods, superior methods for mycobacteria will be identified.

A new text is needed for the mycobacteria because the prevalence of disease caused by the environmental potentially pathogenic mycobacteria is increasing. This increase is due to a number of factors. Host factors contribute to an increasing population of individuals more susceptible to mycobacterial infection. For example, the aging of the human population and the increasing frequency of immunosuppressed individuals as a result of infection (e.g. HIV), chemotherapy, and transplant-associated immunosuppression are all factors leading to increased susceptibility of infection with environment derived mycobacteria. Moreover, the role of mycobacteria as triggers in different autoimmune diseases is more and more evident. It is highly probable that peptidoglycans, lipoglycans, lipoproteins, heat shock proteins and some other structures from the mycobacterial cell wall, participate in different pathways of non-specific inflammatory reactions in humans, namely those with a specific genetic disposition. In such events mycobacteria in drinking water and food, even devitalized, have to be considered as a public health risk.

Second, human-engineered systems such as drinking water distribution systems are creating a habitat for the selection and proliferation of the potentially pathogenic mycobacteria. In as much as drinking water brings together overlapping habitats of both mycobacteria and humans and animals, a review of mycobacterial ecology is timely. The ecology of mycobacteria helps to understand the circulation of mycobacteria into the respective disciplines such as epidemiology, epizootology, immunology, environmental ecology, animal husbandry and environment conservation.

Written for:

Clinicians, nurses, fellows and interns with specialities in respiratory infection and diseases, *HIV* infection, geriatric medicine, cystic fibrosis, pulmonary alveolar porteinosis, alpha-1 antitrypsin deficiency, immunologists, research scientists, farmers, breeders of livestock, agronomists, field veterinarians, consultants, public health personnel

Keywords:

- Environmental Mycobacteria
- Epidemiology
- Epizootology
- Mycobacterial Ecology
- Potentially Pathogenic Mycobacteria

New Book Alert
If you would like to receive information on new books in the subject area of **Microbial Ecology**, please register:
E-mail
Retype E-mail
GO

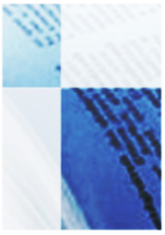

The Ecology of Mycobacteria: Impact on Animal's and Human's Health

Kazda, J., **Pavlik**, I., **Falkinham III**, J.O., **Hruska**, K.

2009, Approx. 260 p., Hardcover

ISBN: 978-1-4020-9412-5

Due: May 2009

approx. 119,95 €

Print
Recommend to others
Download Flyer

All books by these authors

Kazda, Jindrich
Pavlik, Ivo
Falkinham III, Joseph O.
Hruska, Karel

Related subjects

Ecology
Epidemiology
Internal Medicine
Medicine
Microbiology
Public Health

About this book | Table of contents

Table of contents

Preface to the second edition, *J. Kazda*;
Editors' comments;
Contributors;
Authors of photograrhps;
Acknowledgement;
1. The chronology of mycobacteria and the development of mycobacterial ecology, *J. Kazda*;
2. Obligate pathogenic mycobacteria, *J. Kazda, I. Pavlik*
3. Potentially pathogenic mycobacteria, *I. Pavlik, J. O. Falkinham III, J. Kazda*
4. Physiological ecology of environmental saprophytic and potentially pathogenic mycobacteria, *J. O. Falkinham III*
5. Environments providing favourable conditions for the multiplication and transmission of mycobacteria, *I. Pavlik, J. O. Falkinham III, J. Kazda*
6. The occurrence of pathogenic and potentially pathogenic mycobacteria in animals and the role of the environment in the spread of infection, *I. Pavlik, J. O. Falkinham III*
7. Transmission of mycobacteria from the environment to susceptible hosts, *I. Pavlik, J. O. Falkinham III, J. Kazda*
8. Biological role of mycobacteria in the environment, *J. Kazda, J. O. Falkinham III*
9. Key research issues, *J. Kazda, I. Pavlik, J. O. Falkinham III, K. Hruska*
10: Photographs, *I. Pavlik. K. Hruska*
11. Index

New Book Alert

If you would like to receive information on new books in the subject area of **Microbial Ecology**, please register:

E-mail

Retype E-mail

GO

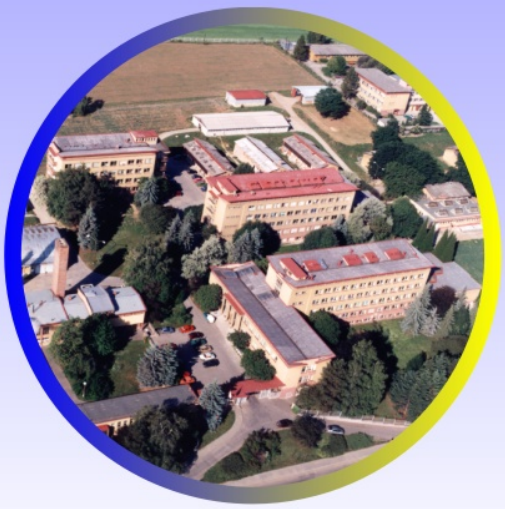

Published by
Výzkumný ústav veterinárního lékařství, v.v.i.
Veterinary Research Institute
Hudcova 70
621 00 Brno
Czech Republic
Tel. +4205 3333 2501
e-mail: centaur@vri, http://www.vri.cz
