# Supplementary material for: Long History of Queries about Bovine Paratuberculosis as a Risk Factor for Human Health
Source: Pathogens. 2021 Oct 28;10(11):1394. doi: 10.3390/pathogens10111394 (PMC8622788; doi:10.3390/pathogens10111394)
Supplement: Supplementary file 1 [file pathogens-10-01394-s001.zip › HRUSKA Publ excerpts.pdf]

PathogenCombat
for safe food

Bacterial triggers in the etiology of Crohn's disease and other autoimmune and autoinflammatory diseases

PUBLICATIONS EXCERPTS

Karel Hruška and Ivo Pavlík (Eds)
Veterinary Research Institute, Brno, Czech Republic

Moderated round table discussion to be held by the
PathogenCombat integrated research project
of the European Union 6th Frame Programme

OIE Reference Laboratory for Paratuberculosis
Veterinary Research Institute, Brno

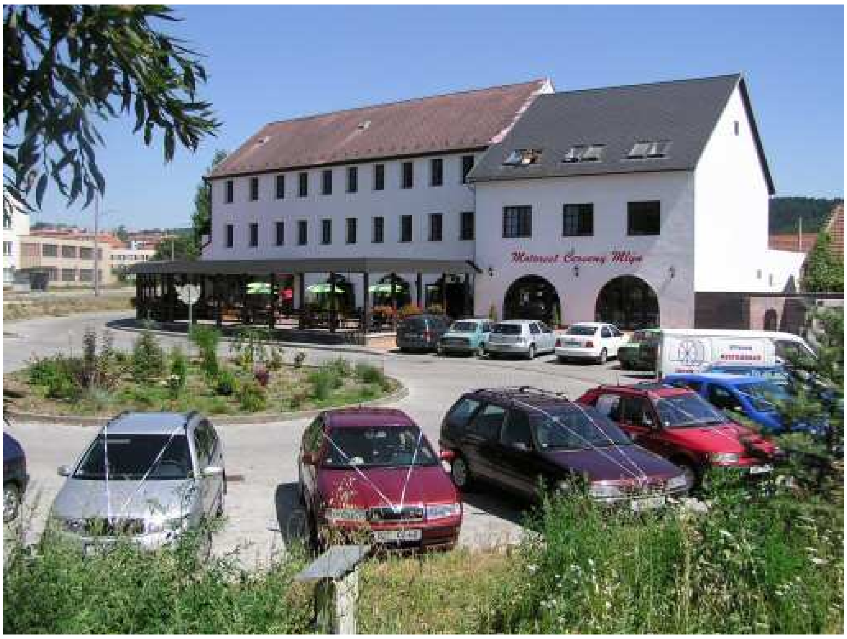

Brno (Tišnov), Czech Republic, 14-15 May, 2009

Electronic bulletin
CENTAUR NEWSLETTER FLASH INFORMATION
Vol. 13, 2009, Supplementum 1
ISSN 1213-368X

CONTENTS

EDITORS' COMMENTS 3
Participants in the discussion are requested
The contributions presented during the discussion
The published data and offered hypothesis should be discussed, taking into consideration the following principles
Publication excerpts style
The facts
The hypotheses
A call to action

INTRODUCTION 7

PART ONE
CROHN'S DISEASE 11

PART TWO
PARATUBERCULOSIS (JOHNE'S DISEASE) 19

PART THREE
AUTOIMMUNE AND AUTOINFLAMMATORY DISEASES 31

PART FOUR
MYCOBACTERIA IN WATER, BIOFILMS AND AEROSOLS 36

PART FIVE
CROHN'S DISEASE AND BREASTFEEDING 40

PART SIX
MUCOSAL IMMUNITY 44

REFERENCES 50

EDITORS' COMMENTS

Participants in the discussion are requested

- To assess the facts as acceptable, to suggest deletion from the list or to suggest new ones
- To formulate more facts
- To change the hypothesis to the fact if there is enough knowledge already available
- To suggest new hypotheses
- To recommend key research to assess the hypotheses
- To recommend measures to decrease the health risk for consumers
- To recommend how to decrease the economic loses in the dairy industry

The contributions presented during the discussion

- Will be requested by organizers immediately after the session as the written, authorized, concise text, to be published in the Proceedings
- Short summaries of the presented contributions can be written by hand, however a PC will be available in the meeting room and the electronic version is preferred
- It is recommended to prepare extensive contributions or powerpoint presentations in advance
- Comments and contributions of registered but absent participants will be available during the discussion and published in the Proceedings; they can be sent as e-mail attachments to the address centaur@vri.cz with the deadline Thursday 14 May 2009, 12:00 h

The published data and opened hypothesis should be discussed taking into consideration the following principles

- The Koch postulates are not applicable to all chronic diseases triggered by bacteria.
- The pathogenesis of CD need not have a single etiology.
- Cell wall components can trigger some pathways unless the cells are alive or replicate in the target tissues.
- Bacterial triggers can prime the changes a long time before the clinical form of disease appears.
- Bacteria themselves need not be present in tissues when clinical disease develops or at times of surgical intervention.
- Bacteria or bacterial triggers can be found in tissues of healthy persons, who are resistant to them, who do not have the necessary genetic disposition or who develop the disease in subsequent months or years.
- Not all anamnestic data are known for every patient, or have not yet been inquired, e.g. breastfeeding, drinking water contamination, preterm birth, intrauterine infection, early postnatal infection, infant swimming, etc.
- MAP from milk and mycobacteria from water can be ingested in the first days after birth when the changes in innate and adaptive immunity are intensive.
- Peptidoglycans and muramylpeptides need not originate from MAP or other mycobacteria only.
- Autoimmune and autoinflammatory diseases have a strong genetic and environmental factors and their impact need not be dose dependent.

8859 (McGonagle and McDermott, 2006)
Generic Definition of Autoimmunity
Self-directed inflammation, whereby aberrant dendritic cell, B and T cell, responses in primary and secondary lymphoid organs lead to breaking of tolerance, with development of immune reactivity towards native antigens. The adaptive immune response plays the predominant role in the eventual clinical expression of disease. Organ-specific autoantibodies may predate clinical disease expression by years and manifest before target organ damage is discernible.
Proposal for a Definition of Autoinflammation
Self-directed inflammation, whereby local factors at sites predisposed to disease lead to activation of innate immune cells, including macrophages and neutrophils, with resultant target tissue damage. For example, disturbed homeostasis of canonical cytokine cascades (as in the periodic fevers), aberrant bacterial sensing (as in Crohn disease), and tissue microdamage predispose one to sitespecific infl ammation that is independent of adaptive immune responses.

PUBLICATION EXCERPTS

- Do not list all papers published: they can not substitute for a comprehensive review article and should represent only the basic data for discussion.
- Sources are cited before the paragraph and listed at the end.
- Text has been extracted from the cited paper.
- Key words or sentences in red have been marked by the editor.
- Citations within the text that are not in References should be identified in the cited paper.
- Editor's comments have been highlighted.

THE FACTS

- Paratuberculosis (Johne's disease) is an animal, namely ruminant, disease with known etiology but very unusual pathogenesis, several years of latency period, irregular immune response and irregular shedding of mycobacteria in feces and milk.
- Contamination of milk, meat, dairy and beef products with *Mycobacterium avium* subsp. *paratuberculosis* (MAP) is not a reason for recall from the market according to current hygienic norms.
- MAP culture requires weeks or months and can deliver false negative results.
- Specific DNA is reliable evidence for MAP contamination.
- Paratuberculosis is a herd associated disease: MAP demonstrated in bulk tank milk, milk filters or manure gives evidence of MAP shedding animals being present in the herd in the past, the present and probably in the future, if strict measures are not applied.
- Paratuberculosis is considered a potential zoonosis.
- Control and eradication of paratuberculosis in cattle and sheep needs time, large investments and the ability of farmers to collaborate.
- Crohn's disease (CD) is a chronic idiopathic inflammatory bowel disease in humans, similar in many features to bovine paratuberculosis and involves an aberrant mucosal immune response in genetically susceptible individuals.
- Mutations in the NOD2 gene, located on chromosome 16q12, have been shown to be associated with CD.
- CD is not a common infectious disease or food born disease.
- CD is currently classified as autoimmune or autoinflammatory disease.
- Koch's postulates cannot be seen as a necessary tool for proving causative links between microbes and some (autoimmune) diseases.
- CD and another inflammatory bowel disease, ulcerative colitis, are not the same disease.
- *Mycobacterium avium* subsp. *paratuberculosis* (MAP) is one of the possible factors in the etiology of CD.
- MAP, namely its cell wall components, can trigger bowel inflammation.

- Peptidoglycan degradation products, either released by the bacteria or processed by the host cell in the lysosomal compartment, are critical in defining the host response to bacterial infection.
- MAP is an ubiquitous organism, shed by diseased animals in enormous numbers in feces and milk and present in meat, milk and dairy products.
- MAP is a resistant microorganism and pasteurization and insufficient cooking do not ensure its inactivation.
- Macrophages that have ingested heat inactivated MAP stimulate T-cells, and induce higher expression of pro-inflammatory cytokines.
- Pasteurization and cooking do not destroy the cell wall components of MAP.
- MAP is a slowly growing organism in culture.
- Differences in innate and adaptive immunity are evident in convential and gnotobiotic animals.
- Intestinal microbiota and their components play a crucial role in the development of the mucosal immune system.
- Breastfeeding is protective against autoimmune diseases.
- Influencing the naive intestinal immune system at a very early stage, can present risk factors for the individual predisposition of developing IBD later in life.
- MAP and other mycobacteria contaminate water in municipal distribution systems, swimming pools, rivers and lakes.
- M. avium was found to form biofilms, especially in water pipe systems of large cities.
- Both CD and paratuberculosis in farm animals are economically important diseases.

THE HYPOTHESES

- Intrauterine infection with MAP or ingestion of milk or water, contaminated with MAP or its cell components during the first days or weeks after birth can sensitize cells involved in the innate or adaptive immunity and clinical form of CD can develop after years due to infection or another trigger.
- MAP can survive in some CD sufferers and can induce inflammation in different parts of the intestinal tract.
- MAP ingested or inhaled, even as dead cells, can stimulate proinflammatory cytokines, namely in persons with NOD2 mutations
- A significant association found between the month of birth and later development of IBD can be associated with a possible higher contamination of tap water with mycobacteria in summer.
- The number of MAA or MAP in tap water and milk fluctuates but can reach values of 10^4 g^{-1}.
- Infant swimming poses a risk of ingestion or inhalation of mycobacteria.
- The global market for baby foods and dairy products may be coincident with increasing CD prevalence in countries which in the past were nearly free from this disease.
- A reliable and simple test can be developed for mycobacteria determination in bulk tank milk and in milk filters.
- A reliable and simple test can be developed for mycobacteria determination in water (tap and bottled drinking water, indoor swimming pool water).
- A reliable and simple test can be developed for mycobacteria determination in meat of slaughtered animals.

A CALL TO ACTION

As physicians and scientists who have been personally affected by Crohn's Disease, we write with a sense of urgency. We do not simply care for patients with this illness. Each of us suffers from it, or has an intimate family member who does so. We therefore find it both exciting and frustrating that growing evidence points to a microbial, infectious cause of Crohn's Disease: exciting because genuine prevention and cure (as opposed to mere palliation) may be achievable, but frustrating because the public as well as the medical community remains largely unaware of this possibility, while hundreds of thousands of people, many of them children, suffer from the illness itself or iatrogenic complications.
....

It has not escaped our notice that the proposed MAP-based etiology for Crohn's will not only have substantial implications for public health, but also important economic, political, and psychological consequences, given that 7-55% of dairy herds in Western Europe and North America appear to be infected with MAP.13 Thus, we anticipate that the emerging pathogenic explanation of Crohn's will encounter considerable resistance, from industry as well as government. The situation is remarkably parallel to that experienced by Nobel Prize recipients Drs. Barry Marshall and Robin Warren, who demonstrated a bacterial cause of gastric ulcers, but whose work was strongly opposed by the medical and pharmaceutical establishment. Given heightened awareness of zoonotic infections, as evidenced by concern about a possible avian influenza pandemic, we are cautiously optimistic that in this case, the medical profession as well as the public will be amenable to our message. We endorse the precautionary principle: when there is a significant threat of harm to human health or the environment, precautionary measures should be undertaken promptly even in the absence of indisputable proof. If even only a proportion of Crohn's disease cases are caused by infection with MAP, action should be taken to protect public health from this pathogen and to establish effective treatment regimes.
....

A scientific revolution is underway, implicating an infectious agent, namely Mycobacterium avium paratuberculosis (MAP) in the etiology of Crohn's disease. We think compelling evidence suggests that Crohn's Disease can be caused by a zoonotic infection that could be prevented with sensible public health measures and improved farming practices. There are, moreover, exciting treatment options for eradication of the bacterium as well as for the enhancement of appropriate host responses to infection. It is socially as well as scientifically irresponsible to ignore or minimize this problem. We, as medical professionals, can and must lead the way toward preventing and curing Crohn's Disease.

Judith Eve Lipton, M.D., J. Todd Kuenstner, M.D., M.S., David Barash, Ph.D., and James Biesecker M.D., Ph.D.

Please, humbly read the full call. 8881 (Lipton et al., 2009)

INTRODUCTION

The database Web of Science has 32 253 records of papers on Crohn's disease (CD) from 1953; 2809 (8.7%) papers were published in 2008. Dalziel (1913) was the first who has suggested a link between MAP and Crohn's disease, a chronic inflammatory bowel disease in humans. Since then, the etiology of the disease has been disputed (0406 (Herthnek et al., 2008). Many authors call attention to link of *Mycobacterium avium* subsp. *paratuberculosis* (MAP) to CD. However, paratuberculosis (Johne's disease, PTB) is not yet assumed to be public health risk.

Publications in the Web of Science database have been sporadic from 1945 with a moderate increase from 1965 and a dramatic increase from the beginning of nineties. The index 2007/1990 for paratuberculosis, Crohn's disease and both key words is 6.0, 8.0 and 6.8, respectively. The increasing importance of paratuberculosis and Crohn's disease, and new knowledge available in the past two decades, are evident.

0428 (Behr, 2008)
In a recent essay on the emergence of infectious diseases, Jared Diamond predicted that future pandemics are likely to likely to come from genera that have already marked the history of mankind [26]. In this vein, mycobacteria have delivered twice before, with leprosy and TB. While efforts targeted against TB are clearly warranted, we should not be surprised if another pathogenic mycobacterium affects mankind on a major scale, most likely following effective control of TB. Rather than contemplating human existence without mycobacterial infection, we may have to choose a preferred mycobacterial species, potentially a live attenuated vaccine, to ward off the consequences of infection with others.

8856 (Economou and Pappas, 2008)
...... epidemiologic studies are of paramount importance in investigating disease etiology: A burst of scientific literature on CD incidence recently observed has mostly supported the idea of a disease of the developed world, with a typical north–south European gradient. The reasoning behind this incidence has been inconsistent; most studies have been localized, often retrospective, and thus subject to inadequate data collection, usually covering limited time periods. Yet, the major existing cohort studies indicate a significant CD incidence rise in the second half of the 20th century. .... CD is definitely emerging worldwide as a major public health threat. The increasing reports of pediatric disease further underline this threat. Changes in lifestyle, at the regional, national, or international level, seem to play an etiologic role in the increasing incidence of the disease: Whether these factors are pure exogenous triggers or part of an exogenous– endogenous immune chain we still do not know. Seventy-five years after Crohn's characterization as a unique entity, we are still in the dark. .... whatever the actual effect imposed by environmental triggers in CD incidence, this effect would require a latent period for disease pathogenesis to evolve and clinical presentation and diagnosis to be made. Even if bacteria or diet were implicated, their pathogenetic effect would require protracted exposure over time, and thus the changes in socioeconomic status should actually precede the changes in CD incidence by a period of 5–10 years at least. .... What unites Canterbury in New Zealand, Nova Scotia and Manitoba in Canada, Amiens in France, Maastricht in the Netherlands, Stockholm in Sweden, and Minnesota in the US (apart from the existence of scientists alert enough to reveal the evolving epidemiologic trends)? Unlocking this strange union would subsequently unlock the mystery of Crohn's etiology, still speculated upon 75 years after its baptism.

8876 (Anon., 2000) **EC Report conclusions (part only)**
Mycobacterium avium subsp. paratuberculosis (Map) is an organism which can cause chronic inflammation of the intestine (Johne's disease) in cattle, sheep and goats as well as in other animals including rabbits and macaques. Map infections are widespread in domestic livestock, including cattle, sheep and goats. Rabbits, deer and other animals have been identified as wildlife hosts of Map. Animals can harbour Map without showing signs of disease or reacting to serological tests. Map is found in large quantities in the intestine of diseased animals and in lesser amounts in lymph nodes, liver, spleen and other tissues. Clinically infected animals may shed up to 10^{12} Map per ml in their faeces. Subclinically infected animals also shed the organism though usually in lower amounts.

Infected dairy cows and sheep shed Map in their milk. .... Map is more robust than M. tuberculosis and M. bovis. The complete destruction of all viable Map in milk by pasteurisation at 65℃ for 30

minutes or 72℃ for 15 seconds, may not be assured. Viable Map has been identified in pasteurised milk supplies. There is preliminary evidence that Map may be present in human milk. If these initial results are confirmed, this would be a significant finding. .... Facilitating the creation and maintenance of a network of researchers at EU level combining expertise in chronic inflammatory bowel diseases in humans and in mycobacterium sp. infections in animals, would greatly contribute to attaining these goals.

Publications in the Web of Science database

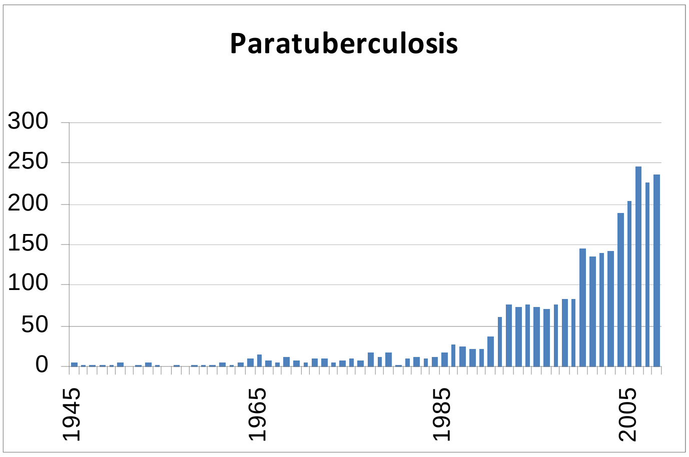

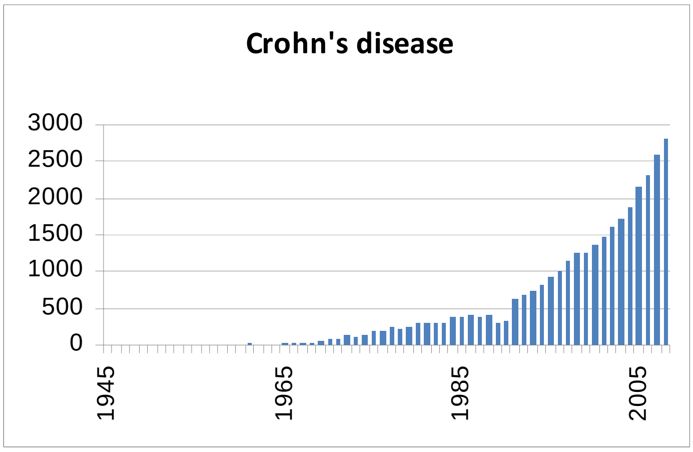

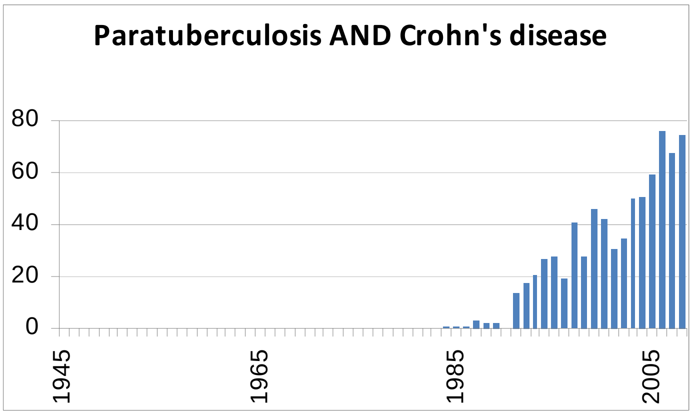

Data available from the Institute of the Health Information and Statistics of the Czech Republic, Prague, demonstrate an increase in patients suffering from Crohn's disease from 3373 in 1995 to 14 407 in 2007. At the beginning of the 1990s, trade with Western Europe was opened and cattle suffering from paratuberculosis were imported. Baby formula used until the 1980s was of domestic origin, produced from milk free from MAP. In the 1990s, the market was opened for milk and milk products, and baby foods produced in countries with paratuberculosis. In 2005, we demonstrated IS900 in 25 (49%) and f57 in 18 (35.3%) of 51 retail baby food dry products on the Czech market. Products from three of eight producers with the highest number of products in the tested panel were positive for IS900 in 41.7%, 45.5% and 85.7% 0521 (Hruska et al., 2005). An increase in paratuberculosis in slaughtered cattle in the Czech Republic has been published by 2691 (Vecerek et al., 2003), who reported indexes 1999-2002/1995-1998 of paratuberculosis in slaughtered cattle as 4.62 and 2.92 for condemned and conditionally edible carcases, respectively.

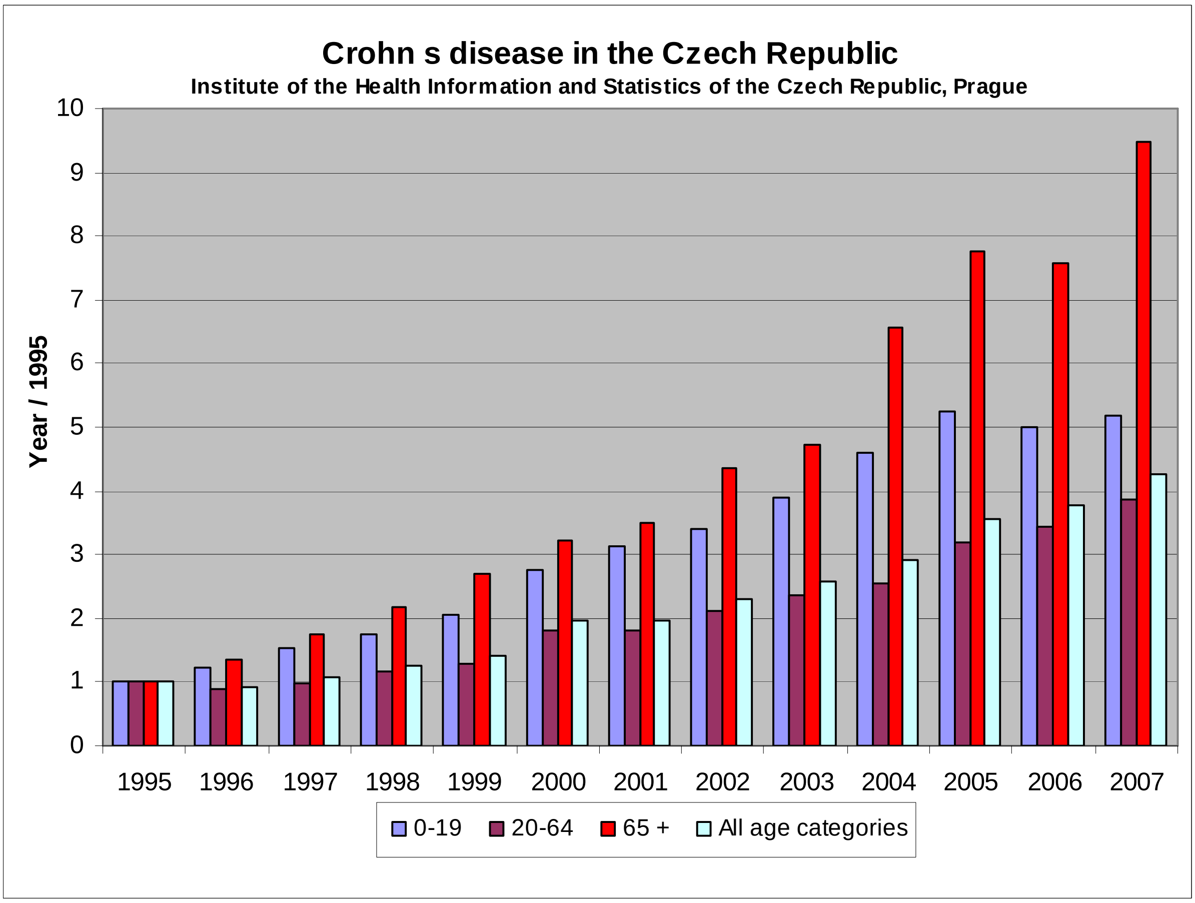

PART ONE
CROHN'S DISEASE
Incidence, etiology, pathogenesis, diagnosis, therapy, epidemiology, social factors

8877 (Chiodini, 1989)
While no firm evidence clearly identifies mycobacteria as an etiologic agent (in Crihn's disease), the notion is supported by suggestive and circumstantial data and by remarkable similarities to other known mycobacterial diseases. A consensus could probably be reached on the notion that, if the etiology of Crohn's disease is microbial in origin, it is most likely mycobacterial. ...... If Crohn's disease has a mycobacterial etiology, the most likely agent would be M. paratuberculosis. This organism is unique among the mycobacteria because the gastrointestinal tract is the only environment in which it can replicate in vivo. ..... Mycobacterial diseases are essentially immunologically mediated disorders; therefore, each species responds differently immunologically and identical pathologic diseases would not be expected. For example, M. paratuberculosis infection in cattle does not produce caseation necrosis, but 25% of goats develop caseating granulomas in response to infection with M. paratuberculosis (52). Humans are likely to respond differently. **Published in 1989.**

8875 (Nacy and Buckley, 2008) **A report from the American Academy of Microbiology**
One acknowledged potential microbial agent of Crohn's disease is *Mycobacterium avium* subspecies *paratuberculosis* (MAP), a microorganism that causes a gastrointestinal disease similar to CD in ruminants, including dairy cattle, called Johne's disease (or paratuberculosis). People with CD have 7:1 odds of having a documented presence of MAP in blood or gut tissues than those who do not have CD, thus the association of MAP and CD is no longer in question (see Figure 1, page 11). The critical issue today is not whether MAP is associated with CD, but whether MAP causes CD or is only incidentally present, not an inciter or participant in the disease process. .... The general consensus of the assembled experts was that there are certainly reasons to suspect a role for MAP in CD:

- MAP persists in contaminated soil and water, which links the environmental factor of CD to the disease.
- MAP has a cell wall that contains muramyl dipeptide (MDP). One genetic trait that is affiliated in certain patients with CD is the NOD2 gene, which regulates ability to respond appropriately to MDP, thus the link between the genetic trait and MAP, or other bacteria.
- MAP causes Johne's disease, an illness of cattle and other ruminants that has many similarities with CD. The similarities of MAP disease in animals, for which the etiologic agent is known, and CD, for which the etiologic agent is unknown, provide a symptomatic link between agent and disease.
- MAP can survive standard milk pasteurization processes and has been identified in off-the-shelf milk in retail grocery stores in the U.S. and the European Union (E.U.). There is increasing concern that MAP can also be found in cheese made from the milk of MAP-infected cattle and meat from Johne's diseased animals. These observations could provide the exposure route of CD patients to MAP.
- Treatment of some CD patients with antibiotics that have activity on certain other Mycobacteria, although not specifically selected for their activity against MAP, provides short-term or long-term relief or remission of symptoms.

.... As of 2001, the Centers for Disease Control and Prevention estimated that over 500,000 people in the U.S. are living with CD, and more recent statistics show that 800,000 people in North America and 1 in 1000 persons in westernized countries have diagnosed CD. In the U.S., roughly 50% of CD patients are children. There is currently no cure for CD. .... Mutations in the NOD2 gene found in CD patients disrupt the ability of immune cells to recognize certain signals that are present in pathogenic bacteria. But it is also clear that heredity is not the full story, nor is an overactive immune system. The inflammation of CD may be due to a malfunction of the immune system that is infectious in origin, genetically controlled, and environmentally exacerbated. The incidence of CD has been rising over the last several decades, as observed in multiple countries by independent investigators. There is an association between rising economic conditions and increasing reports of CD in a population. This has

been interpreted as evidence that an environmental trigger is as, if not more, important in the etiology of CD as human genetics. .... Johne's disease is a $1.5 billion a year cattle industry problem in the

U.S. Johne's disease is found in livestock herds around the globe, and though the condition is easy to identify once symptoms arise, it is challenging to diagnose in the early stages of infection. Healthy but infected animals transmit the organism through milk and manure, invisibly spreading the infection to young animals. As a result of this silent transmission, it is difficult to eliminate MAP from individual herds.

0110 (Marks and Segal, 2008)
Crohn's disease arises from a defective interaction between the highly concentrated mass of bacteria in the gastrointestinal tract and the underlying tissues. It has generally been believed to result from an excessively exuberant inflammatory response or from 'autoimmunity'. Recent evidence has emerged that the problem is instead a failure of the way in which the body responds to the penetration of bacteria and other bowel contents through the intestinal mucosal barrier. Rather than Crohn's disease being caused by excessive inflammation, the primary mechanism is actually that of an immunodeficiency. Failure of inflammatory mediator production leads to insufficient recruitment of neutrophils, resulting in inadequate removal of bacteria and other debris. This impairment of acute inflammation can be compensated in some circumstances by signalling through NOD2. If not cleared, the foreign material in the bowel wall is taken up within macrophages, eliciting a granulomatous reaction and the local and systemic sequelae so characteristic of Crohn's disease. .... Granulomatous inflammation is the hallmark of failure of the cellular inflammatory response to remove foreign material. This may result because it is indigestible (as illustrated by berylliosis or silicosis) because an infective agent evades immune eradication (as seen in tuberculosis), or in cases in which the digestive function of neutrophils is defective. The latter include congenital genetic disorders, such as chronic granulomatous disease (CGD) [48] and glycogen storage disease-Ib (GSD-Ib) [49], in which molecular lesions impair the ability of neutrophils, not only to kill bacteria but also to digest phagocytosed contents. In all the above examples, the exogenous agents are subsequently enveloped and contained within macrophages, which progress to form giant cells and granulomata. .... Thus, the development of Crohn's lesions would require a 'two-hit' pathogenesis, the first being an inherently impaired acute inflammatory response and the second a defect in one or other compensatory pathways (Figure 2). **In Fig. 2 attention should be paid to "Inherently Weak": the wekness could be acquired as a result of epigenetic reprogramming 8879.**

8865 (Phavichitr et al., 2003)
Victoria, Australia: Incidence rates were analyzed in 5-year blocks and expressed per 100 000 children aged 16 years or less. The incidence of Crohn's disease increased by more than 15-fold over the 30-year period .... The increase that we have identified, however, has occurred 1–2 decades later than in these northern European studies. .... Our data, however, emphasize that because the prevalence of the gene is likely to be static, there must be a changing environmental factor which plays a major role in precipitating the clinical illness. There are at present no answers as to the identity of this factor, although interest currently focuses on luminal bacterial flora. .... Significantly more of our patients came from an urban population than would be expected. ..... Further studies are clearly needed to examine this urban predominance, given the implications this may have for an etiology of this condition. It is of interest that an Australia-wide database of children with inflammatory bowel disease has identified a marked variability between different states in the types and distribution of inflammatory bowel disease. Crohn's disease has important implications for an individual's quality of life and also places a heavy load on health care facilities. The increasing costs involved in the therapy of this condition with such drugs as infliximab, highlight a clear need for ongoing prospective surveillance mechanisms in both children and adults.

8866 (Green et al., 2006a)
Incidences of Crohn's disease and ulcerative colitis were observed to be highest among non-Aboriginal persons, persons of high socioeconomic status, persons with the lowest rates of enteric infection, and persons with the highest rates of multiple sclerosis. The observation of an inverse association between IBD incidence and rates of reportable enteric infection at the population level is

consistent with the ‘‘hygiene hypothesis,’’ which suggests that early exposure to enteric agents affords protection against eventual development of IBD. The positive association between IBD incidence rates and multiple sclerosis suggests that these two chronic, immunologically mediated diseases may have a common environmental etiology. This study underscores the importance of environment in IBD causation.

8867 (Moayyedi, 2006) (Invited commentary to 8866 Green et al., 2006)
The hygiene hypothesis was first proposed in 1989 as a mechanism to explain the increase in allergic diseases seen in industrialized nations. The hypothesis holds that exposure to foreign antigens causes activation of T helper cells (subdivided into types 1 (Th1) and 2 (Th2)), which in turn causes inflammation. This is a major mechanism by which the body combats infection, but especially there may be faulty regulation of this Th1/Th2 system if there is lack of early childhood exposure to a variety of infections and other antigens, in the genetically susceptible. This could lead to excess activity of the Th2 system, which is important in many atopic conditions such eczema, asthma, and food allergy, or overexpression of the Th1 system, which is important in Crohn’s disease and multiple sclerosis. This hypothesis is plausible and is supported by immunologic data, but epidemiologic evidence of an association between a ‘‘hygienic’’ environment in early childhood and disease in later life is sparse and contradictory. The main problem with the paper by Green et al. is that it evaluates exposure to a ‘‘hygienic’’ environment in the recent past and not in early childhood........... human beings could be viewed simply as receptacles for our intestinal bacterial flora. Usually this coexistence is mutually beneficial, but subtle changes in gastrointestinal bacterial flora or exposure to mild pathogens may disturb immune function and lead to gastrointestinal, autoimmune, or allergic diseases. .... As a gastroenterologist, I am particularly interested in the hypothesis that the gastrointestinal environment is important in the etiology of many immunologically mediated diseases. Infections such as cholera produce dramatic immediate effects and therefore are comparatively easy to study. Assessing the impact of subtle changes in gastrointestinal flora that may manifest as disease later in life is much more of a challenge, particularly since there are 10^{12} bacteria per gram of colonic contents. The creation of increasingly sophisticated databases with which to characterize the environment we live in and analysis of associations with a variety of diseases will provide valuable clues to the etiology of diseases, including those that might be caused by changes in gastrointestinal flora. The Winnipeg investigators are to be congratulated on having the vision and dedication to create such a database in Manitoba. The challenge is for others to do the same so that we can obtain a more global picture of how environment may influence disease. The notion that enteric infections may cause or contribute to a variety of immunologic diseases might seem fanciful, but so did John Snow’s theories of cholera transmission in the 1850s. His mapping techniques had many deficiencies, but ultimately they led to the discovery of the infectious agent. GIS also has many weaknesses, but it too may play a vital role in our future understanding of how subtle changes in the environment cause disease. **The global market can offer MAP contaminated baby foods in countries free of paratuberculosis or even of cattle and sheep as well as water pipe systems can be diferently contaminated with respect of location and time.**

8868 (Green et al., 2006b) (Respond to 8867 Moayyedi, 2006)
Bernstein et al. found statistically significant associations between subjects with ulcerative colitis and Crohn’s disease and a number of chronic immunoinflammatory diseases. Thus, while these autoimmune diseases do cluster on an individual basis, we hope to conduct further ecologic studies in search of associations worth pursuing on an individual basis. We fully agree with Dr. Moayyedi (1) that the pursuit of the etiology of inflammatory bowel disease and the strength of the associations we have reported could be enhanced greatly by similar studies from other jurisdictions.

8871 (Bernstein et al., 2006)
Approximately 0.5% of the Canadian population has IBD. Canada has the highest incidence and prevalence of CD yet reported.

8869 (Kugathasan et al., 2003)
This study reports the incidence of pediatric CD and UC in a large, defined, ethnically diverse North American population. Important observations identified in this evaluation of pediatric IBD across

Wisconsin include (1) the highest pediatric IBD incidence reported in the world to date, (2) a twofold predominance in pediatric CD incidence compared with UC, (3) a significantly higher rate of

CD diagnosis among boys compared with girls, (4) a low frequency of patients with positive family histories, (5) no modulatory effect of urbanization on pediatric IBD incidence, (6) equal distribution of IBD incidence among all ethnic populations, and (7) the initial presentation of pancolitis among the majority of newly diagnosed children with UC. .... The increased pediatric incidence does not appear to result from an earlier diagnosis of children with IBD who previously were undiagnosed until adulthood, because there are no signs of a corresponding decrease in the incidence in the adult

population. .... The most important factor identified to date that confers risk for the development of IBD is a positive family history. Compared with the general population, there is a several-fold increased risk of developing either CD or UC in the setting of a positive family history with affected firstdegree relatives. Previous studies have shown that the pediatric onset of CD is associated with a 30% likelihood of having affected relatives. In our study, only 11% of children diagnosed with IBD had a family history of IBD. Because our study focused on incidence data in newly diagnosed children, there is the possibility that additional family members may develop IBD in the future. The relatively low rates of affected family members suggest that the incidence of IBD may be increasing in populations that were not previously at high risk. Past studies have shown a high incidence of IBD in Caucasian children, but we did not observe any differences across racial groups, including minority groups previously thought to be at lower risk for disease. However, none of the non-Caucasian children with IBD had family histories of IBD. These data, in conjunction with our equivalent incidence rates between sparsely and densely populated areas of the state, suggest that IBD is now a condition equally afflicting all populations within our region. .... In summary, our study provides new epidemiologic data regarding the incidence and characteristics of pediatric IBD within a well defined population. Overall, the results of this study suggest that the clinical spectrum of IBD continues to evolve, because pediatric IBD now afflicts all races and ethnicities as well as rural and urban populations with a similar frequency, pointing to changing environmental factors as increasingly important in the pathogenesis of IBD.

0423 (Lowe et al., 2008)
Crohn's disease was thought to be an autoimmune disease; however, a series of independent observations from a number of groups point instead to Crohn's disease as an impaired or dysregulated host response to microbial contributors. ..... Therefore, it is essential to not only identify the microbiota associated with disease but also def ne their role in disease pathogenesis. ...... the aetiology of Crohn's disease currently represents the converse challenge; genetic studies are uncovering the molecular basis of susceptibility but there is no consensus on the organisms that exploit these defects. An example of such a predisposition in Crohn's disease is nucleotidebinding-oligomerisation-domain 2 (NOD2) deficiency, where cellular studies of the NOD2 frame-shift mutation have shown a loss of bacterial peptidoglycan recognition. .... So too, one hopes that finding novel associations between microbial species and chronic diseases such as Crohn's disease will serve as the basis for interventional studies in patients, and preventive studies in those at risk. Crohn's disease is common, with an estimated prevalence in parts of Canada of 270 per 100 000 population,60 so there are large numbers of patients who might benefit from treatment. Moreover, NOD2 variants associated with Crohn's disease are found in an estimated 1–4% of healthy white people, suggesting that there is a large pool of susceptible individuals who might benefi t from appropriate preventive measures. ..... A final consideration is whether one can reasonably expect that just one organism is able, on its own, to produce the myriad of signs and symptoms associated with Crohn's disease. .... One potential explanation for the difficulty in assigning causality in Crohn's disease to one organism is that more than one organism is involved. ..... it was reported that mycobacterial mannan can impair phagocytic capacity of macrophages, leading to substantially increased survival of E coli. **Attention should be also paid to the period of life when the immunological mechanisms can be affected although they role is activated after years by the same or similar trigger.**

0005 (Shanahan, 2002) **Very important and interesting paper**
Few disorders in clinical medicine are associated with as much chronic morbidity as Crohn's disease. The disorder is characterised by transmural inflammation that could affect any part of the

gastrointestinal tract, and the disease relapses and remits throughout its course. For many patients, Crohn's disease imposes a substantial personal burden, with unpredictable fluctuating symptoms, time off work, need for expensive drugs, or surgery and multidisciplinary care. With a prevalence of about 0·1% in many developed countries Crohn's disease also places a major burden on public health-care resources. Some investigators have argued that the Crohn's disease and ulcerative colitis are at opposite ends of a continuous range of disease, but more and more evidence suggests that they are distinct disorders that share some genetic and environmental risk factors and differ in others. .... striking increase in frequency of Crohn's disease within the more-developed world over the past 50 years, and the disease's increased recognition with progressive industrialisation in less-developed countries. .... Thus, although subsets of Crohn's disease could involve distinct pathogenic mechanisms tissue damage in most patients with Crohn's disease can be accounted for by the

downstream effects of activated TH1 cells. TH1 cell differentiation takes place when T cells interact with antigen-presenting cells that produce IL-12 in response to exposure to bacteria. The main mediators of tissue destruction are matrix metalloproteinases, which are generated in response to release of TH1 cytokines. The TH1 cytokine milieu is associated with changed signalling from regulatory T cells and increased resistance of T-effector cells to apoptosis—leading to perpetuation of the inflammatory process (figures 1 and 2). ...... Comprehensive management of Crohn's disease needs to address both the host response and the environmental factors driving the inflammatory process. Endogenous modifiers of disease activity such as the effect of the braingut axis and psychological stress also need to be investigated. ..... Stress-induced reactivation of disease is dependent on T cells and can be adoptively transferred.

8836 (Pedley et al., 2004)
The isolated case of Iceland .... Prior to 1930 MAP infection and JD in Iceland were virtually unknown. Then in 1933, 20 Karakul sheep were imported from Germany and, after quarantine, were distributed to 14 farms (Fridriksdottir et al. 2000). Although apparently healthy, some of the Karakul sheep were subclinically infected with MAP. They transmitted MAP to the Icelandic sheep population though they never developed disease themselves. By 1938 clinical JD appeared in Icelandic sheep on five of the original farms. By about 1945, clinical JD was in the cattle on the same farms, although infection in the cattle was difficult to diagnose as the organisms would not grow in culture; a characteristic of sheep MAP strains. The organism from these cattle was later confirmed as the sheep strain of MAP by IS1311 restriction endonuclease analysis (Whittington et al. 2001b). Slowly the infection spread so that by the late 1950s the disease was epidemic with about 30% of sheep farms affected and huge annual losses. The mean incidence of CD (number of cases/105 per year) in the human population was 0.4 from 1950-59, 0.45 from 1960-69, 0.9 from 1970-79, 3.1 from 1980-89 and 5.6 from 1990-94 inclusive, the highest annual figure over this last five-year period being 8.2 in 1992. Young people were particularly affected (Bjornsson 1989; Bjornsson et al. 1998; Bjornsson & Johannsson 2000).

0437 (Asakura et al., 2008)
An analysis of Japanese epidemiological data suggested that the registered number of patients with CD or UC started to increase more than 20 years after an increased daily consumption of dietary animal meat and fats, and milk and dairy products, and after a decreased consumption of rice. Many studies implied a positive role of intestinal microbes in the occurrence of IBD. Intestinal environmental factors, such as Westernized food and intestinal microbes, seem to be involved in the increased occurrence of IBD.

8808 (Hugot et al., 2001)
NOD2 activates nuclear factor NF-kB; this activating function is regulated by the carboxy-terminal leucine-rich repeat domain, which has an inhibitory role and also acts as an intracellular receptor for components of microbial pathogens. These observations suggest that the NOD2 gene product confers susceptibility to Crohn's disease by altering the recognition of these components and/or by over-activating NF-kB in monocytes, thus documenting a molecular model for the pathogenic mechanism of Crohn's disease.

0497 (van Lierop et al., 2009)
Microbial muramyl dipeptide, the smallest bioactive component of peptidoglycan, a component of all bacteria (67,68), has been identified as the specific ligand for NOD2. Different mutations in the NOD2 gene, located on chromosome 16q12, have been shown to be associated with CD. Specifically, NOD2 mutations are thought to alter the susceptibility and location of the disease (2,3,69–71). Recently, a third class of PRRs has been identified: the inflammasome. The inflammasome is an intracellular protein complex that initiates the release of caspase-1. In concert with NF-kB phosphorylation upon TLR signaling, activation of the inflammasome results in secretion of the proinflammatory cytokines interleukin (IL)-1b and IL-18 (72,73). ..... A strong case for enhanced PRR signaling in IBD was offered by research performed in NOD2-deficient mice. Microbial peptidoglycan can be recognized by the extracellular receptor TLR2 and by the intracellular NOD2 protein. When macrophages derived from NOD2- deficient mice were stimulated with peptidoglycan, the result was an overexpression of proinflammatory cytokines compared with wild-type macrophages (133). In humans, it was confirmed that in the absence of NOD2 signaling, TLR2 activation can result in enhanced expression of

proinflammatory cytokines upon stimulation (133–136). ..... pediatric CD may represent a specific subset of patients who are incapable of maintaining a physiological state of epithelial hyporesponsiveness to microbial stimulation (138). .... CD could result from impaired production of thymic stromal lymphopoietin by the intestinal epithelial cells (164). The capacity to serve as innate cells and also as initiators of adaptive responses have emphasized the role of DCs in the immunologic cascade that contributes to both mucosal tolerance and inflammation.

8809 (Damen et al., 2006)
Buccal epithelium of children with Crohn disease is immunologically active, even in the absence of oral lesions. The enhanced chemokine production is associated with pediatric Crohn disease and appears restricted to cells derived from the epithelial barrier. Assessment of chemokine production by buccal epithelial cells may become a new, rapid, noninvasive test for screening and classification of IBD in children. Here, we demonstrate that in children with CD, buccal epithelial cells produce significantly higher levels of CXCL-8, CXCL-9, and CXCL-10, in comparison with children with UC, with controls, or with adults with CD. Interestingly, not all newly diagnosed children with CD presented with an enhanced chemokine production by buccal epithelial cells. Also, no relation was found between the disease activity (PCDAI) or a specific drug that was used and chemokine production. These findings may suggest that the enhanced chemokine production is specifically associated with a subset of patients with a specific (genetic) ethiopathogenesis. Upon stimulation with LPS or zymosan, only buccal epithelial cells derived from pediatric CD patients show an inducible production of chemokines.

0023 (Zur Lage et al., 2003)
.... there seemed to be no difference between infection of J774 with viable M. ptb and avium or treatment with heat-killed bacteria. Thus, the bacterial components that suffice to induce the required signals were not denaturable by the heat treatment and therefore were probably not proteins.

8817 (Basler et al., 2008)
Murine macrophage cell lines represent suitable systems to analyse M. avium ssp. patho-mechanisms and could show that MAP, but not MAA, specifically inhibited the antigen-specific stimulatory capacity for CD4+ T-cells. Macrophages that had ingested heat inactivated MAP could stimulate T-cells and induce higher expression of pro-inflammatory cytokines (data not shown). Taken together, our data suggest that live MAP survives in its niche by subverting the immune defence mechanisms of the host.

0006 (Lotz et al., 2007)
NOD2 was formerly believed to be expressed exclusively in monocytes (Ogura et al., 2001b) but more recent data revealed that it can be up-regulated in epithelial cells by NF-kB activation (Gutierrez et al., 2002) and thus it may be an important player in mucosal host defense. .... The presence of the dense and highly dynamic microbial flora requires mechanisms of discrimination between infection with potentially harmful microbes and commensal colonization. Inappropriate responses may lead to

inflammation, tissue destruction, and organ dysfunction such as seen in patients with the inflammatory bowel diseases, Crohn's disease and ulcerative colitis or necrotic enterocolitis of the newborn.

0260 (Brosbol-Ravnborg et al., 2009)
Pattern recognition receptors (PRRs) are an integral part of the innate immune system and govern the early control of foreign microorganisms. Single nucleotide polymorphisms (SNPs) in the intracellular pattern recognition receptor nucleotide-binding oligomerization domain-containing protein (NOD2, nucleotide oligomerization domain 2) are associated with Crohn's disease (CD). We investigated the impact of NOD2 polymorphisms on cytokine secretion and proliferation of peripheral blood mononuclear cells (PBMCs) in response to Toll-like receptor (TLR) and NOD2 ligands. .... In summary, our data suggest a significant role of GM-CSF in the pathogenesis of CD. Upon TLR-2/NOD2 stimulation, the secretion of GM-CSF was reduced in all CD patients, and was almost abolished in CD patients with NOD2 polymorphisms. This is in contrast to TNF-a secretion, which was down-regulated by NOD2,but more so in healthy individuals than in CD patients. This argues for both NOD2-dependent and NOD2-independent pathways in GM-CSF secretion. The NOD2-independent GM-CSF secretion may be mediated via p38 mitogen-activated protein kinase (MAPK) and subsequent extracellular regulated kinase (ERK-1/2) phosphorylation [42]. The mechanism by which NF-kB-independent reduced GM-CSF production may contribute to disease pathogenesis remains to be investigated, but might be related to diminished innate immune reactivity.

0008 (Basler et al., 2006)
.... the IRG1 gene represents a suitable marker gene to dissect the molecular mechanisms contributing to macrophage activation by mycobacteria. The factors contributing to IRG1 mRNA expression and mRNA stabilization in RAW264.7 macrophages, however, remain to be identified.

0014 (Stratmann et al., 2004)
..... the MptD protein is exposed on the surface of M. avium subsp. paratuberculosis during infection and is therefore a potential target for M. avium subsp. paratuberculosis immunization or treatment. These results together with the predicted functions of other genes in the 38-kb region strongly suggest its association with M. avium subsp. paratuberculosis virulence and that the 38-kb locus should be considered the first pathogenicity island identified in M. avium subsp. paratuberculosis.

0271 (Blink and Miller, 2009)
y delta T cells are most commonly known for their response to mycobacterium and their locations at mucosal sites. They have the potential to influence all levels of inflammation through rapid production of inflammatory mediators, recruitment of inflammatory cells *via* chemokines, influencing T cells differentiation by cytokine production, and/or direct killing *via* production of cytotoxic mediators. Some subsets have the capacity for promoting autoimmunity.

0235 (Hong et al., 2008)
CARD15 genes carrying the 3020insC frameshift polymorphism encode a truncated CARD15 protein that is unresponsive to bacterial muramyl dipeptide, and are strongly associated with increased susceptibility to Crohn's disease (CD). Saliva is a readily accessible source of DNA and RNA for genotyping CD patients for variants of the CARD15 gene, representing an alternative source of nucleic acid to that obtained from venous blood.

8834 (Pena Rossi C. et al., 2009)
Crohn's disease (CD) is an inflammatory, autoimmune disease of the bowel that causes non-specific symptoms including abdominal pain, diarrhoea, anorexia and fatigue. The aetiology of CD is unknown [1] with environmental, immune, psychological and genetic factors believed to be involved [2, 3]. CD is thought to arise primarily due to inappropriate chronic T-lymphocyte activation, with tissue damage induced by secondary macrophage activation [4, 5]. The prospect that CD is a form of T-helper type 1 (Th1) cell-dominant autoimmune disease is gaining acceptance, with support from the current use of immunosuppressants [4, 5].

8818 (Pierce, 2009)
Demonstrating large numbers of MAP organisms in the mesenteric fat, the blood vessels or lymphatics running through the mesenteric fat, the hyperplastic (probably not granulomatous) lymph nodes in the mesenteric fat, or the fistulas from patients with Crohn's disease will help establish that MAP causes Crohn's disease. **The hypothesis of autoinflammatory pathogenesis of CD does not assume the direct correlation of inflammation with numbers of MAP cells involved.**

0523 (Juste et al., 2009)
Our results showed a strong epidemiological association between MAP in blood and specific and non-specific immune responses. Since the type of association observed is unexpected because of its negative character regarding the cellular immune response and positive character related to the humoral immune response, it does not fit with conventional infectious disease models. For this reason, we propose an IBD model in which MAP, a low virulence mycobacterium, infects a large part of the human population but only causes a regional disease in a small part of it. This susceptibility could be related to genetic factors associated with a dysfunctional IFN-g activity that would fail to properly modulate the transition between innate and adaptive immune response or to innate immune response type local pathogenetic mechanisms triggered by the agent.

0274 (Marina-Garcia et al., 2009)
MDP induces caspase-1 activation through the Nlrp3 inflammasome, an event that requires exogenous ATP to deliver MDP from acidified vesicles into the cytosol .... which leads to the degradation of the bacterial cell wall and release of peptidoglycan fragments, was required for NOD2-induced signaling (45). Further studies are needed to understand the mechanism by which MDP and peptidoglycan fragments derived from bacteria activate NOD2.

8873 (Odes, 2008)
Studies on health care cost of ulcerative colitis (UC) and Crohn's disease (CD) are reviewed here. These studies were carried out in various countries with disparate health care systems. In the United States, data were often modeled or retrieved from large insurance schemes. Surgery and in-patient hospitalization accounted for over half the outlay on UC and CD. Fistulous disease in CD and parenteral nutrition were very costly. In Canada, overall charges were lower than in the United States, but there too, surgical costs were relatively high. In European studies, economic data were abstracted directly from patients' files. One pan-European study examined the outlay on UC and CD in a community-based prospective inception cohort followed for 10 years. Overall costs in Europe were lower than in the United States. Surgery, hospitalization, year of follow-up, disease phenotype in CD and ASCA-positivity impacted significantly on costs. In all studies, the cost data were right skewed, aminosalicylates were expensive drugs, and biological agents the most expensive; moreover indirect costs were not calculated. Infliximab raised costs considerably in CD, but there were no long-term followup studies, so that the cost-benefit of biological agents remains unknown. In conclusion, costs of managing UC and CD vary by country, surgery, genotype and several other factors. The most important question for further research is whether the biological therapies are cost-effective in the long-term.

PART TWO

PARATUBERCULOSIS (JOHNE'S DISEASE)

Incidence, etiology, pathogenesis, diagnosis, control. *Mycobacterium avium* subsp. *paratuberculosis* in milk and meat

0334 (De Luna and Sparagano, 2007)
The genus Mycobacterium is of utmost importance in veterinary medicine. Members of the genus cause serious diseases to livestock and they also could be transmitted to humans. Bovine tuberculosis, principally caused by M. bovis, is a widespread disease and causes serious economic losses to farmers worldwide. Paratuberculosis or Johne's disease caused by the M. avium complex, is a slow-developing disease present in several species, particularly cattle, and it has been associated with Chrone's disease of humans. It causes diarrhoea, and weight loss that could be fatal. The common diagnostic methods used rely upon culturing and microbiological identification of the Mycobacterium involved. However, the pathologically important species of Mycobacterium grow very slowly and could take up to eight to ten weeks to successfully grow on plates. Tuberculin and other immunological response tests are also widespread. However, these techniques also take long time to develop and often they are not conclusive. Recently molecular techniques based on the detection of Mycobacterium nucleic acids and proteins have proven a reliable and more important fast method for the diagnosis of Mycobacterium. Some tests can diagnose the presence of the pathogens in a couple of days. We reviewed the molecular methods available from the scientific literature that have reported to be useful in veterinary science.

0285 (Stephan, 2007) in German
The infection of domestic food animals with MAP organisms is associated with significant economic losses to the livestock industry worldwide through subclinical effects and subsequent death of the affected animals. The suggestions by some investigators of a possible link between human Crohn's disease and MAP has led to increased awareness of these microorganisms as far as public health is concerned. The classical detection method is based on isolation of MAP organisms from samples by culture techniques. However, this approach is labor intensive and time consuming. Moreover, this approach may underestimate or fail to detect MAP organisms as a result of the chemical decontamination step involved in order to prevent culture overgrowth by competing microflora. This step may also be deleterious to the MAP organisms in the sample. Immunology based detection methods are faster than culture, but are hampered by low sensitivity and cross reactivity problems. PCR provide a rapid alternative for qualitative and sensitive detection of MAP. Hence, to date a number of conventional PCR assays designed for MAP detection have been described.

0355 (Eamens et al., 2008)
Map was detected in more than 50% of the stored faecal samples from cattle shedding low concentrations of the organism. A pooling rate of 5 samples per pool is required to reliably detect infected low-shedder cattle using PFC based on radiometric culture.

0021 (Valentin-Weigand and Goethe, 1999)
From what is known to date, it seems thatM. a. ptb in its behavior both in vitro and in vivo follows the principle sit and wait for the best opportunity to multiply and spread. It is quite clear that the privileged niche occupied by the pathogen within the host is the intestinal macrophage. ...... it may be speculated that M. a. ptb is able to misdirect the immune response in a deactivating manner comparable, but not similar, to the immunomodulating effects of other mycobacteria.

0518 (Vissa et al., 2009)
.... landmark studies and novel approaches to give the reader an overall appreciation of the major advances in the study and the understanding of mycobacteria that have been accelerated by availability of genome sequences. .... M. avium subsp. paratuberculosis is an infectious agent of enteric disease in a broad range of hosts: cattle, goats, sheep and other wild ruminants. Evidence for

its role in human Crohn's disease is still actively debated [76, 78]. M. avium subsp. paratuberculosis isolates are further classified according to RFLP patterns and other phenotypic properties as S for

sheep and C for cattle, referring to host preferences. The C strains have a broader host range than S strains [41]. Subtractive DNA hybridization techniques lead to the identifi cation of a large deletion in the S strain covering 10 genes (MAP1734 to MAP1743c) of the M. avium subsp. paratuberculosis genome, ... The basis of pathogenicity of mycobacteria is thought to depend completely or in part on members of expanded gene families such as esx, PE-PPE, pks, mce etc. The COG abundance profi les comparisons demonstrate these genes and others that are common or enriched in the three pathogenic species relative to the non-pathogenic species (E. coli, M. avium subsp. avium, C. glutamicum). Nonpathogenic species also have orthologs for one or more of these genes, suggesting functions common to metabolism or biosynthesis of macromolecules. However, we found that the majority of the M. tuberculosis restricted genes are deemed 'non-essential' in experimental models. It is therefore clear that redundant genes (arising from gene duplication events) preclude the precise functional assignment of individual genes, particularly within the large families. Therefore, differential expression and complex genetic interactions are likely to infl uence pathogenicity and fitness of individual mycobacterial species within changing host milieus.

0345 (Cashman et al., 2008)
Since 1994, Irish cattle have been exposed to greater risks of acquiring Mycobacterium avium subspecies paratuberculosis (MAP) infection as a consequence of the importation of over 70,000 animals from continental Europe (O'Doherty et al., 2002; Good, 2004). The number of notified clinical cases of MAP increased annually from three in 1997 to 153 in 2003 (Good, 2004). Brady (2004) also found that in five MAP infected herds, all had imported heifers from continental Europe. Purchase of infected cattle, periparturient cow–calf management and exposure to faeces of infected animals are the main risk factors associated with MAP transmission between and within farms (Collins, 2003; Rossiter et al., 2004). ..... Improving the Johne's disease status of individual infected herds depends on the identification and elimination of infected animals and breaking the cycle of transmission (Johnson-Ifearulundu and Kaneene, 1998). The most rapid and effective progress is achieved when repeated individual animal testing is used within a biosecure management programme .... Culture is a recognised method of identifying viable MAP organisms, even though its sensitivity is relatively low. The milk sock filter is preferable to bulk milk when testing for MAP, noting that the milk sock is used to filter milk from all animals in a dairy herd, including any faecal material. ..... In this study, among the 12 MFR culture positive herds, each was positive at only one of the six testing events over the two year period.

0044 (Collins, 1997)
Mycobacterium paratuberculosis infects 5 to 10% of dairy cattle and approximately 33% of dairy herds in the US (1, 4, 9, 14, 23); also infected are beef cattle, sheep, goats, deer, elk, antelope, and camelids. Recent studies have shown that a high percentage of people with Crohn's disease, a chronic intestinal disease, are infected with M. paratuberculosis. Although no conclusion has been reached as to implications of this apparent association of M. paratuberculosis with Crohn's disease, some suggest that M. paratuberculosis is the cause. The purpose of this article is to review briefly the research concerning three aspects of this potential food safety issue. **Published already in 1997.**

0498 (Davies et al., 2009)
Paratuberculosis is not treatable and vaccinations do not prevent infection; therefore, economic loss is substantial in both the dairy and beef industries. ....susceptibility to M. avium sub. paratuberculosis infection is heritable, with heritability estimates ranging from 0.06 to 0.18 (Koets et al., 2000; Mortensen et al., 2004; Gonda et al., 2006). Evidence of genetic variation in host response to this disease has been reported.......

0501 (Delgado et al., 2009)
Therefore, we believe that immunohistochemistry and in situ hybridization to be useful for the post-mortem diagnosis and research of paratuberculosis.

0489 (Gao et al., 2009)
The animals with high numbers of CFU in milk culture may not be detected by fecal culture at all, and *vice versa*. A significant proportion (29% to 41%) of the positive animals would be missed if only one culture method, instead of both milk and feces, were to be used for diagnosis. This suggests that the shedding of MAP in feces and milk is not synchronized. Most of the infected cows were low-level

shedders. The proportion of low-level shedders may even be underestimated because MAP is killed during decontamination.

0020 (Kohler et al., 2001)
After immunization a cell-mediated immune reaction, reflected in an increased, specifically induced, interferon-c production developed much earlier (1±2 weeks post-immunization) than humoral immunity (8±16 weeks post-c immunization). While the increase in antibody titres was transient, declining to extremely low levels 48±60 weeks post-immunization, cell-mediated immunity remained detectable until the end of the investigation.

0511 (Begg et al., 2009)
The enzyme-linked immunospot (ELISPOT) assay is a highly sensitive technique for the detection of cytokines and has the potential to improve the diagnosis of JD. Of the variables examined, choice of capture antibody and the method by which the peripheral blood mononuclear cells were isolated significantly affected the ability to enumerate IFN-c–secreting cells. The ELISPOT assay was as sensitive as or better than the IFN-c ELISA at detecting ovine JD and could also detect disease at early time points postinoculation.

0014 (Stratmann et al., 2004)
The MptD protein is exposed on the surface of M. avium subsp. paratuberculosis during infection and is therefore a potential target for M. avium subsp. paratuberculosis immunization or treatment. These results together with the predicted functions of other genes in the 38-kb region strongly suggest its association with M. avium subsp. paratuberculosis virulence and that the 38-kb locus should be considered the first pathogenicity island identified in M. avium subsp. paratuberculosis.

0008 (Basler et al., 2006)
Mycobacterium paratuberculosis, Mycobacterium smegmatis, and lipopolysaccharide induce different transcriptional and post-transcriptional regulation of the IRG1 gene in murine macrophages. The regulation of IRG1 mRNA expression in macrophages differs at the transcriptional and the post-transcriptional level, dependent on the stimulatory agent. Although the function of IRG1 in macrophages is not known yet, we think that the IRG1 gene represents a suitable marker gene to dissect the molecular mechanisms contributing to macrophage activation by mycobacteria.

0499 (Pinedo et al., 2009)
The present results indicate a significant association between a polymorphism in the CARD15 gene and paratuberculosis infection in the cattle population under study. The product of CARD15 gene is an intracellular element responsible for the indirect recognition of bacterial peptidoglycan of both Gram negative and positive bacterial cell walls in monocytes, macrophages, dendritic cells, and intestinal epithelial cells, where it is mainly expressed (Ogura et al., 2001a). Structurally, it is composed of three segments: NH2-terminal caspase recruitment domains, a nucleotide-binding domain, and finally, a leucine-rich repeat (LRR) region, as is found in toll-like receptors (Hugot, 2006; Lakatos et al., 2006). Three main mutations of CARD15 have been found to be associated with an increased risk of CD. These mutations occur in the LRR domain or in its vicinity, suggesting an alteration in the recognition of the bacterial components (Lesage et al., 2002). CARD15 has been mapped to the bovine chromosome 18 (BTA18) and its transcript is 5105 bp long and the protein comprises 1013 amino acids...... the first large scale study showing an association between CARD15 and paratuberculosis infection in cattle. One of the potential limitations in a case-control study is that the available diagnostic tests for paratuberculosis detection fail to identify all infected animals. It is concluded that amino acid substitution C733R (SNP1) appears to be associated with susceptibility to paratuberculosis

infection in cattle. The information presented in this study adds new evidence relative to the potential of paratuberculosis as a zoonosis and provides support for an association between MAP and CD.

These data also contribute to the understanding of this disease and present new information for the control of bovine paratuberculosis.

0342 (Taylor et al., 2008)
Toll-like receptors (TLR) are engaged by ligands on microbial pathogens to initiate innate and adaptive immune responses. Little is known about TLR involvement during infection with Mycobacterium avium subsp. paratuberculosis (M. ptb), the cause of Johne's disease in ruminants, although there is a

profound immunopathological response in affected animals.We have analyzed the expression of 10 TLR genes relative to validated reference genes at predilection sites in ileum, jejunum and associated lymph nodes as well as in peripheral blood, to determine if TLR expression is altered in response to infection with M. ptb in outbred sheep. Previously unexposed animals from two flocks and animals from three naturally infected flocks were used with restricted maximum likelihood linear mixed modeling applied to determine significant differences. These were related to the pathologies observed at different stages of infection in exposed sheep, after allowing for other sources of variation. In most cases there were differences in TLR expression between early paucibacillary and multibacillary groups when compared to uninfected sheep, with most TLRs for the paucibacillary group having lower expression levels than the multibacillary group. Increased expression of TLR1–5, and 8 was observed in ileum or jejunum, and TLR1–4, 6, and 8 in mesenteric lymph nodes. There was a trend for increased expression of TLR1, 2, and 6–8 in PBMCs of exposed compared to non-exposed animals. ...... Most Johne's disease cases remain sub-clinical, thereby maintaining infection in a population. Initially, subclinical cases shed low numbers of bacteria in faeces, 10^4 g^{-1}, rising to 10^6 to 10^8 g^{-1} in clinical cases.

0432 (Nielsen, 2008)
The results in this study indicate that antibodies to MAP generally occur prior to shedding of MAP although transient or intermittent shedding can occur prior to the occurrence of antibodies. Progression of MAP infection is generally accompanied by occurrence of antibodies. Positive ELISA-results can only be confirmed with FC in a fraction of animals, because time from occurrence of antibodies to shedding of MAP varies greatly. ..... a positive ELISA was useful for predicting that an animal would subsequently become infectious. ....

0320 (Weiss et al., 2008)
The results of the present study indicated that the NF-κB signaling pathway is activated in MAP-exposed bovine monocytes, and findings of blocking experiments indicated that NF-κB may be involved in initiation of the transcription of cytokines, including TNF-α and IL-10. Results of the blocking experiments further indicated that NF-κB is involved in prevention of apoptosis but does not appear to play an important role in phagosome acidification or organism killing. Results of other recent studies30–32 in bovine monocytes indicate that MAP organisms initiate MAPK-p38 pathway activation through interaction with toll-like receptor-2. Because toll-like receptor-2 is known to activate the NF-κB pathway, it is a potential mediator of MAP-induced NF-κB pathway activation. The findings of our study taken together with results of other studies suggest that NF-κB and MAPK-p38 appear to be major pathways involved in initiating early transcription of IL-10 by MAP-exposed bovine monocytes.

0493 (Mikkelsen et al., 2009)
A significant association exists between early IFN-g and later FC status with occurrence of antibodies. In addition, the early IFN-g and FC status affect the antibody ELISA result at different stages post calving. We observed that only some IFN-g positive animals developed a positive antibody response against MAP, which indicate that cell-mediated immune responses can control or eradicate MAP in many animals.

0498 (Davies et al., 2009)
Paratuberculosis is potentially a serious zoonotic risk and further investigation into this link is essential. Studies of infection status of cattle have indicated that susceptibility to M. avium sub. paratuberculosis infection is heritable, with heritability estimates ranging from 0.06 to 0.18 (Koets et al., 2000; Mortensen et al., 2004; Gonda et al., 2006). Evidence of genetic variation in host response to this disease has been reported and a QTL affecting susceptibility has been mapped recently in US Holsteins to BTA20 (Gonda et al., 2007). Because the diagnosis of infected animals represents a major problem, paratuberculosis is not a good candidate for an OG approach; however, this may change if new diagnostic tests become available, which more easily enable the quantification of host genetic variation.

0495 (East, 2008)
The use of pooled samples or the use of two independent PCR tests can substantially reduce the number of tests required and the associated costs of such a survey

0480 (Keeble and Walker, 2009)
.... the ability of recombinant Mycobacterium avium ssp. paratuberculsis (MAP) HSP70, when administered in conjunction with an adjuvant, to result in a significant reduction in bacterial shedding in cattle infected with MAR Furthermore, its administration does not mask diagnostic assays, allowing clinical diagnosis to be maintained

0494 (Griffin et al., 2009)
All vaccine regimes reduced the overall pathological grading of biopsied intestinal tract (IT) tissues; among these, only GudairTM promoted a significant reduction in the incidence of histopathological IT lesions, while only i.p. injection of lipid-formulated live 316F significantly reduced the incidence of gross IT lesions. All lipid-formulated vaccines (but not GudairTM) significantly reduced the incidence of bacteriological culture-confirmed MAP infection. This study identifies a new vaccination strategy against Johne's disease in sheep using conventional MAP vaccine strains formulated in a metabolisable lipid delivery matrix.

0526 (Sweeney et al., 2009)
Vaccination resulted in the rapid development of an antigen-specific, cell-mediated immune response that was detected 7 days following injection and persisted through the duration of the study. Following the oral challenge with live MAP, calves in both groups had a secondary, steady increase in the IFN-γ response from whole blood cells. However, the response in unvaccinated calves was smaller than the response in vaccinated calves. The present study did not include an unchallenged group of vaccinated calves to determine whether the immune response from vaccination would have persisted without the stimulus of oral challenge with live MAP. The lack of detectable serum antibodies in the vaccinated calves in the present study is consistent with findings of a previous study, in which the earliest time points at which antibodies were detected were 60 to 100 days following vaccination and in some calves as late as 180 days. ..... Strong correlation between results for the MAP liquid culture and HEYM culture was detected in the present study. The liquid culture system has the advantage of a shorter duration of incubation and automated MAP detection. Quantification of MAP, which can be used for comparisons of interventions such as vaccination or antimicrobial treatment, is also possible with the liquid culture system. Additionally, both forms of culture appeared to have greater sensitivity for detection of MAP organisms than histologic examination of tissue samples. .... The results of the study reported here suggested that use of the strain 316F MAP vaccine stimulated an appropriate immune response to MAP in vaccinated calves. This resulted in reduced tissue colonization with the organism following oral MAP challenge.

0020 (Kohler et al., 2001)
Our results have shown that immunization with a live modifed M. paratuberculosis vaccine causes a CMI reaction which can be detected over a long period. Because of the close antigenic relationship, this reaction can interfere with the diagnosis of bovine tuberculosis when performed with methods for the measurement of CMI, such as the tuberculin skin test, IFN-c assay and LTT.

0340 (Karcher et al., 2008)
In paratuberculosis, the progression from a subclinical to a clinical stage of disease is characterized by a shift from cell-mediated (Th1) immunity to an antibody-mediated (Th2) humoral response. This shift in Th1 to Th2 immunity is characterized by a decreased percentage of peripheral blood T-cells and an increase in the percentage of B-cells for clinically infected cows (Waters et al., 1999; Koets et al., 2002). More specifically, the percentages of gd and CD4+ T-cells in peripheral blood are remarkably decreased in clinical cows compared with healthy controls (Koets et al., 2002). ...... Results of this study indicate that in dairy cows the percentages of both lymphocyte subsets and mononuclear cells are modulated by natural infection with MAP and by the periparturient period. In addition, these factors are capable of influencing the expression of the activation marker, CD5, on T cell subpopulations and B cells. The data presented are important because they highlight changes in the immune response of infected cattle at parturition that may be an attempt to limit the progression of Johne's disease during the highly stressful time of parturition.

0462 (Kathaperumal et al., 2009)
The results of our challenge studies showed significant levels of protection when the recombinant antigens were administered with DDA......... all the four recombinant antigens induced good Th1 and

antibody response and conferred protection against MAP infection in a goat model. It is concluded that vaccination of the goats with cocktail of recombinant antigens protected goats againstMAP challenge, which emphasizes that a successful subunit vaccine must have a number of antigenic epitopes recognized differentially in the context of MHC molecules.

0183 (Park et al., 2008)
Based on these results, we cloned and expressed 85A, 85B, 85C, SOD, and 35 kDa-protein genes into the eukaryotic expression plasmid pVR1020. C57BL/6 mice were immunized three times intramuscularly with the recombinant DNA cocktail and pVR1020 DNA alone as control. A significant reduction in the bacterial burden in the spleen and liver of mice immunized with the DNA cocktail as compared to the vector control group was found. Also, the relative severity of the liver and spleen histopathology paralleled the MAP culture results, more granulomas and acid-fast bacilli in the vector control animals. Moreover, mice immunized with the DNA cocktail developed both CD4+ and CD8+ T cell responses to the recombinant antigens and showed significant lymphocyte proliferation. The Th1 response related cytokine (IFN-□) levels increased in splenocytes obtained fromimmunized animals. These results indicate that the use of a recombinant DNA vaccine can provide protective immunity against mycobacterial infection by inducing a Th1 response.

0401 (Bush et al., 2008)
This GM model provides Australian sheep producers and their advisors with an estimate of the total and avoidable on-farm cost of OJD and the benefit of vaccination for Merino and first or second-cross prime lamb enterprises in southern NSW. Breakeven points for vaccination inform affected producers of the length of time required for a return on the investment in vaccination to be achieved. This model is a useful decision-making tool when developing on-farm strategies for the management and control of OJD at different disease levels and provides an opportunity for producers with infected flocks to improve their trading position through the use of vaccination.

0405 (Okafor et al., 2008)
.... a conductometric biosensor could detect MAP IgG in 2 minutes. The biosensor's speed of detection and the equipment involved would, among other things, support its application towards the various point-of care opportunities aimed at JD management and control.

0390 (Lu et al., 2008)
...... based on the simple mathematical model for US dairy herds, show that for farms with good management, culling of only high shedding animals is effective in controlling MAP transmission, but for farms with poor management, in addition to immediate culling of high shedding animals, culling of low shedding animals (based on the results of the fecal culture test), is necessary. Culling of low shedding animals is more efficient than that of high shedding animals to reduce R0. Culling of low shedding

animals may be delayed 6–12 months if a shorter testing interval is applied. This study suggests that if farmers prefer culling only high shedding animals, faster MAP detection tests (such as the fecal PCR and ELISA) of higher sensitivity should be applied with high testing frequency, particularly on farms with poor management. In general, culling of infectious animals with longer testing intervals is not effective in controlling MAP.

0368 (Rosseels and Huygen, 2008)
Existing vaccines, based on whole killed or live-attenuated bacteria, can delay the unset of clinical symptoms but do not protect against infection.

0507 (Branciari et al., 2008)
One-hundred four head of cattle between the ages of 12 and 24 months were tested in an EU slaughter house. The overall prevalence of animals infected with Mycobacterium avium subsp. paratuberculosis found by combining the results obtained by culturing the faeces, followed by confirmation of the positive samples by using PCR and serological tests was 11.54%. The isolation of Mycobacterium avium subsp. paratuberculosis from organ cultures in animals that tested negative in the standard testing methods suggests that the incidence of infection may be underestimated.

0514 (Alinovi et al., 2009)
This short communication documents the presence of MAP in fetal tissues of a heavily infected pregnant doe.

0457 (Whittington and Windsor, 2009)
In utero transmission of Mptb could retard the success of disease control programmes if the opportunities for post natal transmission via colostrum/milk and environmental contamination were able to be controlled.

0512 (Kumanan et al., 2009)
..... the IS900 gene sequence-based lateral flow biosensor assay developed is sensitive and specific for the detection MAP organisms in fecal samples. The assay was found to be effective in detecting as few as 10 organisms per 100 mg of feces. This assay is comparatively cheaper and does not require costly equipments in comparison to real-time PCR or PCR coupled with Southern blotting. ......the results can be obtained in a shorter time, in contrast to MAP culture techniques which take at least 6-8 weeks.

0510 (Selvaraju et al., 2008)
PCR based assays require specialized skills and are still relatively expensive for certain in-house industrial analytical laboratories and low-cost diagnostic facilities. Hence, alternative DNA-based methods involving simple instrumental skills such as microscopy and probing of the whole cells instead of DNA extraction before probing or amplification will be desirable for a routine screening for assessment of exposure or infection. Fluorescence in situ hybridization (FISH) is one such method, which is less expensive and adaptable for routine screening applications in clinical and environmental settings [12,16,23,29].

8841 (Lehtola et al., 2006)
.... a rapid PNA FISH method that can identify clinically important M. avium subsp. avium and M. avium subsp. paratuberculosis bacteria. The method was applied to smears of pure cultures of the bacteria and to biofilm and water samples taken from a Propella biofilm reactor .....

8849 (Sommer et al., 2009)
In MAP infections, a cytotoxic and proinflammatory Th1-like response is essential to control disease. While such a response may initially develop, this typically gives way to a Th2-like response later in infection. Interaction between CD40 receptors on macrophages and CD154 (CD40L) on activated T cells is crucial for maintaining a Th1 response and activation of macrophages. In this report, we investigated the hypothesis that CD40 signalling is impaired in MAP-infected macrophages.

Uninfected bovine monocyte-derived macrophages (MDM) responded to CD40L by up-regulating expression of genes encoding IL-6, TNFa, IL-8, iNOS, IL-10, and IL-12p40.

0520 (Orpin and Sibley, 2009)
Demonstration of 'MAP freedom' at herd level by whatever method chosen appears to be fraught with difficulties. There is the distinct risk that, due to the limitations of the tests applied, herds will be incorrectly assigned a 'MAP free' status only to fail the tests in the future. This creates mistrust and disillusionment among farmers and vets alike, and we end up in the current situation, where Johne's disease is inadequately managed on farms. As part of the development of a new web-based health planning system designed to improve cattle health outcomes, a risk assessment module was created to assess the likely presence of Johne's disease within herds, together with a separate module that assessed the potential spread of MAP within the herd, based on published risk factors (National Johne's Working Group 2003). The system then allows vets to assess herds ...... and develop bespoke surveillance strategies linked to risk. High-risk herds are tested more frequently than low-risk herds. Low-risk herds have options to progress to official accreditation if this is beneficial to their businesses. This creates a system for prospective management of disease for every herd in the practice, rather than retrospective management of known infected herds: truly preventive medicine. All herds are viewed as potentially infected with MAP, and categorisation avoids the concept of labelling a herd as 'free' of disease based on inadequate tests. The objective is to create cost-effective systems that allow vets and farmers to establish the likely prevalence and develop appropriate control programmes according to the individual aspirations of the farmer. Indeed, in many lightly infected

herds, husbandry methods alone may be all that are required to control the spread of MAP. In others, an effective biosecurity plan may be more appropriate.

0406 (Herthnek et al., 2008)
A method to detect MAP in milk with real-time PCR was developed for screening of bulk tank milk. Pellet and cream fractions of milk were pooled and subjected to enzymatic digestion and mechanical disruption and the DNA was extracted by automated magnetic bead separation. The analytical sensitivity was assessed to 100 organisms per ml milk (corresponding to 1–10 CFU per ml) for samples of 10 ml. The method was applied in a study of 56 dairy herds to compare PCR of farm bulk tank milk to culture of environmental faecal samples for detection of MAP in the herds. In this study, 68% of the herds were positive by environmental culture, while 30% were positive by milk PCR. Results indicate that although MAP may be shed into milk or transferred to milk by faecal contamination, it will probably occur in low numbers in the bulk tank milk due to dilution as well as general milking hygiene measures. The concentration of MAP can therefore be assumed to often fall below the detection limit. Thus, PCR detection of MAP in milk would be more useful for control of MAP presence in milk, in order to avoid transfer to humans, than for herd prevalence testing.

8851 (Stratmann et al., 2002)
Based on phage display technology, a peptide-mediated magnetic separation technique was developed to facilitate selective isolation of Mycobacterium avium subsp. paratuberculosis (M. paratuberculosis) from bulk milk of naturally infected dairy herds. Nine recombinant bacteriophages binding to M. paratuberculosis were isolated from a commercial phage-peptide library encoding random 12-mer peptides. Nucleotide sequencing revealed the deduced sequence of the binding peptides. One peptide with the sequence NYVIHDVPRHPA, designated aMP3, was chemically synthesized with an amino-terminal biotin residue attached via an amino-hexacarbonic acid spacer molecule. Paramagnetic beads coated with the phage or with peptide aMP3 enabled the capture of M. paratuberculosis from milk. Combining this peptide-mediated magnetic separation with an ISMav2-based PCR allowed the detection of M. paratuberculosis in artificially spiked milk down to a concentration of 10^1 ml^{-1}. Experiments using milk from naturally infected cows and bulk milk samples from infected herds demonstrated that the peptide-mediated capture PCR is sufficiently sensitive to detect single strong shedders in pooled milk samples. The method, for the first time, applies phage display technology to microbial diagnostics and has potential value as a completely standardizable tool for the routine M. paratuberculosis screening of bulk milk samples at acceptable costs.

8850 (Stratmann et al., 2006)
...... the peptide-mediated capture PCR might present a suitable test for paratuberculosis screening of dairy herds, as it has an analytical sensitivity sufficient for detection of M. avium subsp. paratuberculosis in bulk milk samples under field conditions, relies on a defined and validated ligand-receptor interaction, and is adaptable to routine diagnostic laboratory automation.

0352 (Slana et al., 2008b)
.... review the different methods used for detection of MAP in milk and milk products. We analyze the current methods for direct or non direct identification of MAP and culture and molecular biology methods that can be applied to milk and milk products.

0451 (Slana et al., 2008a)
The highest detected number of MAP in milk was 560 cells/ml. ..... To assess the dilution effect on contaminated milk in the tank, the occurrence of MAP in the tank during the progress of the milking procedure on 1st and 6th of collection days was monitored. Altogether 73 cowswere milked on each of both collection days. At our request cow No. 58 was milked first. After this, the other cows were milked according to the standard order. Milk was stirred in the tank during the whole process of milking. TM samples were collected continuously according to the number of cows milked into the tank (Table 6). MAP was detected in 4 of 5 BTM samples. All 4 positive BTM samples included milk from the clinically infected cow No. 58. In the fifth BTM sample the milk of this cow and 20 others was not present and the BTM sample was negative. .... In this study 32.5% and 80.0% of the individual and BTM milk samples tested positive for MAP by real time qPCR. This prevalence is in agreement with other authors (Pillai and Jayarao, 2002; Stabel et al., 2002). .... Higher levels of positivity for MAP in milk were exhibited by cows in close proximity to the two clinically ill individuals. Other positive cows that were stabled quite far from the clinically ill ones had moderate or very low numbers of MAP in milk. These data suggest that MAP spreads mainly by the exogenous route via the contamination of milking equipment such as teat cup liners. Further study of this matter is required. The described technique for DNA isolation from milk using a commercially available kit is fast, relatively cheap and allows the processing of large numbers of samples. It also allows partial automation, which increases the number of samples that can be processed.

0337 (Clark et al., 2008)
Culture and PCR were able to detect more positive animals than ELISA. Overall, direct fecal PCR was 70.2% sensitive and 85.3% specific when using culture as the gold standard. The ELISA method was 31.3% sensitive and 97.8% specific. When culture reported <10 cfu, the sensitivity and specificity of PCR and ELISA were 57.1 and 85.3%, and 4.8 and 97.8%, respectively. When culture reported 10 to <40 cfu, the sensitivity of PCR and ELISA were 75 and 50%, respectively. When culture reported ≥40 cfu, the sensitivity of PCR and ELISA were 100 and 88.2%, respectively. The direct PCR outperformed the ELISA in detecting animals potentially infected with MAP and was not significantly different when compared with culture. The direct fecal PCR method described here provides faster results than traditional culture and is more sensitive than ELISA at detecting animals suspected of Johne's disease.

8853 (Dieguez et al., 2009)
The results indicate that neither the currently available serum ELISAs nor faecal culture and PCR are effective for the early detection of MAP in dairy cattle.

0274 (Marina-Garcia et al., 2009)
..... the rapid and sensitive detection of tagged PCR amplicons on a giant magneto-resistance detection platform. Actuation of magnetic particles used as detection labels speeds up the total assay time resulting in a 3-min incubation and detection assay. The amplicons can be measured over two orders of magnitude, ranging from 4 to 250pM of PCR product. Experiments have shown that the detection platform can be used for multi-analyte detection of four different amplicons with equal performance. We consider this rapid and sensitive detection system to be very promising for further integration into a point-of-care nucleic-acid analysis instrument, including miniaturized sample pretreatment and amplification.

0527 (Leroy et al., 2009)
.... currently available diagnostic tests still lack performance to be used in large-scale control programs. .... a genomic analysis of Map genome allowed us to identify in silico Map-specific candidate antigens. ..... Obviously, the use of a 'golden' antigen should be able to discriminate all infected animals regardless of their clinical stage and with a very small false-positive rate. But it is now clear that such an antigen probably does not exist and that the most efficient diagnostic test would be based on a combination of purified antigens. Moreover, Bannantine and coworkers (2008a) have demonstrated, using an experimental infection model that diagnostic efficiency of an antigen could vary and decrease over the course of the infection. Thus antigens effective in detection of clinical cases could be inefficient for early diagnosis.

8854 (Mobius et al., 2008)
..... faecal culture on solid media is still the most sensitive and specific method for direct diagnosis of paratuberculosis in animals, and thus the ''gold standard'' (Stabel, 1997). However, the method is too laborious and time consuming to be applicable in large-scale diagnostic programmes. Therefore, direct detection of Mycobacterium avium subsp. paratuberculosis (MAP) in faecal samples by PCR seems to be a practicable alternative. .... selection of IS900-specific primers ensures that IS900 remains a favourite target sequence for amplification of MAP specific loci. The studied PCR systems based on f57, and locus 255 can also be recommended. Revision of ISMav2 primers is necessary. Single-round PCR systems are very reliable. Nested PCR assays were occasionally disturbed by contaminations, thus bearing a risk for routine diagnostics.

0503 (Whittington, 2009)
Culture of Mycobacterium avium subsp. paratuberculosis is the definitive diagnostic test for Johne's disease, a chronic granulomatous enteropathy of animals. Compared to solid media, the identification

of all strains of the organism in liquid media can be more difficult because the appearance of colonies and mycobactin dependence are not observable, and the growth of other organisms needs to be distinguished, commonly by PCR. .... The long-term contamination rate for fecal cultures was about 7%, and that for tissue cultures was <0.2%. Liquid medium was more sensitive than solid medium culture for M. avium subsp. paratuberculosis.

0203 (Scott et al., 2007)
Estimated sensitivity values for the serum ELISA and bacterial culture of feces were slightly lower than those reported elsewhere.The enhancecd qRT-PCR method offered relative improvements in sensitivity of 52% and 59% over serum ELISA and microbial culture, respectively. Pooling of fecal samples and testing with the gel-based PCR assay are not recommended.

0284 (Khare et al., 2008)
DNA-based PCR could be an alternative for situations where intermediate or long-term storage is necessary. Shortterm storage of fecal samples at 4°C and longer term storage at -70℃ appear to have no deleterious effects on M . paratuberculosis viability, but short-term storage at -20℃ should be avoided as it substantially reduces the viability of the bacteria in the sample.

0004 (Schonenbrucher et al., 2008)
In the present study, a robust TaqMan real-time PCR amplifying the F57 and the ISMav2 sequences of Mycobacterium avium subsp. paratuberculosis from bovine fecal samples was developed and validated. .... Comparative analysis of 108 naturally contaminated samples of unknown M. avium subsp. paratuberculosis status resulted in a relative accuracy of 98.9% and a sensitivity of 94.4% for fecal samples containing <10 CFU/g feces compared to the traditional culture method.

0530 (Mucha et al., 2009)
a noteworthy association between TLR mutations and increased susceptibility to MAP infection was found. Considering the highly conserved nature of TLRs among mammals, these novel mutations may play a significant role in MAP infection not only in cattle but also in other mammals including humans.

0359 (Pinedo et al., 2008)
Ability of serum ELISA as indicator of the likelihood of milk shedding of Mycobacterium paratuberculosis in dairy cows is questionable. .... MAP isolation from milk was first reported in 1935. More recent studies have found MAP isolation rates in milk of up to 45% in clinically affected animals and of up to 22% in colostrum or 8% in milk in subclinical cases.

0362 (Haghkhah et al., 2008)
..... herd-level prevalence of MAP infection in the Fars province, southern Iran 11% based on IS900 nested PCR on bulk-tank milk samples. Grant et al. (2002) performed an immunomagnetic PCR on 244 bulk-tank milk samples from the United Kingdom and detected a herd-level prevalence of 7.8%. In another study, Corti and Stephan (2002) examined bulk-tank milk samples from different regions in Switzerland and showed that 19.7% of the 1384 milk samples were IS900 PCR positive. .....

8849 (Sommer et al., 2009)
Johne's disease ranks as one of the most costly infectious diseases of dairy cattle (Sweeney, 1996).

0405 (Okafor et al., 2008)
In the U.S., economic losses from the disease have been estimated to exceed $1,500,000,000 per year, mainly from the effects of reduced milk production.

0358 (Groenendaal and Zagmutt, 2008)
Three scenarios were developed based on the effectiveness of possible risk-mitigation strategies. In the first scenario, it was assumed that an effective strategy exists; therefore, a negligible demand decrease in the consumption of dairy products was expected. In the second scenario, it was assumed that new risk mitigation would need to be implemented to minimize the health hazard for humans. In this case, a small milk demand decrease was expected, but larger demand decreases were also possible. The third scenario assumed that no fully effective risk mitigation was available, and this resulted in a considerable demand decrease and a potential reduction in milk supply as a result of

regulatory measures. A milk demand reduction of 1 or 5% resulted in a reduction in consumer surplus of $600 million and $2.9 billion, and a reduction in dairy farm income of $270 million and $1.3 billion, respectively.

0339 (Tiwari et al., 2008)
For an average Canadian dairy herd with 12.7% of 61 cows seropositive for MAP, the mean loss was $2992 (95% C.I., $143 to $9741) annually, or $49 per cow and $409/seropositive cow per year.

0453 (Nielsen et al., 2009)
Milk production was 20 and 17% less in the last lactation compared with the previous lactation among culled cows with clinical symptoms and among cows that were positive in a serum antibody ELISA with MAP infection confirmed by histology, respectively, in a Dutch study (Benedictus et al., 1987).

0470 (Nielsen and Toft, 2009)
Herd level prevalences were likely to be >50% in many countries. In sheep and goats, the within-flock prevalences could not be estimated. On flock level, the prevalences were thought to be >20% for both sheep and goats, based on studies from Switzerland and Spain.

0026 (Skovgaard, 2007)
The emerging technologies in food production explain how new pathogens can establish themselves in the food chain and compromise food safety. The impact of the food technology is analysed for several bacteria, such as Yersinia, Campylobacter, Arcobacter, Helicobacter pullorum, Enterobacter sakazakii, **Mycobacterium avium spp. paratuberculosis**, prions related to vCJD and others.

0050 (Khol et al., 2007)
Austria is the second European country to declare clinical paratuberculosis a notifiable disease. The prevalence of serologically positive Austrian cattle farms rose significantly to 19.0% during the past

years. .... Paratuberculosis is a worldwide problem that cannot be controlled by one country alone. Actions on a European level are necessary to prevent further spreading of the disease and toprotect MAP free livestock. Another big advantage of declaring clinical paratuberculosis a notifiable disease with compulsory culling of affected animals and compensation is that consequently the disease is recognized as important by farmers, veterinarians and consumers. The introduction of the Paratuberculosis-Regulation in April 2006 caused many discussions about the disease, control strategies of MAP in livestock and the usability of the regulation.

PART THREE
AUTOIMMUNE AND AUTOINFLAMMATORY DISEASES
Bacterial triggers with special attention to mycobacteria (peptidoglycans, muramylpeptides,heat shock proteins), genetic and environmental factors, proinflammatory cytokines

8855 (Carbone et al., 2005)
ASM-American Academy of Microbiology listed Crohn's disease among the chronic diseases for which there is suspicion of an infectious etiology. Koch's postulates, still seen by many as a necessary tool for proving causative links between microbes and disease, is not equal to some of the particular problems of chronic illnesses. Many of the causative organisms involved in chronic illness cannot be cultivated using recognized techniques, and others position themselves in tissues that are difficult to access for sampling, making them hard to detect in the afflicted. In some cases, the span of time between infection and disease onset is too long to carry out meaningful studies of the effect of reintroduction. New tools must be developed to circumvent these problems and criteria must be established against which evidence of causality can be measured in cases where Koch's postulates are not appropriate. .... Other pathogens create their respective chronic diseases by triggering an immune response in genetically susceptible hosts. Susceptible individuals stage responses that may be either overly vigorous (leading to self-destructive fallout) or insufficient to clear the organism. Crohn's disease, for example, does not result from infection alone, but from the confluence of infection and genetic susceptibility. Susceptible individuals, who carry the NOD2 or TNFR polymorphisms, may respond to certain commensal intestinal flora, stimulating acute inflammation that leads to chronic inflammation and colitis.

8848 (Kucharzik et al., 2006)
The following review summarizes the current view on the four basic tenets of the pathophysiological basis of IBD and its implications for therapies of IBD: genetics, immune dysregulation, barrier dysfunction and the role of the microbial flora.

0516 (MacDonald and Monteleone, 2005)
Crohn's disease and UC represent complex genetic diseases but also tend to run in families. Genome-wide mapping has identified Crohn's disease susceptibility loci on chromosomes 1, 5, 6, 12, 14, 16, and 19 (46). In 2001, two groups mapped the locus on chromosome 16 to Nod2 (47, 48). Three major polymorphisms have been specifically associated with È15% of Crohn's disease cases (Arg702Trp, Gly908Arg, and Leu1007fsinsC), and all are in, or around, the LRR region of the protein required for recognition of bacterial muramyl dipeptide (MDP). Individuals who carry two copies of the risk alleles have a 20- to 40-fold increase in their risk of developing Crohn's disease. About 8 to 17% of Crohn's patients carry two copies of the major risk-associated alleles, compared with 1% of the general population. Interestingly, Nod2 has not been found to be associated with Crohn's disease in Japan (49), again highlighting the complex nature of this disease. ..... Two other genes associated with Crohn's disease have been identified. The first of these, located on 5q31, encodes the organic cation transporter (OCTN) genes, and mutations at these loci affect the ability of the transporters to pump xenobotics and amino acids across cell membranes (60). In the gut, these genes are expressed in epithelial cells, macrophages, and T cells, correlating closely with their potential function in IBD. The second gene is located on 10q23 and encodes the guanylate kinase DLG5 (61). The mutation in this gene involves a single amino acid substitution that is thought to impair the ability of DLG5 to maintain epithelial polarity. Both genes may be important in epithelial permeability, and disruption of this function could lead to inappropriate exposure of the mucosal immune system to bacterial products. The other major antigenic challenge facing the gut derives from ingested food antigens. Under normal circumstances, oral administration of protein antigens induces systemic unresponsiveness when the same antigen is given parenterally (a phenomenon known as oral tolerance). In animal models, oral tolerance appears to be a specific consequence of the immune environment in the gut, which favors

the generation of T regulatory cells (62). In recent years, food allergy has become increasingly common, and although there has been little progress in understanding host mechanisms involved at the molecular level, there has been great progress in clinical management (63). ..... Recent years have seen the identification and characterization of dedicated regulatory T cells, in both mice and humans, that have the ability to profoundly suppress a variety of immune responses ...... There are

clear indications that the flora is beneficial but also has the potential to be harmful, and increasing knowledge of how the flora interacts with the immune system should allow exploitation of the former and minimization of the latter.

0517 (Kobayashi et al., 2005)
The Nod2 protein is a critical regulator of bacterial immunity within the intestine, providing a possible mechanism for Nod2 mutations in CD. ....Previous studies have shown that pre-treatment with MDP sensitizes mice to endotoxic shock induced by LPS injection (14) .... Nod2 is essential in the detection of bacterial MDP and is capable of activating the adaptive immune system by acting as an adjuvant receptor for antibody production, either directly or by enhancing the production of a-defensins (27, 28) or other immunostimulatory molecules. Therefore, Nod2 is critical in protecting the host from intestinal bacterial infection. More specifically, we reveal an important role for Nod2 in the regulation of a subgroup of cryptdins, offering a plausible mechanism to explain the association between Nod2 and susceptibility to CD. .... CD-associated Nod2 mutations predispose primarily to ileal lesions (30–33), corresponding to the location of Paneth cells. Recent reports suggest that the expression of a-defensins is diminished in human CD patients, particularly those who have Nod2 gene mutations (34, 35). However, it remains to be established whether a defect in Paneth cell function is the only possible mechanism by which Nod2 mutations might associate with the development of CD in humans. Nevertheless, it seems reasonable to suggest that mutations in Nod2 might promote CD through defective regulation of responses to commensal and/or pathogenic bacteria, rather than acting as an initiating factor for disease.

8806 (Maeda et al., 2005)
Variants of NOD2, an intracellular sensor of bacteria-derived muramyl dipeptide (MDP), increase susceptibility to Crohn's disease (CD). These variants are thought to be defective in activation of nuclear factor kB (NF-kB) and antibacterial defenses, but CD clinical specimens display elevated NF-kB activity. To illuminate the pathophysiological function of NOD2, we introduced such a variant to the mouse Nod2 locus. Mutant mice exhibited elevated NF-kB activation in response to MDP and more efficient processing and secretion of the cytokine interleukin-1b (IL-1b). These effects are linked to increased susceptibility to bacterial-induced intestinal inflammation and identify NOD2 as a positive regulator of NF-kB activation and IL-1b secretion. ...... The intestinal inflammatory response to DSS is dramatically reduced by oral antibiotics, which supports involvement of enteric bacteria (14). When given a high dose of DSS (6%) without oral antibiotics, WT and Nod22939iC mice died within 9 days after DSS administration (12), but mice that received oral antibiotics survived and developed mild inflammation and weight loss, without any genotype-linked differences (fig. S9). Thus, enteric bacteria elicit the inflammatory response to DSS, and without bacterial exposure, Nod22939iC mice have the same reaction as WT counterparts. Exposure of macrophages to bacteria activates inflammatory and apoptotic caspases

8618 (Girardin et al., 2003)
Human Nod1 specifically detects a unique diaminopimelate-containing N-acetylglucosamine– N-acetylmuramic acid (GlcNAc-MurNAc) tripeptide motif found in Gram-negative bacterial peptidoglycan, resulting in activation of the transcription factor NF- B pathway. Moreover, we show that in epithelial cells (which represent the first line of defense against invasive pathogens), Nod1 is indispensable for intracellular Gram-negative bacterial sensing. .... The peptidic composition of the peptidoglycan degradation products, either released by the bacteria or processed by the host cell in the lysosomal compartment, is critical in defining the host response to bacterial infection. In this respect, the characterization of the peptidoglycan motifs sensed by Nod1 and Nod2 suggests that these two molecules have complementary and non-overlapping functions that contribute to innate immunity. Moreover, our results show that Nod1 is likely the sole sentinel molecule in the epithelial barrier

allowing intracellular detection of bacteria through peptidoglycan sensing, thereby highlighting its key role in innate immune defense.

0001 (Cash et al., 2006)
This idea is underscored by the fact that deficiencies in antimicrobial peptide expression are associated with inflammatory bowel disease (IBD) (2, 3), a chronic inflammatory disorder thought to be triggered by resident gut microbes. Paneth cells were harvested by laser capture microdissection from "germ-free" (microbiologically sterile) mice and "conventionalized" mice (germ-free mice

reconstituted for 10 days with an intestinal microflora from conventionally raised mice)......... One of the most prominent responses uncovered by our analysis was a 31-fold increase in the abundance of RegIIIg transcripts in Paneth cells from conventionalized as compared with germ-free mice (table S1). Increased expression of RegIIIg mRNA was confirmed by quantitative real-time polymerase chain reaction (Q-PCR) (Fig. 1A) and correlated with increased protein expression (Fig. 1B).

8883 (Traub et al., 2006)
In 1974, MDP was discovered as the minimal structure responsible for the improved reaction to mycobacteria in Freund's complete adjuvant. Since then, much effort has been made to isolate, synthesize and characterize the activity of MDP and other muropeptides. .... Numerous reports suggest that MDP and other muropeptides directly induce cytokines, thus activating and modulating immune responses and inflammation. .... MDP and other muropeptides are strong immune amplifiers. In contrast to priming effects, where one stimulus is administered before the second stimulus, synergism is defined as a situation in which the combination of two weak stimuli, leads to an enhanced response. Synergistic actions of MDP and LPS have been described in many studies using different cell types and stimulus concentrations. Although different end-points, mostly cytokines, have been measured, all studies consistently describe strong synergistic effects of LPS and muropeptides. .... Free muropeptides are present in the body during infection. They are naturally released during bacterial growth and division, by antibiotic treatment or the activity of lytic host enzymes. The diversity of PGNs from different bacterial strains leads to a variety of possible muropeptide structures. Muramyl dipeptide (MDP) is a prominent motif and represents the minimal biologically active structure. Surprisingly, larger structures do not exert enhanced biological activity. The cytosolic proteins NOD1 and NOD2 have been identified as important muropeptide receptors, although possible contributions of CD14 and TLR cannot be fully excluded. Remarkably, these NOD proteins are intracellular receptors suggesting a role for phagocytosed or intracellular pathogens.

8811 (Laslo et al., 2008)
As a consequence of rapid progress being made in the analysis of transcription factors and miRNAs that regulate the development of innate and adaptive cells of the immune system, it should be possible in the near future to assemble them into complex gene regulatory networks and analyze these intricate control circuits using mathematical and computational modelling. Such modelling may yield counter-intuitive predictions that can be experimentally tested. Progress in this area will also facilitate the directed and efficient generation of specific immune cells and their manipulation for cell based therapies.

8812 (Tamura et al., 2005)
Dendritic cells (DCs)3 are bone marrow (BM)-derived professional APCs. They play central roles in the induction of both innate and adaptive immunity (1). DCs recognize various pathogens and their components through pattern recognition receptors such as TLRs and produce a variety of cytokines. They capture and process Ags to present antigenic peptides associated with MHC molecules to T cells, eliciting the Th1 and Th2 responses as well as inducing tolerance. In addition, DCs regulate other immune cells, including B and NK cells. The extensive diversity of DC function is partly attributed to the presence of multiple DC subsets (2, 3).

0006 (Lotz et al., 2007)
After antigen presentation, lymphocytes in PPs differentiate into effector lymphocytes and B cells start to produce immunoglobulin A (IgA) (Neutra et al., 1996; Macpherson and Uhr, 2004). Subepithelial

polymeric (mostly dimeric) IgA is translocated into the intestinal lumen by the polymeric immunoglobulin receptor (pIgR) and plays an important role in the antibacterial host defense (Fagarasan and Honjo, 2003; Wijburg et al., 2006). pIgR is required for the adaptive host defense against enteropathogenic bacteria and is highly expressed in crypt intestinal epithelial cells (Bens et al., 1996; Tang et al., 2006; Wijburg et al., 2006). Mucosal IgA has additionally been shown to possess anti-inflammatory activity by binding to bacterial lipopolysaccharide (LPS), a potent immunostimulatory membrane glycolipid produced by all Gram-negative bacteria (Fernandez et al., 2003). In addition, dendritic cells (DC) sample microbial antigens by cell extensions that breach through the epithelial layer and reach the intestinal lumen (Niess et al., 2005; Uhlig and Powrie, 2003; Neutra et al., 1996). DCs play an important role as regulators of immunity to pathogens, oral tolerance, and intestinal inflammation. It is probable that the functional characteristics of DCs isolated from intestinal tissue is determined by factors produced by resident stroma cells leading to a specialized intestinal DC phenotype (Sierro et al., 2001; Rimoldi et al., 2005; Niess and Reinecker, 2006). .... the intestinal microflora also influences the development of the adaptive gut immune system. Germfree mice show lower levels of immune cells and soluble immunoglobulins in the intestinal tissue. This situation is rapidly reversed after microbial colonization with lymphocyte expansion, reappearance of germinal centers and rise of immunoglobulin levels (Butler et al., 2000; Fagarasan et al., 2002). .... microbial ligands, the structures recognized by the innate immune system, can also be found in significant concentrations in the gut of germ-free mice ingested by the sterile, but not ligand-free nutrients. .... A similar recognition system for bacterial ligands leading to cellular activation displays the NOD1/NOD2 receptor system. While TLRs are membrane-bound receptors recognizing bacterial constituents, the NOD proteins reside in the cytoplasm. NOD1 binds to diaminopimelic acid-containing tripeptide iEDAP found in mainly Gram-negative bacterial peptidoglycan (Chamaillard et al., 2003; Girardin et al., 2003a), and is broadly expressed in tissues (Inohara et al., 1999). NOD2 detects muramyl dipeptide (MDP), the minimal bioactive peptidoglycan motif shared by all bacteria (Inohara et al., 2003; Girardin et al., 2003b). NOD2 was formerly believed to be expressed exclusively in monocytes (Ogura et al., 2001b) but more recent data revealed that it can be up-regulated in epithelial cells by NF-kB activation (Gutierrez et al., 2002) and thus it may be an important player in mucosal host defense. .... Bacterial peptidoglycan fragments may use the same way to enter the cytoplasm (Viala et al., 2004). .... The presence of functional PRRs such as NOD or TLR molecules within the intestinal mucosa in the presence of the microflora rises a major question: How is cellular activation, the recruitment of professional immune cells and inflammation of the intestinal tissue avoided? The relevance of this question is illustrated by associations found between mutations within PRRs such as NOD2 and the inflammatory bowel disease Crohn’s disease (Hugot et al., 2001; Ogura et al., 2001a; Franchimont et al., 2004). Similarly, mice expressing the homologue mutation within Nod2 exhibit a hyperinflammatory phenotype (Maeda et al., 2005). Also, mice lacking Tlr4 expression showed a more severe disease in the DSS model of colitis (Rakoff-Nahoum et al., 2004). Thus, excess or absent PRR activation within the intestinal mucosal tissue may significantly contribute to organ dysfunction and gastrointestinal disease. ....

0265 (Silva et al., 2009): Recent advances in DNA vaccines for autoimmune diseases
The results obtained strongly suggest that Hsp65 posses immunoimodulatory effects that can be used against different illnesses.

0204 (Deretic, 2008)
A new general bactericidal process, autophagy, which can eliminate intracellular pathogens. This is a new, rapidly growing field replete with possibilities for novel, previously untried immunologic and pharmacologic interventions applicable not only to TB but to other stubborn bacterial, parasitic and viral diseases.

0249 (Chen and Liu, 2009)
Interferon γ (IFNγ) is a cytokine with multiple biological and pathological functions. Many cells express IFNγ receptor and can respond to IFNγ signalling. CD4+ T-cells are a group of cells including Th1, Th2, Th17 cells, T-regulatory cells and cd T-cells, which regulate adaptive immune responses and could be influenced by IFNγ signalling. The action of IFNγ on T-cells may mediate the progression of autoimmune diseases like arthritis, multiple sclerosis and diabetics. IFNγ has been demonstrated to

play important roles in the pathogenesis of the autoimmune diseases through its ability to regulate T-cell function.

0736 (McCullough et al., 2009)
Nature is continually confronting us with new challenges to immune defence development and protection of the host. It is important to consider that nature has one dominant characteristic – chaos – ensuring that the most successful of hosts and pathogens will continue to survive.

8831 (Dow, 2006)
Hsp65 is a heat shock protein that is unique to mycobacteria. There is an important role for heat shock proteins in autoimmunity and infection; glutamic acid decarboxylase (GAD), the prime antigen of Type 1 diabetes, has similar amino acid sequences to Hsp65 and Hsp65 ‘‘should not be completely discarded as having a possible role in the development of Type 1 diabetes’’. In a study of children newly diagnosed with Type 1 diabetes 47/47 were found to respond to mycobacterial Hsp65.

PART FOUR
MYCOBACTERIA IN THE ENVIRONMENT, WATER, BIOFILMS AND AEROSOLS

8858 (Falkinham, 2003)
One factor that will have an impact on nontuberculous mycobacterial disease involves overlaps between human and mycobacterial ecology. Mycobacteria and humans share the water supply. The need to reduce the incidence of water-associated gastro-intestinal disease has led to a widespread implementation of disinfection of drinking water (57). One consequence of this effort is selection for disinfectant-resistant mycobacteria that can grow in the limited organic matter in water made available by the death of microbial competitors. The presence of mycobacteria in drinking water (24) and thisconcentration in hospital hot water systems (58), coupled with an aging human population (changing demographics) and increases in the proportion of the population that are immunodeficient (changing human susceptibility) may place more individuals at risk for nontuberculous mycobacterial disease.

8837 (Collins et al., 1984)
Mycobacteria .... are present in most natural waters and piped water supplies.
Few subtitles from the review published already in 1984:
Resistance of mycobacteria to chlorination
Access, persistance and colonization in piped supplies
Is water the natural habitat of free-living mycobacteria?
Water as a vector for mycobacterial infections
Immune response to environmental mycobacteria
Mycopbacterial as indicateors of pollution

8880 (Pickup et al., 2005)
In South Wales, United Kingdom, a populated coastal region lies beneath hill pastures grazed by livestock in which Mycobacterium avium subsp. paratuberculosis is endemic. The Taff is a spate river running off the hills and through the principal city of Cardiff. We sampled Taff water above Cardiff twice weekly from November 2001 to November 2002. M. avium subsp. paratuberculosis was detected by IS900 PCR and culture. Thirty-one of 96 daily samples (32.3%) were IS900 PCR positive, and 12 grew M. avium subsp. paratuberculosis bovine strains. .... Parallel studies showed that M. avium subsp. paratuberculosis remained culturable in lake water microcosms for 632 days and persisted to 841 days. Of four reservoirs controlling the catchment area of the Taff, M. avium subsp. paratuberculosis was present in surface sediments from three and in sediment cores from two, consistent with deposition over at least 50 years. Previous epidemiological research in Cardiff demonstrated a highly significant increase of Crohn's disease in 11 districts. These bordered the river except for a gap on the windward side. A topographical relief map shows that this gap is directly opposite a valley open to the prevailing southwesterly winds. This would influence the distribution of aerosols carrying M. avium subsp. paratuberculosis from the river.

8882 (Pickup et al., 2006)
Mycobacterium avium subsp. paratuberculosis from infected animals enters surface waters and rivers in runoff from contaminated pastures. We studied the River Tywi in South Wales, United Kingdom, whose catchment comprises 1,100 km^2 containing more than a million dairy and beef cattle and more than 1.3 million sheep. .... 48 of 70 (68.8%) twice-weekly river water samples tested positive by IS900 PCR. In river water, the organisms were associated with a suspended solid which was depleted by the water treatment process. Disposal of contaminated slurry back onto the land established a cycle of environmental persistence. .... 1 of 54 domestic cold water tanks tested positive, indicating the potential for these pathogens to access domestic outlets. In the separate English Lake District region, with hills up to 980 m, ... streams and sediments became positive lower down the catchment. Sediments from 9 of 10 major lakes receiving inflow from these catchments were positive,

with sediment cores indicating deposition over at least 40 to 50 years. Two of 12 monthly 1-liter samples of effluent and a single 100-liter sample from the Ambleside sewage treatment works were positive for M. avium subsp. paratuberculosis. .... there is a potential for these organisms to cycle within human populations.

8836 (Pedley et al., 2004)
Water remains an important potential source of human exposure to environmental mycobacteria. Organisms such as MAP, although difficult to recover from environmental samples, are excreted in large numbers in the faeces of infected animals and are likely to be present in source waters that are abstracted for drinking-water. Some species, such as M. kansasii, can colonize cold water distribution systems whilst M. xenopi and M. avium are more commonly associated with hot water systems. In contrast, M. marinum in swimming pools or aquaria can cause infection of skin abrasions. ..... M. avium is not a contaminant but is a normal inhabitant of drinking-water (von Reyn et al. 1994). Numbers of M. avium in recirculating hot water systems are increased relative to numbers in the input water (du Moulin et al. 1988) suggesting that M. avium is replicating in such systems in agreement with studies in natural water (George et al. 1980). .... mycobacterial growth occurs in the distribution system. ...... Swimming pools yield M. avium (Havelaar et al. 1985; Emde et al. 1992) and long-term exposure to aerosols results in a granulomatous pneumonitis in lifeguards 8835 (Rose et al. 1998). M. avium, M. intracellulare and other mycobacteria have been shown to be present in biofilms (Schulze-Röbbecke & Fischeder 1989; Iivanainen et al. 1999a; Falkinham et al. 2001). ...... the contribution of biofilm to the microbial flora of the drinkingwater is substantial. ...... The type of surface apparently had an effect on mycobacterial biofilm numbers. Numbers of M. intracellulare were 4400 cfu/cm^2 on brass or bronze surfaces compared to 70 cfu/cm^2 on plastic (Falkinham et al. 2001). .... M. avium can be recovered as cfu from droplets ejected from natural waters (Wendt et al. 1980). .....there is enrichment of M. avium and M. intracellulare in natural ejected droplets (~14 000-fold) resulting in considerable transfer of mycobacteria from water to air. ...... exposure to waters whose catchments included heavily grazed pastures was associated with conspicuous clusters of CD. The first of these involved the village of Blockley, a rural community of about 2000 people in Gloucestershire, England in which 12 people developed CD between 1960 and 1983, an increase of observed over expected (for that time) of 6.7-fold and equating with a CD incidence of 28/105 per year (Allan et al. 1986). The village, which had its own water supply from local springs, lay in a hollow surrounded by upland pastures grazed by cattle in which clinical JD was evident .... occurrence of 7 cases of CD amongst 285 graduates of the Mankato West High School class of 1980. All seven had been swimming in local ponds and lakes. ....There are also data which implicate domestic hot water systems. Two case control epidemiological studies carried out independently in the United Kingdom, each unexpectedly identified the availability of fixed hot water supplies in the early childhood home as a significant risk factor for the subsequent development of CD, but not for ulcerative colitis (Gent et al. 1994; Duggan et al. 1998).

0492 (Bolster et al., 2009)
The potential for groundwater contamination by MAP is low; however, the organism may remain bound to the soil near the surface where it can be ingested by grazing animals or be released during run off events. This is the first study looking at the surface characteristics and transport behaviour of Map through aquifer materials and therefore provides important information for understanding the movement of Map in the environment

8838 (Torvinen et al., 2004)
... the occurrence of mycobacteria in drinking water increased from the waterworks toward the distal portion of the system. There was a >2-fold increase, to 80%, in the isolation frequency and a 10-fold increase, to 140 CFU/liter, in the numbers compared to the levels at the waterworks. .... the numbers of mycobacteria in water were higher in the systems using ozone than in those not applying this agent. Ozone is known to degrade organic matter, increasing the fractions that can be assimilated by microbes (30, 51). our results support the recent result reported by Falkinham and coworkers (6) concerning the association between mycobacterial growth and AOC in the system. .... the number of mycobacteria in water also correlated with the iron content, a typical element accumulating in the Finnish systems. The iron content increased with water retention time, probably as a result of the

corrosion of iron pipes, as did the mycobacterial number. .... Our results indicate that mycobacteria may multiply in the distal portions of the system, ... the key site of mycobacterial occurrence in the systems lies in the deposits. Mycobacteria were found in all deposit samples analyzed, and their numbers there, if expressed per liter, exceeded those in the water samples by a

factor of 2.5 x 104. .... the high occurrence of mycobacteria in drinking water pipeline biofilms, and the reported numbers are very similar to those of the present study (10^2 to 10^3 CFU/cm^2). In conclusion, mycobacteria are common in boreal drinking water systems, especially in the deposits.

8840 (Whan et al., 2005)
Of the 192 untreated water samples tested, 15 (8%) tested M. avium subsp. paratuberculosis positive by one or more of the three detection methods. M. avium subsp. paratuberculosis was successfully isolated from eight untreated water samples, three by BACTEC culture and five by culture on HEYM. This work indicates the need to determine the efficacy of water treatment processes to either kill or remove M. avium subsp. paratuberculosis from untreated water and the possible risks posed by contact with recreational water sources.

0533 (Chia-Wei et al., 2009)
Experts estimate that Johne's disease results in a $220 million loss per year in the USA alone [22]. Currently, there is no effective control strategy for Johne's disease and infection with M. ap is hard to diagnose and monitor because of the chronic nature of the disease. Additionally, it is very difficult to remove M. ap from the environment, which threatens any effective control strategy. In recent studies, M. ap was shown to be ubiquitous in animal environments [11,23], especially among wildlife animals [6]. One reason M. ap may be difficult to eliminate from the environment and to treat with antibiotics is the possibility that the M. ap bacilli may form biofilm-like structures. ..... Biofilm formation is considered a virulence phenotype .... M. avium, a bacterium closely related to M. ap, was found to form biofilms especially in water pipe systems of large cities

8807 (Huttunen et al., 2000)
Mycobacterial strains (nonpathogenic Mycobacterium terrae, potentially pathogenic Mycobacterium avium-complex and Mycobacterium scrofulaceum), isolated from a moldy building, were studied with respect to their ability to stimulate macrophages (RAW264.7) to produce inflammatory mediators, and to cause cytotoxicity. Reactive oxygen species (ROS) were measured by chemiluminescence, cytokines (TNF-a, IL-6, IL-1, IL-10) immunochemically, nitric oxide (NO) by Griess-method, expression of inducible NO-synthase (iNOS) with Western Blot analysis and cytotoxicity with MTT-test. All the strains induced dose- and time-dependent production of NO, IL-6 and TNF-a in macrophages, whereas IL-1 or IL-10 production was not detected. .... Environmental mycobacteria include both potentially pathogenic and saprophytic species, which share the unique feature of all mycobacteria; a thick, hydrophobic and lipid-rich cell wall, also containing serologically active polysaccharides (Falkinham, 1996; Chatterjee and Khoo, 1998; Goodfellow and Magee, 1998). The cell wall structure enables mycobacteria to survive and even proliferate in extreme environments, such as in areas contaminated with xenobiotics or inside of host macrophages. The pathogenicity of mycobacteria such as the notorious Mycobacterium tuberculosis and Mycobacterium leprae is based on the immunological responses they elicit in the host, beginning with an ineffective elimination of the bacteria, and ending with the tissue-damaging hypersensitivity reactions (Clark-Curtiss, 1998). Some environmental mycobacteria have also been recognized as effective immunostimulators in murine models (Bradbury and Moreno, 1993; Denis and Ghadirian, 1994; Falcone et al., 1994; Sarmento and Appelberg, 1995). Mycobacteria present in moldy buildings are of special interest, because they are inhalable particles and have potential to stimulate a cascade of cytokines, leading to recruitment and activation of inflammatory cells. .... exposure to environmental mycobacteria, isolated from moldy houses, initiates a cascade of events leading to production of immunoactive and reactive compounds, and eventually cell death in murine RAW264.7 macrophages. If this is the case also in vivo, environmental mycobacteria are an important group of microbes, which may contribute to the adverse health effects observed among the inhabitants of the buildings with moisture and mold damage.

0294 (Tarver, 2009)
..... biofilms can affect virtually any industry that involves a water-based process: nautical shipping, paper manufacturing, cooling systems, drinking water facilities, health care, medical devices, and food processing (MSU, 2008a, 2008b). Although biofilms can contain only one single-celled species, it is more common for them to comprise many bacterial species. Unlike humans from different ethnicities who often struggle to understand each other, the different bacteria forming a biofilm communicate well with each other. Through cell-to-cell signaling known as quorum sensing, bacterial cells coordinate biofilm formation. Biofilms enable bacteria to survive unpredictable environmental stressors such as temperature changes, desiccation, ultraviolet rays, and so on. The formation of a biofilm occurs in five stages: In the first stage, bacterial cells use van der Waals forces to attach to a surface (MSU, 2008a). In stage 1, cell attachment is still reversible, but in stage 2 the cells affix themselves more securely by

forming exopolymeric material, which is a stronger adhesive compound. In stage 3, micro-colonies begin to form, and the biofilm begins to mature. Stage 4 involves more maturation, and the biofilm becomes a three-dimensional structure containing cells packed in clusters with channels running between them. And lastly, in stage 5 the biofilm disperses cells so that they can move on to initiate the formation of new biofilms. It is important to note that cell division is uncommon in mature biofilms. In the mature state, biofilm cells use energy predominantly to produce exopolysaccharides, which the cells use as nutrients (Watnick and Kolter, 2000). .... biofilm cells can detach and inoculate other model food systems (Midelet and Carpentier, 2004). Not surprisingly, once biofilms form, they are difficult to eliminate. Besides causing tooth decay, slippery rock surfaces, and contaminated water, these colonies of microbes may also cause persistent low-level food contamination (Annous et al., 2009). In fact, biofilms containing human pathogens can impair food safety. ....

PART FIVE

CROHN'S DISEASE AND BREASTFEEDING

Notable associations

8874 (Bergstrand and Hellers, 1983)
Crohn's disease patients were particularly overrepresented among those with no or very short periods of breastfeeding. **Published in 1983.**

8870 (Klement et al., 2004)
A meta-analysis was performed on 17 relevant articles that were found by using MEDLINE, EMBASE, the Internet, and articles' references. The results of this meta-analysis support the hypothesis that breastfeeding is associated with lower risks of Crohn disease and ulcerative colitis.

8871 (Schack-Nielsen et al., 2005)
Breastfeeding has been reported to reduce the risk of Crohn's disease and colitis ulcerosa, but it is not know if BF is of major importance for development of these diseases (Davis, 2001; Klement et al., 2004). Also protection against development of multiple sclerosis, rheumatoid arthritis by BF has been reported (0007 Hanson et al., 2001). .... Type 1-diabetes mellitus (DM) is caused by both genetic and environmental factors. A number of studies have shown a protective effect of BF, whereas introduction of formula milk and complementary food seems to increase the risk (Davis, 2001).

8842 (Sonntag et al., 2007)
Exposure to bacterial antigens in combination with a genetically determined dysregulation of the mucosal immune response is one of the potential pathogenic trigger mechanisms.[2,4] Among others, the circumstances at birth, as one of the earliest influencing factors, have dramatically changed over the past years. First, the rate of cesarean section deliveries has more than doubled during the past 20 years and this trend seems to continue.[5] Second, advances in modern neonatal medicine have increased the survival chances of prematurely born infants. ...... breastfeeding has been associated with differences in the microbial flora of infants,[8] and in allergy and atopic disease breastfeeding is generally regarded as a preventive factor.[9] Given the attributed role of bacteria in the development of IBD we hypothesized that specific circumstances around birth such as mode of delivery and preterm birth, through influencing the naive intestinal immune system at a very early stage, can present risk factors for the individual predisposition of developing IBD later in life. .... A recent meta-analysis cautiously supports a protective role of breastfeeding in CD as well as UC.[22] Besides providing immunological protection to the newborn, breastfeeding might reduce a stimulatory effect of bacteria or endotoxins on the immune system either by direct reduction of exposure or by passive transfer of immune responses from the mother, possibly leading to immature immune response mechanisms in the infant. [23] Moreover, a putative protective effect of breastfeeding might be biased by various factors such as environmental antigen exposure, reactions to cow's milk in the mother's diet, or the concentration of IgE in the mother.
The protective effect of breastfeeding or an adverse effect of formula feeding and early stage influence of triggers?

0005 (Shanahan, 2002)
.... striking increase in frequency of Crohn's disease within the more-developed world over the past 50 years, and the disease's increased recognition with progressive industrialisation in less-developed countries.

8856 (Economou and Pappas, 2008)
In Canada the incidence of the disease in the province of Manitoba is characteristically lower in Indian aboriginals; this discrepancy raises questions about its background, i.e., is it related to a different genetic profile or to the lower hygiene standards of this population (although in the latter case one

would expect the opposite effect on CD incidence)? Nevertheless, even in this population a recent increase has been noted, especially in the 30–40 age group.

8827 (Dubinsky et al., 2008)
Potential environmental contaminants in the breast milk and inappropriate immune responses from delayed infections at weaning, as well as other factors such as genetic background, duration of breastfeeding, and use of supplemental formula versus exclusive breastfeeding, will need to be further delineated. ..... The short- and long-term implications of placental and postnatal exposure via lactation to babies born to mothers treated with immunomodulators such as 6MP/AZA or anti- TNF therapies is relatively unknown and has become an important area of investigation. .....

8843 (de Mesquita et al., 2008)
A report from Scotland described three-fold increase in childhood CD between 1968 and 1983, the incidence of UC remained unchanged during this period, which would seem to parallel the increase seen in adults. A similar increase in incidence was also reported from various other European countries such as Wales [13], North France [14], Brittany [15], Great Britain [12], Norway [16], Sweden [17] and the USA [18]. .... The hypothesis that exposure to a specific infectious agent could predispose to IBD was put forward as early as 1913, when Dalziel proposed Mycobacterium paratuberculosis as the agent for Crohn's disease [55]. Since then, smoking, appendicitis, gastrointestinal infections, bottle feeding, and better hygiene during childhood have been implicated as risk factors or protective factors for UC and CD [56–58]. .... infections acquired during the immediate perinatal period or early infancy may establish aberrant immune responses to microbes encountered at a later time, while early-to-mid-childhood infections may promote tolerance to like organisms. .... There is evidence that IBD is immunologically mediated and that genetic factors play an important aetiological role. However, the dynamic epidemiology of both conditions suggests that extrinsic environmental factors acting at the population level may be involved in its pathogenesis. Furthermore, the remarkable increase in the incidence of IBD, and CDin particular, during the last half century points to changes in the environment, since genetic variations are negligible in such a short period of time, and the "hygiene hypothesis" of allergic and autoimmune diseases has been invoked to explain the world-wide spreading of IBD, also in non-western parts of the world as Asia and South-America. Identification of susceptibility genes has highlighted the importance of the innate immune system, and specifically, abnormal detection or handling of gut bacteria as a cause for Crohn's disease. If we are to prevent IBD, we clearly need to be able to identify the enigmatic environmental factors that cause the disease.

8828 (Goldfarb, 2008)
If a newborn is not breastfed, formula, a modification of cow's milk, is the sole source of nutrition. The possibility of antigenic cross reactivity with the protein glycodelin which could influence autoimmune activity needs to be investigated. ..... A large controlled case study in Finland (4) concluded that the age of introduction of dairy products is associated with risk of type-1 diabetes mellitus. ...... Glycodelin has three well-defined isoforms, (17) glycodelin-A, glycodelin-S, and glycodelin-F. All have the same protein sequence, but different carbohydrate moieties. This suggests the glycans mediate the biological actions.

8830 (Banwell et al., 2008)
.... reactivity to one or more milk antigens was common in children with autoimmune disease, the presence of such reactivity did not appear to reflect a general loss of tolerance to dietary proteins, **It could be a result of formula feeding** .... Although reactivity to one or more milk antigens was common in children with autoimmune disease, the presence of such reactivity did not appear to reflect a general loss of tolerance to dietary proteins, ... T-cell–mediated responses are viewed as important contributors to the causative and active inflammatory injury of target tissues in autoimmune diseases such as MS and T1D. ..... One interpretation of our observations is that antigenic mimicry between particular dietary-milk and self antigens may represent an early disease mechanism shared by patients with CIS/MS and with T1D. ...... However, an alternate interpretation of our findings is that induction of autoimmune disease and subsequent tissue injury may promote T-cell populations that can also cross-recognize environmental antigens. .... Our study highlights the challenges involved in

efforts to elucidate the relation between dietary antigens and autoimmunity. In both human T1D and its rodent models, heightened cow-milk reactivities have been reported (19,33,34) and linked to observations that delayed weaning to foreign protein diets may protect T1D-prone hosts from disease(12,34). This hypothesis currently is being tested in a global diabetes prevention trial, TRIGR, that compares weaning to standard versus severely hydrolyzed, nonantigenic infant formula (15).

Although similar observations are unavailable for MS, high milk consumption was proposed as a possible MS risk factor years ago. ..... children with CIS, MS, T1D, and CNS injury harbor circulating T cells that exhibit heightened reactivities to a range of self-antigens, and that these abnormal autoantigen responses are not restricted to the target tissue of the disease. Our findings may point to a relatively nonspecific abnormality of T-cell regulation as an early feature of both CIS/MS and T1D, and highlight the relation between tissue injury and autoimmunity. The coexistence of a relatively nonspecific dysregulation of T-cell responses, combined with additional host and environmental factors, may then determine the emergence and possibly also the target-organ selectivity of autoimmune disease.

8831 (Dow, 2006)
Several studies indicate an association between early exposure to dietary cow's milk proteins and an increased risk of T1DM [45–47]. These studies have centered around the observation that children at risk for T1DM who were breast fed exclusively for more than six months were less likely to have T1DM later in life than similar risk children who were weaned onto cow's milk-based formula at an earlier age. .... there is something about cow's milk protein that is an immunologic trigger for T1DM .... This paper postulates that Mycobacterium avium ss. paratuberculosis (MAP) acts as an immune antigen in the pathogenesis of T1DM. As the link between MAP and Crohn's disease becomes more compelling, MAP is increasingly recognized for its ability to act as an occult antigen. Genetic evidence suggests that there are states of macrophage dysfunction that promote both T1DM and mycobacterial infection. These states can be viewed as templates of macrophage incompetence that individually or in combination allow obligate intracellular pathogens such as MAP to persist and serve as immune antigens. The epidemiologic association of T1DM with early exposure to cow's milk has prompted the large TRIGR study. The hypothesis offered here is that Mycobacterium avium ss. paratuberculosis acts as an immune antigen, a trigger, of T1DM.

8864 (Rigas et al., 1993)
With respect to Crohn's disease ... only breasfeeding has a dose-depandent statistically significant negative association that could be interpreted as reflecting a protective effect of breastfeeding on Crohn's disease risk.

8826 (Mikhailov and Furner, 2009)
Human breast milk contains many substances that may influence growth and development as well as function of the gastrointestinal tract. Some of these factors may have age-dependent effects [67]. Furthermore, the composition of colonic flora differs between breastfed and bottle-fed infants [68]. IBD pathogenesis is presumed to be a complex interaction between genetic predisposition and inappropriate activation of the mucosal immune system driven by the presence of enteric flora and resulting in tissue injury [1-3]. Thus, it seems quite plausible that breastfeeding would have a protective effect on the development of IBD in genetically predisposed individuals, at least in childhood. **In other words: Bottle feeding could have an adverse effect.**

8860 (Hanson et al., 2002)
..... most recently a critical review of published reports on breastfeeding and allergy reveals solid support for the concept that breastfeeding protects, at least for the first decade of life. This effect is most obvious in high-risk children

0007 (Hanson et al., 2001)
Recent data confirm that breastfeeding decreases the risk of developing coeliac disease in childhood (45.). Single studies also suggest preventive effects on Crohn's disease and ulcerous colitis. Similar single observations also exist for multiple sclerosis and rheumatoid arthritis.

8861 (Corrao et al., 1998)
..... several reports showed that IBD patients were breastfed for comparatively shorter periods, if at all. In the present study, we found that lack of breastfeeding during infancy was positively associated with the risk of CD and UC.

8846 (Joossens et al., 2005)
a significant association was found between the month of birth and later development of IBD; a significant reduced risk to develop CD was observed for people born in June. Moreover, environmental yearly reoccurring factors during pregnancy or postpartum might be associated with the occurrence of CD later in life.

8844 (Sorensen et al., 2001)
We found a seasonal pattern in month of birth in this nationwide cohort of people with Crohn's disease in Denmark.We expect that variation in seasonal prevalence of an infectious agent, sensitivity of a fetus to the agent, subsequent events affecting susceptibility, and development of Crohn's disease would all attenuate any observable seasonality. Despite such attenuation our data provide some evidence that the occurrence of Crohn's disease in childhood may result in part from experience with one or more infectious agents operating in utero or early in childhood.

8847 (Chowers et al., 2004)
It was suggested that perinatal exposure to infectious agents may have a role in the pathogenesis of CD. Due to the seasonal nature of some infections, a linkage between birth dates and a risk to develop CD would support such a hypothesis. Birth during the winter period (NOV to FEB) in Israel was associated with increased risk to develop CD.

8846 (Joossens et al., 2005)
In this Belgian cohort, a significant association was found between the month of birth and later development of IBD; a significant reduced risk to develop CD was observed for people born in June. Moreover, environmental yearly reoccurring factors during pregnancy or postpartum might be associated with the occurrence of CD later in life.

8862 (Gearry et al., 2006)
The crude incidence rates of 25.2 per 100,000 per year, 16.5 per 100,000 per year, and 7.6 per 100,000 per year for IBD, CD, and UC in Canterbury for 2004 are at the upper end of those described in Western countries previously, The highest incidence of CD reported in the medical literature is 15.6 per 100,000 person-years from Manitoba in Canada between 1987 and 1996. .... As with other epidemiologic studies of IBD from New Zealand, this study shows that IBD is rare in Maori and Pacific Islanders. It is difficult to know what, if any, genetic or environmental factors may protect these groups from IBD, and it will be interesting to observe whether IBD incidence increases rapidly in these groups as it has in other nonwhite populations. This also presents interesting opportunities for genetic admixture mapping studies. Previous IBD epidemiology studies in New Zealand have suggested that IBD was less common in New Zealand than elsewhere. This is not the case in Canterbury, where the incidence and prevalence of CD are very high. .... However, ethnic and possibly undefined environmental factors are also likely to have a significant effect on rates throughout the New Zealand. This cohort provides a unique opportunity to study etiologic factors, particularly those of an environmental and genetic nature, in a population-based cohort. **What about to seek factors different for Maori and other population, which do not protect the former but trigger IBD in the latter. Could anybody know the differences betwen ethnics in breastfeeding and bottle feeding?**

8863 (Griffiths et al., 2007)
A total of 18 150 natural mothers (11 286 (8207 white) living in England) of singleton infants. ....
White mothers were more likely to discontinue breast-feeding (62%) and introduce solids early (37%) than most other ethnic minority groups;

PART SIX
MUCOSAL IMMUNITY
Possible modifications during postnatal development

0003, 8815, 8814, 0022, 8816
(Singh et al., 2001; Hudcovic et al., 2001; Tlaskalova-Hogenova et al., 2004; Uhlig et al., 2006; Stepankova et al., 2007)
Starting from first hours after the delivery from sterile uterine environment the interaction of the macroorganisms with microorganisms begins: the main portal of entry of microbes is skin and mucosal surfaces of the gastrointestinal, respiratory and urogenital tracts. Interaction with commensal bacteria which outnumber the cells forming our body occurs physiologically on epithelial surfaces and is usually balanced. Our studies have shown that intestinal microbiota and their components play a crucial role in the development of the mucosal immune system. However under some conditions the interaction of commensal bacteria with the host could be harmfull. ..... Animal models are helping to elucidate the etiology and pathogenetic mechanisms of human diseases. An unvaluable tool for studying role of microflora in the development of IBD is represented by experimental models reared in germfree conditions and colonized by defined bacterial mixtures or strains. Intestinal inflammation could develop as the consequence of abberant immune reactions to gut microbiota in the early postnatal period. Identifying the components of commensal bacteria (resident or transient) involved in intestinal inflammation could help in finding tools for new approaches to prevention and therapy of human IBD.

0022 (Uhlig et al., 2006)
The immune pathogenesis of CD is associated with increased inflammatory cytokines including tumor necrosis factor-a (TNF-a), interferon-g (IFN-g), and interleukin-12 and interleukin-23 p40 (IL- 12p40, IL-23p40). ...... The IBDs are of complex multifactorial pathogenesis and involve the activation of the innate and adaptive immune system. Animal models have suggested T cell-dependent and -independent mechanisms of intestinal inflammation (Strober et al., 2002). In IBD, there is evidence that alterations in the innate immune response contribute to disease development because mutations in the NOD2 gene, which lead to abnormal function of this innate pathogen recognition receptor, confer susceptibility to CD in some patients (Eckmann and Karin, 2005). .... Through neutralizing antibody and gene ablation studies, we now show that IL-23 is the key driver of T cell-independent mucosal inflammation.

8814 (Tlaskalova-Hogenova et al., 2004)
There is no doubt that not only living and multiplying bacteria but also their components which are expressed, secreted and could be released from bacterial body after microbial death are responsible for various immunomodulatory effects. Only some of them could be mentioned: lipopolysaccharides (endotoxins), peptidoglycans, bacterial CpG–DNA motifs, heat shock proteins and superantigens.

8857 (Rook et al., 2004)
The increase in disease incidence is not confined to allergic disorders. In the rich, developed parts of the world, there has been a steady and simultaneous increase in at least three groups of disease: (1) allergies, (2) inflammatory bowel diseases (IBD; e.g. Crohn’s disease and ulcerative colitis) [63, 83], and (3) autoimmunity (e.g. type 1 diabetes and multiple sclerosis) [6]. There is also a North-South gradient in the incidences of these diseases, all of which tend to be more common in Northern Europe [6]. However these areas of medicine are compartmentalised, and while allergy specialists were suggesting that Th2- mediated diseases might be increasing in developed countries because of diminished exposure to Th1-inducing infections, other workers interested in the increase in Th1-mediated autoimmune diseases such as type 1 diabetes and multiple sclerosis, were coming to precisely the reverse conclusion, and suggesting that there was excessive activity of Th1 cells, and that consequently autoimmunity might be treated by enhancing Th2 responses. The latter concept has been applied to Crohn’s disease, since this also is predominantly Th1 mediated [26]. More recently,

however, it has been recognised that the increases in allergies and type 1 diabetes are precisely correlated both within Europe and outside Europe [88]. If Th1-mediated and Th2-mediated diseases are increasing in parallel, the increases cannot have anything to do with changes in Th1/Th2 balance, so another explanation must be sought. Regulatory cells (Treg) can provide such an explanation. ....

The notion that exposure to microorganisms can modulate susceptibility to all three groups of immunoregulatory disorder (whether Th1 or Th2 mediated) is not new. .... Thus background exposure to harmless micro-organisms can influence Th1 mediated autoimmunity. It seems probable that certain organisms that have been present and harmless throughout mammalian evolutionary history are now recognised by the innate immune system as “old friends” and fail to trigger destructive responses. The absence of colitis following introduction of lactobacilli into the guts of IL- 10`/` mice is a nice example [84]. We suggest that the “old friends” have a particular ability to trigger rapid Treg responses.

0516 (MacDonald and Monteleone, 2005)
The gut immune system has the challenge of responding to pathogens while remaining relatively unresponsive to food antigens and the commensal microflora. In the developed world, this ability appears to be breaking down, with chronic inflammatory diseases of the gut commonplace in the apparent absence of overt infections. In both mouse and man, mutations in genes that control innate immune recognition, adaptive immunity, and epithelial permeability are all associated with gut inflammation. This suggests that perturbing homeostasis between gut antigens and host immunity represents a critical determinant in the development of gut inflammation and allergy. .... In westernized countries, most infectious diseases of the gut are largely under control, yet gastrointestinal food allergies and idiopathic inflammatory conditions have dramatically increased; in other words, we now have inflammation without infection.

0198 (Qian et al., 2008)
Components of commensal enteric microorganisms are potent stimuli for both innate and adaptive immune responses. The initial sensing of bacterial stimuli by the host is mediated by a class of transmembrane pattern recognition receptors designated Toll-like receptors (TLRs) that are expressed on a variety of cell types, most notably innate immune cells.2 Various TLRs recognize diverse bacterial products. TLR2 is activated by peptidoglycan-polysaccharide (PG-PS), a bacterial cell wall component. Lipopolysaccharide (LPS) is a glycolipid derived from the outermost membrane of Gram-negative bacteria, which induces signaling through TLR4. Flagellin, the structural component of flagella produced by many Gram-negative bacteria, mediates its effects through TLR5. CpG-containing bacterial DNA oligonucleotides bind TLR9. In addition, the intracellular pattern recognition receptor NOD2/CARD15 is activated by bacterial muramyl- dipeptide (MDP). Ligation of each of the TLRs or NOD2/CARD15 activates NF-_B and induces proinflammatory gene expression, which subsequently controls activation of adaptive immune responses.3,4 Altered host–microbial interactions are implicated in experimental colitis and IBD. This is underscored by the finding of an association of NOD2/CARD15 mutations and Crohn’s disease (CD).5,6 Variants in TLR2 and TLR4 also confer susceptibility to both CD and ulcerative colitis.7–9 Patients with IBD display immunological reactivity against commensal luminal flora and their components.10,11 In line with the in vivo observations from human studies, we and others have reported that lysates of cecal bacteria, containing a highly complex assortment of bacterial antigens and adjuvants, can selectively activate intestinal and mesenteric lymph node T helper (Th) 1 cells and accessory cells (non-T cells) to secrete a group of proinflammatory cytokines including IFN-gama, IL-12, and TNF, in colitic HLA-B27 transgenic (TG) rats,12,13 IL-10 deficient mice,14 and C3H/HeJBir mice.

8879 (Tang and Ho, 2007)
Developmental epigenetics is believed to establish “adaptive” phenotypes to meet the demands of the later-life environment. Resulting phenotypes that match predicted later-life demands will promote health, while a high degree of mismatch will impede adaptability to later-life challenges and elevate disease risk. ...... Epidemiologic studies now support an early origin of adult human diseases. .... understanding how environmental factors influence various epigenetic processes during developmental reprogramming should provide new insights into early diagnosis, prevention, and

treatment of these diseases. .... The fact that the epigenetic “memory” can be transmitted to daughter cells and across several generations, influence sexual behavior, and thus impact Darwinian evolution makes this a field of investigation that should be highly valuable for generating new insights into the basis of disease development.

8813 (DeKoter et al., 1998)
Hematopoiesis can be viewed as a patterning process in which a self-renewing, pluripotent stem cell (HSC) gives rise through a series of cell divisions to the eight major cell types of the blood. During its differentiation, the HSC appears to generate a hierarchical array of developmental intermediates, consisting of multipotent and lineage-committed progenitors. The latter cells give rise to erythrocytes, megakaryocytes, mast cells, granulocytes (e.g. neutrophils), macrophages, and B and T lymphocytes. ...... PU.1 is a unique regulatory protein required for the generation of both the innate and the adaptive immune system. It functions exclusively in a cellintrinsic manner to control the development of granulocytes, macrophages, and B and T lymphocytes. We demonstrate that mutation of the PU.1 gene causes a severe reduction in myeloid (granulocyte/macrophage) progenitors. ....

0002 (Ruemmele et al., 2006)
Pediatric epidemiological data from the UK, northern France, Sweden and the US (2–6) indicate an increased incidence for pediatric Crohn disease (CD) with a stable incidence of pediatric ulcerative colitis (UC). .... Some experimental and in vivo animal data indicate regulatory defects within the innate immune system as causal in the development of IBD (11–13). .... The first months of life are a critical and vulnerable period in the initiation of a normal host immune response toward exogenous aggressions. ... the number of pediatric IBD patients steadily increasing but also affected children are increasingly younger. One major factor is a particular genetic predisposition to develop IBD, however, genes do not significantly change within 1 or 2 generations. Therefore, it is more likely that environmental trigger factors initiating the disease evolved over the years.

8845 (Begue et al., 2006)
There is increasing evidence that a breakdown of the physiological tolerance towards non-pathogenic luminal bacteria plays a key role in the pathogenesis of Crohn’s disease (CD). With the recent identification of CARD15 as the first susceptibility gene for CD significant advances in the molecular understanding of this breakdown were made (Hugot et al., 2001; Ogura et al., 2001a). CARD15 is part of the innate immune system ..... we demonstrate for the first time that CARD15 is easily inducible and functional upon bacterial stimulation, indicating that bacteria are implicated in the control of the innate immune system on the level of IEC: the simple physical contact with a non-pathogenic bacterium is sufficient to induce CARD15 in IECs, similar to the effect of pro-inflammatory cytokines.

0001 (Cash et al., 2006)
Paneth cells are key effectors of small intestinal antimicrobial defense. These specialized epithelial cells are located at the crypt base and harbor abundant cytoplasmic secretory granules containing antimicrobial proteins, including a-defensins. To gain new insights into how intestinal surfaces cope with microbial challenges, we used DNA microarrays to identify Paneth cell antimicrobial factors whose expression is altered by bacteria. Paneth cells were harvested by laser capture microdissection from Bgerm-free[(microbiologically sterile) mice and Bconventionalized[mice (germ-free mice reconstituted for 10 days with an intestinal microflora from conventionally raised mice). Paneth cell mRNAs from both groups were amplified to generate complementary RNAs (cRNAs) in sufficient quantity to hybridize to Affymetrix mouse genome 430 2.0 GeneChip arrays. The results of our screen revealed 149 transcripts whose expression was changed 2- to 45-fold by microbial colonization .... One of the most prominent responses uncovered by our analysis was a 31-fold increase in the abundance of RegIIIg transcripts in Paneth cells from conventionalized as compared with germ-free mice .... findings suggested that RegIIIg binds peptidoglycan, a molecule that is exposed on the Gram-positive bacterial surface, We also assayed for changes in RegIIIg mRNA expression during postnatal intestinal development. RegIIIg mRNA levels rose dramatically during the weaning period .... Weaning is associated with dramatic changes in gut microflora composition, as well as withdrawal of maternal immunoglobulin A (IgA) antibodies. .... Our results suggest that RegIIIg expression is triggered by increased microbial epithelial contact at mucosal surfaces. Enhanced

expression of Reg proteins such as HIP/PAP in IBD patients may therefore be a compensatory response that limits mucosal penetration by gut microbes. .... It is not yet known whether Reg expression is triggered by direct bacterial interactions with gut epithelia, or whether other intestinal cells (e.g., macrophages) direct epithelial Reg expression. Further investigation will therefore be required to decipher the host and microbial factors that regulate antimicrobial lectin expression. These studies will contribute to a better understanding of IBD pathogenesis and will provide new insights into how symbiotic hostmicrobial relationships are maintained.

0281 (Rajaiah and Moudgil, 2009) **Important review.**
Heat shock protein Hsp65 has been linked to the induction and perpetuation of several immune-mediated diseases (Table 1). The priming of humoral and/or cellular immune response against microbial or self Hsp is a critical component of the disease-related immune events in these disorders. ...... , three of the proposed mechanisms are discussed below. **Activation of innate immunity:** Hsps can activate macrophages and dendritic cells (DC) and these early innate responses in turn can be funneled into and direct the type of adaptive immune response to Hsp65. Furthermore, antibodies to Hsp65 and immune complexes consisting of Hsp65 and anti-Hsp65 antibodies can trigger potentially pathogenic effector mechanisms via activation of the complement system. **Stress-induced Hsp expression as well as altered antigen processing and presentation:** The expression of endogenous Hsps is significantly increased when cells are exposed to an inflammatory environment or other stressors. For example, Hsp65 expression can be enhanced by various atherogenic chemicals and other risk factors. These self Hsps may further propagate the ongoing inflammation, and also constitute an attractive target for T cells and antibodies induced by foreign Hsps. In addition, inflammation can alter the antigen processing of Hsps to reveal certain epitopes more efficiently or display neodeterminants, including hidden (cryptic) epitopes, which can prime an immune response leading to immune pathology. **Molecular mimicry:** Hsps are highly conserved proteins that are good immunogens as well. Hsp65 family is among the most highly conserved families of proteins with more than 97% homology among prokaryotic Hsp65s, and more than 70% homology between prokaryotic and human Hsp65. Moreover, microbial Hsp65s possess B- and T-cell epitopes that are cross-reactive with self Hsp65s. Accordingly, the T cells and antibodies induced by microbial Hsps may target the related self Hsps and lead to tissue damage via ‘molecular mimicry’. .... Immune response to Hsp65 has been invoked in the pathogenesis of psoriasis, autoimmune intestinal inflammation, autoimmune demyelination (e.g., experimental autoimmune encephalomyelitis; EAE), Kawasaki disease, Behcet's disease, juvenile dermatomyositis and Sjögren's syndrome. The precise etiology of many of these diseases is not fully clear, but there is evidence for an autoimmune reaction stimulated by an infectious agent or an environmental toxin. Adoptively transferred CD8+ T cells originally primed by microbial Hsp65 but crossreactive with self Hsp65 can induce autoimmune intestinal inflammation in mice, and the immune pathology involves recognition of self Hsp65 expressed in the intestine. The inflammation is restricted primarily to the small intestine, and the disease can be induced in germ-free mice. ...... these examples, along with those of autoimmune diseases discussed above in more detail, illustrate the broad impact of Hsp65 immunity not only in mediating tissue inflammation, organ pathology, and clinical disease, but also in inducing remission from and offering protection against inflammation associated with autoimmunity and other diseases.

0524 (Gutierrez et al., 2009)
A statistically significant increase in all Th1-derived cytokines in CD but not in UC patients with the presence of bactDNA was observed in comparison with patients without bactDNA and controls. Conclusions: BactDNA is present in IBD patients, irrespective of their disease activity. This fact is associated with a marked Th1-driven immune reaction in CD patients, even in those in remission. Whether bactDNA is inducing or is favored by an increased inflammatory scenario in these patients remains under discussion.

0214 (Silk et al., 2008)
Structural and functional aspects of lipid presentation by CD1 molecules are presented in the context of the function of CD1-restricted T cells in antimicrobial responses, antitumor immunity, and the regulation of the tolerance and autoimmunity immunoregulatory axis. Particular emphasis is on invariantNKT(iNKT) cells and their ability to modulate innate and adaptive immune responses. .....

To date the only characterized exogenous CD1a-restricted lipid antigen is DDM (Moody et al. 2004) (Figure 1a). Initial experiments demonstrated that a lipid fraction derived from Mycobacterium tuberculosis was capable of sensitizing responses in a CD1a-restricted fashion (Rosat et al. 1999). Subsequent experiments led to the identification of the antigenic lipopeptide DDM, consisting of a complex peptide linked via acylation of a lysine moiety to a single alkyl chain of approximately 20 carbons (Moody et al. 2004). Because this group of lipopeptides, defined as mycobactins, is widely produced by mycobacteria, DDM-related structures may also be presented to CD1-restricted T lymphocytes. ..... The first CD1c-restricted lipid was isolated from the cell wall of both Mycobacterium avium and M. tuberculosis (Moody et al. 2000) (Figure 1c). Mycobacteria-specific T cell lines recognized two previously unknown mycobacterial hexosyl-1-phosphoisoprenoids and structurally

related mannosyl-1-phosphodolichols. Responses to mannosyl-1-phosphodolichols were common among CD1c-restricted T cell lines and peripheral bloodTcells of human subjects recently infected with M. tuberculosis but were not seen in naive control subjects (Moody et al. 2000). These antigens contain a single fully saturated alkyl chain that is similar to isoprenoid lipids, with methyl branches at every fourth carbon, and are referred to as mannosyl phosphoisoprenoids. Mycobacteria-specific T cells also recognized purified natural Mycobacterium smegmatis– derived β-d-mannopyranosyl-1-phosphoheptaprenol released by treatment of mycolated phospholipid (Moody et al. 2000).

0157 (van Kooyk and Rabinovich, 2008)
Glycans synthesized by prokaryotic and eukaryotic pathogens (for example, meningococci, Trypanosoma and Helicobacter) can include terminal glycan structures that are similar to those found in mammalian glycans (so-called 'molecular mimicry'). However, some pathogens, including Schistosoma mansoni and Mycobacterium tuberculosis, may also express specific glycan epitopes. ..... Within the immune system, various classes of glycan-binding receptors (lectins) exist that recognize specific glycan structures presented on a protein backbone or lipid structure. These receptors are secreted or are found on the cell surface of immune cells. ..... In several inflammatory autoimmune diseases, such as systemic lupus erythematosus, effector T cell populations with altered T cell glycosylation, especially those exposing terminal GalNAc and Gal-β(1-4)- GlcNAc structures, are highly upregulated. .... O-glycans are also the chief components of the intestinal mucus layer that covers the gastrointestinal epithelium and protects mucosal tissue from potentially harmful pathogens. It has been recently found that deficiency of the core 3 transferase C3GnT results in elimination of core 3–derived O-glycans and presentation of naked Tn antigen in mouse colonic epithelium. C3GnT-deficient mice have an increased susceptibility to inflammatory bowel disease and colorectal adenocarcinoma, suggesting that core 3–derived O-glycans are key components of intestinal mucin and that disruption of these structures is associated with the initiation and perpetuation of intestinal inflammation.

0196 (Wen et al., 2008)
...... interaction of the intestinal microbes with the innate immune system is a critical epigenetic factor modifying T1D predisposition.

8878 (Rousseau et al., 2008)
there is increasing, but still inconsistent evidence that vaccinations and childhood infections may play a role in the normal maturation of the immune system, and in the development and balance of immune regulatory pathways. Several epidemiological studies have focussed on a possible link between BCG vaccination and the development of asthma and allergic diseases, but this issue remains unclear. Much fewer investigations have addressed chronic diseases just as pertinent because of their inflammatory and autoimmune characteristics, such as IDDM, CD, and cancer. Despite null results in a majority of studies, the evidence for an association observed in some populations deserves to be further explored. There appear to be several chronic conditions for which a modulation of the immune function could play an aetiological role. Altogether, this raises the possibility that common or related immunological mechanisms could be involved in the development of these seemingly different conditions. The study of BCG vaccination and of other well-documented sources of immune stimulation in the early years of life has the potential to contribute crucial knowledge on the complex influences that might have a long-term impact on chronic diseases. Further population health studies

are needed to shed more light on the role of the immune function and its modulation on health and disease. .... Given the potential public health implications, it is imperative to acquire a better understanding of the role of the modulation of the immune function in the aetiology of such chronic health conditions in the population.

REFERENCES

0514_Alinovi_PTB
Alinovi C.A., Wu C.C., Lin T.L. (2009): In utero Mycobacterium avium subspecies paratuberculosis infection of a pygmy goat. Veterinary Record, 164, 276-277.

8876_Anon_EXC
Anon. (2000): Possible links between Crohn's disease and Paratuberculosis. Report of the Scientific Committee on Animal Health and Animal Welfare Adopted 21 March 2000 (SANCO/B3/R16/2000), 1-76.

0437_Asakura_PTB
Asakura H., Suzuki K., Kitahora T., Morizane T. (2008): Is there a link between food and intestinal microbes and the occurrence of Crohn's disease and ulcerative colitis? Journal of Gastroenterology and Hepatology, 23, 1794-1801.

8830_Banwell_EXC
Banwell B., Bar-Or A., Cheung R., Kennedy J., Krupp L.B., Becker D.J., Dosch H.M. (2008): Abnormal T-cell reactivities in childhood inflammatory demyelinating disease and type 1 diabetes. Annals of Neurology, 63, 98-111.

8817_Basler_EXC
Basler T., Geffers R., Weiss S., Valentin-Weigand P., Goethe R. (2008): Mycobacterium avium subspecies induce differential expression of pro-inflammatory mediators in a murine macrophage model: Evidence for enhanced pathogenicity of Mycobacterium avium subspecies paratuberculosis. Immunobiology, 213, 879-888.

0008_Basler_EXC
Basler T., Jeckstadt S., Valentin-Weigand P., Goethe R. (2006): Mycobacterium paratuberculosis, Mycobacterium smegmatis, and lipopolysaccharide induce different transcriptional and post-transcriptional regulation of the IRG1 gene in murine macrophages. Journal of Leukocyte Biology, 79, 628-638.

0511_Begg_PTB
Begg D.J., de Silva K., Bosward K., Di Fiore L., Taylor D.L., Jungersen G., Whittington R.J. (2009): Enzyme-linked immunospot: an alternative method for the detection of interferon gamma in Johne's disease. Journal of Veterinary Diagnostic Investigation, 21, 187-196.

8845_Begue_EXC
Begue B., Dumant C., Bambou J.C., Beaulieu J.F., Chamaillard M., Hugot J.P., Goulet O., Schmitz J., Philpott D.J., Cerf-Bensussan N., Ruemmele F.M. (2006): Microbial induction of CARD15 expression in-intestinal epithelial cells via toll-like receptor 5 triggers an antibacterial response loop. Journal of Cellular Physiology, 209, 241-252.

0428_Beh_PTB
Behr M.A. (2008): Current Mycobacterium: what's on tomorrow's menu? Microbes and Infection, 10, 968-972.

8874_Bergstrand_EXC
Bergstrand O., Hellers G. (1983): Breast-Feeding During Infancy in Patients Who Later Develop Crohns-Disease. Scandinavian Journal of Gastroenterology, 18, 903-906.

8872_Bernstein_EXC
Bernstein C.N., Wajda A., Svenson L.W., MacKenzie A., Koehoorn M., Jackson M., Fedorak R., Israel D., Blanchard J.F. (2006): The epidemiology of inflammatory bowel disease in Canada: A population-based study. American Journal of Gastroenterology, 101, 1559-1568.

0271_Blink_CD_PTB
Blink S.E., Miller S.D. (2009): The Contribution of y delta T Cells to the Pathogenesis of EAE and MS. Current Molecular Medicine, 9, 15-22.

0492_Bolster_PTB
Bolster C.H., Cook K.L., Haznedaroglu B.Z., Walker S.L. (2009): The transport of Mycobacterium avium subsp paratuberculosis through saturated aquifer materials. Letters in Applied Microbiology, 48, 307-312.

0507_Branciari_PTB
Branciari R., Mammoli R., Miraglia D., Avellini P., Cioffi A., Ranucci D. (2008): Prevalence of Mycobacterium Avium Subsp. Paratuberculosis Infection in Young Beef Animals Submitted to A Regional Abattoir. Italian Journal of Food Science, 20, 471-478.

0260_Brosbol-Ravnborg_CD_PTB
Brosbol-Ravnborg A., Hvas C.L., Agnholt J., Dahlerup J.F., Vind I., Till A., Rosenstiel P., Hollsberg P. (2009): Toll-like receptor-induced granulocyte-macrophage colony-stimulating factor secretion is impaired in Crohn's disease by nucleotide oligomerization domain 2-dependent and -independent pathways. Clinical and Experimental Immunology, 155, 487-495.

0401_Bush_PTB
Bush R.D., Windsor P.A., Toribio J.A., Webster S.R. (2008): Financial modelling of the potential cost of ovine Johne's disease and the benefit of vaccinating sheep flocks in southern New South Wales. Australian Veterinary Journal, 86, 398-403.

8855_Carbone_EXC
Carbone K.M., Luftig R.B., Buckley M.R. (2005): Microbial triggers of chronic human ilness. American Academy of Microbiology Colloquium, 1-14.

0001_Cash_EXC
Cash H.L., Whitham C.V., Behrendt C.L., Hooper L.V. (25-8-2006): Symbiotic bacteria direct expression of an intestinal bactericidal lectin. Science, 313, 1126-1130.

0345_Cashman_PTB
Cashman W., Buckley J., Quigley T., Fanning S., More S., Egan J., Berry D., Grant I., O' Farrell K. (2008): Risk factors for the introduction and within-herd transmission of Mycobacterium avium subspecies paratuberculosis (MAP) infection off 59 Irish dairy herds. Irish Veterinary Journal, 61, 464-467.

0249_Chen_CD_PTB
Chen J.Z., Liu X.S. (2009): The role of interferon gamma in regulation of CD4(+) T-cells and its clinical implications. Cellular Immunology, 254, 85-90.

0533_Chia-wei_PTB
Chia-Wei W.A., Schmoller S.K., Bannantine J.P., Eckstein T.M., Inamine J.M., Livesey M., Albrecht R., Talaat A.M. (2009): A novel cell wall lipopeptide is important for biofilm formation and pathogenicity of Mycobacterium avium subspecies paratuberculosis. Microbial Pathogenesis, 46, 222-230.

8877_Chiodini_EXC
Chiodini R.J. (1989): Crohn Disease and the Mycobacterioses - A Review and Comparison of 2 Disease Entities. Clinical Microbiology Reviews, 2, 90-117.

8847_Chowers_EXC
Chowers Y., Odes S., Bujanover Y., Eliakim R., Meir S.B., Avidan B. (2004): The month of birth is linked to the risk of Crohn's disease in the Israeli population. American Journal of Gastroenterology, 99, 1974-1976.

0337_Clark_PTB
Clark D.L., Koziczkowski J.J., Radcliff R.P., Carlson R.A., Ellingson J.L.E. (2008): Detection of Mycobacterium avium subspecies paratuberculosis: Comparing fecal culture versus serum enzyme-linked immunosorbent assay and direct fecal polymerase chain reaction. Journal of Dairy Science, 91, 2620-2627.

8837_Collins_EXC
Collins C.H., Grange J.M., Yates M.D. (1984): Mycobacteria in Water. Journal of Applied Bacteriology, 57, 193-211.

0044_Collins_EXC
Collins M.T. (1997): Mycobacterium paratuberculosis: A potential food-borne pathogen? Journal of Dairy Science, 80, 3445-3448.

8861_Corrao_EXC
Corrao G., Tragnone A., Caprilli R., Trallori G., Papi C., Andreoli A., Di Paolo M., Riegler G., Rigo G.P., Ferrau O., Mansi C., Ingrosso M., Valpiani D. (1998): Risk of inflammatory bowel disease attributable to smoking, oral contraception and breastfeeding in Italy: a nationwide case-control study. International Journal of Epidemiology, 27, 397-404.

8809_Damen_EXC
Damen G.M., Hol J., de Ruiter L., Bouquet J., Sinaasappel M., van der Woude J., Laman J.D., Hop W.C.J., Buller H.A., Escher J.C., Nieuwenhuis E.E.S. (2006): Chemokine production by buccal epithelium as a distinctive feature of pediatric Crohn disease. Journal of Pediatric Gastroenterology and Nutrition, 42, 142-149.

0498_Davies_PTB
Davies G., Genini S., Bishop S.C., Giuffra E. (2009): An assessment of opportunities to dissect host genetic variation in resistance to infectious diseases in livestock. Animal, 3, 415-436.

0334_DeLuna_PTB
De Luna C.J., Sparagano O.A.E. (2007): Molecular diagnostic to identify Mycobacterial species in veterinary science. Thai Journal of Veterinary Medicine, 37, 19-24.

8843_de Mesquita_CD_PTB
de Mesquita M.B., Civitelli F., Levine A. (2008): Epidemiology, genes and inflammatory bowel diseases in childhood. Digestive and Liver Disease, 40, 3-11.

8813_DeKoter_EXC
DeKoter R.P., Walsh J.C., Singh H. (1998): PU.1 regulates both cytokine-dependent proliferation and differentiation of granulocyte/macrophage progenitors. Embo Journal, 17, 4456-4468.

0501_Delgado_PTB
Delgado F., Etchechoury D., Gioffre A., Paolicchi F., Viera F.B., Mundo S., Romano M.I. (2009): Comparison between two in situ methods for Mycobacterium avium subsp paratuberculosis detection in tissue samples from infected cattle. Veterinary Microbiology, 134, 383-387.

0204_Deretic_CD_PTB
Deretic V. (2008): Autophagy, an immunologic magic bullet: Mycobacterium tuberculosis phagosome maturation block and how to bypass it. Future Microbiology, 3, 517-524.

8853_Dieguez_PTB
Dieguez F.J., Gonzalez A.M., Menendez S., Vilar M.J., Sanjuan M.L., Yus E., Arnaiz I. (2009): Evaluation of four commercial serum ELISAs for detection of Mycobacterium avium subsp paratuberculosis infection in dairy cows. Veterinary Journal, 180, 231-235.

8831_Dow_EXC
Dow C.T. (2006): Paratuberculosis and type I diabetes is this the trigger? Medical Hypotheses, 67, 782-785.

8827_Dubinsky_EXC
Dubinsky M., Abraham B., Mahadevan U. (2008): Management of the Pregnant IBD Patient. Inflammatory Bowel Diseases, 14, 1736-1750.

0355_Eamens_PTB
Eamens G.J., Walker D.M., Porter N.S., Fell S.A. (2008): Radiometric pooled faecal culture for the detection of Mycobacterium avium subsp paratuberculosis in low-shedder cattle. Australian Veterinary Journal, 86, 259-265.

0495_East_PTB
East I.J. (2008): Addressing the problems of using the polymerase chain reaction technique as a stand-alone test for detecting pathogens in aquatic animals. Revue Scientifique et Technique-Office International des Epizooties, 27, 829-837.

8856_Economou_CD_PTB
Economou M., Pappas G. (2008): New global map of Crohn's disease: Genetic environmental and socioeconomic correlations. Inflammatory Bowel Diseases, 14, 709-720.

8858_Falkinham_EXC
Falkinham J.O. (2003): The changing pattern of nontuberculous mycobacterial disease. Can.J Infect Dis., 14, 281-286.

0489_Gao_PTB
Gao A.L., Odumeru J., Raymond M., Hendrick S., Duffield T., Mutharia L. (2009): Comparison of milk culture, direct and nested polymerase chain reaction (PCR) with fecal culture based on samples from dairy herds infected with Mycobacterium avium subsp paratuberculosis. Canadian Journal of Veterinary Research-Revue Canadienne de Recherche Veterinaire, 73, 58-64.

8862_Gearry_EXC
Gearry R.B., Richardson A., Frampton C.M.A., Collett J.A., Burt M.J., Chapman B.A., Barclay M.L. (2006): High incidence of Crohn's disease in Canterbury, New Zealand: Results of an epidemiologic study. Inflammatory Bowel Diseases, 12, 936-943.

8618_Girardin_EXC
Girardin S.E., Boneca I.G., Carneiro L.A.M., Antignac A., Jehanno M., Viala J., Tedin K., Taha M.K., Labigne A., Zahringer U., Coyle A.J., Bertin J., Sansonetti P.J., Philpott D.J. (2003): Nod1 detects a unique muropeptide from Gram-negative bacterial peptidoglycan. Science, 300, 1584-1587.

8828_Goldfarb_EXC
Goldfarb M.F. (2008): Relation of time of introduction of cow milk protein to an infant and risk of type-1 diabetes mellitus. Journal of Proteome Research, 7, 2165-2167.

/8866_Green_EXC
Green C., Elliott L., Beaudoin C., Bernstein C.N. (1-10-2006a): A population-based ecologic study of inflammatory bowel disease: Searching for etiologic clues. American Journal of Epidemiology, 164, 615-623.

/8868_Green_EXC
Green C., Elliott L., Beaudoin C., Bernstein C.N. (1-10-2006b): Green et al. respond to "Clues to the etiology of inflammatory bowel disease". American Journal of Epidemiology, 164, 627-628.

0494_Griffin_PTB
Griffin J.F.T., Hughes A.D., Liggett S., Farquhar P.A., Mackintosh C.G., Bakker D. (2009): Efficacy of novel lipid-formulated whole bacterial cell vaccines against Mycobacterium avium subsp paratuberculosis in sheep. Vaccine, 27, 911-918.

8863_Griffiths_EXC
Griffiths L.J., Tate A.R., Dezateux C. (2007): Do early infant feeding practices vary by maternal ethnic group? Public Health Nutrition, 10, 957-964.

0358_Groenendaal_PTB
Groenendaal H., Zagmutt F.J. (2008): Scenario analysis of changes in consumption of dairy products caused by a hypothetical causal link between Mycobacterium avium subspecies paratuberculosis and Crohn's disease. Journal of Dairy Science, 91, 3245-3258.

0524_Gutierrez_PTB
Gutierrez A., Frances R., Amoros A., Zapater P., Garmendia M., NDongo M., Cano R., Jover R., Such J., Perez-Mateo M. (2009): Cytokine Association with Bacterial DNA in Serum of Patients with Inflammatory Bowel Disease. Inflammatory Bowel Diseases, 15, 508-514.

0362_Haghkah_PTB
Haghkhah M., nsari-Lari M., Novin-Baheran A.M., Bahramy A. (2008): Herd-level prevalence of Mycobacterium avium subspecies paratuberculosis by bulk-tank milk PCR in Fars province (southern Iran) dairy herds. Preventive Veterinary Medicine, 86, 8-13.

8860_Hanson_EXC
Hanson L.A., Korotkova M., Haversen L., Mattsby-Baltzer I., Hahn-Zoric M., Silfverdal S.A., Strandvik B., Telemo E. (2002): Breast-feeding, a complex support system for the offspring. Pediatrics International, 44, 347-352.

0007_Hanson_EXC
Hanson L.A., Silfverdal S.A., Stromback L., Erling V., Zaman S., Olcen P., Telemo E. (2001): The immunological role of breast feeding. Pediatric Allergy and Immunology, 12, 15-19.

0406_Herthnek_PTB
Herthnek D., Nielsen S.S., Lindberg A., Bolske G. (2008): A robust method for bacterial lysis and DNA purification to be used with real-time PCR for detection of Mycobacterium avium subsp paratuberculosis in milk. Journal of Microbiological Methods, 75, 335-340.

0235_Hong_CD_PTB
Hong J.W., Leung E., Fraser A., Krissansen G.W. (2008): Nucleic Acid from Saliva and Salivary Cells for Noninvasive Genotyping of Crohn's Disease Patients. Genetic Testing, 12, 587-589.

0521_Hruska_EXC
Hruska K., Bartos M., Kralik P., Pavlik I. (2005): Mycobacterium avium subsp paratuberculosis in powdered infant milk: paratuberculosis in cattle - the public health problem to be solved. Veterinarni Medicina, 50, 327-335.

8815_Hudcovic_EXC
Hudcovic T., Stepankova R., Cebra J., Tlaskalova-Hogenova H. (2001): The role of microflora in the development of intestinal inflammation: Acute and chronic colitis induced by dextran sulfate in germ-free and conventionally reared immunocompetent and immunodeficient mice. Folia Microbiologica, 46, 565-572.

8808_Hugot_EXC
Hugot J.P., Chamaillard M., Zouali H., Lesage S., Cezard J.P., Belaiche J., Almer S., Tysk C., O'Morain C.A., Gassull M., Binder V., Finkel Y., Cortot A., Modigliani R., Laurent-Puig P., Gower-Rousseau C., Macry J., Colombel J.F., Sahbatou M., Thomas G. (31-5-2001): Association of NOD2 leucine-rich repeat variants with susceptibility to Crohn's disease. Nature, 411, 599-603.

8807_Huttunen_EXC
Huttunen K., Ruotsalainen M., Iivanainen E., Torkko P., Katila M.L., Hirvonen M.R. (2000): Inflammatory responses in RAW264.7 macrophages caused by mycobacteria isolated from moldy houses. Environmental Toxicology and Pharmacology, 8, 237-244.

8846_Joossens_EXC
Joossens M., Joossens S., Van Steen K., Pierik M., Vermeire S., Rutgeerts P., Van Ranst M. (2005): Crohn's disease and month of birth. Inflammatory Bowel Diseases, 11, 597-599.

0523_Juste_PTB
Juste R.A., Elguezabal N., Pavon A., Garrido J.M., Geijo M., Sevilla I., Cabriada J.L., Tejada A., Garcia-Campos F., Casado R., Ochotorena I., Izeta A. (2009): Association between Mycobacterium avium subsp paratuberculosis DNA in blood and cellular and humoral immune response in inflammatory bowel disease patients and controls. International Journal of Infectious Diseases, 13, 247-254.

0340_Karcher_PTB
Karcher E.L., Beitz D.C., Stabel J.R. (2008): Parturition invokes changes in peripheral blood mononuclear cell populations in Holstein dairy cows naturally infected with Mycobacterium avium subsp paratuberculosis. Veterinary Immunology and Immunopathology, 124, 50-62.

0462_Kathaperumal_PTB
Kathaperumal K., Kumanan V., McDonough S., Chen L.H., Park S.U., Moreira M.A.S., Akey B., Huntley J., Chang C.F., Chang Y.F. (2009): Evaluation of immune responses and protective efficacy in a goat model following immunization with a coctail of recombinant antigens and a polyprotein of Mycobacterium avium subsp paratuberculosis. Vaccine, 27, 123-135.

0480_Keeble_PTB
Keeble J.R., Walker K.B. (2009): Therapeutic vaccine comprising Mycobacterium HSP70. Expert Opinion on Therapeutic Patents, 19, 95-99.

0284_Khare_PTB
Khare S., Adams L.G., Osterstock J., Roussel A., David L. (2008): Effects of shipping and storage conditions of fecal samples on viability of Mycobacterium paratuberculosis. Journal of Clinical Microbiology, 46, 1561-1562.

0050_Khol_PTB
Khol J.L., Damoser J., Dunser M., Baumgartner W. (2007): Paratuberculosis, a notifiable disease in Austria - Current status, compulsory measures and first experiences. Preventive Veterinary Medicine, 82, 302-307.

8870_Klement_EXC
Klement E., Cohen R.V., Boxman J., Joseph A., Reif S. (2004): Breastfeeding and risk of inflammatory bowel disease: a systematic review with meta-analysis(1-3). American Journal of Clinical Nutrition, 80, 1342-1352.

0517_Kobayashi_EXC
Kobayashi K.S., Chamaillard M., Ogura Y., Henegariu O., Inohara N., Nunez G., Flavell R.A. (2005): Nod2-dependent regulation of innate and adaptive immunity in the intestinal tract. Science, 307, 731-734.

0020_Kohler_EXC
Kohler H., Gyra H., Zimmer K., Drager K.G., Burkert B., Lemser B., Hausleithner D., Cussler K., Klawonn W., Hess R.G. (2001): Immune reactions in cattle after immunization with a Mycobacterium paratuberculosis vaccine and implications for the diagnosis of M. paratuberculosis and M. bovis infections. Journal of Veterinary Medicine Series B-Infectious Diseases and Veterinary Public Health, 48, 185-195.

8848_Kucharzik.EXC
Kucharzik T., Maaser C., Lugering A., Kagnoff M., Mayer L., Targan S., Domschke W. (2006): Recent understanding of IBD pathogenesis: Implications for future therapies. Inflammatory Bowel Diseases, 12, 1068-1083.

8869_Kugathasan_EXC
Kugathasan S., Judd R.H., Hoffmann R.G., Heikenen J., Telega G., Khan F., Weisdorf-Schindele S., Pablo W.S., Perrault J., Park R., Yaffe M., Brown C., Rivera-Bennett M.T., Halabi I., Martinez A., Blank E., Werlin S.L., Rudolph C.D., Binion D.G. (2003): Epidemiologic and clinical characteristics of children with newly diagnosed inflammatory bowel disease in Wisconsin: A statewide population-based study. Journal of Pediatrics, 143, 525-531.

0512_Kumanan_PTB
Kumanan V., Nugen S.R., Baeumner A.J., Chang Y.F. (2009): A biosensor assay for the detection of Mycobacterium avium subsp paratuberculosis in fecal samples. Journal of Veterinary Science, 10, 35-42.

8811_Laslo_EXC
Laslo P., Pongubala J.M.R., Lancki D.W., Singh H. (2008): Gene regulatory networks directing myeloid and lymphoid cell fates within the immune system. Seminars in Immunology, 20, 228-235.

8841_Lehtola_EXC
Lehtola M.J., Torvinen E., Miettinen L.T., Keevil C.W. (2006): Fluorescence in situ hybridization using peptide nucleic acid probes for rapid detection of Mycobacterium avium subsp avium and Mycobacterium avium subsp paratuberculosis in potable-water biofilms. Applied and Environmental Microbiology, 72, 848-853.

0527_Leroy_PTB
Leroy B., Viart S., Trinchero N., Roupie V., Govaerts M., Letesson J.J., Huygen K., Wattiez R. (2009): Use of Mycobacterium avium subsp paratuberculosis specific coding sequences for serodiagnosis of bovine paratuberculosis. Veterinary Microbiology, 135, 313-319.

8881_Lipton_EXC
Lipton J.E., Kuenstner J.T., Barash D., Biesecker J. (2009): The New Paradigm for Crohn's Disease: A Call to Action. http://www.crohnscanada.org/english/researchsites/Call26.pdf,

0006_Lotz_EXC
Lotz M., Menard S., Hornef M. (2007): Innate immune recognition on the intestinal mucosa. International Journal of Medical Microbiology, 297, 379-392.

0423_Lowe_PTB
Lowe A.M., Yansouni C.P., Behr M.A. (2008): Causality and gastrointestinal infections: Koch, Hill, and Crohn's. Lancet Infectious Diseases, 8, 720-726.

0390_Lu_PTB
Lu Z., Mitchell R.M., Smith R.L., Van Kessel J.S., Chapagain P.P., Schukken Y.H., Grohn Y.T. (2008): The importance of culling in Johne's disease control. Journal of Theoretical Biology, 254, 135-146.

0516_MacDonald_EXC
MacDonald T.T., Monteleone G. (2005): Immunity, inflammation, and allergy in the gut. Science, 307, 1920-1925.

8806_Maeda_EXC
Maeda S., Hsu L.C., Liu H., Bankston L.A., Iimura M., Kagnoff M.F., Eckmann L., Karin M. (4-2-2005): Nod2 mutation in Crohn's disease potentiates NF-kappaB activity and IL-1beta processing. Science, 307, 734-738.

0274_Marina-Garcia_CD_PTB
Marina-Garcia N., Franchi L., Kim Y.G., Hu Y.J., Smith D.E., Boons G.J., Nunez G. (2009): Clathrin- and Dynamin-Dependent Endocytic Pathway Regulates Muramyl Dipeptide Internalization and NOD2 Activation. Journal of Immunology, 182, 4321-4327.

0110_Marks_CD_PTB
Marks D.J.B., Segal A.W. (2008): Innate immunity in inflammatory bowel disease: a disease hypothesis. Journal of Pathology, 214, 260-266.

0736_McCullough_PCVD
McCullough K.C., Ruggli N., Summerfield A. (2009): Dendritic cells - At the front-line of pathogen attack. Veterinary Immunology and Immunopathology, 128, 7-15.

8859_McGonagle_EXC
McGonagle D., McDermott M.F. (2006): A proposed classification of the immunological diseases. Plos Medicine, 3, 1242-1248.

8826 Mikhailov EXC
Mikhailov T.A., Furner S.E. (2009): Breastfeeding and genetic factors in the etiology of inflammatory bowel disease in children. World Journal of Gastroenterology, 15, 270-279.

0493_Mikkelsen_PTB
Mikkelsen H., Jungersen G., Nielsen S.S. (2009): Association between milk antibody and interferon-gamma responses in cattle from Mycobacterium avium subsp paratuberculosis infected herds. Veterinary Immunology and Immunopathology, 127, 235-241.

8867_Moayyedi_EXC
Moayyedi P. (1-10-2006): Invited commentary: Clues to the etiology of inflammatory bowel disease - A return to John Snow? American Journal of Epidemiology, 164, 624-626.

8854_Mobius_EXC
Mobius P., Hotzel H., Rassbach A., Kohler H. (2008): Comparison of 13 single-round and nested PCR assays targeting IS900, ISMav2, f57 and locus 255 for detection of Mycobacterium avium subsp paratuberculosis. Veterinary Microbiology, 126, 324-333.

0530_Mucha_PTB
Mucha R., Bhide M.R., Chakurkar E.B., Novak M., Mikula I. (2009): Toll-like receptors TLR1, TLR2 and TLR4 gene mutations and natural resistance to Mycobacterium avium subsp paratuberculosis infection in cattle. Veterinary Immunology and Immunopathology, 128, 381-388.

8875_Nacy_EXC
Nacy C., Buckley M. (2008): Mycobacterium avium paratuberculosis: Infrequent human pathogen or public health threat? A report from the American Academy of Microbiology, -, 1-37.

0432_Nielsen_PTB
Nielsen S.S. (2008): Transitions in diagnostic tests used for detection of Mycobacterium avium subsp paratuberculosis infections in cattle. Veterinary Microbiology, 132, 274-282.

0453_Nielsen_PTB
Nielsen S.S., Krogh M.A., Enevoldsen C. (2009): Time to the occurrence of a decline in milk production in cows with various paratuberculosis antibody profiles. Journal of Dairy Science, 92, 149-155.

0470_Nielsen_PTB
Nielsen S.S., Toft N. (2009): A review of prevalences of paratuberculosis in farmed animals in Europe. Preventive Veterinary Medicine, 88, 1-14.

8873_Odes_EXC
Odes S. (21-11-2008): How expensive is inflammatory bowel disease? A critical analysis. World Journal of Gastroenterology, 14, 6641-6647.

0405_Okafor_PTB
Okafor C., Grooms D., Alocilja E., Bolin S. (2008): Fabrication of a novel conductometric biosensor for detecting Mycobacterium avium subsp paratuberculosis antibodies. Sensors, 8, 6015-6025.

0520_Orpin_PTB
Orpin P., Sibley R. (2009): Approaches to Johne's disease. Veterinary Record, 164, 311-311.

0183_Park_CD_PTB
Park S.U., Kathaperumal K., McDonough S., Akey B., Huntley J., Bannantine J.P., Chang Y.F. (2008): Immunization with a DNA vaccine cocktail induces a Th1 response and protects mice against Mycobacterium avium subsp paratuberculosis challenge. Vaccine, 26, 4329-4337.

8836_Pedley_EXC
Pedley S., Bartram J., Rees G., Dufour A., Cotruvo J.A.E. (2004): Pathogenic Mycobacteria in Water - A Guide to Public Health Consequences, Monitoring and Management. Emerging Issues in Water and Infectious Disease Series, World Health Organization titles with IWA Publishing, 1-222.

8834_Pena_Rossi_EXC
Pena Rossi C., Hanauer S.B., Tomasevic R., Hunter J.O., Shafran I., Graffner H. (20-3-2009): Interferon beta-1a for the maintenance of remission in patients with Crohn's disease: results of a phase II dose-finding study. BMC Gastroenterol., 9, 22-

8865_Phavichitr_EXC
Phavichitr N., Cameron D.J.S., Catto-Smith A.G. (2003): Increasing incidence of Crohn's disease in Victorian children. Journal of Gastroenterology and Hepatology, 18, 329-332.

8880_Pickup_EXC.
Pickup R.W., Rhodes G., Arnott S., Sidi-Boumedine K., Bull T.J., Weightman A., Hurley M., Hermon-Taylor J. (2005): Mycobacterium avium subsp paratuberculosis in the catchment area and water of the river Taff in South Wales, United Kingdom, an its potential relationship to clustering of Crohn's disease cases in the city of Cardiff. Applied and Environmental Microbiology, 71, 2130-2139.

8882_Pickup_EXC
Pickup R.W., Rhodes G., Bull T.J., Arnott S., Sidi-Boumedine K., Hurley M., Hermon-Taylor J. (2006): Mycobacterium avium subsp paratuberculosis in lake catchments, in river water abstracted for domestic use, and in effluent from domestic sewage treatment works: Diverse opportunities for environmental cycling and human exposure. Applied and Environmental Microbiology, 72, 4067-4077.

8818_Pierce_EXC
Pierce E.S. (2009): Where Are All the Mycobacterium avium Subspecies paratuberculosis in Patients with Crohn's Disease? PLoS Pathog., 5, e1000234-

0499_Pinedo_PTB
Pinedo P.J., Buergelt C.D., Donovan G.A., Melendez P., Morel L., Wud R., Langaee T.Y., Rae D.O. (2009): Association between CARD15/NOD2 gene polymorphisms and paratuberculosis infection in cattle. Veterinary Microbiology, 134, 346-352.

0359_Pinedo_PTB
Pinedo P.J., Williams J.E., Monif G.R.G., Rae D.O., Buergelt C.D. (2008): Mycobacterium paratuberculosis shedding into milk: Association of ELISA seroreactivity with DNA detection in milk. International Journal of Applied Research in Veterinary Medicine, 6, 137-144.

0198_Qian_CD_PTB
Qian B.F., Tonkonogy S.L., Sartor R.B. (2008): Aberrant Innate Immune Responses in TLR-ligand Activated HLA-B27 Transgenic Rat Cells. Inflammatory Bowel Diseases, 14, 1358-1365.

0281_Rajaiah_CD_PTB
Rajaiah R., Moudgil K.D. (2009): Heat-shock proteins can promote as well as regulate autoimmunity. Autoimmunity Reviews, 8, 388-393.

8864_Rigas_EXC
Rigas A., Rigas B., Glassman M., Yen Y.Y., Lan S.J., Petridou E., Hsieh C.C., Trichopoulos D. (1993): Breast-Feeding and Maternal Smoking in the Etiology of Crohns-Disease and Ulcerative-Colitis in Childhood. Gastroenterology, 104, A770-A770.

8857_Rook_EXC
Rook G.A.W., Adams V., Hunt J., Palmer R., Martinelli R., Brunet L.R. (2004): Mycobacteria and other environmental organisms as immunomodulators for immunoregulatory disorders. Springer Seminars in Immunopathology, 25, 237-255.

0368_Rosseels_PTB
Rosseels V., Huygen K. (2008): Vaccination against paratuberculosis. Expert Review of Vaccines, 7, 817-832.

8878_Rousseau_EXC
Rousseau M.C., Parent M.E., St-Pierre Y. (2008): Potential health effects from non-specific stimulation of the immune function in early age: The example of BCG vaccination. Pediatric Allergy and Immunology, 19, 438-448.

0002_Ruemmele_EXC
Ruemmele F.M., El Khoury M.G., Talbotec C., Maurage C., Mougenot J.F., Schmitz J., Goulet O. (2006): Characteristics of inflammatory bowel disease with onset during the first year of life. Journal of Pediatric Gastroenterology and Nutrition, 43, 603-609.

8871_Schack-Nielsen_EXC
Schack-Nielsen L., Larnkjaer A., Michaelsen K.F. (2005): Long term effects of breastfeeding on the infant and mother. Early Nutrition and Its Later Consequences: New Opportunities, 569, 16-23.

0004_Schonenbrucher_EXC
Schonenbrucher H., Abdurnawjood A., Failing K., Bulte M. (2008): New triplex real-time PCR assay for detection of Mycobactetium avium subsp paratuberculosis in bovine feces. Applied and Environmental Microbiology, 74, 2751-2758.

0203_Scott_PTB
Scott H.M., Fosgate G.T., Libal M.C., Sneed L.W., Erol E., Angulo A.B., Jordan E.R. (2007): Field testing of an enhanced direct-fecal polymerase chain reaction procedure, bacterial culture of feces, and a serum enzyme-linked immunosorbent assay for detecting Mycobacterium avium subsp paratuberculosis infection in adult dairy cattle. American Journal of Veterinary Research, 68, 236-245.

0510_Selvaraju_PTB
Selvaraju S.B., Kapoor R., Yadav J.S. (2008): Peptide nucleic acid-fluorescence in situ hybridization (PNA-FISH) assay for specific detection of Mycobacterium immunogenum and DNA-FISH assay for analysis of pseudomonads in metalworking fluids and sputum. Molecular and Cellular Probes, 22, 273-280.

0005_Shanahan_EXC
Shanahan F. (5-1-2002): Crohn's disease. Lancet, 359, 62-69.

0214_Silk_CD_PTB
Silk J.D., Salio M., Brown J., Jones E.Y., Cerundolo V. (2008): Structural and Functional Aspects of Lipid Binding by CD1 Molecules. Annual Review of Cell and Developmental Biology, 24, 369-395.

0265_Silva_CD-PTB
Silva C.L., Bonato V.L.D., dos Santos R.R., Zarate-Blades C.R., Sartori A. (2009): Recent advances in DNA vaccines for autoimmune diseases. Expert Review of Vaccines, 8, 239-252.

0003_Singh_EXC
Singh B., Read S., Asseman C., Malmstrom V., Mottet C., Stephens L.A., Stepankova R., Tlaskalova H., Powrie F. (2001): Control of intestinal inflammation by regulatory T cells. Immunological Reviews, 182, 190-200.

0026_Skovgaard_EXC
Skovgaard N. (2007): New trends in emerging pathogens. International Journal of Food Microbiology, 120, 217-224.

0451_Slana_PTB
Slana I., Kralik P., Kralova A., Pavlik I. (2008a): On-farm spread of Mycobacterium avium subsp paratuberculosis in raw milk studied by IS900 and F57 competitive real time quantitative PCR and culture examination. International Journal of Food Microbiology, 128, 250-257.

0352_Slana_PTB
Slana I., Paolicchi F., Janstova B., Navratilova P., Pavlik I. (2008b): Detection methods for Mycobacterium avium subsp paratuberculosis in milk and milk products: a review. Veterinarni Medicina, 53, 283-306.

8849_Sommer_PTB
Sommer S., Pudrith C.B., Colvin C.J., Coussens P.M. (2009): Mycobacterium avium subspecies paratuberculosis suppresses expression of IL-12p40 and NOS genes induced by signalling through CD40 in bovine monocyte-derived macrophages. Veterinary Immunology and Immunopathology, 128, 44-52.

8842 Sonntag_ EXC
Sonntag B., Stolze B., Heinecke A., Luegering A., Heidemann J., Lebiedz P., Rijcken E., Kiesel L., Domschke W., Kucharzik T., Maaser C. (2007): Preterm birth but not mode of delivery is associated with an increased risk of developing inflammatory bowel disease later in life. Inflammatory Bowel Diseases, 13, 1385-1390.

8844_SorensenCD_PTB
Sorensen H.T., Pedersen L., Nogard L., Fonager K., Rothman K.J. (20-10-2001): Does month of birth affect risk of Crohn's disease in childhood and adolescence? British Medical Journal, 323, 907-907.

8816_Stepankova_EXC
Stepankova R., Powrie F., Kofronova O., Kozakova H., Hudcovic T., Hrncir T., Uhlig H., Read S., Rehakova Z., Benada O., Heczko P., Strus M., Bland P., Tlaskalova-Hogenova H. (2007): Segmented filamentous bacteria in a defined bacterial cocktail induce intestinal inflammation in SCID mice reconstituted with CD45RB(high) CD4+ T cells. Inflammatory Bowel Diseases, 13, 1202-1211.

0285_Stephan_PTB
Stephan R. (2007): Diagnostic system for detection of Mycobacterium avium subsp paratuberculosis.(In German). Journal fur Verbraucherschutz und Lebensmittelsicherheit-Journal of Consumer Protection and Food Safety, 2, 222-227.

8850_Stratmann_EXC
Stratmann J., Dohmann K., Heinzmann J., Gerlach G.F. (2006): Peptide aMptD-mediated capture PCR for detection of Mycobacterium avium subsp paratuberculosis in bulk milk samples. Applied and Environmental Microbiology, 72, 5150-5158.

0014_Stratmann_EXC
Stratmann J., Strommenger B., Goethe R., Dohmann K., Gerlach G.F., Stevenson K., Li L.L., Zhang Q., Kapur V., Bull T.J. (2004): A 38-kilobase pathogenicity island specific for Mycobacterium avium subsp paratuberculosis encodes cell surface proteins expressed in the host. Infection and Immunity, 72, 1265-1274.

8851_Stratmann_EXC
Stratmann J., Strommenger B., Stevenson K., Gerlach G.F. (2002): Development of a peptide-mediated capture PCR for detection of Mycobacterium avium subsp paratuberculosis in milk. Journal of Clinical Microbiology, 40, 4244-4250.

0526_Sweeney_PTB
Sweeney R.W., Whitlock R.H., Bowersock T.L., Cleary D.L., Meinert T.R., Habecker P.L., Pruitt G.W. (2009): Effect of subcutaneous administration of a killed Mycobacterium avium subsp paratuberculosis vaccine on colonization of tissues following oral exposure to the organism in calves. American Journal of Veterinary Research, 70, 493-497.

8812_Tamura_EXC
Tamura T., Tailor P., Yamaoka K., Kong H.J., Tsujimura H., O'Shea J.J., Singh H., Ozato K. (2005): IFN regulatory factor-4 and-8 govern dendritic cell subset development and their functional diversity. Journal of Immunology, 174, 2573-2581.

8879_Tang_EXC
Tang W.Y., Ho S.M. (2007): Epigenetic reprogramming and imprinting in origins of disease. Reviews in Endocrine & Metabolic Disorders, 8, 173-182.

0294_Tarver_FPD
Tarver T. (2009): BIOFILMS A Threat to Food Safety. Food Technology, 63, 46-+.

0342 Taylor PTB
Taylor D.L., Zhong L., Begg D.J., de Silva K., Whittington R.J. (2008): Toll-like receptor genes are differentially expressed at the sites of infection during the progression of Johne's disease in outbred sheep. Veterinary Immunology and Immunopathology, 124, 132-151.

0339_Tiwari_PTB
Tiwari A., VanLeeuwen J.A., Dohoo I.R., Keefe G.R., Weersink A. (2008): Estimate of the direct production losses in Canadian dairy herds with subclinical Mycobacterium avium subspecies paratuberculosis infection. Canadian Veterinary Journal-Revue Veterinaire Canadienne, 49, 569-576.

8814_Tlaskalova_EXC
Tlaskalova-Hogenova H., Stepankova R., Hudcovic T., Tuckova L., Cukrowska B., Lodinova-Zadnikova R., Kozakova H., Rossmann P., Bartova J., Sokol D., Funda D.P., Borovska D., Rehakova Z., Sinkora J., Hofman J., Drastich P., Kokesova A. (15-5-2004): Commensal bacteria (normal microflora), mucosal immunity and chronic inflammatory and autoimmune diseases. Immunology Letters, 93, 97-108.

8838_Torvinen_EXC
Torvinen E., Suomalainen S., Lehtola M.J., Miettinen I.T., Zacheus O., Paulin L., Katila M.L., Martikainen P.J. (2004): Mycobacteria in water and loose deposits of drinking water distribution systems in Finland. Applied and Environmental Microbiology, 70, 1973-1981.

8883_Traub_EXC
Traub S., von Aulock S., Hartung T., Hermann C. (2006): MDP and other muropeptides - direct and synergistic effects on the immune system. Journal of Endotoxin Research, 12, 69-85.

0022_Uhlig_EXC
Uhlig H.H., McKenzie B.S., Hue S., Thompson C., Joyce-Shaikh B., Stepankova R., Robinson N., Buonocore S., Tlaskalova-Hogenova H., Cua D.J., Powrie F. (2006): Differential activity of IL-12 and IL-23 in mucosal and systemic innate immune pathology. Immunity, 25, 309-318.

0021_Valentin-Weigand_EXC
Valentin-Weigand P., Goethe R. (1999): Pathogenesis of Mycobacterium avium subspecies paratuberculosis infections in ruminants: still more questions than answers. Microbes and Infection, 1, 1121-1127.

0157_ VanKooyk_CD_PTB.pdf
van Kooyk Y., Rabinovich G.A. (2008): Protein-glycan interactions in the control of innate and adaptive immune responses. Nature Immunology, 9, 593-601.

0497_Van_Lierop_PTB
van Lierop P.P.E., Samsom J.N., Escher J.C., Nieuwenhuis E.E.S. (2009): Role of the Innate Immune System in the Pathogenesis of Inflammatory Bowel Disease. Journal of Pediatric Gastroenterology and Nutrition, 48, 142-151.

2691_Vecerek_EXC
Vecerek V., Kozak A., Malena M., Tremlova B., Chloupek P. (2003): Veterinary meat inspection of bovine carcasses in the Czech Republic during the period of 1995-2002. Veterinarni Medicina, 48, 183-189.

0518_Vissa_PTB
Vissa V., Sakamuri R., Li W., Brennan P. (2009): Defining mycobacteria: Shared and specific genome features for different lifestyles. Indian Journal of Microbiology, 49, 11-47.

0320_Weiss_PTB
Weiss D.J., Souza C.D., Evanson O.A. (2008): Effects of nuclear factor-kappa B on regulation of cytokine expression and apoptosis in bovine monocytes exposed to Mycobacterium avium subsp paratuberculosis. American Journal of Veterinary Research, 69, 804-810.

0196_Wen_CD_PTB
Wen L., Ley R.E., Volchkov P.Y., Stranges P.B., Avanesyan L., Stonebraker A.C., Hu C.Y., Wong F.S., Szot G.L., Bluestone J.A., Gordon J.I., Chervonsky A.V. (2008): Innate immunity and intestinal microbiota in the development of Type 1 diabetes. Nature, 455, 1109-1U10.

8840_Whan_EXC
Whan L., Ball H.J., Grant I.R., Rowe M.T. (2005): Occurrence of Mycobacterium avium subsp paratuberculosis in untreated water in Northern Ireland. Applied and Environmental Microbiology, 71, 7107-7112.

0503_Whittington_PTB
Whittington R.J. (2009): Factors Affecting Isolation and Identification of Mycobacterium avium subsp paratuberculosis from Fecal and Tissue Samples in a Liquid Culture System. Journal of Clinical Microbiology, 47, 614-622.

0457_Whittington_PTB
Whittington R.J., Windsor P.A. (2009): In utero infection of cattle with Mycobacterium avium subsp paratuberculosis: A. critical review and meta-analysis. Veterinary Journal, 179, 60-69.

0023_Zur_Lage_EXC
Zur Lage S., Goethe R., Darji A., Valentin-Weigand P., Weiss S. (2003): Activation of macrophages and interference with CD4(+) T-cell stimulation by Mycobacterium avium subspecies paratuberculosis and Mycobacterium avium subspecies avium. Immunology, 108, 62-69.

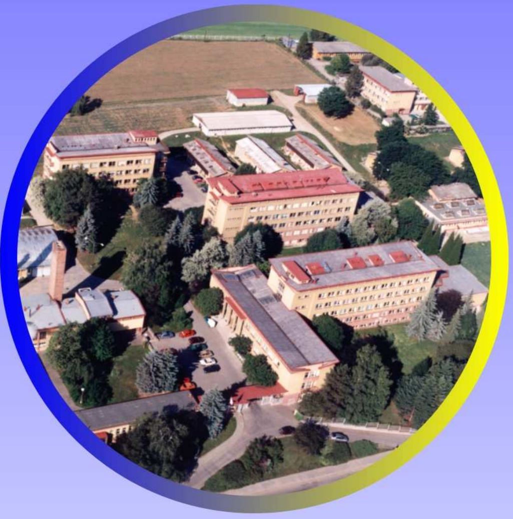

Published by
Výzkumný ústav veterinárního lékařství, v.v.i.
Veterinary Research Institute
Hudcova 70
621 00 Brno
Czech Republic
Tel. +4205 3333 2501
e-mail: centaur@vri, http://www.vri.cz
