# Supplementary material for: Long History of Queries about Bovine Paratuberculosis as a Risk Factor for Human Health
Source: Pathogens. 2021 Oct 28;10(11):1394. doi: 10.3390/pathogens10111394 (PMC8622788; doi:10.3390/pathogens10111394)
Supplement: Supplementary file 1 [file pathogens-10-01394-s001.zip › KAZDA.pdf]

Springer Link

© 2009

The Ecology of Mycobacteria: Impact on Animal's and Human's Health

- Authors
- (view affiliations)

- Jindrich Kazda
- Ivo Pavlik
- Joseph O. Falkinham III
- Karel Hruska

- All current information on the Ecology of Mycobacteria in one volume

- Presentation of exhaustive discussion of routes of infection, thus providing guidance to prevention of infection

- The physiology and genetics relating to the ecology of mycobacteria thus allowing the prediction of the presence of mycobacteria in novel habitats, particularly human engineered habitats

Book

- 40 Citations
- 7.4k Downloads

- Chapters
- About
- Reviews

Table of contents

1. Front Matter
   Pages i-xviii
   PDF
2. The Chronology of Mycobacteria and the Development of Mycobacterial Ecology
   J. Kazda
   Pages 1-11
3. Obligate Pathogenic Mycobacteria
   J. Kazda, I. Pavlik
   Pages 13-19
4. Potentially Pathogenic Mycobacteria

I. Pavlik, J.O. Falkinham III, J. Kazda
Pages 21-79
5. Physiological Ecology of Environmental Saprophytic and Potentially Pathogenic Mycobacteria
J.O. Falkinham III
Pages 81-87
6. Environments Providing Favourable Conditions for the Multiplication and Transmission of Mycobacteria
I. Pavlik, J. O. Falkinham III, J. Kazda
Pages 89-197
7. The Occurrence of Pathogenic and Potentially Pathogenic Mycobacteria in Animals and the Role of the Environment in the Spread of Infection
I. Pavlik, J.O. Falkinham III
Pages 199-281
8. Transmission of Mycobacteria from the Environment to Susceptible Hosts
I. Pavlik, J.O. Falkinham III, J. Kazda
Pages 283-312
9. Biological Role of Mycobacteria in the Environment
J. Kazda, J. O. Falkinham III
Pages 313-329
10. Key Research Issues
J. Kazda, I. Pavlik, J.O. Falkinham III, K. Hruska
Pages 331-338
11. Photographs
I. Pavlik, K. Hruska
Pages 339-516
12. Back Matter
Pages 517-520
PDF

About this book

Introduction

The Ecology of Mycobacteria principally emphasizes the ecological characteristics of the environmental mycobacteria. It is now well understood that the incidence and prevalence of potentially pathogenic mycobacteria is increasing in humans and animals. Further, proof that mycobacteria are normal inhabitants of drinking water distribution systems and household water systems, indicates that humans and animals are surrounded by mycobacteria and thus at risk. It is anticipated that the emphasis on ecology and routes of infection will result in a text of widespread use for clinicians and for research scientists in medicine, academia, and industry. In addition to identifying habitats and thereby sources of mycobacteria infecting humans and animals, the text identifies those mycobacterial characteristics that determine its range of habitats. Additionally, the text comments critically on the available methods to identify those protocols with values in mycobacterial research. In that manner, although there are no chapters specifically devoted to methods, superior methods for mycobacteria will be identified.

A new text is needed for the mycobacteria because the prevalence of disease caused by the environmental potentially pathogenic mycobacteria is increasing. This increase is due to a number of factors. Host factors contribute to an increasing population of individuals more susceptible to mycobacterial infection. For example, the aging of the human population and the

increasing frequency of immunosuppressed individuals as a result of infection (e.g. HIV), chemotherapy, and transplant-associated immunosuppression are all factors leading to increased susceptibility of infection with environment derived mycobacteria. Moreover, the role of mycobacteria as triggers in different autoimmune diseases is more and more evident. It is highly probable that peptidoglycans, lipoglycans, lipoproteins, heat shock proteins and some other structures from the mycobacterial cell wall, participate in different pathways of non-specific inflammatory reactions in humans, namely those with a specific genetic disposition. In such events mycobacteria in drinking water and food, even devitalized, have to be considered as a public health risk.

Second, human-engineered systems such as drinking water distribution systems are creating a habitat for the selection and proliferation of the potentially pathogenic mycobacteria. In as much as drinking water brings together overlapping habitats of both mycobacteria and humans and animals, a review of mycobacterial ecology is timely. The ecology of mycobacteria helps to understand the circulation of mycobacteria into the respective disciplines such as epidemiology, epizootology, immunology, environmental ecology, animal husbandry and environment conservation.

Keywords

Epidemiology HIV autoimmune disease bacteria bacterial cell bacterial infection infection infectious diseases

Authors and affiliations

- Jindrich Kazda (1)
- Ivo Pavlik (2)
- Joseph O. Falkinham III (3)
- Karel Hruska (4)

1. , Borstel, Germany
2. Veterinary Research Institute, , Brno, Czech Republic
3. Dept. of Biological Sciences (MC 406), Virginia Tech, , Blacksburg, U.S.A.
4. Veterinary Research Institute, , Brno, Czech Republic

Bibliographic information

- Book Title The Ecology of Mycobacteria: Impact on Animal's and Human's Health
- Authors Jindrich Kazda
  Ivo Pavlik
  Joseph O. Falkinham III
  Karel Hruska
- DOI https://doi.org/10.1007/978-1-4020-9413-2
- Copyright Information Springer Science+Business Media B.V. 2009
- Publisher Name Springer, Dordrecht
- eBook Packages Biomedical and Life Sciences Biomedical and Life Sciences (R0)
- Hardcover ISBN 978-1-4020-9412-5
- Softcover ISBN 978-94-017-7710-0
- eBook ISBN 978-1-4020-9413-2
- Edition Number 1
- Number of Pages XVIII, 522
- Number of Illustrations 0 b/w illustrations, 0 illustrations in colour

- Topics Bacteriology
  Infectious Diseases
  Epidemiology
  Microbial Ecology
  Microbiology
  Veterinary Medicine/Veterinary Science
- Buy this book on publisher's site

Reviews

From the reviews: “If you are by any means interested in the microbial ecology of mycobacteria … this book is your choice. … The pictures are of high technical standard and imminently supply the body of the text by references. … addresses a wide range of scientists and others who are engaged in epidemiology, epizootology, immunology, environmental ecology, and animal husbandry. It presents the state of the art of the ecology of mycobacteria, a virtual gold mine of the subject. Do not miss it.” (Niels Skovgaard, International Journal of Food Microbiology, Vol. 136, 2010) “The primary focus is the ecology of mycobacteria species in human and animal diseases, including mycobacteria interactions and roles in the environment. … The editors have included examples, graphics, bibliographies, and references to the illustrations … to explain the concepts and ideas discussed. The information included in this work is recent and relevant and will be useful to readers wishing to gain an understanding of general mycobacterial ecology. Summing Up: Recommended. Lower-division undergraduates through graduate students, general audiences.” (P. M. Watt, Choice, Vol. 47 (7), March, 2010)

SPRINGER NATURE

© 2020 Springer Nature Switzerland AG. Part of Springer Nature.

Not logged in Not affiliated 95.82.188.174
