# Supplementary material for: Long History of Queries about Bovine Paratuberculosis as a Risk Factor for Human Health
Source: Pathogens. 2021 Oct 28;10(11):1394. doi: 10.3390/pathogens10111394 (PMC8622788; doi:10.3390/pathogens10111394)
Supplement: Supplementary file 1 [file pathogens-10-01394-s001.zip › KUENSTNER.pdf]

frontiers in Public Health

PERSPECTIVE
published: 27 September 2017
doi: 10.3389/fpubh.2017.00208

Check for updates

The Consensus from the *Mycobacterium avium ssp. paratuberculosis* (MAP) Conference 2017

J. Todd Kuenstner*, Saleh Naser, William Chamberlin, Thomas Borody, David Y. Graham, Adrienne McNees, John Hermon-Taylor, Amy Hermon-Taylor, C. Thomas Dow, Walter Thayer, James Biesecker, Michael T. Collins, Leonardo A. Sechi, Shoor Vir Singh, Peilin Zhang, Ira Shafran, Stuart Weg, Grzegorz Telega, Robert Rothstein, Harry Oken, Stephen Schimpff, Horacio Bach, Tim Bull, Irene Grant, Jay Ellingson, Heinrich Dahmen, Judith Lipton, Saurabh Gupta, Kundan Chaubey, Manju Singh, Prabhat Agarwal, Ashok Kumar, Jyoti Misri, Jagdip Sohal, Kuldeep Dhama, Zahra Hemati, William Davis, Michael Hier, John Aitken, Ellen Pierce, Nicole Parrish, Neil Goldberg, Maher Kali, Sachin Bendre, Gaurav Agrawal, Robert Baldassano, Preston Linn, Raymond W. Sweeney, Marie Fecteau, Casey Hofstaedter, Raghava Potula, Olga Timofeeva, Steven Geier, Kuruvilla John, Najah Zayanni, Hoda M. Malaty, Christopher Kahlenborn, Amanda Kravitz, Adriano Bulfon, George Daskalopoulos, Hazel Mitchell, Brett Neilan, Verlaine Timms, Davide Cossu, Giuseppe Mameli, Paul Angermeier, Tomislav Jelic, Ralph Goethe, Ramon A. Juste and Lauren Kuenstner

Temple University Health System, Philadelphia, PA, United States

OPEN ACCESS

Edited by:
Olivier Vandenberg, Free University of Brussels, Belgium

Reviewed by:
Walter M. R. Oelemann, Federal University of Rio de Janeiro, Brazil
Jose Roberto Lapa E. Silva, Federal University of Rio de Janeiro, Brazil

****Correspondence:***
J. Todd Kuenstner
John.Kuenstner@tuhs.temple.edu

Specialty section:
This article was submitted to Infectious Diseases – Surveillance, Prevention and Treatment, a section of the journal Frontiers in Public Health

Received: *29 May 2017*
Accepted: *28 July 2017*
Published: *27 September 2017*

Citation:
Kuenstner JT, Naser S, Chamberlin W, Borody T, Graham DY, McNees A, Hermon-Taylor J, Hermon-Taylor A, Dow CT, Thayer W, Biesecker J, Collins MT, Sechi LA, Singh SV, Zhang P, Shafran I, Weg S, Telega G, Rothstein R, Oken H, Schimpff S, Bach H, Bull T, Grant I, Ellingson J, Dahmen H, Lipton J, Gupta S, Chaubey K, Singh M, Agarwal P, Kumar A, Misri J, Sohal J, Dhama K, Hemati Z, Davis W, Hier M, Aitken J, Pierce E, Parrish N, Goldberg N, Kali M, Bendre S, Agrawal G, Baldassano R, Linn P, Sweeney RW, Fecteau M, Hofstaedter C, Potula R, Timofeeva O, Geier S, John K, Zayanni N, Malaty HM, Kahlenborn C, Kravitz A, Bulfon A, Daskalopoulos G, Mitchell H, Neilan B, Timms V, Cossu D, Mameli G, Angermeier P, Jelic T, Goethe R, Juste RA and Kuenstner L (2017) The Consensus from the Mycobacterium avium ssp. paratuberculosis (MAP) Conference 2017. Front. Public Health 5:208. doi: 10.3389/fpubh.2017.00208

On March 24 and 25, 2017 researchers and clinicians from around the world met at Temple University in Philadelphia to discuss the current knowledge of *Mycobacterium avium* ssp. *paratuberculosis* (MAP) and its relationship to human disease. The conference was held because of shared concern that MAP is a zoonotic bacterium that poses a threat not only to animal health but also human health. In order to further study this problem, the conferees discussed ways to improve MAP diagnostic tests and discussed potential future anti-MAP clinical trials. The conference proceedings may be viewed on the www.Humanpara.org website. A summary of the salient work in this field is followed by recommendations from a majority of the conferees.

Keywords: *Mycobacterium avium* subspecies *paratuberculosis*, Crohn's disease, zoonosis and health public, Johne's disease, type I diabetes, complex regional pain syndrome, lymphangiomatosis

REVIEW OF THE LITERATURE AND CONCLUSIONS OF THE CONFEREES

Clinical and pathological similarities between the chronic regional inflammatory disease in humans and the disease in ruminants later called Johne's disease (JD) were pointed out shortly after the cattle disease was described in 1895 (1). Its *Mycobacterium avium* spp. *paratuberculosis* (MAP) etiology was identified by Twort et al. in culture (2) and human pathological features described by Dalziel (3). The recovery of MAP following prolonged culture of ileal tissue from some patients with Crohn's Disease (CD) (4), its identification by nucleic acid hybridization as MAP in the intestinal tissue of some patients with CD (5) and ability to culture viable MAP from peripheral blood monocytes and breast milk from CD patients all support the theory that MAP is a cause of CD (6, 7). In a blind study, three independent laboratories using Naser's method have confirmed that viable MAP is found at a

higher prevalence in CD patients than in controls (8). This theory remains plausible and valid (9, 10).

Explanations for a cause of CD must account for a number of established observations: (a) two meta-analyses of the large number of studies have confirmed that MAP can be detected at a higher prevalence in patients with CD than in healthy controls (11, 12), (b) MAP has been cultured from peripheral blood mononuclear cells significantly more often in patients with CD than normal controls (6), and (c) open label (13, 14) and controlled clinical trials of antibiotic therapy of CD have shown a therapeutic benefit with ciprofloxacin, metronidazole, clarithromycin, rifamycin analogs, and clofazimine (15). In a recent confirmation of the MAP association with CD by another research group, Timms et al. used 3 PCR assays (IS900, f57, and nested IS900) and age- and sex-matched controls and also found a significant association of MAP and CD ($p = 0.02$) (16). Detection of MAP in non-CD patients is alarming since MAP has also been linked to several other chronic inflammatory syndromes (17).

The hypothesis that MAP is a cause of CD was dealt a blow when an Australian Trial (the Selby study) of anti-MAP antibiotics in CD patients suggested no therapeutic benefit (18). However, an "intention-to-treat" re-analysis showed that the addition of combination antibiotics to standard of care therapy and steroids used in the trial performed significantly better than placebo plus standard of care therapy and steroids (19). The current interpretation of the Australian Trial is that the antimycobacterial therapy was beneficial in treating CD but was not curative. It is important to note that the doses used in the Australian trial were lower than the doses used in current clinical trials (RedHill Biopharma first FDA phase III trial NCT01951326).

Anecdotal case reports support a causal role for MAP in CD with antibiotic therapy producing long-term profound remission off any active therapy, which resembles cured CD. For example, in 2002, Hermon-Taylor described a patient with classic CD and a positive MAP ELISA test [one of the tests used to identify MAP infection in cattle (20)] The patient received antimycobacterial therapy (clarithromycin, rifabutin, and clofazimine) for 1.5 years and has remained without disease since 2001 (Hermon-Taylor J, Personal Communication, 2017). Additional cases of patients that were prospectively treated for MAP infection have also shown long-term profound remission/cures (21, 22) including one patient with a positive MAP blood culture that became negative along with resolution of lesions associated with CD (23). Similar reports have been published including resolution of disease in two patients, one with classic CD and the other with complex regional pain syndrome, following treatment for blood cultures positive for MAP with resolution of disease and blood cultures for MAP becoming negative (24). Eleven further CD patients with profound, clinical, and histological remission for 3–22 years off all therapy were presented at the conference (25). Such reports indicate that anti-MAP therapies are bringing us closer to approaching a cure (14). Singh et al. (26) described an Indian CD patient with stool culture positive for typical MAP colonies on HEY agar slants containing mycobactin J. Biotyping of the colonies with the IS*1311* PCR-RE and IS*1311* L2 PCR-REA demonstrated the bacterium was the "Indian Bison type," of MAP, the predominant form of MAP in India (27). The patient's serum was also positive for MAP antibodies by ELISA. Following 1 year of antibiotic therapy, the disease was suppressed and the organism could no longer be cultured from the stool.

Mycobacterium avium ssp. *paratuberculosis*, the organism proven to cause JD in ruminant animals and identical to the organism isolated in the previously described patients, has been cultured from pasteurized milk (28–30), from beef (31), and from commercial infant formula (32). By USDA estimates, the herd-level prevalence of MAP infection in U.S. dairy herds has increased from 21.6% in 1996 to 91.1% in 2007 (33). The magnitude of the difference in prevalence over this period of time suggests that the change is unlikely due to sampling methods alone. During this same period, CD has increased in the US and now affects 201 per 100,000 adults (34). Hospital stays for any-listed CD increased from >120,000 (44.2 per 100,000) in 2003 to >196,000 (59.7 per 100,000) in 2013 ($p < 0.05$) (35).

Using a standard definition of consensus, which is the majority opinion, the conference participants reached consensus on several issues relating to MAP. A majority of the conferees (78%) at the Temple University MAP conference concluded that the accumulating information now strongly supports the theory that MAP is a zoonotic bacterium while 22% were uncertain whether MAP causes human disease. A majority of the conferees (72%) noted that MAP present in dairy products and meat causes disease in some humans and thus poses a public health threat while 28% were uncertain whether MAP is a public health risk. In addition, to better understand the role of this organism in the human host, the conferees proposed the following future efforts.

To address concerns about the reproducibility and sensitivity of the different diagnostic methods currently used, the group agreed to undertake a cross-laboratory comparison of four different already available blood culture methods (6, 36–38). The MAP blood culture test that performs best will then be compared to the intestinal tissue culture method (37). MAP isolates will undergo genetic sequencing and be compared to published MAP genomic data (39) to confirm identity.

A cross-laboratory comparison of the available diagnostic serologic MAP antibody tests will also be undertaken (40–43). The preceding studies will be performed with blinded samples from CD patients and from healthy controls obtained from multiple clinical sites.

The blood culture method could then be used to select patients for a study of a combination of antibiotics in CD patients. Suggested inclusion criteria for a clinical trial include an established CD diagnosis and two positive blood cultures separated by at least 1 week. Additional cultures would be done during and after the study to test the association between the clinical response and MAP detection.

The antibiotic therapy that would be prescribed in the treatment group remains to be finalized. As previously described, RedHill Biopharma is already conducting a US first FDA phase III clinical trial of a combination of clarithromycin, rifabutin, and clofazimine in CD patients. Instead of repeating the work of this groundbreaking study, other options should be considered for future clinical trials. Such possible options include: combinations of clarithromycin, ethambutol, methotrexate, Vitamin D,

metronidazole, rifaximin, rifampicin, gentamicin, or amikacin (22, 44–49). Each combination requires preliminary "proof of concept" studies and the study authors will agree on the best choice at a later date, taking into account previous experience which has been reported in the literature.

Based on the results of the genome wide association studies linking CD with innate immune deficiency, other therapies that enhance immunity should be considered. It also was noted that gallium nitrate and monensin sodium have been proposed for use to treat JD and should be considered for possible human use (50).

A majority of the conferees concluded that current evidence strongly supports the theory that MAP causes CD in some genetically susceptible human hosts. While it is impossible and inappropriate to feed MAP to healthy human infants to test whether genetically predisposed individuals develop CD, such exposure to viable MAP in infant formula and milk occurs daily. The best proof of causation is still Koch's and later Relman's postulates, which have now been satisfied for MAP in CD (51, 52). Ideally, reliably curing the disease with anti-MAP therapy, and linking profound remission/cure to the posttreatment absence of the MAP organism in the human host would be very supportive. This latter step has been accomplished in the small number of patients as described in the preceding references. Controlled clinical trials will only prove or disprove that a particular therapy is better than the control placebo or an alternate therapy. Nevertheless, even today, the precautionary principle (53) should apply for the protection of the public health.

A majority of the conferees request that the appropriate governmental agencies (NIH, FDA, USDA and the CDC, and their counterparts in other nations) review all available literature on JD (in cattle) and CD (in humans) that supports that these two diseases have a shared etiology (MAP) and consider epidemiologic studies (including available data from other countries including Italy, India, China, Israel, Japan, and Bahrain) (54) to explore this association further, using the Mini Sentinel and/or other databases.

A majority of the conferees strongly urge that the possibility that MAP causes human disease no longer be ignored. Should further compelling evidence become available, it is recommended that the FDA and USDA (and their counterparts in other nations) have contingency plans in place to rapidly eliminate MAP from the milk and meat supply through effective MAP control measures including biosecurity and hygiene, vaccination, and test-and-cull programs (20). Even if public health measures are not put in place by the appropriate regulatory agencies, food producers are encouraged to offer food products from animals in MAP control programs. Many food producers are already undertaking voluntary control practices (55) and this effort is encouraged and commended. The international representation in the authorship of this article attests to the observation that CD is now a worldwide epidemic.

AUTHOR CONTRIBUTIONS

All of the coauthors contributed to the consensus opinions of this conference and to the writing of this manuscript.

REFERENCES

1. Johne HA, Frothingham L. Ein eigenthümlicher Fall von Tuberculose beim Rind. *Deutsche Zeitschrift für Thiermedizin und vergleichende Pathologie* (1895) 21:438–55.
2. Twort FW, Ingram GL, Ingram Y. A method for isolating and cultivating *Mycobacterium* enteritidis chronicae pseudotuberculosae bovis johne and some experiments on the preparation of a diagnostic vaccine for pseudotuberculosae enteritis of bovines. *Proc Royal Soc Lond* (1912) 84:517–43. doi:10.1098/rspb.1912.0011
3. Dalziel TK. Chronic interstitial enteritis. *Br Med J* (1913) 2:1068–70.
4. Chiodini RJ, Van Kruiningen HJ, Merkal RS, Thayer WR Jr, Coutu JA. Characteristics of an unclassified *Mycobacterium* species isolated from patients with Crohn's disease. *J Clin Microbiol* (1984) 20(5):966–71.
5. Yoshimura HH, Graham DY, Estes MK, Merkal RS. Investigation of association of mycobacteria with inflammatory bowel disease by nucleic acid hybridization. *J Clin Microbiol* (1987) 25:45–51.
6. Naser SA, Ghobrial G, Romero C, Valentine JF. Culture of *Mycobacterium avium* subspecies *paratuberculosis* from the blood of patients with Crohn's disease. *Lancet* (2004) 364:1039–44. doi:10.1016/S0140-6736(04)17058-X
7. Naser SA, Schwartz D, Shafran I. Isolation of *Mycobacterium avium* subsp. *paratuberculosis* from breast milk of Crohn's disease patients Letter to the Editor. *Am J Gastroenterol* (2000) 95:1094–5. doi:10.1111/j.1572-0241.2000.01954.x
8. Naser S, Collins M, Crawford J, Valentine J. Culture of *Mycobacterium avium* subspecies *paratuberculosis* (MAP) from the blood of patients with Crohn's disease: a follow-up blind multicenter investigation. *Open Inf J* (2009) 2:22–3. doi:10.2174/1875041900902010022
9. Hermon-Taylor J, Bull TJ, Sheridan JM, Sumar N. Causation of Crohn's disease by *Mycobacterium avium* subspecies *paratuberculosis*. *Can J Gastroenterol* (2000) 14(6):521–39. doi:10.1155/2000/798305
10. Behr M, Kapur V. The evidence for *Mycobacterium paratuberculosis* in Crohn's disease. *Curr Opin Gastroenterol* (2008) 24(1):17–21. doi:10.1097/MOG.0b013e3282f1dcc4
11. Feller M, Huwiler K, Stephan R, Altpeter E, Shang A, Furrer H, et al. *Mycobacterium avium* subspecies *paratuberculosis* and Crohn's disease: a systematic review and meta-analysis. *Lancet Infect Dis* (2007) 7:607–13. doi:10.1016/S1473-3099(07)70211-6
12. Abubakar I, Myhill D, Aliyu SH, Hunter PR. Detection of *Mycobacterium avium* subspecies *paratuberculosis* from patients with Crohn's disease using nucleic acid-based techniques: a systematic review and meta-analysis. *Inflamm Bowel Dis* (2008) 14:401–10. doi:10.1002/ibd.20276
13. Shafran I, Kugler L, El-Zaatari FAK, Naser SA, Sandoval J. Open clinical trial of rifabutin and clarithromycin therapy in Crohn's disease. *Digest Liver Dis* (2002) 34:22–8. doi:10.1016/S1590-8658(02)80055-X
14. Borody TJ, Leis S, Warren EF, Surace R. Treatment of severe Crohn's disease using antimycobacterial triple therapy-approaching a cure? *Digest Liver Dis* (2002) 34(1):29–38. doi:10.1016/S1590-8658(02)80056-1
15. Feller M, Huwiler K, Schoepfer A, Shang A, Furrer H, Egger M. Long-term antibiotic treatment for Crohn's disease: systematic review and meta-analysis of placebo-controlled trials. *Clin Infect Dis* (2010) 50:473–80. doi:10.1086/649923
16. Timms VJ, Daskalopoulos G, Mitchell HM, Neilan BA. The association of *Mycobacterium avium* subsp. *paratuberculosis* with inflammatory bowel disease. *PLoS One* (2016) 11(2):e0148731. doi:10.1371/journal.pone.0148731
17. Sechi LA, Dow CT. *Mycobacterium avium* ss. *paratuberculosis* zoonosis – The Hundred Year War – beyond Crohn's disease. *Front Immunol* (2015) 6:96. doi:10.3389/fimmu.2015.00096
18. Selby W, Pavli P, Crotty B, Florin T, Radford-Smith G, Gibson P, et al. Two-year combination antibiotic therapy with clarithromycin, rifabutin, and clofazimine for Crohn's disease. *Gastroenterology* (2007) 132:2313–9. doi:10.1053/j.gastro.2007.03.031
19. Behr MA, Hanley J. Antimycobacterial therapy for Crohn's disease: a reanalysis. *Lancet Infect Dis* (2008) 8(6):344. doi:10.1016/S1473-3099(08)70104-X
20. Sweeney RW, Collins MT, Koets AP, McGuirk SM, Roussel AJ. ACVIM consensus statement: paratuberculosis (Johne's disease) in cattle and other susceptible species. *J Vet Int Med* (2012) 26:1239–50. doi:10.1111/j.1939-1676.2012.01019.x

21. Lipton JE. My personal MAP-quest. *The Paratuberculosis Newsletter*. International Association for Paratuberculosis (2012). p. 17–8. Available from: www.paratuberculosis.info/web/images/newsletters/2012q2.pdf
22. Goldberg ND, Vadlamudi A, Parrish N. Treatment of refractory Crohn's disease and Pyoderma Gangrenosum with a combination regimen of rifaximin, gentamicin and metronidazole. *Case Rep Gastroenterol* (2015) 9:25–8. doi:10.1159/000369965
23. Chamberlin W, Borody TJ, Campbell J. Primary treatment of Crohn's disease: combined antibiotics taking center stage. *Expert Rev Clin Immunol* (2011) 7(6):751–60. doi:10.1586/eci.11.43
24. Kuenstner JT, Chamberlin W, Naser SA, Collins MT, Dow CT, Aitken JM, et al. Resolution of Crohn's disease and complex regional pain syndrome following treatment of paratuberculosis. *World J Gastroenterol* (2015) 21:4048–62. doi:10.3748/wjg.v21.i13.4048
25. Agrawal G, Jayewardene AF, Leis S, Gadalla S, De Zoysa P, Huynh R, Borody TJ. Prolonged endoscopic remission with mucosal healing in crohn's patients-treatment cessation for 3-23 years (2017). Poster Presentation (P2169). Orlando, FL. USA: World Congress of Gastroenterology.
26. Singh SV, Kuenstner JT, Davis WC, Agarwal P, Kumar N, Gupta S, et al. Concurrent resolution of chronic diarrhea likely due to Crohn's disease and infection with *Mycobacterium avium paratuberculosis*. *Front Med* (2016) 3:49. doi:10.3389/fmed.2016.00049
27. Singh SV, Sohal JS, Singh PK, Singh AV. Genotype profiles of *Mycobacterium avium* subspecies *paratuberculosis* isolates recovered from animals, commercial milk, and human beings in North India. *Int J Infect Dis* (2009) 13:e221–7. doi:10.1016/j.ijid.2008.11.022
28. Millar D, Ford J, Sanderson J, Withey S, Tizard M, Doran T, et al. IS*900* PCR to detect *Mycobacterium paratuberculosis* in retail supplies of whole pasteurized cows' milk in England and Wales. *Appl Environ Microbiol* (1996) 62(9):3446–52.
29. Grant IR, Ball HJ, Rowe MT. Incidence of *Mycobacterium paratuberculosis* in bulk raw and commercially pasteurized cows' milk from approved dairy processing establishments in the United Kingdom. *Appl Environ Microbiol* (2002) 68:2428–35. doi:10.1128/AEM.68.5.2428-2435.2002
30. Ellingson JL, Anderson JL, Koziczkowski JJ, Radcliff RP, Sloan SJ, Allen SE, et al. Detection of viable *Mycobacterium avium* subsp. *paratuberculosis* in retail pasteurized whole milk by two culture methods and PCR. *J Food Prot* (2005) 68:966–72. doi:10.4315/0362-028X-68.5.966
31. Savi R, Ricchi M, Cammi G, Garbarino S, Leo S, Pongolini S, et al. Survey on the presence of *Mycobacterium avium* subsp. *paratuberculosis* in ground beef from an industrial meat plant. *Vet Microbiol* (2015) 177(3–4):403–8. doi:10.1016/j.vetmic.2015.03.013
32. Botsaris G, Swift BMC, Slana I, Liapi M, Christodoulou M, Hatzitofi M, et al. Detection of viable *Mycobacterium avium* subspecies *paratuberculosis* in powdered infant formula by phage-PCR and confirmed by culture. *Int J Food Microbiol* (2016) 216:91–4. doi:10.1016/j.ijfoodmicro.2015.09.011
33. Lombard JE, Gardner IA, Jafarzadeh SR, Fossler CP, Harris B, Capsel BR, et al. Herd-level prevalence of *Mycobacterium avium* subsp. *paratuberculosis* infection in United States dairy herds in 2007. *Prev Vet Med* (2013) 108(2–3):234–8. doi:10.1016/j.prevetmed.2012.08.006
34. Centers for Disease Control and Prevention. *Inflammatory Bowel Disease*. (2017). Available from: https://www.cdc.gov/ibd/ibd-epidemiology.htm
35. Centers for Disease Control and Prevention. Hospitalizations for Crohn's disease – United States, 2003-2013. *Morb Mortal Wkly Rep* (2017) 66(14): 377–81. doi:10.15585/mmwr.mm6614a1
36. Gearry RB, Aitken JM, Roberts RL, Ismail S, Keenan J, Barclay ML. Gastrointestinal: *Mycobacterium avium paratuberculosis* and Crohn's Disease. *J Gastroenterol Hepatol* (2005) 20:1943. doi:10.1111/j.1440-1746.2005.04187.x
37. Bull TJ, Munshi T, Mikkelsen H, Hartman SB, Sorensen MR, Garcia JS, et al. Improved culture medium (TiKa) for *Mycobacterium avium* subspecies *paratuberculosis* (MAP) matches qPCR sensitivity and reveals significant proportions of non-viable MAP in lymphoid tissue of vaccinated MAP challenged animals. *Front Microbiol* (2017) 7:2112. doi:10.3389/fmicb.2016.02112
38. Swift BMC, Huxley JN, Plain KM, Begg DJ, de Silva K, Purdie AC, et al. Evaluation of the limitations and methods to improve rapid phage-based detection of viable *Mycobacterium avium* subsp. *paratuberculosis* in the blood of experimentally infected cattle. *BMC Vet Res* (2016) 12:115. doi:10.1186/s12917-016-0728-2
39. Lingling L, Bannantine JP, Zhang Q, Amonsin A, May BJ, Alt D, et al. The complete genome sequence of *Mycobacterium avium* subspecies *paratuberculosis*. *Proc Natl Acad Sci U S A* (2005) 102:12344–9. doi:10.1073/pnas.0505662102
40. Bach H, Ko HH, Raizman EA, Attarian R, Cho B, Biet F, et al. Immunogenicity of *Mycobacterium avium* subsp. *paratuberculosis* proteins in Crohn's disease patients. *Scand J Gastroenterol* (2011) 46:30–9. doi:10.3109/00365521.2010.513061
41. Shin AR, Kim HJ, Cho SN, Collins MT, Manning EJ, Naser SA, et al. Identification of seroreactive proteins in the culture filtrate antigen of *Mycobacterium avium* ssp. *paratuberculosis* human isolates to sera from Crohn's disease patients. *FEMS Immunol Med Microbiol* (2010) 58:128–37. doi:10.1111/j.1574-695X.2009.00617.x
42. Di Sabatino A, Paccagnini D, Vidali F, Rosu V, Biancheri P, Cossu A, et al. Detection of *Mycobacterium avium* subsp. *paratuberculosis* (MAP)-specific IS*900* DNA and antibodies against MAP peptides and lysate in the blood of Crohn's disease patients. *Inflamm Bowel Dis* (2011) 17(5):1254–5. doi:10.1002/ibd.21461
43. Zhang P, Minardi LM, Kuenstner JT, Zekan SM, Zhu F, Hu Y, et al. *Cross Reactivity of Antibodies against Microbial Proteins to Human Tissues as Basis of Crohns Disease and Other Autoimmune Diseases*. Cold Spring Harbor, NY: Cold Spring Harbor Laboratory bioRxiv beta. doi:10.1101/116574
44. Miwa S, Shirai M, Toyoshima M, Shirai T, Yasuda K, Yokomura K, et al. Efficacy of clarithromycin and ethambutol for *Mycobacterium avium* Complex pulmonary disease. A preliminary study. *Ann Am Thorac Soc* (2013) 11:23–9. doi:10.1513/AnnalsATS.201308-266OC
45. Greenstein RJ, Su L, Haroutunian V, Shahidi A, Brown ST. On the action of methotrexate and 6-mercaptopurine on *M. avium* subspecies *paratuberculosis*. *PLoS One* (2007) 2:e161. doi:10.1371/journal.pone.0000161
46. Greenstein RJ, Su L, Brown ST. Vitamins A & D inhibit the growth of mycobacteria in radiometric culture. *PLoS One* (2012) 7:e29631. doi:10.1371/journal.pone.0029631
47. McDonald JWD, Wang Y, Tsoulis DJ, MacDonald JK, Feagan BG. *Methotrexate for Induction of Remission in Refractory Crohn's Disease*. Hoboken, NJ: John Wiley & Sons, Ltd (2014).
48. Prantera C, Lochs H, Grimaldi M, Danese S, Scribano ML, Gionchetti P, et al. Rifaximin-extended intestinal release induces remission in patients with moderately active Crohn's disease. *Gastroenterology* (2012) 142:473–81. doi:10.1053/j.gastro.2011.11.032
49. Griffith DE, Aksamit T, Brown-Elliott BA, Catanzaro A, Daley C, Gordin F, et al. An official ATS/IDSA statement: diagnosis, treatment, and prevention of nontuberculous mycobacterial diseases. *Am J Respir Crit Care Med* (2007) 175:367–416. doi:10.1164/rccm.200604-571ST
50. Fecteau M. *Antimicrobial Activity of Gallium Nitrate and Monensin Sodium against Mycobacterim avium subsp. Paratuberculosis (MAP) In Vitro*. Philadelphia, PA: National Institute of Food and Agriculture. Project number PENV2008-5033 (2008).
51. Chamberlin W, Borody T, Naser S. MAP-associated Crohn's disease – MAP, Koch's postulates, causality and Crohn's disease. *Digest Liver Dis* (2007) 39:790–4. doi:10.1016/j.did.2007.05.012
52. Fredricks D, Relman D. Sequence-based identification of microbial pathogens: a reconsideration of Koch's postulates. *Clin Microbiol Rev* (1996) 9:18–33.
53. Weir E, Schabas R, Wilson K, Mackie C. A Canadian framework for applying the precautionary principle to public health Issues. *Can J Public Health* (2010) 101(5):396–8. doi:10.17269/cjph.101.2635
54. Zayyani NR, Malaty HM, Graham DY. Increasing incidence of Crohn's disease with familial clustering in the Kingdom of Bahrain: a 25-year population-based study. *Inflamm Bowel Dis* (2017) 23:304–9. doi:10.1097/MIB.0000000000001016
55. McCullough D. *Dairy Industry Launches Disease Eradication Plan to Prevent 'BSE-Style' Disaster*. Farm Ireland (2017). Available from: http://m.independent.ie/business/farming/dairy/dairy-industry-launches-disease-eradication-plan-to-prevent-bsestyle-disaster-35269781.html

Conflict of Interest Statement: Kuenstner, Naser, Chamberlin, Borody, Dow, John and Amy Hermon-Taylor, Aitken, Bull, Bach, and Zhang have proprietary interests in MAP therapies or diagnostic tests. All other authors declare that the research was conducted in the absence of any commercial or financial relationships that could be construed as a potential conflict of interest.

Copyright © 2017 Kuenstner, Naser, Chamberlin, Borody, Graham, McNees, Hermon-Taylor, Hermon-Taylor, Dow, Thayer, Biesecker, Collins, Sechi, Singh, Zhang, Shafran, Weg, Telega, Rothstein, Oken, Schimpff, Bach, Bull, Grant, Ellingson, Dahmen, Lipton, Gupta, Chaubey, Singh, Agarwal, Kumar, Misri, Sohal, Dhama, Hemati, Davis, Hier, Aitken, Pierce, Parrish, Goldberg, Kali, Bendre, Agrawal, Baldassano, Linn, Sweeney, Fecteau, Hofstaedter, Potula, Timofeeva, Geier, John, Zayanni, Malaty, Kahlenborn, Kravitz, Bulfon, Daskalopoulos, Mitchell, Neilan, Timms, Cossu, Mameli, Angermeier, Jelic, Goethe, Juste and Kuenstner. This is an open-access article distributed under the terms of the Creative Commons Attribution License (CC BY). The use, distribution or reproduction in other forums is permitted, provided the original author(s) or licensor are credited and that the original publication in this journal is cited, in accordance with accepted academic practice. No use, distribution or reproduction is permitted which does not comply with these terms.
