# Supplementary material for: Long History of Queries about Bovine Paratuberculosis as a Risk Factor for Human Health
Source: Pathogens. 2021 Oct 28;10(11):1394. doi: 10.3390/pathogens10111394 (PMC8622788; doi:10.3390/pathogens10111394)
Supplement: Supplementary file 1 [file pathogens-10-01394-s001.zip › LIPTON.pdf]

11/25/05

The New Paradigm for Crohn's Disease: A Call to Action

By Judith Eve Lipton, M.D., J. Todd Kuenstner, M.D., M.S., David Barash, Ph.D., and James Biesecker M.D., Ph.D.

As physicians and scientists who have been personally affected by Crohn's Disease, we write with a sense of urgency. We do not simply care for patients with this illness. Each of us suffers from it, or has an intimate family member who does so. We therefore find it both exciting and frustrating that growing evidence points to a microbial, infectious cause of Crohn's Disease: exciting because genuine prevention and cure (as opposed to mere palliation) may be achievable, but frustrating because the public as well as the medical community remains largely unaware of this possibility, while hundreds of thousands of people, many of them children, suffer from the illness itself or iatrogenic complications.

Most gastroenterologists consider Crohn's to be an autoimmune disease, and treat it as such. However, scientific evidence is mounting that bacterial infection with *Mycobacterium avium* ssp. *paratuberculosis* (MAP), can present as Crohn's Disease. MAP is a variant of Mycobacterium avium complex (MAC), and is thus related to *Mycobacterium tuberculosis* and *Mycobacterium leprae.*

Standard treatments for Crohn's Disease are unsatisfactory. Overall quality of life for people who are treated with steroids, immune suppressors and immune modulators is not good, because the drugs are variably efficacious and have horrific side effects. Two of us have children with Crohn's, one is a physician with Crohn's, and one is the husband of a woman with Crohn's. After the diagnoses, we each independently researched the scientific literature, and separately found the connection between MAP and Crohn's, which, for whatever reason, had not been digested by our own gastroenterologists.

James Biesecker, M.D., Ph.D. (a pathologist) learned about MAP when his 14 year old son Tristan was hospitalized with Crohn's. Dr. Biesecker flew to Australia to meet Dr. Thomas Borody, a gastroenterologist and infectious disease specialist, and an expert on anti-MAP therapy for Crohn's. Dr. Biesecker started treating his son Tristan with antibiotic drugs in 2002; Tristan went into remission. Now 17, Tristan has regained weight and strength, and is currently a nationally ranked tennis star.

Judith Lipton, M.D., (a psychiatrist) had severe Crohn's, and was unstable on steroids, azathioprine and infliximab. She learned about MAP while sick and home in bed, after conventional therapies failed. She lives on a small farm and keeps livestock; accordingly, she was disconsolate at the prospect of permanent immune suppression. With Dr. Borody's help, she began antibiotics in December, 2004, tapered off the immune suppressors, and has been in remission ever since.

Todd Kuenstner M.D., M.S. (a pathologist), learned about MAP in July, 2004, the day his then 9 year old son Will was diagnosed with Crohn's, with typical clinical signs and symptoms including marked weight loss, anorexia, abdominal pain and diarrhea. At endoscopy, Will had several small aphthous ulcers in the colon which, upon biopsy, showed classic granulomas. MAP was cultured from his blood. Will was treated for MAP and went into remission.

The authors met online and then on the telephone through the auspices of Dr. William Chamberlin in El Paso, Texas, and Dr. Borody. We are a privileged group: As physicians and scientists, we have been able to interpret the emerging literature and either prescribe appropriate antibiotic regimens or persuade colleagues to do so; the general public lacks this option. This disparity is simply unfair.

Clinical signs of mycobacterial infection evidently occur when the host is genetically susceptible, has compromised immunity, and/or is exposed to a sufficiently large inoculum of the pathogen.[1] This is a highly controversial topic,[2, 3] the terms of which changed dramatically when MAP was recently cultured from the gut lesions, blood and breast milk of Crohn's patients, as compared with normal controls.[4, 5] Skeptics claim that MAP bacteremia is a secondary consequence of Crohn's, resulting from colonization of diseased mucosa and a "leaky bowel." This explanation does not account for the clinical improvement in Crohn's patients following anti-MAP therapy. (A similar claim was made for *H. pylori* and gastric ulcers.)

A MAP-Crohn's connection proceeds from Johne's disease, an inflammatory intestinal disorder of cattle, sheep, goats and other animals, whose manifestation bears an uncanny similarity to Crohn's in human beings. Johne's disease is unquestionably caused by MAP**;** all four of Koch's Postulates have been achieved. The evidence for a MAP causation of Crohn's Disease fails to meet only one of Koch's postulates. MAP has been identified in Crohn's patients, and cultured from them (interestingly, no one has yet cultured *M. leprae*, although its connection to leprosy is undisputed). PCR-based molecular identification has, moreover, confirmed the presence of MAP DNA and RNA in the blood and gastrointestinal lesions of Crohn's patients.[6,7,8] In addition, persistent and aggressive antibiotic protocols for MAP utilizing knowledge gained from the treatment of *M. tuberculosis* and MAC show promising results.[9,10] It appears that Crohn's is a zoonotic infection, transmitted to people via the consumption of MAP-infected milk, meat, and/or water. All that remains to fulfill Koch's postulates is a MAP ingestion study**:** perhaps one of the skeptics wishes to enlist a large group of young family members and friends to drink MAP-shakes and eat MAP-burgers, as a community service. Otherwise, a feeding study in primates ought to suffice. Eradication of MAP bacteremia from humans, associated with clinical improvement, would also be highly significant.

Like *M. tuberculosis* and *M. leprae*, MAP is difficult to culture. Clinical trials of conventional antibiotics showed poor to mixed results, consistent with the fact that Mycobacteria generally are highly resistant to traditional antibiotics.

As a result, earlier medical literature was equivocal, and so a possible MAP-Crohn's connection was largely discounted. But now, a genuine paradigm shift is underway, analogous to the revolution in tuberculosis treatment when Koch discovered that the tubercle bacillus - and not bad air or an artistic temperament – caused tuberculosis. Current treatments for Crohn's involving immune suppression are like treating tuberculosis with surgery, sanitariums, and mountain air. (The latter two are far less risky than is chronic immune suppression, while ironically, many unfortunate patients end up with the Crohn's equivalent of lung resection for tuberculosis; namely, bowel surgery.)

Susceptibility to Crohn's may be heritable, via the NOD2/CARD15 mutation. Interestingly, the protein encoded by the NOD2/CARD15 mutation is implicated in bacterial cell wall recognition, and a high percentage of Crohn's patients with this mutation have been shown to be infected by MAP.[11,12] Other contributing infections may also play an undetermined role in activating Crohn's. Crohn's Disease thus seems to be an exemplary nature/nurture interaction: genetically susceptible individuals, or people exposed to large amounts of MAP at a young age, develop Crohn's disease, while those with better cell wall recognition or less exposure do not. This interpretation is also consistent with emerging theory in evolutionary biology, whereby an array of human diseases evidently derive from complex host-pathogen interactions rather than some mysterious and inexplicable auto-immune dysfunction.

It has not escaped our notice that the proposed MAP-based etiology for Crohn's will not only have substantial implications for public health, but also important economic, political, and psychological consequences, given that 7-55% of dairy herds in Western Europe and North America appear to be infected with MAP.[13] Thus, we anticipate that the emerging pathogenic explanation of Crohn's will encounter considerable resistance, from industry as well as government. The situation is remarkably parallel to that experienced by Nobel Prize recipients Drs. Barry Marshall and Robin Warren, who demonstrated a bacterial cause of gastric ulcers, but whose work was strongly opposed by the medical and pharmaceutical establishment. Given heightened awareness of zoonotic infections, as evidenced by concern about a possible avian influenza pandemic, we are cautiously optimistic that in this case, the medical profession as well as the public will be amenable to our message.

We endorse the precautionary principle: when there is a significant threat of harm to human health or the environment, precautionary measures should be undertaken promptly even in the absence of indisputable proof. If even only a proportion of Crohn's disease cases are caused by infection with MAP, action should be taken to protect public health from this pathogen and to establish effective treatment regimes.

In view of the above, we urge the following:

1) The public, medical and pharmaceutical communities need to familiarize themselves with the likely infectious etiology of Crohn's Disease. This connection needs to be publicized and explored, not dismissed as the mutterings of microbiologists, rural veterinarians, activist trouble-makers, or desperate patients, vulnerable to quack cures.

2) Basic MAP-related research needs substantial support from the NIH, the FDA, the CDC, the Crohn's & Colitis Foundation of America, and pharmaceutical companies. Current funding for MAP research is miniscule, which is a gross misallocation of resources devoted to Crohn's research.

3) The USDA should give greater attention to Johne's disease. Independent of its zoonotic potential, this complex and incompletely understood disease is already a huge problem for agriculture, resulting in more than $1.5 billion in US herd losses annually. No one in North America has contracted Mad Cow Disease, although the mere prospect has been galvanizing. By contrast, between 1 out of 500 and 1 out of 800 North Americans are afflicted with Crohn's.[14, 15]

4) Vigorous public health measures are needed now to prevent further transmission of MAP. Such measures should include, but not be limited to, federally mandated Johne's disease control programs for dairy, beef cattle, goats and sheep. Farmers should be required to institute "best practice management" for their animals so that MAP and other infectious diseases can be better controlled. MAP needs to be eliminated from the food chain in general and human grocery products in particular. At present, cattle with advanced stage

Johne's disease (i.e., disseminated MAP infection), are being slaughtered and fed to the public[16]; this practice is unacceptable.

5) The FDA needs to ban the distribution and sale of MAP infected milk. Improved methods of pasteurization should be developed. Conventional pasteurization does not reliably kill MAP, and so milk containing this pathogen in viable form is typically retailed in the United States, thereby putting the public at risk.[17]

7) Human and animal vaccines for MAP need to be developed.

8) Expert practice guidelines need to be developed and disseminated to practicing gastroenterologists and infectious disease specialists so that clinicians can proceed with Anti-MAP therapy when MAP infection is demonstrated or strongly suspected.

9) Reliable tests including serology, culture, and PCR need to be developed and made available to practicing physicians. Ironically, veterinarians frequently evaluate animals for MAP using ELISA tests and fecal cultures, whereas physicians have no readily available comparable tests for human patients.

10) Double blind, randomized, controlled studies are needed to evaluate contemporary medications efficacious for MAC for Crohn's patients. Such studies should test multiple drug combinations, as is the standard of care for tuberculosis. These drugs include but are not limited to the macrolides, rifabutin, fluoroquinolones, ethambutol, and clofazimine. Open label clinical trials using many of the above medications suggest that some patients will obtain durable

remissions. However, relapse even with antibiotics is not uncommon, and there is no clinically established end point for treatment.

11) Therefore, we also recommend that pharmaceutical companies develop more effective MAP eradication therapies rather than life-long immunosuppressive treatment. Drugs that boost the ability of macrophages to respond to MAP ought to be more effective and less toxic than antibiotics.

A scientific revolution is underway, implicating an infectious agent, namely *Mycobacterium avium paratuberculosis* (MAP) in the etiology of Crohn's disease. We think compelling evidence suggests that Crohn's Disease can be caused by a zoonotic infection that could be prevented with sensible public health measures and improved farming practices. There are, moreover, exciting treatment options for eradication of the bacterium as well as for the enhancement of appropriate host responses to infection. It is socially as well as scientifically irresponsible to ignore or minimize this problem. We, as medical professionals, can and must lead the way toward preventing and curing Crohn's Disease.

---

[1] Grant, I., Zoonotic potential of Mycobacterium avium ssp. paratuberculosis: the current position. *Journal of Applied Microbiology.* 2005. 98: 1282–1293.

[2] Sartor, R.B., Does Mycobacterium avium subspecies paratuberculosis cause Crohn's disease? *Gut.* 2005;54: 896-898.

[3] Cohen, R.D., Mycobacterium in Crohn's: Something to Ruminate About? *Gastroenterology* 2005;128:2167–2174

[4] Naser, S.A., Ghobrial, G., Romero, C., Valentine, J.F. Culture of Mycobacterium avium subspecies paratuberculosis from the blood of patients with Crohn's Disease, *The Lancet.* 2004; 364:1039-1044.

[5]Naser, S.A., Schwartz, D. Isolation of *Mycobacterium avium* subsp.*paratuberculosis* from breast milk of Crohn's disease patients. *Am. J. Gastroenterol.2000;* 95:1094 10995.

[6] Greenstein, R.J., Is Crohn's disease caused by a mycobacterium? Comparisons with leprosy, tuberculosis, and Johne's disease. *The Lancet Infec Dis.* 2003: 3; 507-514.

[7] Bull, T.J., McMinn, L., Sidi-Boumedine, K., Skull, A., Durkin, D., Neild, P., Rhodes, G., Pickup, R., Hermon-Taylor, J. Detection and verification of *Mycobacterium avium* subspecies *paratuberculosis* in fresh ileocolonic mucosal biopsy specimens from individuals with and without Crohn's disease. *J. Clin. Microbiol. 2003;* 2915-23.

[8] Autschbach,F., Eisold, S., Hinz,U., Zinser, S., Linnebacher, M., Giese, T., Loffler, T., Buchler, M.W., Schmidt J. High prevalence of *Mycobacterium avium* subspecies *paratuberculosis* IS900 DNA in gut tissues from individuals with Crohn's disease. *Gut.* 2005;54:944 – 949.

[9] Hermon-Taylor, J., Treatment with drugs active against *Mycobacterium avium* subspecies *paratuberculosis* can heal Crohn's disease: more evidence for a neglected Public Health tragedy. *Digest. Liver Dis. 2004;* 34:9-12.

[10] Shafran, I., Kugler, L., El-Zaatari, F.A.K. and Sandoval, J. Open clinical trial of rifabutin and clarithromycin therapy in Crohn's disease. *Digest Liver Dis.* 2002;34**:** 22-28.

[11] Hugot, J-P., Chamaillard, M., Zouali, H., Lesage, S., Cezard, J-P., Belaiche, J., Almer, S., Tysk, C., O'Morain, C.A., Gassuli, M., Binder, V., Finkel, Y., Cortot, A., Modigliani, R., Laurent-Puig, P., Gower-Rousseau, C., Macry, J., Colombel, J-F., Sahbatou, M. and Thomas, G. Association of NOD2 leucine-rich repeat variants with susceptibility to Crohn's disease. *Nature* 2001; 411, 599-603.

[12] Sechi, L.A., Gazouli, M., Ikonomopoulos, J., Lukas, J.C., Scanu, A.M., Niyaz, A., Fadda, G., Zanetti, S., *Mycobacterium avium* subsp. *Paratuberculosis*: Genetic Susceptibility to Crohn's Disease, and Sardinians: the Way Ahead *Journal of Clinical Microbiology.* 2005; 43,10:5275–5277

[13] Grant, op. cit. page 1283

[14] Loftus, E.V., Silverstein, M.D., Sandborn, W.J., Tremaine, W.J., Harmsen, W.S., Zinsmeister, A.R., Crohn's disease in Olmsted County, Minnesota, 1940-1993: incidence, prevalence, and survival. *Gastroenterology*. 1998;114(6):1161-1168.

[15]Bernstein, C.N., JF Blanchard, J.F., Rawsthorne, P., Wajda, A., Epidemiology of Crohn's disease and ulcerative colitis in a central Canadian province: a population-based study *American Journal of Epidemiology*. 1999;149, 10: 916-924.

[16] Rossiter, C. *Journal of Animal Sciences*. 2001; 79: 113 (abstract #471)

[17] Ellingson, J.L., Anderson, J.L., Koziczkowski, J.J., Radcliff, R.P., Sloan, S.J., Allen, S.E., Sullivan, N.M. Detection of Viable Mycobacterium avium subsp. paratuberculosis in Retail Pasteurized Whole Milk by Two Culture Methods and PCR. *Journal of Food Protection*. 2005; *68:*966–972.

Email contact: jelipton@gmail.com
