# Supplementary material for: Long History of Queries about Bovine Paratuberculosis as a Risk Factor for Human Health
Source: Pathogens. 2021 Oct 28;10(11):1394. doi: 10.3390/pathogens10111394 (PMC8622788; doi:10.3390/pathogens10111394)
Supplement: Supplementary file 1 [file pathogens-10-01394-s001.zip › NACY.pdf]

A REPORT FROM THE AMERICAN ACADEMY OF MICROBIOLOGY

MYCOBACTERIUM AVIUM PARATUBERCULOSIS:

Infrequent Human Pathogen or Public Health Threat?

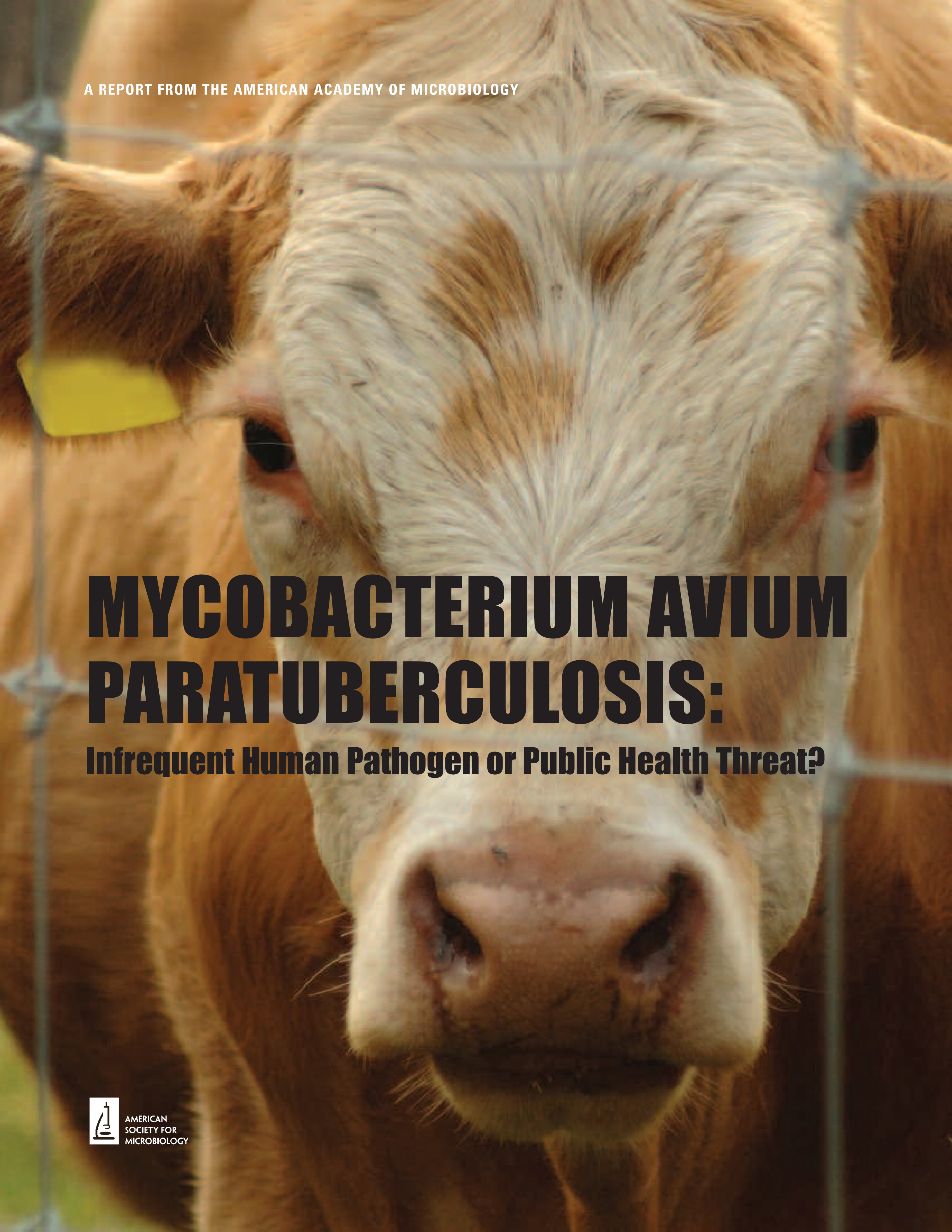

AMERICAN
SOCIETY FOR
MICROBIOLOGY

MYCOBACTERIUM AVIUM PARATUBERCULOSIS:
Infrequent Human Pathogen or Public Health Threat?

BY CAROL NACY, PH.D., AND MERRY BUCKLEY, PH.D.

This report is based on a colloquium, sponsored by the American Academy of Microbiology, convened June 15-17, 2007, in Salem, Massachusetts.

The American Academy of Microbiology is the honorific leadership group of the American Society for Microbiology. The mission of the American Academy of Microbiology is to recognize scientific excellence and foster knowledge and understanding in the microbiological sciences. The Academy strives to include underrepresented scientists in all its activities.

The American Academy of Microbiology is grateful for the generosity of the following organizations for support of this project:

- U.S. Department of Agriculture, Animal and Plant Health Inspection Service (APHIS)
- U.S. Department of Agriculture, Cooperative State Research, Education, and Extension Service (CSREES)
- U.S. Department of Agriculture, CSREES, Johne's Disease Integrated Program (USDA-CSREES-NRI Award No. 2007-01019)
- BD
- Broad Foundation
- Canadian Foundation for Infectious Diseases
- Crohn's & Colitis Foundation of Canada
- Giaconda Limited
- Hawk Corporation
- Kauver Foundation
- Oxford Immunotec
- Sequella, Inc.
- TREK Diagnostic Systems, Inc.

The opinions expressed in this report are those solely of the colloquium participants and do not necessarily reflect the official positions of our sponsors or the American Society for Microbiology.

Copyright © 2008
American Academy of Microbiology
1752 N Street, NW
Washington, DC 20036
http://www.asm.org

MYCOBACTERIUM AVIUM PARATUBERCULOSIS:
Infrequent Human Pathogen or Public Health Threat?

BOARD OF GOVERNORS, AMERICAN ACADEMY OF MICROBIOLOGY

R. John Collier, Ph.D. (Chair)
Harvard University Medical School

Kenneth I. Berns, M.D., Ph.D.
University of Florida Genetics Institute

Edward DeLong, Ph.D.
Massachusetts Institute of Technology

E. Peter Greenberg, Ph.D.
University of Washington

Carol A. Gross, Ph.D.
University of California, San Francisco

Lonnie O. Ingram, Ph.D.
University of Florida

J. Michael Miller, Ph.D.
Centers for Disease Control
and Prevention

Stephen A. Morse, Ph.D.
Centers for Disease Control
and Prevention

Edward G. Ruby, Ph.D.
University of Wisconsin-Madison

Patricia Spear, Ph.D.
Northwestern University

George F. Sprague, Jr., Ph.D.
University of Oregon

COLLOQUIUM STEERING COMMITTEE

Carol A. Nacy, Ph.D. (Chair)
Sequella, Inc., Rockville, Maryland

Marcel A. Behr, M.D.
Montreal General Hospital, Montreal,
Québec, Canada

Charles N. Bernstein, M.D.
University of Manitoba, Winnipeg,
Manitoba, Canada

Judith Lipton, M.D.
Redmond, Washington

Mary E. Torrence, D.V.M., Ph.D.
U.S. Department of Agriculture (ARS),
Greenbelt, Maryland

Carol Colgan, Director
American Academy of Microbiology

SCIENCE WRITER

Merry Buckley, Ph.D.
Ithaca, New York

MYCOBACTERIUM AVIUM PARATUBERCULOSIS:
Infrequent Human Pathogen or Public Health Threat?

COLLOQUIUM PARTICIPANTS

Douwe Bakker, Ph.D.
Central Institute for Animal Disease Control, CIDC-Lelystad, The Netherlands

Marcel A. Behr, M.D.
Montreal General Hospital, Montreal, Québec, Canada
(grant support from the Broad Foundation and the Crohn's & Colitis Foundation of Canada)

Charles N. Bernstein, M.D.
University of Manitoba, Winnipeg, Manitoba, Canada
(grant support from the Crohn's & Colitis Foundation of Canada)

Edgar C. Boedeker, M.D.
New Mexico VA Health Science Center, Albuquerque, New Mexico

Thomas J. Borody, M.D., Ph.D.
Center for Digestive Diseases, Five Dock, New South Wales, Australia
(grant support from the Broad Foundation and shareholder and chief medical officer, Giaconda)

Sheldon T. Brown, M.D.
Veterans Affairs Medical Center, Bronx, New York

Michael T. Collins, D.V.M., Ph.D.
School of Veterinary Medicine, University of Wisconsin-Madison
(consultant: BD Diagnostic Systems)

Daniel N. Frank, Ph.D.
University of Colorado, Boulder

Yrjo T. Grohn, D.V.M., Ph.D.
Cornell University

John Hermon-Taylor, FRCS, MChir
St. George's Hospital Medical School, London, England

Vivek Kapur, Ph.D.
University of Minnesota

Preston Linn, M.S.
Durham, North Carolina

Judith Lipton, M.D.
Redmond, Washington

Elizabeth J.B. Manning, D.V.M., M.P.H.
School of Veterinary Medicine, University of Wisconsin-Madison

Carol A. Nacy, Ph.D.
Sequella, Inc., Rockville, Maryland
(founder, chief executive officer, and chair of the board, Sequella, Inc.)

Saleh A. Naser, Ph.D.
University of Central Florida, Orlando, Florida
(grant support from the Broad Foundation)

Mihai G. Netea, M.D.
Radboud University Nijmegen Medical Center, The Netherlands

Warwick Selby, MBBS, M.D., FRACP
University of Sydney, New South Wales, Australia

Mary E. Torrence, D.V.M., Ph.D.
U.S. Department of Agriculture (ARS), Greenbelt, Maryland

SPONSOR REPRESENTATIVES

Michael A. Carter, D.V.M., M.P.H.
National Johne's Disease Program, USDA-APHIS Veterinary Services, Riverdale, Maryland

Ian Durrant, Ph.D.
Oxford Immunotec Ltd., Abington, Oxfordshire, England

Patrick McLean
Giaconda Limited, Sydney, New South Wales, Australia

Biljana Mihajlovic
Health Canada

Morris E. Potter, D.V.M.
U.S. Food and Drug Administration, Atlanta, Georgia

Nadine M. Sullivan, Ph.D.
TREK Diagnostic Systems, Inc., Sun Prairie, Wisconsin

Ronald E. Weinberg, M.B.A.
Hawk Corporation, Cleveland, Ohio

EXECUTIVE SUMMARY

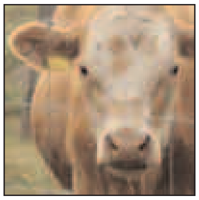

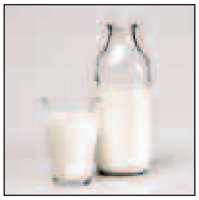

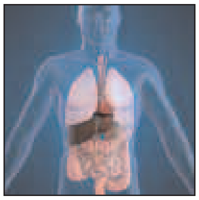

Crohn's Disease (CD) is a devastating illness in search of a cause and a cure. More than 800,000 people in North America suffer from CD, a gastrointestinal disorder characterized by severe abdominal pain, diarrhea, bleeding, bowel obstruction, and a variety of systemic symptoms that can impede the ability to lead a normal life during chronic episodes that span months to years. Researchers and clinicians agree that onset of CD requires a series of events; implicated are certain inherited genetic traits, an environmental stimulus, and an overzealous immune and inflammatory response. The combination of these factors contributes to a disease whose course is variable among patients and whose symptoms range from mild to devastating on any given day. The economic and social impact of this disease is substantial for the patient, the family, the community, and the healthcare system.

Long considered an autoimmune and chronic inflammatory disorder, current CD therapies are designed to treat symptoms of overactive inflammation in the gut. Chronic inflammation, however, does not generally induce itself. Inflammation is normally caused by a "foreign body," an inanimate object (i.e., splinter) or animate objects like rogue cells (i.e., cancer) or microorganisms (i.e., bacterium, virus, or fungus). Until the cause of inflammation is eliminated, the body continues to send in its clean-up crew, the white blood cells of inflammation whose job it is to expel the tissue invader. Inflammation only subsides when the causative agent is finally banished.

There is suspicion, supported by reports of genetic inability to interact appropriately with certain bacteria or bacterial products in some patients, that CD may have a currently unrecognized infectious origin, perhaps environmentally derived. That CD is a set of wide-ranging symptoms, more like a syndrome than a specific disease, suggests that if its origin is microbial, more than one etiologic agent may ultimately be identified. Bacterial suspects at the moment include a Mycobacterium and a variant of the normal bacterial flora of the gut, *Escherichia coli.* The possibility of more than one infectious cause that leads to a similar set of symptoms confounds the research agenda to find both a cause and a cure for CD.

One acknowledged potential microbial agent of CD is *Mycobacterium avium* subspecies *paratuberculosis* (MAP), a microorganism that causes a gastrointestinal disease similar to CD in ruminants, including dairy cattle, called Johne's disease (or paratuberculosis). People with CD have 7:1 odds of having a documented presence of MAP in blood or gut tissues than those who do not have CD, thus the association of MAP and CD is no longer in question (see Figure 1, page 11). The critical issue today is not whether MAP is associated with CD, but whether MAP causes CD or is only incidentally present, not an inciter or participant in the disease process.

If MAP is involved in the disease process of CD or other gastrointestinal disorders, then we need to determine how people are exposed to this microorganism, how to prevent that exposure, and how to treat the infection.

Despite its prevalence in the U.S. population in numbers that exceed most cancers, CD is not a focus of research attention in the same way as these other feared diseases. The American Academy of Microbiology convened a colloquium with experts in medicine, microbiology, veterinary pathology, epidemiology, infectious diseases, and food safety to describe the state of knowledge about the relationship between MAP and CD and to make recommendations for effective research that will move the field forward.

The general consensus of the assembled experts was that there are certainly reasons to suspect a role for MAP in CD:

- MAP persists in contaminated soil and water, which links the environmental factor of CD to the disease.

- MAP has a cell wall that contains muramyl dipeptide (MDP). One genetic trait that is affiliated in certain patients with CD is the NOD2 gene, which regulates ability to respond appropriately to MDP, thus the link between the genetic trait and MAP, or other bacteria.

- MAP causes Johne's disease, an illness of cattle and other ruminants that has many similarities with CD. The similarities of MAP disease in animals, for which the etiologic agent is known, and CD, for which the etiologic agent is unknown, provide a symptomatic link between agent and disease.

- MAP can survive standard milk pasteurization processes and has been identified in off-the-shelf milk in retail grocery stores in the U.S. and the European Union (E.U.). There is increasing concern that MAP can also be found in cheese made from the milk of MAP-infected cattle and meat from Johne's diseased animals. These observations could provide the exposure route of CD patients to MAP.

- Treatment of some CD patients with antibiotics that have activity on certain other Mycobacteria, although not specifically selected for their activity against MAP, provides short-term or long-term relief or remission of symptoms.

Circumstantially, these observations appear to make a compelling case for MAP as involved in CD. On the other hand, the ability to definitively identify MAP as the cause of CD, or the cause of a significant number of CD cases, has been stymied by the elusive characteristics the organism itself, the lack of broadly available and validated clinical tools to easily and definitively identify MAP in accessible tissues, and the late symptomatic stage at which CD is finally diagnosed, where the origin of the destructive inflammation could have

been years before the patient sought medical care. Most important, however, is the lack of resources, financial and scientific, to generate the tools that clinicians and patients need to determine whether MAP is involved in the disease process or not.

Several important clinical trials of antibiotics have been attempted in CD patients, with variable results. Treating CD patients with existing antibiotics with activity against other Mycobacteria (*M. tuberculosis*, which causes TB, and *M. avium* complex, or MAC, which is pathogenic in immune compromised persons) have either failed to provide relief (TB drugs) or produced promising outcomes for some patients, but not all (MAC drugs). Confounding these clinical results is the lack of information about which patients in the clinical trial population were actually infected with MAP, and whether any MAP organisms in infected patients were susceptible to the antibiotics used in the trials. Without sensitive and specific diagnostics that can detect early MAP infection, knowledge of how and where to isolate MAP for antibiotic susceptibility studies, and drugs that are known to be active against MAP itself, alone or in combination, the role of MAP in CD will remain circumstantial and the controversy over CD etiology will continue.

There is little known about where exactly viable MAP can be found in human tissues or, since most pathogenic Mycobacteria are intracellular, in which cells MAP can live and grow in humans. While the site of infection and tissue pathologies of MAP in animals can be assessed at necropsy, there is enough dissimilarity between digestive processes of ruminants and humans that this information may not necessarily inform studies in humans.

Of concern from a public health perspective is the ongoing presence of MAP disease in commercial livestock that supply the U.S. with dairy and meat products. If, in fact, CD is a zoonotic infection (one that is passed from animals to humans) and MAP is the (or one) cause of CD, then early identification of MAP disease in veterinary practice and appropriate management of these animals to safeguard the food supply will be critical to guard the public health.

Even in animals, it is nearly impossible to diagnose Johne's disease in the early stages of disease. Diagnosis is by a combination of clinical observation (wasting and reductions in milk production in dairy cattle, for instance) and microbiological, histopathological, and immunological testing of Johne's disease suspects. Although efforts to eliminate Johne's disease and MAP from livestock herds are ongoing, the lack of an accurate and easily-administered diagnostic for early disease onset is hampering these efforts. The results are mixed, and food products containing MAP or MAP DNA can be found on supermarket shelves. Veterinary diagnostics that are sensitive (detect MAP at early stages of infection) and specific (identify MAP and not other microorganisms) will be necessary to eliminate Johne's disease from the commercial food supply. Research to discover and validate these techniques may also shed light on diagnosis of human disease.

Colloquium participants agreed that research to elucidate the role of MAP in CD must address two major unknowns: (1) whether MAP from livestock and other animals is transmissible to humans and how it is transmitted and (2) whether humans are susceptible to infection and disease after exposure to MAP. No single study will fill all the gaps in our understanding of the possible relationship between MAP and CD. Furthermore, participants agreed that validated, reproducible biological markers confirming human MAP infection are desperately needed. If MAP can be causally associated with CD using reproducible analytical techniques, appropriate patient populations can be treated with antibiotics that are selected for their MAP activity. Then, at least MAP-infected CD patients will have both a cause and a cure.

INTRODUCTION

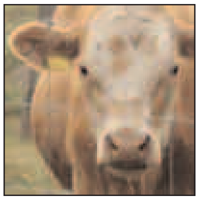

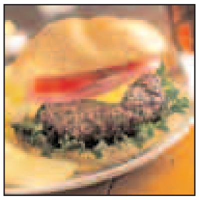

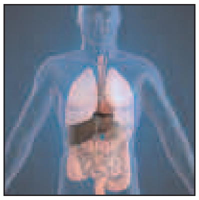

CROHN'S DISEASE (CD)

CD was named after Burrill B. Crohn, an American physician who published a paper in 1932 clearly distinguishing CD from intestinal TB (1). CD is a syndrome characterized by chronic and debilitating inflammation of the gastrointestinal tract that can be accompanied by mild to excruciating pain, frequent bouts of diarrhea, and malnutrition due to rapid passage of food through the inflamed intestinal tract. Some patients must be fed intravenously during the most difficult of their episodes. These devastating and episodic symptoms can force patients to maintain a limited work schedule or to refrain from working altogether, and the psychological effects of the disease are profound. The uncertainty of their condition and the ever-present possibility of symptomatic flare-ups often drive patients into anxiety, depression, and isolation. As of 2001, the Centers for Disease Control and Prevention estimated that over 500,000 people in the U.S. are living with CD (2), and more recent statistics show that 800,000 people in North America and 1 in 1000 persons in westernized countries have diagnosed CD. In the U.S., roughly 50% of CD patients are children. There is currently no cure for CD.

With the right anti-inflammatory drug or immunomodulatory biologic regimen, CD patients can experience temporary remission of symptoms, but long-term flare-ups inevitably follow, coming on suddenly or developing gradually over time. Surgery to remove inflamed sections of the bowel is the only option to alleviate the symptoms of many people living with CD. Many other patients endure endless regimens of drugs to alleviate some of their symptoms, but these drugs, too, have side effects, some that can even be life threatening.

Table 1. Drugs for Therapy of Crohn's Disease, 2008[1]

CLASS	DRUG	DOSE	TABLET SIZE	AV. WHOLESALE PRICE/TAB ($)[2]	AV. DOSE (70KG)	# TABLETS	COST/DAY	COST/MO	DURATION	NOTES
**GUT ANTI-INFLAMMATORY**										
	sulfazalazine	3g/day	500mg	0.50	3 grams	6	$3.00	$90.00	ongoing	
	olsalazine	1g/day	250mg	1.66	1 gram	4	$7.00	$210.00	ongoing	
	balsalazide	6.75 grams	750mg	4.45	6.75 grams	9	$40.00	$1200.00	ongoing	
	mesalamine	3 grams	400mg	1.40	3 grams	8	$11.00	$330.00	ongoing	
	mesalamine suppository	1 gram	1gm	5.41	1 gram	2	$10.82	$325.00	acute	
	mesalamine	4 grams	enema 60cc	25.86		1	$25.86	$776.00	acute	
	mesalamineXR	4 grams	500mg	1.79	4 grams	8	$14.00	$420.00	ongoing	
**STEROIDS**										
	prednisone	20-60mg	20mg	0.10	40mg	2	$0.20	$6.00	acute, then taper	
	dexamethasone	0.75-9.0mg	4mg	0.58	4mg	1	$0.58	$17.00	acute use only, taper	
	prednisolone	5-60mg	5mg	0.03	40mg	8	$0.24	$7.00	acute increase then taper	
	hydrocortisone	20-240mg	10mg	0.41	20mg	2	$0.82	$25.00	acute, then taper if possible	
	budesonide	9mg	3mg	5.72	9mg	3	$17.00	$510.00	acute, taper	
**ANTIBIOTICS**										
	ciprofloxacin	1 gram	500mg	5.37	1 gram	2	$10.74	$322.20	2-4 weeks	cost offset by medicare
	metronidazole	1 gram	250mg	1.45	1 gram	4	$5.80	$174.00	2-4 weeks	
	ethambutol	15mg/kg	100mg	0.60	1 gram	10	$6.00	$180.00	ongoing	
	rifabutin	600mg	150mg	10.07	600mg	4	$40.28	$1208.00	ongoing	
	clarithromycin	1 gram	500mg	4.52	1 gram	2	$9.04	$271.00	ongoing	
	azithromycin	500mg	500mg	15.55	500mg	1	$15.55	$467.00	ongoing	
	rifaximin	600mg	200mg	3.72	600mg	3	$11.00	$330.00	ongoing	
	clofazimine	100mg	100mg	n/a	100mg	1			ongoing	n/a in U.S. Free in rest of world
**BORODY COCKTAIL (CLARITHROMYCIN, RIFABUTIN, CLOFAZIMINE)**										
							$49.32	$1479.00	ongoing	Assumes clofazimine is free
**IMMUNE SUPPRESSANTS**										
	azathioprine	2.5mg/kg/d	50mg	1.31	100mg	2	$2.62	$79.00	ongoing	Requires hepatic testing
	6-mercaptopurine	2mg/kg/d	50mg	4.08	100mg	2	$8.16	$244.80	ongoing	
	methotrexate	25mg IM/wk	2.5mg	3.56	25mg	I0 units	$5.09	$152.70	ongoing	
	cyclosporine	2.5-15 mg/kg/d	100mg	1.65	7mg	7	$49.50	$1485.00	ongoing	
	tacrolimus	0.1-0.2 mg/kg/d	5mg	23.82	10.50mg	5	$119.10	$3573.00	ongoing	investigational

Table 1. Drugs for Therapy of Crohn's Disease, 2008[1]

CLASS	DRUG	DOSE	TABLET SIZE	AV. WHOLESALE PRICE/TAB ($)[2]	AV. DOSE (70KG)	# TABLETS	COST/DAY	COST/MO	DURATION	NOTES
**BIOLOGICAL RESPONSE MODIFIERS**										
	infliximab	5mg/kg IV at wk 0,2,6						$2542.00 /infusion		Cost of infusion ~ same as AWP Initial cost = $7,626.00
		5mg/kg IV every 8 weeks		726.18/100mg	350mg			$1271.00 /mo average		Cost of infusion ~ same as AWP IV infusion total cost ~$6,000/2 mo
	adalimumab	40mg/wk SQ	40mg			1 pen/wk		Starter pack $5100.00		
		40mg/wk SQ	40mg	866/pen		1 pen/wk		$3464.00		
	thalidomide	50-300mg	100mg	190.74		100mg	$190.74	$5722.20	investigational	Immune suppressant, now considered TNF inhibitor
	natalizumab	300mg IV q 4 weeks		n/a					investigational, for MS only	
	alicaforsen	n/a							investigational	
	sargromostim	6mg/kg/d SQ	500mg	373.36					investigational	
	cortolizumab	n/a							investigational	
**STEM CELLS**										
	Stem Cell Transplant	n/a						$100,000	investigational	Risk: sepsis/death from infection
**ANTIDIARRHEAL AGENTS**										
	diphenoxylate	1 tab QID		0.42		4	$1.68	$50.40		
	paregoric	5-10cc up to QID		0.70 per 5cc	10cc		$1.40	$42.00		Narcotic, not for long term use
**PROBIOTICS**										
	Saccharomyces boulardii	1-3 caps/d						$12.80		Online cost
	VSL #3	1 pack/d					$2.65	$79.50		Online cost
**IMMUNE STIMULANTS - PARASITES**										
	hookworms							$4000	lasts 5 yr	
	pig whipworms			450.00 for 2500 eggs				$900	every 2 wk	
**UNKNOWN MECHANISM**										
	Low dose naltrexone	4.5mg/d		Compounded Regular dose is 50mg						Cost in Canada is 25.00/mo for 4.5mg tabs
**TOTAL PARENTERAL NUTRITION**										
				$5600	weekly	Medicare allows $1400/wk				

[1] Table compiled by J. Lipton, M.D. with assistance from F. Cunningham (UC Berkeley, CA) and I. Barash, Ph.D., M.D. (UCSD).

[2] Average Wholesale Prices (AWP), provided B. Faulkner, Woodinville Medical Center Pharmacy, Woodinville, WA 98072 USA on February 29, 2008. NOTE: AWP change from day to day based on information from the drug manufacturers and/ or by calculations made by drug wholesalers. The AWP does not always accurately reflect the retail cost of a drug, especially in the case of many generic drugs which can be purchased and sold below the AWP in many markets. AWP is the price used in comparing prices of drugs, although for some drugs it is not an accurate reflection of cost to the patient.

PROMINENT THEORIES ON THE ETIOLOGY OF CD

AUTOIMMUNE DISEASE

For decades, CD was considered an autoimmune disease, where the immune system erroneously identifies tissues of the gastrointestinal tract, specifically the large intestine, as “altered” or “foreign” and begins a relentless attack. In recent years, however, the pathologies associated with CD have been observed in other conditions that affect the regulation of immune and inflammatory cells to cope with bacterial pathogens, such as Chronic Granulomatous Disease (CGD) and Chronic Variable Immunodeficiency (CVI). Moreover, studies of white blood cells present in tissue biopsies from CD patients demonstrate a dysfunction in the ability of these cells to ingest particles, a preliminary event for clearance of bacteria that invade tissues (3). These more recent studies call into question the autoimmune etiology of CD. This disease does not appear to be a result of immune activities directed specifically against human tissue.

GENETIC DISORDER

Studies strongly suggest that CD has a genetic component (4, 5), one that controls an immune defect. Whole genome studies of CD patients identified an association of the disease with genes of the NOD2 family that influence the immune system (3, 5). Mutations in the NOD2 gene found in CD patients disrupt the ability of immune cells to recognize certain signals that are present in pathogenic bacteria. But it is also clear that heredity is not the full story, nor is an overactive immune system. The inflammation of CD may be due to a malfunction of the immune system that is infectious in origin, genetically controlled, and environmentally exacerbated.

ENVIRONMENTAL TRIGGER

The incidence of CD has been rising over the last several decades, as observed in multiple countries by independent investigators. There is an association between rising economic conditions and increasing reports of CD in a population. This has been interpreted as evidence that an environmental trigger is as, if not more, important in the etiology of CD as human genetics.

MICROORGANISMS

That NOD2 gene mutations are found in 25-35% of CD patients suggests a microbial factor in disease onset for at least a subset of patients. CD tissue pathology and symptoms could be due either to an inappropriate response to otherwise harmless bacteria, or to an appropriate (but ineffective) immune response to harmful bacteria that are introduced into the gut. In these scenarios, tissues of the gut become damaged and inflamed as ancillary events to the main battle, the attempt of the immune and inflammatory cells to eliminate the offending bacteria. The bacteria that have been implicated to date include certain soil Mycobacteria and variant of the normal flora of the gut, *Escherichia coli.*

MULTI-FACTORIAL ETIOLOGY

The cause (or causes) of CD is not known, and the disease is complex. It is possible that CD is a syndrome with many different origins. Considering the diverse range of symptoms in different patients, CD could result from different infectious agents and/or different underlying genetic or immune factors.

SOURCES OF MAP FOR ANIMALS AND HUMANS

Soil contains a wide variety of habitats and ecological niches that teem with life, playing host to as many as 10 million bacteria, fungi, and parasites per gram. MAP belongs to a large and diverse Mycobacteria family of bacteria, many of whose members, some 40 different species, are ubiquitous in the environment. Most of these Mycobacteria are not pathogenic for animals or humans, but live out their lives contributing to the richness and fertility of croplands or pastures. Some, like *M. kansasii*, *M. abscissium*, or *M scrofulaceum*, live primarily in the soil, but under the right circumstances can become "opportunistic pathogens" and cause disease if introduced into mammalian tissues inappropriately or inadvertently.

On the other end of the family spectrum are several mycobacterial species that are not generally found in soil and that are highly adapted to life inside mammalian hosts. These Mycobacteria are facultative intracellular pathogens that spend much of their life within mammalian white blood cells called macrophages, the very cells that should be the first line of defense against infection. They can cause serious, life-threatening chronic illnesses in both animals and humans and have been acknowledged public health threats for over a century. They belong to the *M. tuberculosis* complex (*M. tuberculosis*, *M. bovis*, *M. africanum*, *M. microti* and *M. canetti*) and are the etiologic agents of contagious and fatal tuberculosis (TB) disease in animals (from *M. bovis*) or humans (from *M. tuberculosis*). Human TB can be acquired from infected animals (a zoonosis) or from other infected humans through contaminated respiratory secretions. So pathogenic are these agents that 2 billion of the 6 billion people in the world are infected with *M. tuberculosis* (1 of every 3 people), and 2-3 million deaths from TB are recorded each year.

Species of another mycobacterial family, *M. avium* Complex (MAC), represent another threat to human health. These bacteria are found in the environment in soil and water, but certain MAC members have specifically adapted to life within particular mammalian or avian hosts. The MAC family evolved two distinct subfamilies that distinguish themselves both genetically and phenotypically (6) (see Figure 2, Page 22).

- *M. avium* subspecies avium (MAA) has evolved to be more like the soil Mycobacteria that can become opportunistic pathogens as described above. MAA strains cluster genetically into a group of free-living bacteria that replicate in the environment, especially in water. But MAA can also infect birds and human lungs, causing a transient low-pathology infection or triggering a chronic inflammation that is ultimately fatal. For example, MAA can cause a potentially fatal pneumonia in immunocompromised hosts, and is one of the more intractable opportunistic pathogens of Human Immunodeficiency Virus (HIV)-infected individuals.

- *Mycobacterium avium* subspecies paratuberculosis, or MAP, comprises a second genetic subgroup that can survive in soil or water for months to years. It is not clear whether MAP can actually replicate in the environment because it is unable to produce mycobactin, an essential iron transport chemical synthesized by every other Mycobacterium sp. Instead, MAP has learned to successfully infect and replicate inside the same white blood cells of mammals as *M. tuberculosis*, macrophages. Rather than targeting the respiratory tract, however, MAP has adapted to the gastrointestinal tract of ruminants and other animals and can cause a slowly developing, but eventually fatal, inflammatory disorder called Johne's disease.

The question the colloquium addressed is whether MAP has also evolved to infect human gastrointestinal tissue, and whether, once there, it can cause disease.

Figure 1. Mycobacteria family genealogy. MAP is genetically linked to other MAC strains, but is a genetically distinct subspecies. (Courtesy of M. Behr, McGill University, Canada)

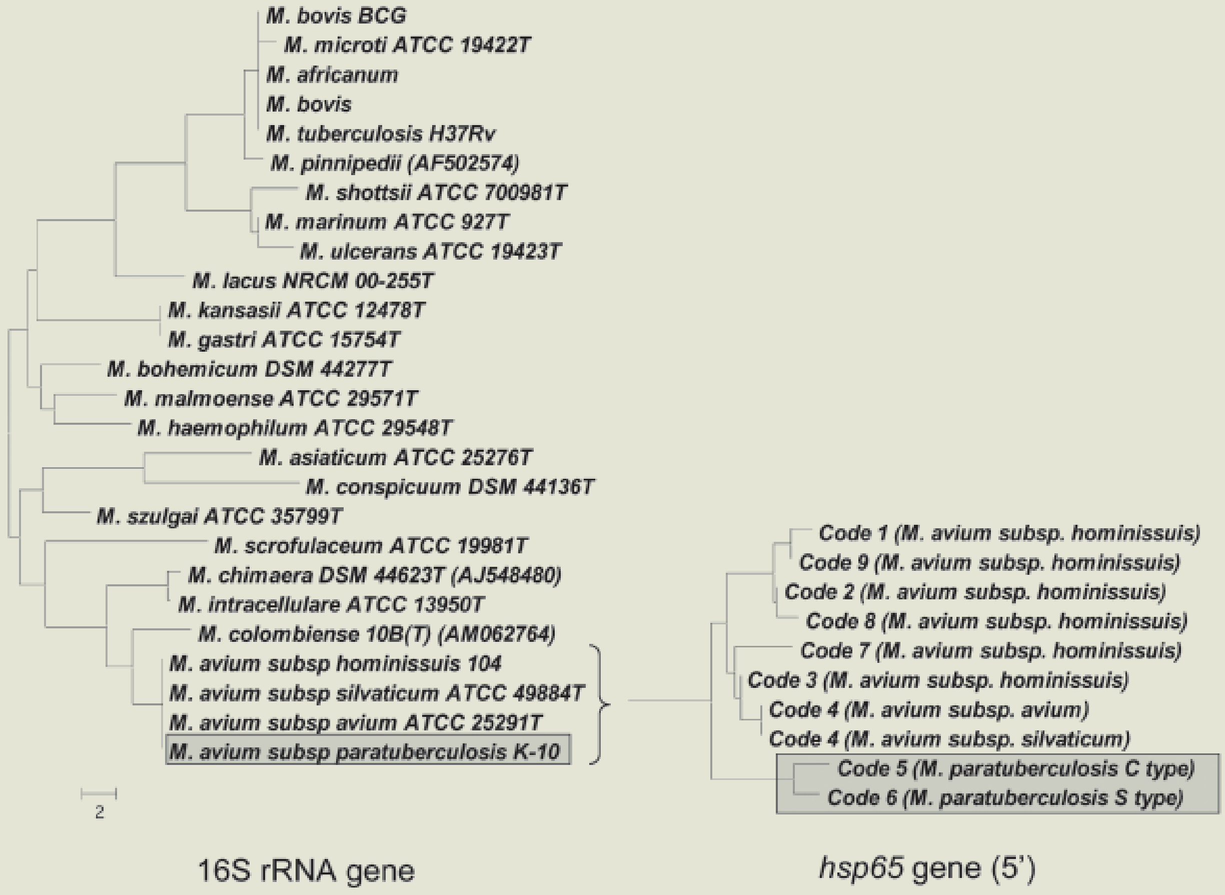

MAP AND JOHNE'S DISEASE

Johne's disease is a $1.5 billion a year cattle industry problem in the U.S. (7). Johne's disease is found in livestock herds around the globe, and though the condition is easy to identify once symptoms arise, it is challenging to diagnose in the early stages of infection. Healthy but infected animals transmit the organism through milk and manure, invisibly spreading the infection to young animals. As a result of this silent transmission, it is difficult to eliminate MAP from individual herds.

Prevalence

Although MAP, the cause of Johne's disease, has a broad host range, the most commonly infected animals are ruminants, such as cattle, sheep, goats, or deer, both wild and domestic (8). Johne's disease is more common in the more closely confined dairy cattle than in beef cattle, however, where higher animal density and more extensive premise contamination means greater contact with the organism. While there is evidence for host predisposition for certain strains of MAP, it is believed that all strains are capable of causing infection and pathology.

MAP has a broad geographic distribution, and Johne's disease has been reported on every continent in virtually every country that has animal agriculture and laboratory diagnostic capability. In 1996, 3-10% of U.S. dairy cattle were infected with MAP, and 22% of U.S. dairy herds had a MAP prevalence of >10% (9). A similar survey in 2007 reported that 68% of U.S. dairy herds are infected with MAP. In Europe, MAP infection rates among dairy cattle range from ~0% now in Sweden and Norway, where new infections are actively culled, to 50% in the Netherlands and 80% in Denmark. Infection rates vary widely across the various cattle-growing regions of Australia. Few countries are free of MAP and Johne's disease, but the reported prevalence of infected animals in any country is only as good as the diligence with which the disease is surveyed. Although difficult early diagnosis certainly contributes to the uncertainty of the actual figures, a recent increase in the number of infected herds is noticeable, including in countries that previously had low infection rates. A study ending in the fall of 2008 is expected to reveal much higher U.S. domestic MAP infection rates than previously reported (10).

MAP is shed from infected animals in feces, and infection transmission between and among animals is similar to other fecal-orally-transmitted diseases. There is an age-dependent susceptibility to MAP infection, and most new infections happen early in the animal's life. Infection of newborns likely happens through transmission *in utero* (before birth), by contaminated colostrum or milk from fecal-contaminated teats or a MAP-contaminated birthing environment.

Infected animals are the only source of MAP in nature, since the pathogen does not generally replicate outside of mammalian cells. When found in soil or water samples, one can assume that the environment was contaminated by MAP in feces deposited by an infected animal (11). MAP shed by infected animals can persist in the environment for years, and soil is another possible source of new animal infec-

tions, although more research is needed to clarify the role of MAP-contaminated water and soil on animal infection rates. The second source of infection, one more amenable to experimentation, is the transmission of MAP from dam to offspring through nursing from contaminated teats or contaminated milk.

RESEARCH:
The high variability in Johne's disease infection rates in various geographic areas presents an opportunity to learn more about the factors that maintain MAP infection and Johne's disease in cattle herds. Epidemiological studies should be designed to make use of data on infection rates and information regarding hygiene practices.

Symptoms

Johne's disease in cattle is characterized by chronic or intermittent diarrhea, weight loss, decline in milk production, and, eventually, death. Animals usually become infected with MAP prior to six months of age, but the signs of Johne's disease are not likely to be observed until two or more years after the initial infection. This extended delay between infection and onset of illness makes the disease difficult to manage in cattle herds, since infected non-symptomatic cattle can contaminate the livestock areas and spread the infection to other animals.

Diagnosis

There are several types of diagnostic tests available for Johne's disease; some detect the presence of MAP-specific antibody (serology), some depend on identification of the organism itself (fecal, tissue or environmental sample culture; PCR to detect MAP-specific nucleic acids) (12). Additional support of a positive Johne's disease diagnosis can be obtained from assessing the clinical condition of the animal and from microscopically examining tissue samples for characteristic pathologic lesions and/or the presence of the organism itself, by culture or PCR.

In general, accuracy of diagnosis is affected by stage of the infection, host species, and MAP strain differences (6). Laboratory diagnostic tests for Johne's disease have high specificity (low false-positive results), but low sensitivity (percentage of MAP-infected animals that test positive). ELISA, a commonly used immunological technique to detect MAP-specific antibodies in blood, detects only about 30% of infected animals when they are still in the preclinical disease phase. In the very early stages of infection, before animals begin to shed MAP in feces or develop an immune response to infection, all animals usually test negative. As the infection progresses and clinical symptoms appear, most of the tests become positive. Diagnostics work best to confirm MAP infection in animals with clinical signs of disease, i.e., diarrhea and/or weight loss.

In the absence of sensitive and accurate diagnostics for early infection, animals are culled when they begin to exhibit decreased milk production, a practice that may result in unnecessary destruction of cattle and economic losses. The lack of tools for early diagnosis also cripples the ability to interrupt the chain of MAP transmission, critical for limiting loss of livestock and, if MAP is a human pathogen, for limiting human exposure.

Control Measures

A number of different control measures are available to limit spread of MAP within herds of cattle and other livestock. Animals become infected with MAP by ingesting the organism through contaminated feed or water. Young animals can

acquire MAP by consuming tainted milk or colostrum. Newborns may become infected *in utero*, but the significance of this route of infection has been difficult to ascertain.

Most dairy cattle herds have frequent turnover (35-40% per year), so preventing the introduction of new animals with uncertain MAP credentials to an uninfected herd is critical. Control measures for MAP infection include (13):

- Segregating birthing facilities from the general herd premises and maintaining good hygiene,
- Bottle-feeding colostrum from test-negative cows,
- Using milk replacer (pasteurized) instead of herd milk (raw),
- Diagnostic test surveillance for the infection and culling test-positive animals,
- Certifying MAP-free herds to provide sources of her replacement, and
- Vaccinating against MAP.

Although anti-MAP vaccines attenuate severity of Johne's disease in domestic cattle, sheep, goats and deer, and may reduce MAP shedding by up to 90%, vaccination does not protect against all new cases of infection by MAP (7). It may, paradoxically, facilitate transmission of MAP within a herd and to other herds, by diminished culling of infected animals. Until otherwise certified as MAP-free, MAP-vaccinated animals should be regarded as potentially infected with MAP.

There is evidence that the physical control measures described above can reduce the prevalence of Johne's disease (14), but regional disparities in effectiveness do exist. This may be because most control programs are voluntary, in part due to the costs of replacing culled animals. Considering this, and the current limitations in testing and control measures, eradication of MAP and Johne's disease does not appear to be a practical target.

Unless MAP is demonstrated to cause disease in humans, however, there is no immediate imperative for widespread mandatory cattle culling. If MAP is not a pathogen of humans, but only animals, then economic considerations, the effectiveness of other transmission control measures, and local policy considerations will help inform the need for culling infected animals.

RESEARCH:

A number of methodological and technical improvements are needed to refine existing veterinary diagnostic tests for Johne's disease and to develop new molecular and antigenic targets using MAP gene sequences or other approaches to improve speed, accuracy, and sensitivity of diagnosis in the earliest stages of infection. These improvements will facilitate cattle and other domestic animal disease management, interrupt the MAP transmission cycle, and stem the financial losses due to culling that the dairy industry currently experiences. If MAP is a human pathogen, then a better diagnostic for early-stage Johne's disease will help to reduce MAP in the food supply and environment and may help prevent development of human disease.

MAP IN THE FOOD SUPPLY

Cattle are the source of milk on the shelves of our supermarkets and the source of beef in our freezers. Cattle herds in the U.S. that are plagued with MAP infections and Johne's disease in increasing numbers should be a public health concern until the controversy over MAP as an etiologic agent of human disease is resolved.

Prevalence

A recent study using culture-dependent methods detected viable MAP in 2.8% of homogenized milk cartons sampled from supermarket shelves in the U.S. Molecular detection methods, which detect the presence of MAP nucleic acids, detected MAP in 64% of samples (15). Other studies detected MAP in samples of soft cheese (16, 17). There is some evidence that MAP can be found in meat, but research in this area is scanty (18, 19). Overall, different levels of MAP contamination of milk and food products have been noted in the U.S. and a variety of countries around the world. MAP has also been identified in other environmental sources in addition to food, including river water and municipal water (20).

Prevention of MAP in the food chain could be assured by only sourcing raw products from animal herds free of MAP. A formal regulatory policy for mandatory screening for MAP in consumer foods should only be considered, however, when focused research studies determine the actual risks of human exposure from this source. Knowing the incidence of MAP in the food chain is of no use unless we also have a detailed understanding of the infectious MAP dose from these sources and an understanding of the consequences of infection.

Control Measures

Standard HTST pasteurization reduces the number of culturable MAP cells in milk products, but does not eradicate MAP (21, 22). "Light pasteurization" (thermization), a process that is less rigorous than regular pasteurization, permits development of higher densities of MAP cells. Successful methods for reducing MAP in milk may include:

- Ultra-high temperature pasteurization (UHT milk),
- UV irradiation,
- Dehydration followed by rehydration,
- Freezing before pasteurization,
- Gamma irradiation,
- Centrifugation, and
- Mycobacteriophages (viruses that infect and kill mycobacteria).

RESEARCH:

Better methods to determine the true prevalence of MAP in foods are needed, as well as methods to determine whether MAP in food is viable and infectious, the infectious dose for animals, if not humans, and the dose that can cause disease in animals and in humans. More research is needed to determine which processes, if any, are effective at killing MAP in food products. More research is also needed to identify whether MAP can be carried away from MAP-contaminated farms and pastures to potable water supplies via surface water and dust, and whether these MAP are sources of infection and disease in animals or humans.

Public Health Concerns

Scientists and public health authorities do not know whether meat and milk products contaminated with MAP expose the public to any risk of illness. When seen on a global scale, the potential implications of such a scenario are staggering. Responsibility for managing exposure and risk from MAP in consumer goods in the U.S., in this case, would fall to the Department of Agriculture, the Food and Drug Administration, the Centers for Disease Control and Prevention, and other federal agencies.

The question of whether MAP-infected animals are fit for human consumption depends on whether MAP is a zoonotic pathogen. Although there is evidence for public exposure to MAP in consumer goods, the biological significance of MAP presence in milk, cheese, and meat is not known, and it is also not known whether eating these products leads to MAP infection, or whether MAP infection necessarily leads to disease.

The appropriateness of taking precautionary measures in the face of a plausible but uncertain risk to human health by exposure to MAP in food sources is a hotly contested debate. What is the level of evidence that must be met in order to take action to limit human exposure to MAP?

In the face of current evidence, at least minimal actions, like removing clinically diseased animals from the food chain, are considered appropriate.

MAP AS A HUMAN PATHOGEN

At the heart of the controversy of MAP as an etiologic agent of CD is whether MAP can initiate human infection at all. MAP can be detected in the human body, and researchers and clinicians have published this fact in a number of case reports in the scientific literature. Moreover, persons with CD are seven-fold more likely than the general population to have MAP associated with their disease (23). However, there has been no definitive causal relationship established between MAP and a specific disease process in humans similar to that which has been established in animals (Johne's disease).

The current methods for isolating MAP from human intestinal tissue for authoritative identification are invasive and are generally obtained from individuals suffering the later stages of CD. The relationship between MAP and the initial stages of CD cannot be extrapolated from testing such late disease-stage tissue samples, and the role of MAP in the initiation of the infection is unclear. The question that needs to be answered is whether MAP is:

- A "professional" human pathogen (pathogenic to otherwise healthy individuals),

- A co-conspirator with other pathogenic bacteria (either one or both partners pathogenic only in the presence of the other, similar to the "enhancement effect" seen in certain virus pairs),

- An opportunistic pathogen (a pathogen in only diseased, genetically-susceptible or immune-compromised individuals), or

- A harmless commensal organism that is simply a bystander, with no role in any human disease process.

MAP is unequivocally pathogenic in animals and may also be pathogenic in humans, but until sensitive and specific diagnostics are discovered and distributed for widespread use, the controversy over MAP and its role in any disease, including CD, will continue.

It is possible that MAP plays a role not only in CD and other gastrointestinal dysfunctions, but also in a variety of other diseases. MAP has been detected in tissues outside the gastrointestinal tract, in a patient with HIV, in lymph tissue, and in breast milk from a lactating mother.

Although the numbers of MAP organisms isolated from any tissue, gastrointestinal or other, has been invariably small, there is precedence for paucibacillary (exhibiting few bacteria) disease caused by other Mycobacteria. Tuberculoid leprosy, caused by *M. leprae*, is a very good example of a mycobacterial disease in which extensive tissue pathology, immunologic in nature, is observed in the absence (or sparse presence) of the inciting bacteria. Since MAP is not widely recognized as a pathogen, physicians do not consider MAP during differential diagnosis, and pathological tissues are seldom tested for the presence of MAP. Compounding this problem is the absence of non-invasive and specific diagnostics available to test for MAP in humans, even if it were suspected.

The noted differences between bovine MAP strains and human MAP strains pose another barrier to our understanding the possible role of MAP in human disease. If MAP is transmitted from infected livestock to humans through the consumption of contaminated food, then testing for the presence of bovine MAP strains in humans could be useful for determining the epidemiology of MAP disease. However, species-specific strain identification is infrequently performed, so it is not known whether ovine strains can lead to human illness.

RESEARCH:
Efficient and effective clinical methods for sub-speciation of MAP into ruminant-specific strains would enable the identification of sources of human MAP and assist in the epidemiology of MAP infection.

Prevalence

The limited number of studies looking for MAP in CD tissue revealed the prevalence of MAP to be between 2% and 20% in various control groups, as well 25-35% in persons with CD (www.cdc.gov). The prevalence of MAP in the general population is currently unknown because the main method for isolation of MAP is intestinal tissue biopsy, and healthy people are not routinely subjected to this procedure. Prevalence and incidence of MAP infection in the general population

awaits the identification and development of specific and sensitive non-invasive diagnostics for this organism.

Infections with MAP must be distinguished from infections caused by other agents, since identifying the correct etiologic agent is the basis for understanding the epidemiology of the disease and its public health implications.

RESEARCH:
Non-invasive screening diagnostics with high specificity for MAP will need to be developed for epidemiology studies in humans to assess incidence and prevalence of MAP infections and determine the public health importance of these infections.

Pathologies

For every condition MAP causes in animals, there may also be a parallel condition in humans. Moreover, it is possible that MAP can cause disease similar to that caused by its close mycobacterial family members or distant cousins, although evidence for MAP infections of other organs (i.e., lungs) is certainly more limited than for CD. MAP has been implicated in scrofula, ulcerative colitis, sarcoidosis, and type 1 diabetes, and there are also isolated reports of MAP disseminating outside the gastrointestinal tract, causing bacteremia (24).

Diagnosis

Individual research laboratories have developed a number of different assays and techniques for diagnosing MAP disease in animals and humans, including bacterial culture, PCR techniques to identify cultured bacteria or to identify MAP DNA in tissues, immunological techniques (cell proliferation and serologic assay), and histopathological examination of tissue using special stains. Samples for diagnosing MAP infection include blood cells or serum, stool, breast milk, biopsies of bowel tissue, and tissue from bowel resection. Culture, stain, or DNA assays are most frequently performed on tissue samples, generally from patients with late-stage disease, and the ability to do these tests on other samples, such as blood, are limited to just a few research labs (24, 25). Immunologic assays have not been sufficiently sensitive for diagnosis of early Johne's disease, where MAP is the definitive causative agent, so it is not a surprise that serologic studies to identify MAP infection of healthy humans or patients with gastrointestinal or other disorders are not demonstrating differences in these populations.

RESEARCH:
Scientists and clinicians in different labs should be encouraged to collaborate to establish standardized, reproducible assays, to make inter-laboratory comparisons, and to clarify the possible connection between the presence of MAP in tissues and disease.

From a regulatory and commercial perspective, there are no approved tests for diagnosing MAP infection in humans. GenProbe's Amplified™ MTD test is often used for "diagnosing" MAP, but this product is not specific for MAP and will also be positive for other members of the broad Mycobacteria family. Unfortunately, the most definitive research techniques for diagnosing MAP infection are not widely used in clinical laboratories, so isolation and identification of MAP is spotty across a wide distribution of interested parties.

Prophylaxis

Despite decades of research, there are no universally effective vaccines against other Mycobacteria sp., such as *M. tuberculosis* (TB) or *M. leprae* (leprosy). Like these well-known pathogens, it may be possible to develop a MAP vaccine using post-genomic techniques, but so far these efforts have not been fruitful for any of the Mycobacteria. *M. tuberculosis*, *M. leprae*, *M. avium*, and MAP (26) genomes

have been sequenced, and comparative analysis might provide direction for MAP vaccine design. Until MAP is decisively identified as a human pathogen, however, vaccine developers are likely to direct their efforts elsewhere.

ANIMAL MODELS OF MAP INFECTION IN HUMANS

If MAP is a human pathogen and causes human disease, it is one of the few in which Koch's postulates were satisfied before the associated disease in humans was described. MAP can be isolated from ruminants with Johne's disease, identified genetically as MAP, and can be re-infected into the same ruminant class (or different ruminants) to cause the same disease. MAP unequivocally causes Johne's disease.

If any human gastrointestinal syndrome is equivalent to Johne's disease, then ruminants could help in the identification of diagnostics and drugs for MAP. However, the link between any gastrointestinal disease and Johne's disease is still highly contentious, and the pathologies of these diseases and Johne's disease, while sharing many characteristics, also have certain characteristics that are distinct. Veterinary experience with Johne's disease identified potential ways to diagnose of human MAP infections, but none of these tests have yet been validated, and they are not routinely available in clinical laboratories. Since it is impractical to treat MAP infections in animals, no guidance for treatment of human MAP infections is found in veterinary literature.

RESEARCH:
Once MAP is definitively identified as a human pathogen, the research community must develop, characterize, and standardize appropriate animal models that can address critical research questions about protective anti-MAP immunological processes and can demonstrate candidate vaccine efficacy. These animal models will be necessary to power the vaccine development process.

Small animal models of CD are available, but the extent of their relevance to human disease remains uncertain.

Treatment

If MAP is an infectious cause of human disease, there is only one therapeutic regimen that could address cure: antibiotic therapy. The only antimicrobial therapies currently recommended for potential MAP infections use drugs presumed to have activity against MAP (macrolides, rifamycins, clofazimine, and ethambutol), all of which actually have limited potency against MAP. They were selected for clinical evaluation because they worked in a small number of CD patients whose disease was presumed to be initiated by MAP. To date, there are no antibiotics that have been specifically developed for treatment of MAP infections.

As is the case with other mycobacterial diseases (including *M. avium* pneumonias, TB, and leprosy), multi-drug therapy with agents from different antibiotic classes may be necessary to eliminate MAP infections and avoid development of MAP resistance. There is recent evidence that existing anti-inflammatory agents may also affect MAP directly. This possibility should be considered in clinical study design and analysis, since most patients with suspected MAP infections are treated for their inflammatory disorder before (and sometime during) treatment with antibiotics.

RESEARCH:

Suitable *in vitro* techniques or animal models that can be used to accurately predict the effectiveness of prophylactics, diagnostics, and antimicrobials useful in MAP infections in humans will be an important research agenda. MAP can infect and cause disease in primates. Primate research has the potential to contribute substantially to the understanding of pathogenicity of MAP and risk of MAP exposure of humans, as well as development of new anti-MAP prophylaxis and therapies.

Table 2. Clinical similarities between CD and Johne's Disease[a]

FEATURE	CROHN'S DISEASE	PARATUBERCULOSIS
Diarrhea	Yes	Yes
Intermittent diarrhea	Yes	Yes
Abdominal pain	Yes	---[b]
Weight loss	Yes	Yes
Obstruction	Yes	No
Ileac region mass	Yes	No
Blood in stool	Rare	Rare
Vomiting	Yes	No[c]
Quiescent periods	Yes	Yes

[a] Table abstracted from Chiodini, 1989 (31)
[b] Domestic animals generally fail to display chronic pain
[c] Vomiting (regurgitation) is uncommon in ruminants, although they eructate (move ingesta from their stomach into their mouth for repeated mastication, commonly called "chewing their cud")

MAP AS ETIOLOGIC AGENT OF CD

Researchers and physicians debate whether MAP, which causes the gastrointestinal inflammation, diarrhea, and weight loss of Johne's disease in animals, may also be to blame for CD. Since Johne's disease shares a number of similarities with CD, a connection between infected livestock and a disease in humans is conceivable. In fact, a correlation between CD and Johne's disease was first suggested in 1913, nearly 100 years ago. Dalziel (27) described several patients with chronic intestinal enteritis that, although very similar to intestinal TB, was believed to be a new disorder. He compared this new disease to a disease in cattle described first in 1894, now known as paratuberculosis, "...in which the histological characters and naked-eye appearances are as similar as may be to those we have found in man. In many cases the absence of acid-fast bacilli would suggest a clear distinction, but the histological characters are so similar as to justify a proposition that the disease may be the same."

Evidence of MAP as an etiologic agent includes the similarity of CD to Johne's disease caused by MAP (Table 2), the detection of MAP in the bowels, blood, or breast milk of some CD patients (24, 25, 28, 29), the effectiveness of antimicrobial drugs in certain MAP-positive and MAP-negative CD patients (30), and the seven-fold higher incidence of MAP in CD patients compared to the population in general (23).

Since MAP can be found in soil, it is not a mystery how grazing ruminants can be infected. But how are CD patients exposed? MAP has been traced to potable water from streams and rivers adjacent to MAP-infected dairy herds. MAP transmission could also occur by ingestion of contaminated milk or milk products or meat from Johne's-infected animals. Molecular analysis of MAP isolates from humans and animals show remarkable similarity. Exposure to a potential enteropathogenic bacterium through the commercial food supply is a troubling prospect.

Genetic Susceptibility

Susceptibility to CD is multigenetic and can be conferred by any of a number of different genes. A recent investigation (4, 5) compared the gene expression patterns of healthy individuals with those of CD patients and was able to identify nine genes linked to susceptibility to this disease. One follow-up study (3) implicated certain immune pathways as genetically deficient in CD patients and suggested that defects in the immune response to intracellular bacteria in particular may be responsible for CD. The pathways that are associated with increased susceptibility to intracellular pathogens have not, however, been specifically investigated for MAP.

Genetic susceptibility traits and exposure to bacteria, however, are not sufficient to predict the development of CD in an individual. Other non-genetic factors appear to be at work.

RESEARCH:

Research should be targeted to identify novel anti-mycobacterials that act specifically and synergistically against MAP. This will require better methods to culture MAP, identification of drug targets that are specific for MAP, development of high throughput screens to evaluate existing drug compound libraries to establish structure-activity relationships, and creation of new chemical entities that are safe and effective for MAP disease.

Figure 2. Farm to Fork transmission of MAP: possible route of human exposure

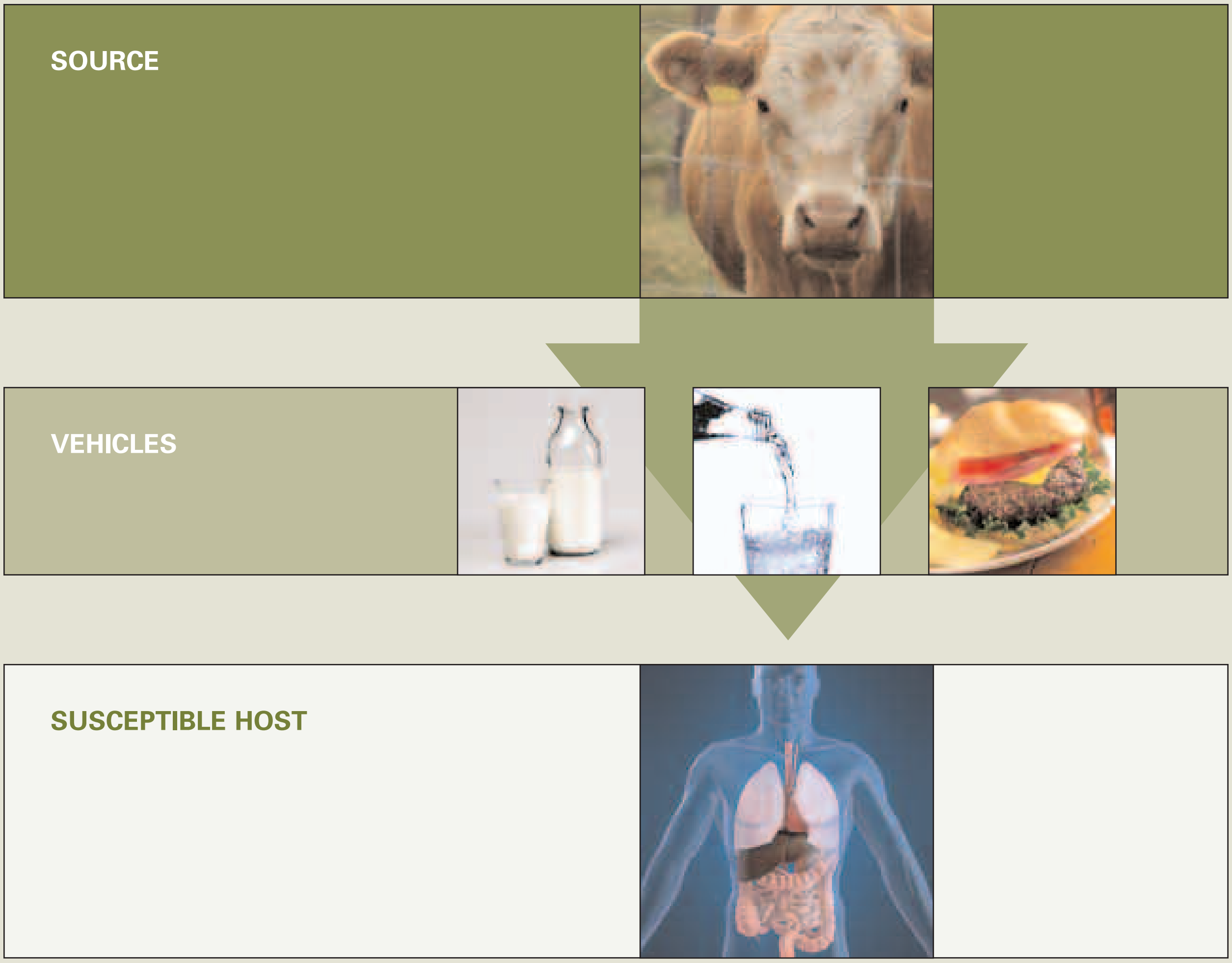

Diagnosis

The principle hurdle in diagnosis of CD is simply recognizing the possibility that CD is in the differential, since disease symptoms vary from patient to patient, and CD symptoms are difficult to distinguish from other gastrointestinal disorders, such as Inflammatory Bowel Disease (IBD). Diagnosis of CD is currently made by combining clinical observations with radiologic, endoscopic, and histological findings. Because of the range of symptoms, CD can be a difficult condition to diagnose with certainty.

CD can be stratified according to age at the onset of symptoms, anatomical location of the diseased tissue, and the behavior of the disease (including inflammatory, structuring, or fistulizing forms).

RESEARCH:

There is an urgent need to find the underlying cause of CD in order to design therapies that can cure this disease. Fully exploring the potential links between CD and MAP is critical to rule in or rule out MAP as a pathogen of humans and as a public health threat transmitted through the commercial food supply.

The prevalence of CD varies from nation to nation and often varies even among the various ethnic groups within a country. The annual incidence of CD is ~6-10/100,000 and the prevalence of CD is about 20 times the incidence, or approximately 150/100,000 people in the U.S. (www.cdc.gov). Prevalence is roughly 250/100,000 in the Canadian province of Manitoba. CD appears to be increasing among Asian populations, and researchers note that individuals of Native American heritage experience lower rates of CD than other ethnic groups.

RESEARCH:

Investigation of the genes responsible for CD susceptibility could enable scientists to understand the underlying host issues in development of CD, and could generate further research into therapies that address a specific genetic defect, resulting in individualized therapy and facilitating the current trend for personalized medicine.

Treatment

There are currently no treatments that cure CD. Medical treatment for CD can be divided into treatments to induce remission of clinical symptoms and treatments to maintain remission. Treatment approaches are also different for fistulizing and non-fistulizing CD. The primary goal of CD treatment today is to control the disease by increasing the length and frequency of disease-free remissions. Because the current CD therapies address only the symptoms of disease, at least 80-90% of CD patients have to undergo surgery at least once for their condition during their lifetimes, and 50% of CD patients undergo a second surgery.

Table 1 lists all the current anti-inflammatory agents, biologics, and other treatments for CD, duration of treatment, and wholesale costs for a 30-day supply. Retail costs will be substantially higher, and costs for administering IV infusions are higher yet because hospital or clinic costs must also be incorporated into the final cost. There are many treatments of varying efficacy, and the majority of these therapies treat the symptoms of a runaway inflammatory response, not the etiologic agent of the disease, if there is one.

RESEARCH:

Off-label use of existing approved antibiotics for other conditions may be applicable to CD, and clinical research on these drugs and drug combinations should continue. Research should also, however, be targeted to identify novel antimycobacterials that act specifically against MAP and that, if MAP is discovered to be the (or one) etiologic agent, are efficacious for CD.

Treatments for maintaining remission of CD symptoms include, in order of effectiveness (most effective to least effective): immunomodulatory agents (i.e., azathioprine, 6-mercaptopurine, and methotrexate); anti-tumor necrosis factor-alpha drugs (anti-TNF alpha); and the antibiotics ciprofloxacin and metronidazol. Steroids can rapidly induce remission of CD symptoms, but steroids are rarely able to maintain remission and can be quite toxic if administered for long periods of time. The long-term outcomes that could be achieved using newer approaches and treatments (anti-TNF alpha drugs) will require time to assess, as most of these biologics have been approved only in the last year or so. One major impediment to their widespread use is, however, cost (see Table 1, page 11).

If CD is caused by MAP or any other microorganism, appropriate antibiotic therapy will be critical for its cure. The only antibacterial drugs approved for use in CD in the U.S. were selected because they worked in a small number of patients (30). There are no antibiotics available today that have been developed specifically for treatment of MAP-induced CD. As described in MAP in Human Infections, it is anticipated that combination drug therapy will ultimately be recommended for CD, if MAP is an etiologic agent, to reduce the probability of MAP drug resistance.

Most drugs used for treating CD are associated with significant side effects and toxicities, some life-threatening, and all require long-term monitoring of the patient.

EVIDENCE FOR AND AGAINST A ROLE FOR MAP IN CD

Evidence for a possible link between MAP and CD includes similarity of disease patterns with Johne's disease, immune response data, the results of tissue sample analysis, and the effectiveness of antimycobacterial drugs. Epidemiologic pattern similarities between Johne's disease (known to be caused by MAP) and CD include:

- Triggering event is in early in life.
- Prolonged period of time between trigger and clinical disease (incubation period).
- Clinical disease onset commonly after sexual maturity.
- Both diseases follow a normal distribution pattern for onset.
- Main target organ is the ileum.
- Host response for both is chronic granulomatous inflammation.

Immune Responses to MAP in CD

The results of immunological testing of CD patients are mixed; some CD patients respond specifically to MAP antigens, while others do not (32). Consistent and reproducible immune responses to MAP by patients with CD would be a strong indication that MAP plays a direct role in this condition, but the lack of a consistent immune response would not necessarily mean the lack of an association. For example:

- If MAP causes CD and susceptibility to MAP depends upon diminished and dysfunctional immune responses, then immune cells of a CD patient could be insensitive to MAP. Immune responses to MAP in cattle are only evident at late stages of disease, when the clinical signs and symptoms of disease are overwhelming. So impaired early immune responses to MAP may be the norm.
- Testing of CD patients may be confounded by the use of immunomodulatory therapies to ameliorate symptoms of disease, which would also dampen the development of antigen-reactive T cells or production of antibodies.
- Since CD symptoms vary, it is also possible that not all CD, but only a portion of CD, is caused by MAP and the rest is caused by a different etiologic agent(s). An immunological survey of all CD patients in a geographic region looking for those who had immunologic responses to MAP might demonstrate that only a segment of the CD population had MAP antibodies. This would be expected with multiple etiologies, but could be misinterpreted as lack of

RESEARCH:
Development of MAP-specific immunologic assays will be necessary to distinguish between MAP and other Mycobacteria family members, many of whom are present in soil and water, and many of which confound current screening techniques for such public health hazards as TB in broad populations in the US and around the world. Validation of immunological assays will be essential and could be problematic, since there is no gold standard technique against which a new method can be measured and current human MAP diagnostic tests are not standardized.

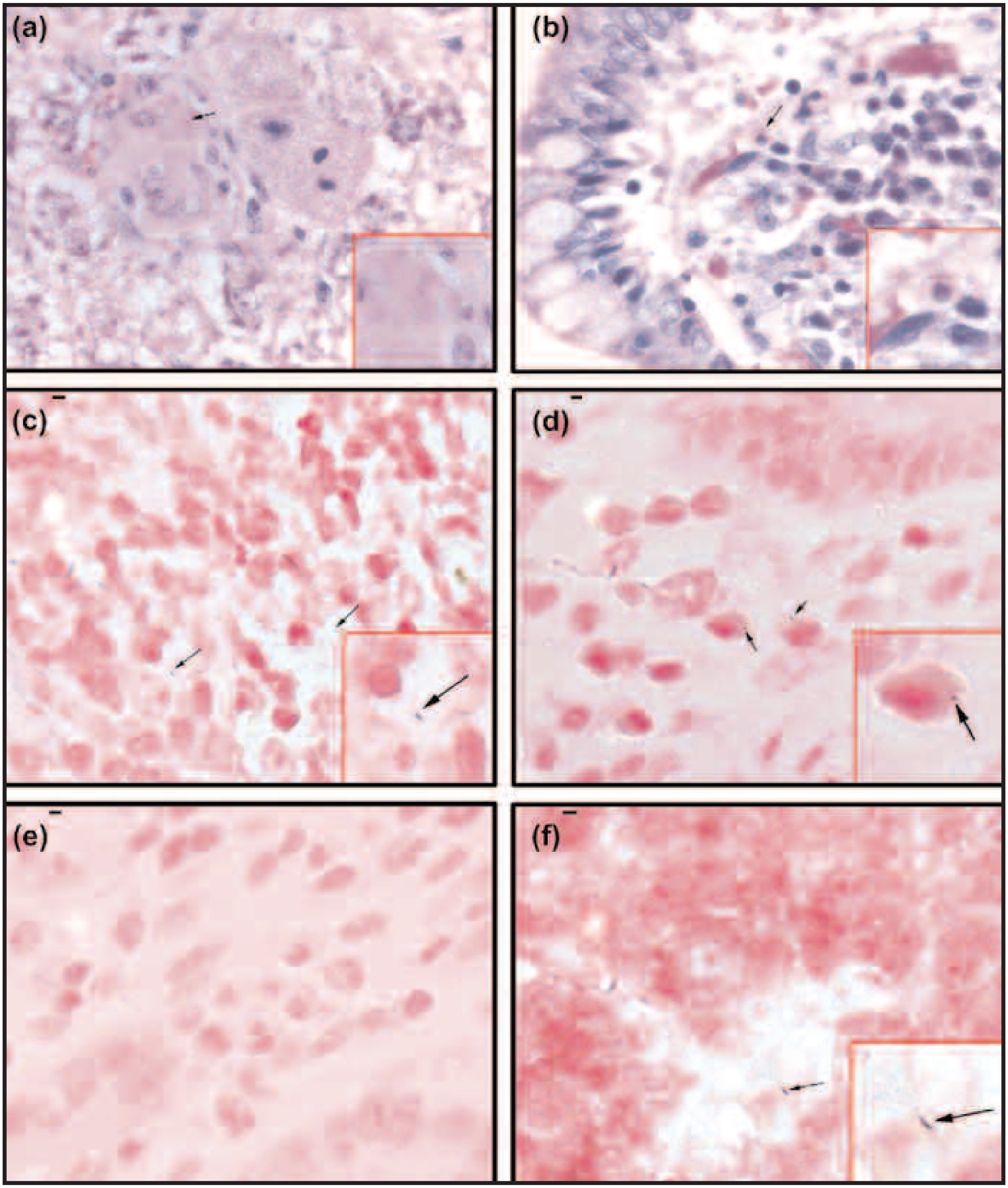

This figure was published in Microbes and Infection, 9, Mangalakumari Jeyanathan, Odette Boutros-Tadros, Jasim Radhi, Makeda Semret, Alain Bitton, Marcel A. Behr, Visualization of Mycobacterium avium in Crohn's tissue by oil-immersion microscopy, page 1572, Copyright Elsvier (2007).

causality if the other agents are unknown. Moreover, antibodies are only an indirect indication of exposure and do not necessarily relate to disease.

- It also follows that, if CD is in part controlled by genetic susceptibility, randomly surveying a population for MAP antibodies could identify healthy individuals who had experienced and controlled MAP infection or (unless the antigen was specific only to MAP) any other Mycobacteria in the past. This would also lead (and has led) to confusion about a MAP etiology of CD.

RESEARCH:
Investigators involved in MAP or CD research must develop and agree upon standards for proficiency testing in molecular methods of MAP detection, so that results from different labs can be compared meaningfully.

Tissue Samples from CD Patients and MAP

Nucleic acid amplification methods (which rely on the presence of DNA or RNA) can detect MAP in diseased tissues obtained from CD patients and patients with ulcerative colitis, but not consistently. These variable results could be due to at least four possibilities: (1) distribution and number of bacteria may vary among individuals experiencing CD symptoms or vary with the specific symptoms (differences in sensitivity to infective doses); (2) people may have different infection rates in different parts of the country (geographical differences); (3) different methods may be used by different investigators, some more sensitive or robust than others (methodological differences); or (4) only a portion of CD is caused by MAP (etiologic differences).

Due to the exacting requirements for growth of MAP for *in vitro* culture, it is difficult to detect MAP by culture-dependent techniques. It is critical to expand the successes achieved in some laboratories to other laboratories involved in determination of CD etiology. Until more consistent cultivation methods are developed, however, molecular methods are the most efficient surrogate for detecting MAP.

ANTIMYCOBACTERIAL DRUGS AND CD

There are reports of the successful treatment of CD, including remission and sustained response, using drugs selected for their antimicrobial activity against *M. avium* organisms. The only reported controlled clinical trial of combination antibiotic treatment in CD, using these drugs, showed a significant short-term advantage of anti-MAP therapy over steroid therapy, but did not show long-term effects in the majority of patients (32).

Prior clinical trials with anti-tuberculous therapies used to combat *M. tuberculosis* infections (the front-line TB drugs rifampin, isoniazid, ethambutol, and pyrazinamide) did not show a benefit for CD patients. These drugs, however, are very specific for *M. tuberculosis,* and even *M. avium* is far less susceptible to any of these drugs than *M. tuberculosis*. So these results were not unexpected.

No studies have systematically tested for the presence of MAP in CD patients before and after treatment with anti-*M. avium* therapies, so it is not known whether these drugs actually reduce the numbers of MAP bacteria in CD patients. The reported success of these drugs in treating CD disease could be due to their effects on MAP, or could be due to other activities of the drugs, or to their effects on other bacteria.

Table 3. Pros and Cons of the Association Between MAP and CD

Facts that SUPPORT the existence of a link between MAP and Crohn's disease:	Facts that DO NOT support the existence of a link between MAP and CD:
■ MAP causes a severe and fatal gastrointestinal disease in ruminants (Johne's Disease) that has clinical and pathological similarities to CD (7).<br><br>■ MAP has been detected in milk, cheese, meat, and water targeted for human consumption, signifying a possible route of exposure to MAP for the general public (15-20).<br><br>■ Genes associated with CD suggest that inappropriate response to an intracellular pathogen may trigger this disease, and MAP is an intracellular pathogen (3, 5).<br><br>■ Increased serological responses to MAP have been detected in CD patients.<br><br>■ MAP has been detected using molecular and histopathological techniques in tissues from CD patients, including blood (24, 25).<br><br>■ MAP has been observed in tissues of CD patients by Ziehl-Neelsen staining (35).<br><br>■ MAP has been grown from various tissues and fluids taken from CD patients (24, 25).<br><br>■ One clinical study identified a significant and prolonged response to antimicrobial therapy in patients with CD, another study demonstrated a short-term benefit to antimicrobial therapy (30).<br><br>■ CD patients have a seven-fold more likely chance of having MAP in their tissues than the general population (23).	■ Dairy farmers and others who may have greater exposure to MAP than the general population did not experience higher rates of CD in one study (33).<br><br>■ There are some dissimilarities in the clinical and pathological presentation of CD and Johne's disease (31).<br><br>■ Cell-mediated immune responses to MAP or MAP antigens have not been demonstrated in CD patients.<br><br>■ There have been no systematic studies, but CD has not yet been reported to worsen with progressive immunocompromise, such as happens with *M. tuberculosis* infections (exacerbated by HIV or anti-TNF therapy) (34).<br><br>■ In a controlled clinical trial, CD patients undergoing six months of antibiotic therapy did not maintain a sustained response to the drugs, and the relapse rate after two years was similar in treated and control groups (30).

Considering reports of success in using antibiotic therapy to treat CD in some patients, it is perplexing that more physicians aren't actively pursuing antimicrobial drug therapies for their CD patients and more research is not happening to discover new and better (more specific) drugs to treat this disease. It is likely that the current lack of evidence-based studies on the MAP causality of CD is the basis for this reluctance. On the positive side of antibiotic therapy, most antibacterial drugs are generally well tolerated compared to the anti-inflammatory drugs used to suppress CD symptoms. Side effects are well characterized through trials for other infections and are not notably different in CD patients. On the other hand, use of antibiotics for a disease without a known infectious etiology could induce drug resistance in MAP or other bacteria, adding more antimicrobial resistance genes to the pool of genes circulating in the population. Despite these reservations, physicians are encouraged to continue helping CD patients with whatever tools they deem necessary.

There has been no systematic and widespread effort to explore possible links between exposure to MAP bacteria (in food or elsewhere) and the subsequent development of CD disease. In the U.S., CD is not reported to public health agencies, so public documents on the actual prevalence of CD over time are extrapolated from academic and clinical publications in various populations. There is both insufficient data on the occurrence of MAP in the environment and an absolute requirement for a widely available, sensitive, and specific human MAP diagnostic(s) to undertake an epidemiological study of sufficient scope to discover a possible link.

More research support and substantial additional research effort by both scientists and clinicians is necessary before we will know whether CD has an infectious etiology, and whether MAP is the culprit. It is possible that a subset of CD patients have MAP disease; future research must be directed toward establishing whether there is a strong and reproducible relationship between MAP and CD.

RESEARCH:

Despite the difficult nature of MAP culture, attempts to develop consistent and reproducible methods to isolate and culture MAP from tissues of CD patients continues to be an important research activity, the results of which will eventually inform many of the outstanding questions regarding the diagnosis and treatment of CD.

RECOMMENDED RESEARCH TO CLARIFY MAP AS A HUMAN PATHOGEN

The prospect that MAP could play a role in the incitement or development of CD is a sobering one, and once the situation becomes clearer, there could be important changes in store for agriculture, food safety, and public health. It is in the best interest of the public that the possible connection between CD and MAP be explored exhaustively.

Colloquium participants strongly urge scientists to carry out rigorous, exacting research and epidemiological studies on MAP and CD and to develop reproducible diagnostics for MAP in order to provide the data needed to achieve consensus on this matter in the scientific and regulatory communities.

RESEARCH PRIORITIES

There are no easy answers to the problem of identifying the role of MAP in CD or other human infections; no single "home run" study will fill the many gaps in the collective scientific knowledge. Instead, researchers must tackle the problem from multiple directions.

The two broad areas of uncertainty are:

- The transmissibility of MAP from food and the environment to humans, and

- The susceptibility of the human body to MAP infection and disease.

To clear up these uncertainties, a strong international research collaboration must be forged with a variety of stakeholders, all with common interests in solving the puzzle—microbiologists and mycobacteriologists, veterinarians, food microbiologists and regulators, epidemiologists, research scientists, product developers and regulators, domestic food-source animal industries, public health officials, clinicians, gastroenterologists, and patient populations. If MAP is associated with human disease and is in the U.S. food supply, this will be a public health concern to rival that of TB in the early part of the 20th century.

RESEARCH ISSUES

Transmissibility of MAP to Humans

MAP exists in Johne's-infected livestock, in livestock waste, on farms, and possibly in the food and water supplies (see MAP in the Food Supply, above), but it is far from clear whether humans can become infected with MAP upon exposure to these sources. And if they become infected with MAP, do humans develop disease?

Of primary importance to the issue of MAP transmissibility is whether or not the MAP strains isolated from humans can be traced back to MAP strains isolated from animals. Are they related genetically? To determine this will require development of robust and highly specific MAP speciation techniques and the ability to

isolate MAP from human tissues. Genetic similarities and differences may also identify potential virulence factors in human MAP isolates.

Direct assessment of transmissibility will be difficult. A potential source of MAP exposure for humans is the consumption of contaminated milk from Johne's disease-infected cows, but it is unethical to conduct a prospective study to clarify this possibility (i.e., humans fed deliberately contaminated milk). Retrospective studies, which require subjects to think back on past exposure to milk, are not particularly illuminating, since subjects must recall when and how much milk was consumed, and researchers are unable to confirm whether or not the milk drunk in the past was contaminated at the time of consumption. The best direct answer to MAP transmission by contaminated milk may be to employ an animal model. Johne's disease in goats, whose pathophysiologic symptoms are the most similar of the ruminants to human CD, could be developed into a surrogate animal model for this purpose. The incidence of Johne's disease in groups of animals fed contaminated or MAP-free milk (or other foods or water) could then be compared, and the dose of MAP that is infective by this route could be established. Moreover, having a well-characterized animal model would allow researchers to determine the relative infectivity and pathogenicity of MAP strains isolated from humans.

MAP transmissibility to humans from environmental sources, particularly from MAP-infected livestock, could possibly be uncovered and evaluated by epidemiologic studies that look at populations living in geographical regions with a low rate of reported Johne's disease, including Sweden, Iceland, and the eastern regions of Australia, compared to geographic regions with a high rate of Johne's disease. For these studies to occur, sensitive and specific MAP diagnostics must first be developed and standardized, and for these studies to be informative, simultaneous (or contemporaneous) MAP surveys of cattle and other ruminants in the area must also be performed. MAP contamination of milk and water from the specific region will also be important to assess. If MAP infection rates are absent or significantly lower in low Johne's disease regions, and are high in high Johne's disease regions, then a strong inference could be made that humans contract MAP (directly or indirectly) from infected livestock. However, this will be complicated by the extensive regional and international trade of foods, making it challenging to assure that the foods consumed originated from the place where the CD patients live.

If it is established that humans exposed to MAP in their environments become infected (i.e., diagnostics detect MAP), all the stakeholders will need to know the health consequences of exposure in order to gauge the possible risks of disease from MAP. These studies will not be easy, because all measurements will be an indirect indication (or surrogate marker) of infection or disease—symptoms or immunological reactions to MAP. In some instances, new animal models may be needed to address questions of MAP pathogenesis and therapy.

Human Immune Responses to MAP

One way to assess exposure (but not necessarily infection or disease) is to determine human MAP immunological reactions in the general population or in specific disease populations like CD. Onset of immune responses to most Mycobacteria, especially the intracellular pathogens, takes months. Because of their intracellular habitat, these pathogens are nearly impossible to eradicate, although they can be immunologically contained. The immune response accelerates and expands to include all manner of reactions and immune factors over time, and tissue destruction in both TB and leprosy is largely due to the constant antigenic challenge of immune cells and resulting immunologic chaos.

To understand the interaction of human immune system following exposure to MAP, it would be helpful to first have an indication of the timing, type, and duration of inflammatory and immune events in an animal model that is undergoing disease reasonably similar to CD. That would at least narrow down the types of immune responses that should be investigated with priority in humans.

A basic understanding of the ongoing MAP-specific cellular and humoral immune interactions that constitute a response to MAP in humans could facilitate identification of specific immunological responses that occur in CD patients versus healthy individuals, or that occur in different stages or types of CD. Of course, it will not be useful to survey all CD patients for MAP responses if MAP causes only a portion of CD cases; in that event, sensitive and specific human diagnostics will need to be identified, developed, and standardized so that patients can be segregated in to MAP-infected CD and non-MAP CD. It may then be possible to identify MAP-specific human immune reactions that can be developed into additional diagnostics to detect early symptoms of relapse or verify cure. It may also be possible to correlate human MAP-specific immune reactions to bacillary load in the infected tissues or to other markers of disease.

What if MAP is not the sole agent of CD? What if the relevant aspect of MAP infection is its ability to modulate human immune response(s) to other pathogens? Or to divert the proper immune reaction for a different bacterium to an ineffective one, thus allowing the second pathogen to gain a foothold in tissues that it is not normally allowed to penetrate? Some understanding of the "typical" human immune reaction to MAP may enable scientists to recreate the more complicated CD induction scenario (more than one pathogen necessary to create disease) in appropriate animal models of CD.

There are reports that a percentage of healthy humans are positive for MAP serology. If screening diagnostic tests are highly specific for MAP, these healthy MAP-exposed individuals could provide a window into the "normal" MAP response. Research including these subjects could provide information on immuno-dominant MAP antigens, as well as a comparison with immune responses observed in CD patients.

At the moment, and unfortunately, human MAP infection is most obvious and easiest (although not easy) to identify in CD patients with symptoms of disease. These patients are generally being treated with powerful anti-inflammatory agents or immune suppressive therapies. There may be no way to assess a "typical" anti-MAP immune response in CD patients unless clinicians are able to identify MAP-infected persons when they first seek medical care and are not yet on therapeutics that suppress immune cells. Good MAP diagnostics will be essential. If diagnostics are not yet available, good animal models that mimic CD could be helpful, with the caveat that immune systems and immune reactivity differ between species. With an animal model, however, MAP can ethically be administered by itself or co-administered with other pathogens to determine outcomes.

Therapeutics

The antimicrobial agents available to treat MAP infections or CD were not developed specifically to treat MAP and appear inadequate to provide significant long-term results for patients. Clinical research to establish effective therapeutic regimens with existing antibiotics is encouraged. Further research should also be directed toward identifying new and effective antimicrobials for treating MAP infection.

Other Research Priorities

The National Research Council has made recommendations for research examining possible links between CD and MAP (2003). Recommendations 17 through 25 in the report are particularly compelling and require follow-up. In addition, two new reports will be released by agencies in the U.S. Federal Government in 2008, and these reports may contain additional Research Recommendations:

- Development of an Action Plan to Address Surveillance, Epidemiologic, Laboratory and Environmental Issues Related to Disease Caused by Nontuberculous Mycobacteria. Centers for Disease Control and Prevention External Consultation. May 2007 – April 2008.

- National Advisory Committee on Microbiological Criteria for Foods. Assessment of Food as a Source of Exposure to Mycobacterium avium subspecies paratuberculosis (MAP). FINAL DRAFT REPORT. September 24, 2007

Development of Reproducible Analytical Techniques for MAP

There is a conspicuous and fundamental barrier to accomplishing the research on MAP as an etiologic agent of CD: the lack of a reproducible, sensitive, and specific diagnostic(s) for MAP. New tools to identify and isolate MAP are critical to every recommended activity outlined in this document, and without them researchers cannot move forward. Public health laboratories and the U.S. Centers for Disease Control and Prevention laboratories have made it clear that they cannot grow MAP, which hinders diagnosis and reporting. Research would also benefit from more sensitive methods for identifying MAP when it is isolated, and for speciating MAP isolates from infected animals and humans.

A gold-standard test for MAP that researchers and clinicians alike can turn to may stratify testing results, clearly separating individuals with MAP infections from uninfected CD (or other) patients, and thus clarify the role of MAP in CD. Research that uses unreliable, inconsistent technologies for detecting MAP will always be open to criticism and negative scrutiny.

Inter-laboratory collaborations to establish reproducible, standardized techniques for detecting MAP are strongly encouraged. Research in this area often requires the comparison of assay results from different laboratories. Therefore, standardized techniques that produce the same results regardless of the service provider are critical.

Once better methods have been developed, a network of proficiency testing for laboratories that offer MAP diagnostics should be established. A strong, unbiased, external laboratory proficiency evaluation program or quality assessment program is critical to establishing and maintaining confidence in MAP research and clinical testing.

A task force should be established to move forward and develop more specific recommendations for developing improved methods for MAP detection.

Eventually, interested parties will be forced to address the problem of which specimen types are most appropriate for testing for MAP infections in humans. Blood, intestinal mucosal tissue, stool, and colonic exudate are all possibilities for testing purposes. Better assays are also needed for detecting MAP in environmental (water and soil) samples, as well as food.

A Note About Resolving Causality

There are certain details to consider in attempting to find a causal link between MAP and CD. For example, if MAP is associated with CD-affected tissues, the possibility that MAP is an opportunist taking advantage of inflammation caused by another organism must be considered and investigated. In this scenario, anti-MAP therapies would alleviate the secondary MAP infection, but not the underlying inflammation due to a different pathogen, as yet identified.

Another imperative in defining causality is the use of appropriate controls. Whenever researchers test tissue for the presence of MAP, they should also check for the presence of other organisms in order to bolster confidence in the results.

CD is likely to be a multifactorial disease, created by the confluence of many different phenomena, and the dividing line between CD and other similar diseases, like ulcerative colitis and IBD, is sometimes difficult to discern. Researchers need to clearly define the conditions of study participants in order to make the conclusions of the work as unambiguous as possible.

RECOMMENDATIONS

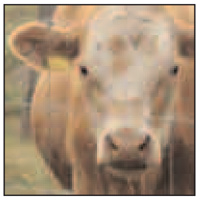

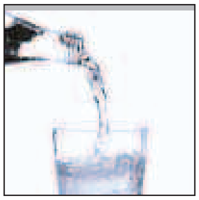

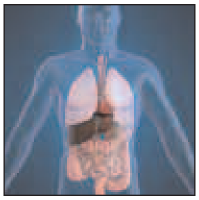

- Research to discover and standardize diagnostics that are both sensitive and specific for MAP in animals and in humans and can determine the source of MAP cultured from human tissue is imperative. Virtually all the research topics that will clarify the role of MAP in CD rely on this.
- Research must address the issue of MAP transmissibility and determine whether or not the MAP strains isolated from animals or food are genetically identical to the MAP strains isolated from humans.
- Research should also examine the potential virulence factors in human MAP isolates and determine whether MAP can be transmitted from human to human.
- Researchers must develop better animal models for evaluating MAP effects on human hosts and for evaluating the effectiveness of potential therapies for MAP infection.
- A policy for regularly screening foods for MAP should not be put into place until focused research studies can determine the actual risks of exposure and disease.
- Identifying novel MAP-specific antimicrobials and effective antibiotic treatment regimens for MAP infections is a research priority.

A Word on the Precautionary Principle

The Precautionary Principle (http://en.wikipedia.org) as a formal concept evolved from German social and legal tradition in the early 20th century. It is underpinned by common sense aphorisms that pre-date the term, such as "an ounce of prevention is worth a pound of cure," "better safe than sorry," and "look before you leap." The Precautionary Principle is also considered to have evolved from the ancient medical principle, "first, do no harm," as it applies to institutions and institutional decision-making processes, rather than individuals.

The 1998 Wingspread Statement on the Precautionary Principle summarizes the principle this way: "When an activity raises threats of harm to human health or the environment, precautionary measures should be taken, even if some cause and effect relationships are not fully established scientifically" (Science and Environmental Health Network). In deciding how to apply the principle, one uses cost-benefit analysis, assessing both the opportunity cost of not acting and the option value of waiting for further information before acting. In modern policy making, there is often an irreducible conflict between different interests, so the debate is necessarily political. This is no more evident than in the debate about MAP and CD.

When should policy makers evaluate the Precautionary Principle as it relates to the role of MAP in CD? There are indications that MAP is pathogenic for humans, and MAP is found in U.S. cattle that supply food for human consumption and in milk and perhaps other foods on the supermarket shelf. A significant number of U.S. citizens, more than 500,000, are affected by CD.

The colloquium participants were not prepared to recommend as a group that public health authorities move today to mitigate the public's exposure to Johne's disease-infected animals by enacting mandatory agriculture and food safety regulatory policies to eliminate potential routes of exposure to MAP.

It is important, however, for all regulatory bodies concerned with agriculture and public health to consider this report, and the other Federal reports that will be published later this year, in light of the Precautionary Principle. The science and clinical communities will continue to identify gaps in the current knowledge relevant to MAP exposure and, with appropriate funds and collaborations, begin to provide data that will inform these deliberations. But the decision to invoke the Precautionary Principle should be a political debate at the highest levels of our federal institutions charged with guarding the public health, and the timing of this debate should be now.

REFERENCES

1. Crohn, B.B., L. Ginzburg, G.D. Oppenheimer. 1984 Regional ileitis. A pathological and clinical entity. (Landmark article 1932). JAMA 251: 251:73-79.

2. Ashford, D., B. Imhoff, F. Angulo, and the FoodNet working group, CDC, Atlanta, GA. Over a Half-Million Persons May Have Crohn's Disease in the United States. Infectious Diseases Society of America. San Francisco, CA, October 2001. http://www.cdc.gov/enterics/publications/24_ashford_2001.pdf

3. Ferwerda, G., B.J. Kullberg, D.J. deJong, et al. 2007. Mycobacterium paratuberculosis is recognized by Toll-like receptors and NOD2. J. Leukoc Biol 82:1011-1018.

4. Wellcome Trust Case Control Consortium. 2007. Genome-wide association study of 14,000 cases of seven common diseases and 3,000 shared controls. Nature 447:661-678. http://www.nature.com/nature/journal/v447/n7145/pdf/nature05911.pdf

5. Parkes, M., J.C. Barrett, N.J. Prescott, et al. 2007. Sequence variants in the autophagy gene IRGM and multiple other replicating loci contribute to Crohn's disease susceptibility. Nature Gen 37:830-832.

6. Turenne, C.Y., R. Wallace, Jr., and M.A. Behr. 2007. Mycobacterium avium in the post-genomic era. Clin Micro Rev 20:205-229. http://cmr.asm.org/cgi/reprint/20/2/205.pdf

7. Harris, N.B., R.G. Barletta. 2001. Mycobacterium avium subsp. paratuberculosis in Veterinary Medicine. Clin Micro Rev 14:489-512.7. http://cmr.asm.org/cgi/reprint/14/3/489.pdf

8. Chiodini, R.J., H.J. Van Kruiningen, R.S. Merkal. 1984. Ruminant paratuberculosis (Johne's disease): the current status and future prospects. Cornell Vet 74:218-262.

9. Committee on Diagnosis and Control of Johne's Disease, National Research Council. 2003. Diagnosis and control of Johne's disease. The U.S. National Academy of Sciences Board on Agriculture and Natural Resources Report. National Academy Press, Washington, D. http://www.nap.edu/catalog.php?record_id=10625#toc

10. Veterinary Services Report, due 2008, Johne's Disease on U.S. Dairy Farms, 1991-2007. United States Department of Agriculture.

11. Whittington, R.J., D.J. Marshall, P.J. Nichols, et al., 2004. Survival and dormancy of Mycobacterium avium subspecies paratuberculosis in the environment. Appl Environ Microbiol 70:2989-3004. http://aem.asm.org/cgi/reprint/70/5/2989

12. Collins, M.T., I.A. Gardner, F.B. Garry, A.J. Roussel, S.J. Wells. 2006. Consensus recommendations on diagnostic testing for the detection of paratuberculosis in cattle in the United States. J Am Vet Assoc 229:1912-1919.

13. USDA. 2005. Dairy 2002 Johne's disease follow-up study: Management practices and within-herd prevalence. APHIS Info Sheet, Veterinary Services. Centers for Epidemiology and Animal Health. http://www.aphis.usda.gov/vs/ceah/ncahs/nahms/dairy/dairy02/Dairy02_Johnes_followup_study.pdf

14. Goodger, W.J., M.T. Collins, K.V. Nordlund, C. Eisele, J. Pelletier, C.B. Thomas and D.C. Sockett. 1996. Epidemiologic study of on-farm management practices associated with prevalence of Mycobacterium paratuberculosis infections in dairy cattle. J Am Vet Assoc 208, 1877-1881.

15. Corti, S. and R. Stephan. 2002. Detection of Mycobacterium avium subspecies paratuberculosis specific IS900 insertion sequences in bulk-tank milk samples obtained from different regions throughout Switzerland. BMC Microbiol. 2, 15. http://www.biomedcentral.com/content/pdf/1471-2180-2-15.pdf

16. Clark, D.L., J.L. Anderson, J.J. Koiziczkowski and J.L.E. Ellingson. 2006. Detection of Mycobacterium avium subspecies paratuberculosis genetic components in retail cheese curds purchased in Wisconsin and Minnesota by PCR. Mol. Cel. Probes 20, 197-202.

17. Donaghy, J.A., N.L., Totton, and M.T. Rowe. 2004. Persistence of Mycobacterium paratuberculosis during manufacture and ripening of cheddar cheese. Appl Environ Microbiol 70, 4899-4905. http://aem.asm.org/cgi/reprint/70/8/4899.pdf

18. Jaravata, C.V., W.L. Smith, G.J. Rensen, J. Ruzante, and J.S. Cullor. 2007. Survey of ground beef for the detection of Mycobacterium avium paratuberculosis. Foodborne Pathog Dis 4, 103-106.

19. Abubakar, I., D.J. Myhill, A.R. Hart, et al. 2007. A case-control study of drinking water and dairy products in Crohn's disease—further investigation of the possible role of Mycobacterium avium subspecies paratuberculosis. Am J Epidemiol 165:776-783. http://aje.oxfordjournals.org/cgi/reprint/165/7/776

20. Pickup, R.W., G. Rhodes, T.J. Bull, S. Arnott, K. Sidi-Boumedine, M. Hurley and J. Hermon-Taylor. 2006 Mycobacterium avium subsp. paratuberculosis in Lake Catchments, in river water abstracted for domestic use, and in effluent from domestic sewage treatment works: Diverse opportunities for environmental cycling and human exposure. Appl Environ Microbiol 72:4067-4077. http://aem.asm.org/cgi/reprint/72/6/4067

21. Chiodini, R.J. and J. Hermon-Taylor. 1993. The thermal resistance of Mycobacterium paratuberculosis in raw milk under conditions simulating pasteurization. J Vet Diagn Invest 5:629-631. http://jvdi.org/cgi/reprint/5/4/629.pdf

22. Donaghy, J.A., M. Linton, M.F. Patterson, and M.T. Rowe. 2007. Effect of high pressure and pasteurization on Mycobacterium avium subsp. paratuberculosis in milk. Lett Appl Microbiol 45:154-159.

23. Feller, M., K. Huwiler, R. Stephen, et al. 2007. Mycobacterium avium subspecies paratuberculosis and Crohn's disease: a systematic review and meta-analysis. Lancet Infec Dis 7:607-613.

24. Chamberlin, W., G. Ghobrial, M. Chehtane, S.A. Naser. 2007. Successful treatment of a Crohn's disease patient infected with bacteremic Mycobacterium paratuberculosis. Am J Gastroenterol. 102:689-91.

25. Chamberlin, W. and S. A. Naser. 2007. Blood Cultures of 19 Crohn's Disease Patients. Am J Gastroenterol. (In press)

26. Li, L., J.P. Bannantine, Q. Zhang, A. Amosin, B.J. May, D. Alt, N. Banerji, S. Kanjilal and V. Kapur. 2005. The complete genome sequence of Mycobacterium avium subspecies paratuberculosis. PNAS 102:12344-12349. http://www.pnas.org/cgi/reprint/102/35/12344.pdf

27. Dalziel, T.K. 1913. Chronic interstitial enteritis. Br. Med. J. 2:1068-1070. http://www.springerlink.com/content/l094786t467v7478/fulltext.pdf

28. Bull, T.L, E.J. McMinn, K. Sidi-Boumedine, et al. 2003. Detection and verification of Mycobacterium avium subspecies paratuberculosis in fresh ileocolonic mucosal biopsies from individuals with and without Crohn's disease. J Clin Micro 41:2915-2923. http://jcm.asm.org/cgi/reprint/41/7/2915.pdf

29. Autschbach, F., S. Eisold, U. Hinz, et al. 2005. High prevalence of Mycobacterium avium subspecies paratuberculosis IS900 DNA in gut tissues from individuals with Crohn's disease. Gut 54:944-949. http://gut.bmj.com/cgi/content/abstract/54/7/944

30. Selby, W., P. Pavli, B. Crotty, et al. 2007. Two-year combination antibiotic therapy with clarithromycin, rifabutin, and clofazimine for Crohn's disease. Gastroenterol 132:2313-2319.

31. Chiodini RJ. 1989. Crohn's disease and the mycobacterioses: a review and comparison of two disease entities. Clin Microbiol Rev 2: 90-117. http://alan.kennedy.name/crohns/research/diagnos/chiodini.htm

32. Marks, D.J., M. W. Harbord, R. MacAllister, 2006. Defective acute inflammation in Crohn's disease: a clinical investigation. Lancet 367:668-678. http://www.pubmedcentral.nih.gov/articlerender.fcgi?artid=2092405

33. Jones, P.H., T.B. Farver, B. Beaman, et al. 2006.Crohn's disease in people exposed to clinical cases of bovine paratuberculosis. 2006. Epidemiol Infect 134:49-56.

34. Sartor, R.B. 2005. Does Mycobacterium avium subspecies paratuberculosis cause Crohn's disease? Gut 54:896-898. http://gut.bmj.com/cgi/reprint/54/7/896

35. Jeyanathan, M., O. Boutros-Tadros , J. Radhi, M. Semret , A. Bitton, and M.A. Behr. 2007. Visualization of Mycobacterium avium in Crohn's tissue by oil-immersion microscopy. Microb and Infect 9: 1567-1573.
