# Supplementary material for: Long History of Queries about Bovine Paratuberculosis as a Risk Factor for Human Health
Source: Pathogens. 2021 Oct 28;10(11):1394. doi: 10.3390/pathogens10111394 (PMC8622788; doi:10.3390/pathogens10111394)
Supplement: Supplementary file 1 [file pathogens-10-01394-s001.zip › SWEENEY.pdf]

ACVIM Consensus Statement
J Vet Intern Med 2012;26:1239–1250

Consensus Statements of the American College of Veterinary Internal Medicine (ACVIM) provide the veterinary community with up-to-date information on the pathophysiology, diagnosis, and treatment of clinically important animal diseases. The ACVIM Board of Regents oversees selection of relevant topics, identification of panel members with the expertise to draft the statements, and other aspects of assuring the integrity of the process. The statements are derived from evidence-based medicine whenever possible and the panel offers interpretive comments when such evidence is inadequate or contradictory. A draft is prepared by the panel, followed by solicitation of input by the ACVIM membership which may be incorporated into the statement. It is then submitted to the Journal of Veterinary Internal Medicine, where it is edited prior to publication. The authors are solely responsible for the content of the statements.

Paratuberculosis (Johne's Disease) in Cattle and Other Susceptible Species

R.W. Sweeney, M.T. Collins, A.P. Koets, S.M. McGuirk, and A.J. Roussel

Paratuberculosis (Johne's disease) is a widespread and costly disease. This consensus statement will summarize recommendations regarding diagnosis, control, and treatment of Johne's disease in cattle and other species. Each section of recommendations is followed by a statement that subjectively characterizes the strength of the supporting evidence. The role played by *Mycobacterium avium* subsp. *paratuberculosis* (MAP) in the pathogenesis has been a matter of controversy for many years. This statement concludes with an assessment of the evidence in favor of MAP as a potential zoonotic pathogen.

Key words: Johne's disease; mycobacterium; Paratuberculosis; Ruminant.

Johne's disease, the enteric disorder caused by *Mycobacterium avium* subsp. *paratuberculosis* (MAP), was first reported in cattle in Europe in 1895 and in the United States in 1908. Over the ensuing 100+ years, the infection has spread to the point that NAHMS estimates now suggest that 70% of all US dairy herds and 5–10% of US beef herds have infected animals.[1,2]

The clinical signs of Johne's disease in cattle are easily recognized: pipestream diarrhea, weight loss, and edema due to hypoproteinemia caused by protein-losing enteropathy. However, the fastidious growth requirements of MAP in vitro, long in vivo incubation period ("eclipse" phase), and blunted humoral immune response have made diagnosis a challenge in many situations. Less typical clinical signs have added to the challenge of diagnosis in sheep, goats, and camelids. Persistence of MAP in the environment and lack of effective vaccines have complicated on-farm control. Occasionally, treatment of individual animals with clinical Johne's disease is attempted, with varied success. Finally, until recently, the veterinary community has had the luxury of treating MAP as an animal pathogen only, and control has been voluntary. However, a mounting body of evidence suggests a link between MAP and human disease, and some countries have now begun to approach Johne's disease control as a food safety issue.[3]

Abbreviations:

AGID	agar gel immunodiffusion
CRC	controlled release capsule
CVJDPCP	Canadian Voluntary Johne's Disease Prevention and Control Program
DHIA	Dairy Herd Improvement Association
ELISA OD	enzyme-linked immunosorbent assay optical density
ELISA S/P	enzyme-linked immunosorbent assay sample to positive OD ratio
HTST	high-temperature short time (pasteurization)
MAP	*Mycobacterium avium* subsp. *paratuberculosis*
NAHMS	National Animal Health Monitoring System
PCR	polymerase chain reaction
RAMP	risk assessment and management plan
SNP	single nucleotide polymorphism
USVBJDCP	US Voluntary Bovine Johne's Disease Control Program

From the Department of Clinical Studies-New Bolton Center, University of Pennsylvania School of Veterinary Medicine, Kennett Square, PA (Sweeney); the Department of Pathobiological Sciences, School of Veterinary Medicine, University of Wisconsin-Madison, Madison, WI (Collins); the Department of Farm Animal Health, Utrecht University Faculty of Veterinary Medicine, Utrecht, The Netherlands (Koets); the Department of Medical Sciences, School of Veterinary Medicine, University of Wisconsin-Madison, Madison, WI (McGuirk); and the Department of Large Animal Clinical Sciences, College of Veterinary Medicine and Biomedical Sciences, Texas A&M University, College Station, TX (Roussel).

Corresponding author: R.W. Sweeney, Department of Clinical Studies-New Bolton Center, University of Pennsylvania School of Veterinary Medicine, New Bolton Center, 382 W. Street Rd., Kennett Square, PA 19348; e-mail: rsweeney@vet.upenn.edu.

Submitted August 25, 2012; Revised August 25, 2012; Accepted September 6, 2012.

Copyright © 2012 by the American College of Veterinary Internal Medicine

10.1111/j.1939-1676.2012.01019.x

This statement will summarize the ACVIM consensus recommendations regarding diagnosis, control, and treatment of Johne's disease in cattle and other species. Each section of recommendations is followed by a statement, which subjectively characterizes the supporting evidence as "strong" (eg, controlled prospective clinical trials or rigorous experimental studies), "moderate" (eg, retrospective studies, expert consensus), or "weak" (eg, conflicting results, uncontrolled studies). The Consensus Statement concludes with the panel's assessment of MAP as a potential zoonotic pathogen.

Diagnosis of MAP Infection

Available Testing Methods

Bacterial Culture of Fecal Samples. Detection of live MAP in fecal samples has the advantage that a positive result confirms the presence of viable MAP in the sample, and also allows for strain typing for molecular epidemiologic purposes. The sensitivity of culture is estimated to be approximately 60% relative to necropsy, with specificity >99%.[4] Through enumeration of colony-forming units (on solid media), or time to detection (on liquid media), the relative amount of MAP shedding in feces, and thus the transmission risk posed by the animal, can be evaluated. Samples that have positive results at a very low level of MAP should be interpreted with caution, if collected from a cow residing in a heavily MAP-contaminated environment, because the result could represent "pass through" of organisms recently consumed rather than shed from infected intestinal tissue (false-positive culture result).[5,6] Culture can be performed on fecal samples from individual animals, on pooled samples, or on samples from the environment and manure storage areas such as a lagoon or slurry pit. A disadvantage of culture relative to polymerase chain reaction-based organism detection (PCR) and antibody detection methods is the longer incubation period, which can be 4–8 weeks for liquid culture systems (MGIT, BD, Franklin Lakes, NJ; para-JEM, Trek Diagnostic Systems Inc, Cleveland OH) and 8–16 weeks for solid media (Herrolds Egg Yolk Medium).

Detection of MAP DNA in Fecal Samples. Detection of MAP DNA by PCR offers the advantage of reduced test turnaround time compared with culture. Diagnostic sensitivity and specificity vary according to method, but for 1 commercially available USDA-approved real-time PCR method, sensitivity and specificity are similar to culture methods. Real-time PCR methods also allow estimation of amount of MAP shedding in feces, with good correlation with culture methods.[7] These can also be applied to pooled samples and samples collected from the environment and manure storage. Detection of MAP by PCR does not confirm the presence of viable organisms and does not permit strain differentiation. With real-time PCR assays, results expressed as cycles to cross threshold (Ct values) are inversely related to the amount of MAP in the sample, and thus permit evaluation of degree of fecal shedding.

Detection of Antibodies in Serum and Milk. Detection of antibodies in serum and milk has a major cost savings advantage compared with methods used to detect organisms, and a great advantage in throughput and turnaround time compared with culture. Generally, the sensitivity of these tests is limited by the biology of the immune response to MAP, wherein detectable antibodies are usually not produced until late in infection and after fecal shedding has begun.[8] Sensitivity of serum ELISA is estimated at 30% relative to necropsy.[4] ELISA on milk samples has comparable performance.[9,10] Incorporation of milk ELISA testing on samples collected for DHIA purposes adds to the convenience and reduced sample acquisition cost of this test. Clinicians are urged not to accept simple dichotomous (positive/negative) ELISA results, but rather to interpret quantitative results (ELISA OD or S/P ratio), which correlate well with the likelihood of (and level of) fecal shedding of MAP.[11] For the most part, ELISA has supplanted AGID testing because of superior performance on samples from subclinically infected cattle, but AGID can be useful for diagnosis in clinically affected cattle, in which case the sensitivity approaches that of the ELISA (>90%).[12]

Detection of Cell-Mediated Immune Response. Whereas cell-mediated immune response probably predominates early in MAP infection, methods to detect such a response offer promise in early detection during the "eclipse" phase before antibodies or fecal shedding of MAP are detectable. Such tests include the intradermal johnin test, and an in vitro assay of antigen-induced gamma-interferon release from lymphocytes of test subjects in vitro. Because of cost, variable test performance, or both, neither of these methods is recommended at the current time.

Diagnostic Test Recommendations

For a more thorough analysis, readers are referred to a consensus statement on paratuberculosis diagnostic testing developed by a panel of experts, including two of the co-authors of this ACVIM consensus statement (AJR, MTC), and a subsequent review.[4,13]

Diagnosis of MAP Infection in an Individual Animal with Clinical Signs. In situations where a bovid has clinical signs compatible with Johne's disease, antibody tests, fecal culture, and fecal PCR will all perform with comparable sensitivity (>90%). If clinical disposition of the animal depends on the results, then culture is not practical and one of the more rapid methods should be employed. If MAP has never been diagnosed in the herd, organism detection to confirm the diagnosis is preferred.

Evidence level for this recommendation: Strong. Multiple blinded diagnostic test comparison studies have been published.

Diagnostic Tests to Determine Herd MAP Status. Producers may choose herd testing with the goal of participation in the US Voluntary Bovine Johne's Disease Control Program (USVBJDCP), the Canadian Voluntary Johne's Disease Prevention and Control

Program (CVJDPCP), or similar programs in other countries. Alternatively, the producer might wish to determine herd status or within-herd prevalence outside the auspices of an official control program. The reader is referred to program guideline documents for the USVBJDCP and CVJDPCP, which outline prescribed testing strategies to establish MAP infection status for dairy and beef herds.[14,15] Increasing stringency of testing allows a herd to advance through the classification ranks, indicating higher confidence of uninfected status. Examples of testing strategies include the following:

Environmental testing: Organism detection tests applied to samples of manure collected from 6 on-farm cow congregation areas, alleyways, freestall barns, and manure storage areas (pit, lagoon, slurry) allow determination of whether a herd is MAP-infected, with 70–80% sensitivity.[16] This low-cost procedure is the most cost-effective method to determine if a herd is infected or likely uninfected and eligible to advance to more advanced test-negative status, because it allows for awarding entry-level status in the USVBJDCP (for dairies) and might be employed in some provinces according to the CVJDPCP. This is recommended as a cost-effective testing procedure for initial determination of herd infection status in previously untested herds.

Evidence level for this recommendation: Strong (for dairy), based on prospective epidemiologic field trials. Evidence for beef cow-calf herds is weak.

Testing of samples from individual animals: To estimate prevalence within a herd, samples for ELISA testing (serum, milk for dairy cows) or individual fecal samples for organism detection testing can be collected from every animal in the herd, from a random statistical subset of animals, or from targeted animals (based on age and clinical appearance/body condition score). Organism detection methods can be applied to individual samples or pooled for cost savings. In most scenarios, cows over 36 months of age are targeted. The choice of which strategy (ELISA versus organism detection, whole-herd, subset, targeted, pooled) will be determined by size of herd, costs, and goals of the producer including whether he/she aspires to a specific Herd Classification Level in the USVBJDCP, CVJDPCP, or other regulatory program. Clinicians are urged to consult program guidelines, which indicate the number of animals and specific tests to be chosen for each classification level.[14,15] It is likely that most commercial herds will choose ELISA strategies that employ subsets of animals, or pooled fecal strategies, while genetic seed-stock herds, which have a requirement to establish a more stringent Status Level more quickly, will opt for whole-herd tests, employing organism detection, despite the higher cost.

Evidence level for this recommendation: Strong, based on sample size calculations and published values for diagnostic test performance characteristics in the development of USVBJDCP and CVJDPCP testing standards.

Bulk tank milk testing: Bulk milk offers an attractive testing matrix as a "whole-herd pool" that could provide information on herd status for the cost of a single sample test. Organism detection methods (PCR and culture) have not shown sufficient sensitivity to warrant a recommendation for their use at this time.[17] Results of studies on sensitivity of ELISA applied to bulk tank milk have also yielded mixed results. While some studies have suggested insufficient test sensitivity and specificity, a more recent large-scale study employing modified ELISA cut-offs for bulk milk demonstrated 85% sensitivity to detect herds with a 3% or higher seroprevalence, with 96% specificity.[18,19] This test shows promise, but is not recommended at this time as further validation is required.

Evidence level for this recommendation: The evidence in favor of employing these tests is weak, based on conflicting study results and only a single large-scale prospective study showing promise.

Diagnostic Testing in a Known-Infected Herd's Control Program. Determination of individual cows' MAP infection status might be a component of some control programs in infected herds. For example, testing might facilitate colostrum management, segregation of cows at the time of calving, and culling of fecal shedders to reduce environmental MAP load and risk of exposure for susceptible animals. For commercial herds, with MAP apparent prevalence >5%, use of a serum (beef and dairy) or milk (dairy) ELISA is recommended. It is imperative that management decisions are based on the magnitude of the result, not just the dichotomous result. For example, the control protocol might call for limiting access of all test-positive cows to calving pens or use of colostrum only from all ELISA-negative animals, but only those animals with "high positive" results are targeted for culling. In herds with <5% prevalence, organism detection tests applied to pooled or individual fecal samples is recommended, because the predictive value of ELISA-positive results in those low prevalence herds is low.

Evidence level for this recommendation: Strong evidence exists that infectious cows can be detected by available diagnostic tests, and that doing so is important for control. Studies documenting the overall efficacy of management recommendations for control of paratuberculosis are limited.[20]

Diagnostic Testing for Eradication of MAP from an Infected Herd. Diagnostic testing to identify MAP-infected animals for culling will be a component of the plan for most infected herds attempting eradication. The choice of tests will be dictated by how aggressively the producer wishes to pursue eradication, which in turn will hinge on economic factors such as starting prevalence, and whether income from selling seed stock is a goal. The cost will generally be inversely related to the rapidity with which eradication can be approached. For example, the most rapid and most expensive scheme would be to perform organism detection tests on fecal samples from individual cows. While this might be necessary for a seed-stock herd, for example, a commercial dairy might prefer a more gradual but less expensive approach, such as employing pooled

fecal testing or serum or milk ELISA, until the prevalence has been reduced to <5%. Regardless of the initial approach or type of herd, once the prevalence has been reduced to <5%, ELISA will not have sufficient sensitivity, and organism detection testing applied to individual fecal samples will be necessary.

Evidence level for this recommendation: Moderate. The test characteristics are well-described. Reports of eradication of paratuberculosis from infected herds using a combination of test-cull and management recommendations are scarce.

Diagnostic Testing to Screen Herd Additions, Embryo Transfer Recipients, Herd Sires, etc. It is not appropriate to rely on diagnostic tests of purchased animals to avoid introduction of MAP-infected animals into herds, especially when these animals are <36 months old. Rather, prepurchase screening should first and foremost be based on proof of Johne's disease status of the source herd. In order of preference, the source herd should (1) be certified in the USVBJDCP Test Negative Level 4 or higher (or comparable level in CVJDPCP or other official program); (2) if not enrolled in an official program, have a history of testing comparable to USVBJDCP Status Level 4; (3) prior to executing the sale, allow ELISA testing of all cows >36 months (or statistical subset) to confirm the source herd's low-risk status; (4) if source herd testing is not possible, prepurchase testing of herd additions by both serum ELISA and fecal organism detection, with at least annual follow-up testing.

Evidence level for this recommendation: Strong. Risk of purchase of MAP-infected animals from untested source herds in the United States is well-documented in national cross-sectional prevalence surveys conducted by NAHMS.[1] Risk reduction in associated with satisfaction of VBJDCP Status Test Negative levels is also well-documented.[21]

Diagnostic Testing in Sheep, Goats, and Camelids. Both of the ELISA kits commercially available in the United States (IDEXX-PourquierHerdCheck; Prionics Parachek2) may be used with serum from sheep and goats. Sensitivity and specificity in these species appear comparable to cattle.[22,23] Thus, these tests can be applied in a similar fashion as for cattle, eg, for confirmation of diagnosis in an animal with clinical signs of Johne's disease (subject to confirmation by organism detection or postmortem if 1st case in herd/flock), or for screening subclinical animals in infected herds for culling or management decisions. Sheep strains of MAP are difficult to cultivate in vitro, but a modified liquid culture method with good sensitivity has been developed in Australia.[24] Using this culture method, testing fecal samples in pools of 50 sheep provided 95% confidence of detecting 2% prevalence in the flock, and was more sensitive than ELISA for identifying infected flocks.[25] Testing by PCR is more sensitive than culture of individual fecal samples from experimentally infected sheep.[26] In goats, culture for MAP also has been shown to have low sensitivity,[23] thus the recommended organism detection method for fecal samples is PCR. Serologic tests have not been useful in camelids, and organism detection by PCR on fecal samples is recommended.

Evidence for this recommendation: Moderate for goats and sheep, based on multiple field and experimental studies. Evidence is weak for camelids, based on case series and small experimental studies.

Control of Paratuberculosis

Keep Free Herds Free

At the herd level, control of paratuberculosis requires good biosecurity to prevent introduction of MAP. The potential cost associated with paratuberculosis control, once introduced, warrants active efforts at infection prevention. Producers should be educated about the risk and costs of MAP infection introduction, proper hygiene, and biosecurity measures. These measures are not MAP control-specific, but are also applicable for control of other pathogens such as BVDV, BHV (IBR), viral diarrhea, *Salmonella*, and mastitis pathogens.

Recommendations to Prevent MAP Introduction into Herds:

- Do not introduce (buy or lease) animals from herds with Johne's disease, or from herds with an unknown Johne's disease status.
- Do not participate in auctions or shows where there is a risk that animals will be reintroduced into the herd following prolonged (indirect) contact with animals with unknown status. Recent data suggest that adult infection is a realistic scenario.[27]
- Young stock management:
  - On-farm calf rearing according to management and hygiene procedures described below
  - Off-site (commercial) calf rearing when such a rearing facility adheres to the appropriate management practices and accepts only calves from MAP-tested unsuspected herds.
- Breed rather than buy replacement heifers.
- Avoid applying manure from farms with Johne's disease, or from herds with an unknown Johne's disease status, to pastures or forage crops.
- Avoid using equipment potentially contaminated with manure or soil from properties with Johne's disease, or from herds with an unknown Johne's disease status.
- Demand and enforce biosecurity measures from professionals (veterinary and otherwise) and incidental visitors as well as family members with access to other herds.
- Document the herd status with a long-term monitoring system through use of a repeated diagnostic testing scheme, such as those prescribed in the US-VBJDCP or CVJDPCP guidelines. Combined with a risk assessment and management plan, this would also enable official recognition of herd status.

Evidence level for risk assessment-based control procedures is moderate and mostly based on epidemiologic

risk assessment association studies and expert opinions; evidence level for preventing introduction through biosecurity measures is strong, evidence level for adult infections is moderate. Evidence level for introduction via contaminated fecal material is weak.

Managing Infected Herds

The procedures for management of MAP-infected herds will be dictated by the aim of the intervention. The intervention might be aimed at paratuberculosis control at the herd level (stabilize and preferably decrease prevalence) or to eradicate paratuberculosis. An alternative reason for intervention could be taken from a food safety perspective, aiming to reduce or eradicate the burden of (viable) MAP in dairy or meat products. These types of interventions could be dictated by dairy or beef producer organizations, industry (dairy or beef processing companies), or regulatory authorities.

Managing MAP-infected herds can be done through various combinations of three basic strategies:

- *Prevent new infections*: biosecurity, hygiene and management solutions
- *Manage infected animals*: test-and-manage/cull programs
- *Improve resistance to paratuberculosis*: vaccination and genetic selection

These strategies can be employed in varying degrees of stringency or aggressiveness, depending on whether the goal is stabilization or reduction in prevalence, or eradication.

To have the desired effect, a program should be herd-specific and practical, and the herd owner must be committed. This can be achieved by developing a farm-specific Risk Assessment and Management Plan (RAMP).[28,29] Although the collection of the necessary information requires extensive on-farm time, it is the basis for a control program to which producers are likely to adhere.[30] Time lines for managing infected herds are long (10+ years), and this requires regular monitoring by the herd veterinarian to keep the program "on-task." It is of little value to initiate costly testing schemes, without a long-term commitment to act on test results and pursue management changes as needed. Recent data indicate that with implementation of a long-term management strategy, Johne's disease can be controlled successfully.[20]

Prevent New Infections. The most susceptible animals in a herd are the newborn calves and young stock and the majority of new infections probably occur in these age groups, so preventing exposure of these animals is a major component of control protocols.[31] The younger that animals are exposed to MAP, the more likely they are to start shedding early and progress to clinical disease.[31,32]

In a proportion of animals, in utero transmission will occur. The prevalence of infected fetuses among cows with subclinical and clinical disease has been estimated to be 9% (95% confidence limits 6–14%) and 39% (20–60%), respectively.[33] It is important to note that little information is available regarding the consequences of in utero infection. Case studies indicate that chronic infection is likely; however, it is unknown if in utero-infected animals progress to clinical disease faster or start shedding earlier in life.[33] Farms attempting to control Johne's disease should be advised to cull calves born to dams with clinical signs of disease. For farms trying to eradicate Johne's disease, more rigorous measures would include culling offspring born the year before the dam developed clinical disease, and most stringent would be culling of animals born to subclinically infected dams.

The fecal–oral route is generally considered the major transmission route postpartum. However, recent data suggest that alternative transmission routes such as bioaerosol should be considered.[34] Transmission of infection likely can be most effectively stopped at the level of preventing newborn calves and young stock from contact with fecal matter from adult cattle, be it via feed, water, fomites, dust, or other environmental sources.[35] Separation of calves from cows at birth, as well as spatial separation of calf-raising areas from adult animals, is advised. Spatial separation should preferably be in separate barns to reduce contact with infectious MAP bioaerosols (such as dust).[34] Separate equipment should be used for handling feed and scraping or hauling manure.

Specific recommendations—dairy:

- Maternity pen/calving area:
  - Do not allow sick, lame, or Johne's disease test-positive cows in group maternity pens.
  - Maintain clean maternity pen bedding by adding 25 pounds of new straw per cow per day and providing 100 square feet of space per cow in that pen.
  - Keep cows in calving pens clean; limit manure on flanks, legs, udders, and teats.
  - Remove or isolate calves from cows within 10 minutes of calving or before standing attempts are made. This limits calf contact with manure in the calving pen or on commingled cattle, which is inevitable during standing attempts and teat seeking. Immediate removal of calves from the calving area is a necessity when other adult occupants of the calving area may be MAP-infected.
- Colostrum management:
  - Do not feed colostrum from cows of unknown MAP test status to calves.
  - Colostrum from MAP test-negative cows can be used to build a colostrum bank.
  - Clean teats thoroughly to insure sanitary colostrum collection.
  - Do not pool colostrum; use individual bags or containers.

- ○ Heat treat colostrum—60°C (140°F) for 60 minutes.[36] Use standard plate and coliform counts to monitor the effectiveness of pasteurization.
- ○ Store colostrum at 3–4°C (38–40°F) for no longer than 3 days, then discard or freeze.
- ○ Colostrum replacement products made from plasma have been shown to reduce the likelihood of calves becoming MAP-infected as compared with calves fed raw colostrum.[37]

- Calf rearing:

- ○ Raise calves well-separated from adult cattle.
- ○ Feed calves milk replacer or pasteurized milk-batch pasteurization at 65.5°C (149.9°F) for 30 minutes[38] or high-temperature short time (HTST) pasteurization at 72°C (161°F) for 15 seconds.[39] Use standard plate and coliform counts to monitor the effectiveness of pasteurization. Feeding pasteurized milk or milk replacer minimizes, but does not eliminate, the risk of MAP infection. Pasteurization of waste milk reduces the level of bacteria, but does not sterilize it. Compared with feeding waste milk, the risk of feeding MAP in pasteurized milk is significantly lower. It is recognized that, even with effective pasteurization, postpasteurization contamination with MAP is possible. Uncontaminated milk replacer powder is MAP-free. With exemplary mixing and delivery in sanitized equipment, MAP exposure is unlikely.
- ○ Prevent contamination of calf grain, water, feeding implements, and calf pens by manure from adult cattle (eg, shoes, hands, clothing of personnel, vehicles, carts).
- ○ Do not feed manger sweepings to heifers.
- ○ For heifers originating from high prevalence dairy herds, consider testing for fecal shedding between 7 and 14 months of age.[40]

Specific recommendations—beef cow-calf:

- Environmental management:

- ○ Remove cow-calf pairs from calving area as soon as possible after calving.
- ○ Maintain segregated calving areas for MAP test-positive cows.
- ○ Assure that cattle have clean water source; ponds may become MAP-contaminated and serve as a source of MAP infection.

- Herd sires are MAP test-negative in bull-bred herds

Manage infected animals. *Test and cull/manage strategies:* The management of infected animals should be dictated by a farm-specific RAMP. The RAMP should include a cost-effective diagnostic testing plan to identify infected animals and to categorize them according to risk based on quantitative results (colony count on solid culture media, time to detection on liquid media culture media, Ct value for PCR, OD or S/P ratio for milk or serum ELISA). The RAMP-dictated management or fate of the animals assigned to the various risk categories will depend on the herd goals, which might vary from stabilizing MAP infection prevalence all the way to eradication. The RAMP should specify how infected animals in each risk category are managed viz-a-viz calving stall management, colostrum management, culling (immediate versus end of lactation), and culling of infected cows' offspring.

For example, a commercial dairy herd with >5% infection prevalence, with a goal of managing or reducing prevalence, might manage infected animals in the following way:

- Clinical suspects: cull immediately.
- High risk/heavy shedder (high ELISA OD values, high positive organism detection): cull immediately.
- Medium risk/medium shedder: cull if other problems, or cull at end of lactation, do not breed.
- Low risk (low ELISA OD, low organism detection): Do not breed, cull at end of next lactation, do not use colostrum, segregated calving pen.

If eradication is the goal, then a more aggressive approach to culling, including culling of daughters of clinical cases and heavy shedders might be implemented.

Decisions on animals from noncommercial farms (pets, hobby farm, rescue, petting zoo, etc) or valuable genetic stock can be substantially different, as reluctance to cull and a desire to treat may be encountered. In these cases, potential exposure of other animals and people to animals excreting large amounts of MAP should be part of the final decision-making plan.

Improving Resistance to Paratuberculosis. *Vaccination:* Vaccination against paratuberculosis was first described in 1926 as a whole-cell vaccine with an oil-based adjuvant, and has essentially not changed over the years. Vaccination against Johne's disease has been recognized as a measure to control the disease in cattle and small ruminants. In several countries in southern Europe, as well as in Australia, vaccination is extensively and successfully used to control Johne's disease, especially in goats and sheep.[41] In cattle, the use of the vaccine is less common in most countries. In many countries, this is caused by the fact that whole-cell Johne's disease vaccines may interfere with diagnostic testing for bovine tuberculosis.[42]

Only one approved vaccine is available in the United States, Mycopar (Boehringer Ingelheim). The use of the vaccine is regulated (USDA Veterinary Services Memo No. 553.4 *Mycobacterium paratuberculosis bacterin: Use in Johne's Disease Vaccination Programs in Participating States*), and purchase and administration are limited to state-approved veterinarians. The vaccine is based on a formulation of (killed) Mycobacterium cells in an oil-based adjuvant and is therefore comparable to Freund's Complete Adjuvant, and

has potentially severe adverse effects when incorrectly applied or following accidental self-inoculation.

Both experimental and field studies show that the bacterin prevents the progression of infected cattle to clinical disease in most cases. The effect on reduction of bacterial shedding is less pronounced, and the effect on the prevalence of infected animals in many studies is limited. Therefore, it is essential to use vaccination as one of many tools consistent with a RAMP. The vaccine cannot replace a good management plan and beneficial effects of vaccination can be overwhelmed by heavy MAP exposure.

The vaccine performs better in sheep, and clinical trials in Australia with the Gudair (Pfizer Animal Health Australia, West Ryde, Australia) bacterin vaccine show a 90% reduction in within-flock prevalence in vaccinated flocks.[43] Vaccination has since become a valuable part of Australian ovine Johne's disease control.

Genetic selection: Using genetic selection as a tool to control JD is a relatively new approach. The phenotype (infection status) shown by individual animals is a combination of genetically determined factors (susceptibility and resistance genes) and environmental factors (exposure to MAP). Following initial studies indicating genetically transmittable effects of susceptibility to MAP in Dutch dairy cattle,[44] similar effects have been observed in cattle populations in the United States.[45] Genetic effects can be quantified best at sire level using phenotype of daughters, and heritability estimates range from 1 to 18% with the majority of estimates between 9 and 12%. Similar heritability estimates have been reported for sheep. Estimates depend on methods used to determine phenotype (diagnostic assays) and sample size, which probably explains most of the difference between estimates. Both candidate-gene approaches (studying selected genes based on expected biologic importance) and whole-genome approaches (eg, using single nucleotide polymorphism [SNP] arrays) have been used to find and evaluate genes potentially involved in susceptibility to MAP infection. Current data indicate that significant genetic variation is present in the Holstein cattle population, which would allow for genetic selection strategies, which are probably most suited to breeding more resistant animals, rather than using individual animal genetic marker profiles, although the latter will probably be developed as genetic typing techniques further develop.[46]

It should be considered that using a test-and-cull strategy in a management plan also entails a form of genetic selection at the phenotype level, but a strategy likely to be less effective compared with sire-based strategies. A potential problem but hereto not investigated question is whether selection based on immunologically determined phenotypes (eg, the commonly used ELISA assays) leads to selection for not responding in these diagnostic assays rather than resistance to Johne's disease. A safer route would be to use organism detection diagnostic data for these purposes. The advantage of using genetic marker profiling would be to predict the risk of the animal in contracting Johne's disease, which could be used in selection decisions prior to the animal becoming infectious.[46]

The evidence for the efficacy of stabilizing or reducing within-herd MAP infection prevalence through a combination of preventing new infections and managing infected animals is strong, based on experimental studies, clinical reviews, and expert opinion. The evidence for vaccination reducing incidence of clinical Johne's disease is strong, but the evidence for reducing prevalence or fecal shedding rate is weak based on conflicting results of different studies. The evidence for genetic influence on resistance is strong, but evidence for the utility of genetic selection in paratuberculosis control is yet to be generated.

Treatment of Paratuberculosis

Before discussing treatment of paratuberculosis, it is prudent to consider several facts. There is currently no cure for paratuberculosis. Treatment is usually aimed at reducing clinical signs of weight loss and diarrhea, but cannot be expected to prevent shedding of the organism or clearance of the organism from tissues. Therefore, if appropriate biosecurity practices are not implemented, treatment can potentially increase the risk of environmental contamination and transmission by extending the life of a normal appearing animal that is shedding the organism. There are no drugs approved for treatment of paratuberculosis in North America, although monensin is approved for control in Canada. Treatment, in the context of this paper, refers to treatment of exceptional production or sport animals to allow for the collection of embryos or semen, and treatment of pet animals that will be managed in a way to prevent contamination of the environment with MAP. With the exception of monensin, none of these drugs is approved for use in food animal species. Because withdrawal times have not been established for most of them, and because treatment will be lifelong, the owners should be made aware that they are relinquishing the possibility of salvage of these animals for human food if they choose to try treatment. In addition, the treatment cost for most of these medications for cattle is high.

Traditionally, drugs for treatment of paratuberculosis were selected because of their use against other mycobacterial infections, such as tuberculosis in humans. These drugs include isoniazid, rifampin, and the anti-leprosy drug, clofazimine. Interestingly, in vitro susceptibility testing does not predict efficacy for these antibiotics against MAP, despite the clinical reports of success.[47] In vitro testing has shown MAP to be susceptible to azithromycin, which has not been tested in vivo, amikacin, which is usually cost-prohibitive, and monensin.[47,48]

One of the first drugs to be used as a treatment for paratuberculosis in cattle was isoniazid (isonicotinic acid). There are a number of case reports citing its use in the treatment of paratuberculosis, as well as bovine tuberculosis.[49,50] Unfortunately, these case reports usually described treatment in a very few animals.

Isoniazid is readily absorbed following oral administration to ruminants.[51] This drug only kills MAP during the growing phase, so it acts as a bacteriostatic drug, creating a state of remission while the treatment is being administered. A bacteriologic cure cannot be expected from treatment with isoniazid alone. Results from use of this drug have varied from rapid and dramatic improvement, with resolution of diarrhea in about 2 weeks, to complete failure.[49,50] The therapeutic spectrum is narrow. The recommended daily dosage range is 10–20 mg/kg. Intoxication has been reported at a dosage of 30 mg/kg daily.

Clofazimine, a drug used to treat leprosy in people, has been used successfully to treat ruminants with paratuberculosis. It is currently unavailable in the United States. It was initially used as a chemoprophylactic agent and as a treatment in sheep with experimentally induced and naturally occurring paratuberculosis, respectively. It appeared to reduce the number of organisms in tissues of infected animals when administered orally at 15 mg/kg daily.[52] It was later reported to be effective in cattle with naturally occurring paratuberculosis at an oral dosage of 2 mg/kg daily.[53,54] The clinical response was rapid and sustained during treatment, but bacteriologic cure was not achieved even though shedding was reduced. The authors of the study recommended a dosage of 600–1,000 mg administered orally daily to mature cattle for their entire life.

Rifampin, which has been used extensively for the treatment of human tuberculosis and *Rhodococcus equi* infections in foals, has also been used in combination with other drugs for the treatment of paratuberculosis. Combination therapy of streptomycin (10 mg/kg twice daily), rifampin (10 mg/kg once daily), and levamisole was successful in reducing the clinical signs in experimentally infected rabbits with paratuberculosis.[55] This combination, with isoniazid added, was also used to treat a goat successfully.[56] In both of these situations, no MAP was cultured from tissues at necropsy. Based on pharmacologic studies, the recommended dosage of rifampin in ruminants is 10–20 mg/kg administered orally.[57,58]

A study has suggested that weekly injections of levamisole at a dosage of 2.5 mg/kg reduced fecal shedding (based on acid fast staining) in cattle with subclinical paratuberculosis.[59] The authors suggested that the effect was mediated through the immunomodulatory effects of the compound. Levamisole may be a reasonable adjunctive therapy for animals treated for paratuberculosis.

Gallium nitrate, which has an inhibitory effect against several intracellular bacterial species, has been shown to inhibit the growth of MAP in vitro in a dose-dependent fashion.[60] Calves challenged with viable MAP organisms and then administered gallium nitrate at 20 mg/kg for 45 days had a lower bacterial burden in tissues at necropsy than control calves.[61]

Dietzia subspecies *C79793-74* was first observed to be a bacterial contaminant that inhibited the growth of MAP on fecal culture. Several papers from a single research group at a commercial laboratory describe the use of this probiotic bacterium as a preventative and treatment for paratuberculosis. In these studies, the probiotic was administered to cattle diagnosed by culture or serologic test with subclinical paratuberculosis, animals with clinical disease, and neonatal calves exposed to MAP in the environment.[62–64] Subclinically affected cattle appeared to live longer than controls, responded to higher doses of *Dietzia,* which were administered when clinical signs appeared, and in some cases, the clinical and pathologic effects of infection appeared to be suppressed. Several clinically affected cattle responded to *Dietzia,* with or without dexamethasone, in a manner similar to the response seen after other therapeutic agents described above.[63] Finally, neonatal calves fed viable *Dietzia,* but not inactivated *Dietzia* or control rations, appeared to be resistant to infection.[62] While the authors have a commercial interest in the product, and the methods of the trial were somewhat unconventional, there appears to be enough evidence of efficacy to warrant further study.

One of the most promising therapeutic agents for the treatment of clinical paratuberculosis appears to be monensin. It is approved for use in food-producing animals in North America for other indications with no withdrawal time. It is relatively nontoxic when fed at recommended levels and it is relatively inexpensive. However, in the United States, the use of a feed additive in an extra-label fashion is prohibited. Therefore, cattle can only benefit from the effects of monensin on paratuberculosis if they are being fed the product for another label indication. The first report of using monesin in cattle with paratuberculosis reported that histologic lesions improved after cattle were fed monensin at 450 mg/head for 120 days.[65] While not part of the statistical evaluation, body condition score in the treated cattle was substantially better than the control cattle. In an experimental challenge model, monensin was shown to reduce the number of MAP organisms in the tissues of infected calves compared with controls.[66] While there are no published reports of the use of monensin to treat clinically affected cattle, one of the authors (AJR) has observed several cattle with advanced clinical disease, which experienced a dramatic improvement with resolution of diarrhea and substantial increase in weight and body condition while being fed monensin at a dose approved for other indications. Monensin is approved for use in cattle in Canada for the following indication: "For the reduction in fecal shedding of *Mycobacterium avium paratuberculosis* (MAP) in mature cattle in high risk Johne's disease herds as an aid in the herd control of Johne's disease as one component of a multi-component Johne's disease control program." Two studies were performed on Canadian cattle to evaluate the effect of monensin as a feed additive or as a controlled release capsule (CRC) on cattle with paratuberculosis.[67,68] Monensin was associated with reduced odds of a cow testing positive to ELISA in herds in which paratuberculosis had not been diagnosed before. In herds with a prior history of paratuberculosis, feeding monensin to breeding age heifers was associated with decreased

odds of cows in the herd being ELISA-positive.[67] Cattle receiving a CRC had a slight reduction in fecal shedding of MAP.[68] It appears that treatment with monensin can result in amelioration of clinical signs in cattle with paratuberculosis, but has a minimal effect on shedding of the organisms. This could potentially lead to an increase in the total bioburden on infected dairy farms. Cattle shedding the organism may remain economically viable for a longer period of time, thus increasing their total lifetime environmental contamination potential.

On the basis of these reports, the recommended treatment protocol for a cow, sheep, goat, or alpaca with clinical Johne's disease is once-daily oral treatment with rifampin (10–20 mg/kg) and isoniazid (10–20 mg/kg). Monensin should be included if it can be legally administered for its label claims (prevention of coccidiosis in goats [22 ppm in feed] and beef cattle [200 mg/head/day], increased milk production efficiency in dairy cattle [410 mg/head/day]).

Evidence for the recommendation of monensin is moderate, based on experimental studies and clinical reports. Evidence for rifampin and isoniazid is weak, based on clinical reports.

Zoonotic Potential of MAP

MAP has been incriminated as a triggering agent for Crohn's disease in genetically susceptible individuals. There are multiple similarities and few differences between Johne's disease in animals and Crohn's disease in humans. Both diseases are chronic inflammatory bowel diseases primarily targeting the ileum, characterized by diffuse granulomatous inflammation without caseation. The triggering event in Johne's disease is known to be MAP. The triggering event for Crohn's disease is not known, but MAP is one of several agents suspected to be a contributing factor. Current thought suggests that Crohn's disease ensues as an aberrant host–pathogen interaction—an abnormally florid immune response to antigens in the gastrointestinal tract. Many scientists now believe that MAP is one such organism whose antigens can trigger the response. The etiology of Crohn's disease is clearly multifactorial, with genetically determined susceptibility and exposure to triggering antigens just two of the factors involved. The disease may well be a syndrome with multiple etiologies, one of which may be MAP. Some authors argue that there is "no conclusive evidence" that MAP is a cause of Crohn's disease. Direct scientific evidence that MAP is a human pathogen by experimental challenge of young children to fulfill Koch's postulates is unethical. There will probably never be conclusive evidence. Instead, the scientific community must base any decision on the zoonotic potential of MAP on multiple indirect lines of evidence as discussed below.

MAP is a versatile pathogen. It primarily infects ruminants, but has also been reported in nonruminants, including nonhuman primates. Molecular fingerprinting demonstrates extensive MAP strain sharing among species.[69] Evidence that MAP can infect taxonomically diverse species is strong.

MAP contaminates foods of animal origin.[70–72] The infection in ruminants becomes disseminated (systemic) in the latter stages, probably around the time animals become test-positive for antibodies in serum or milk.[73] MAP has been recovered from lymph nodes and organs distant from the gastrointestinal tract and the fetus of pregnant females as well as muscle tissue.[33,74] It is primarily excreted in feces, but is also excreted in colostrum and milk. For these reasons, raw meat and milk products can be MAP-contaminated antemortem as well as postmortem via fecal contamination of carcasses at the abattoir or milk at the time of milking. Domestic water supplies originating from surface waters represent another potential route of human exposure.[75] Evidence that humans are exposed to MAP through foods derived from infected animals is strong and evidence of exposure via domestic water supplies is weak.

The number of MAP in foods of animal origin is greatly reduced by processes such as pasteurization and cooking in a time- and temperature-dependent fashion. Low levels of MAP have been found in retail HTST (72°C × 15 s) pasteurized milk and infant formula by multiple studies in multiple countries.[76–78] Evidence for human exposure to MAP via HTST-pasteurized milk is strong. A recent report that MAP can produce spores provides an explanation for its thermal resistance.[79] Evidence for human exposure to MAP via ground beef is weaker; while MAP has been recovered from muscle meat, no surveys of retail ground beef for MAP have been reported.

Crohn's disease is regarded by most medical gastroenterologists as a genetically mediated autoimmune disease. However, the incidence of Crohn's disease is rising in most countries, suggesting that an environmental trigger plays an important role for genetically susceptible people. Interestingly, many of the genes associated with higher frequency of Crohn's disease are also those affecting susceptibility to mycobacterial infection.[80]

MAP is found in Crohn's patients significantly more often than in unaffected controls. Two meta-analyses arrived at this conclusion based on rigorous scrutiny of dozens of publications.[81,82] These data support an association of MAP and Crohn's disease. Most studies searching for MAP in Crohn's patients report MAP detection by PCR methods and few successfully culture the pathogen. Experts suspect that MAP finds humans an abnormal host and adopts a different form, eg, a spheroplast (cell wall-deficient) or other viable nonculturable form. Crohn's patients also have both humoral and cellular immune responses directed specifically at MAP. Evidence for MAP association with Crohn's disease is strong, but association must not be confused with causation.

A recent review paper evaluated the casual relationship between MAP and Crohn's disease using Hill's 10 criteria for causality. For seven of the criteria, the authors gauged the strength of evidence as "strong" or "moderate," and for two criteria, "conflicting."[3] Conflicting evidence comes from MAP detection studies

where MAP detection has ranged from zero to 100% of Crohn's patients, and MAP has been found in a low proportion of control subjects, although meta-analysis supports MAP association with Crohn's disease.[83] Interventional studies, ie, treatment trials using anti-mycobacterial drugs to treat Crohn's disease, are also conflicting.[84] It is beyond the scope of this document to discuss these reports in more detail, but it should be noted that treatment of *Mycobacterium avium* infections is problematic; in vitro drug susceptibility often fails to predict in vivo treatment efficacy.[85]

Viewed as a murder suspect, MAP has both the means (it is an obligate pathogen requiring animal infections to sustain itself in nature) and opportunity (viable MAP contaminate food).[71] Evidence of a causal relationship between MAP and Crohn's or any other human disease will be indirect. At this point in history, we judge the strength of evidence that MAP is a cause of Crohn's disease to be moderate. Successful randomized double-blind treatment trials using appropriate anti-MAP drugs in combination, for a sufficient duration (likely 2 years), combined with diets that are MAP antigen-free, will be the evidence that most strongly affects scientific opinion on the zoonotic potential of MAP.

Acknowledgment

Conflict of Interest Declaration: Authors disclose no conflict of interest.

References

1. USDA. 2008. Johne's Disease on US Dairies, 1991–2007. Fort Collins, CO: USDA-APHIS-VS, CEAH, National Animal Health Monitoring System. N521.0408.

2. USDA. 1999. What Do I Need to Know About Johne's Disease in Beef Cattle? Fort Collins, CO: USDA-APHIS-VS, CEAH, National Animal Health Monitoring System. N309.899.

3. Chiodini RJ, Chamberlin WM, Sarosiek J, et al. Crohn's disease and the mycobacterioses: A quarter century later. Causation or simple association? Crit Rev Microbiol 2012;38:52–93.

4. Collins MT, Gardner IA, Garry FB, et al. Consensus recommendations on diagnostic testing for the detection of paratuberculosis in cattle in the United States. J Am Vet Med Assoc 2006;299:1912–1919.

5. Sweeney RW, Whitlock RH, Hamir AN, et al. Isolation of *Mycobacterium paratuberculosis* after oral inoculation in uninfected cattle. Am J Vet Res 1992;53:1312–1314.

6. Fecteau ME, Whitlock RH, Buergelt CD, et al. Exposure of young dairy cattle to *Mycobacterium avium* subsp. *paratuberculosis* (MAP) through intensive grazing of contaminated pastures in a herd positive for Johne's disease. Canadian Vet J 2010;51:198–200.

7. Aly SS, Mangold BL, Whitlock RH, et al. Correlation between Herrold's egg yolk medium culture and real-time quantitative polymerase chain reaction results for *Mycobacterium avium* subspecies *paratuberculosis* in pooled fecal and environmental samples. J Vet Diagn Invest 2010;22:677–683.

8. Sweeney RW, Whitlock RH, McAdams S, et al. Longitudinal study of ELISA seroreactivity to *Mycobacterum avium* subsp. *paratuberculosis* in infected cattle and culture-negative herd mates. J Vet Diagn Invest 2006;18:2–6.

9. Lombard JE, Byrem TM, Wagner BA, et al. Comparison of milk and serum enzyme-linked immunonsorbent assays for diagnosis of *Mycobacterium avium* subspecies *paratuberculosis* infection in dairy cattle. J Vet Diagn Invest 2006a;18:448–458.

10. Collins MT, Wells SJ, Petrini KR, et al. Evaluation of five antibody detection tests for diagnosis of bovine paratuberculosis. Clin Diagn Lab Immunol 2005;12:685–692.

11. Collins MT. Interpretation of a commercial bovine paratuberculosis enzyme-linked immunosorbent assay by using likelihood ratios. Clin Diagn Lab Immunol 2002;9:1367–1371.

12. Sherman DM, Markhamk RJF, Bates F. Agar gel immunnodiffusion test for diagnosis of clinical paratuberculosis in cattle. J Am Vet Med Assoc 1984;185:179–182.

13. Collins MT. Diagnosis of paratuberculosis. Vet Clin North Am Food Anim Pract 2011;27:581–591.

14. USDA-APHIS. Uniform program standards for the voluntary bovine Johne's disease control program. 2010. Available at: http://www.aphis.usda.gov/animal_health/animal_diseases/johnes/index.shtml. Accessed April 16, 2012.

15. Canadian Animal Health Coalition. The Canadian voluntary Johne's disease prevention and control program. Agriculture and Agri-Food Canada. 2006. Available at: http://www.animalhealth.ca/Programs/Documents.aspx. Accessed June 30, 2012.

16. Lombard JE, Wagner BA, Smith RL, et al. Evaluation of environmental sampling and culture to determine *Mycobacterium avium* subspecies *paratuberculosis* distribution and herd infection status on US dairy operations. J Dairy Sci 2006b;89:4163–4171.

17. Stabel JR, Wells SJ, Wagner BJ. Relationships between fecal culture, ELISA, and bulk tank milk test results for Johne's disease in US dairy herds. J Dairy Sci 2002;85:525–531.

18. Nielsen SS, Thamsborg SM, Houe H, et al. Bulk-tank milk ELISA antibodies for estimating the prevalence of paratuberculosis in Danish dairy herds. Preventive Vet Med 2000;44:1–7.

19. van Weering H, van Schaik G, van der Meulen A, et al. Diagnostic performance of Pourquier ELISA for detection of antibodies against *Mycobacterium avium* subspecies *paratuberculosis* in individual milk and bulk milk samples of dairy herds. Vet Microbiol 2007;125:49–58.

20. Collins MT, Eggleston V, Manning EJB. Successful control of Johne's disease in nine dairy herds: Results of a six-year field trial. J Dairy Sci 2010;93:1638–1643.

21. Carpenter TE, Gardner IA, Collins MT, et al. Effects of prevalence and testing by enzyme-linked immunosorbent assay and fecal culture on the risk of introduction of *Mycobacterium avium* subsp. *paratuberculosis*-infected cows into dairy herds. J Vet Diagn Invest 2004;16:31–38.

22. Gumber S, Eamens G, Whittinghton RJ. Evaluation of a Pourquier ELISA kit in relation to agar gel immunodiffusion (AGID) test for assessment of the humoral immune response in sheep and goats with and without *Mycobacterium paratuberculosis* infection. Vet Microbiol 2006;115:91–101.

23. Nielsen SS, Toft N. Ante mortem diagnosis of paratuberculosis: A review of accuracies of ELISA, interferon-gamma assay and faecal culture techniques. Vet Microbiol 2008;129:217–235.

24. Whittington RJ, Marsh I, McAllister S, et al. Evaluation of modified BACTEC 12B radiometric medium and solid media for culture of *Mycobacterium avium* subsp. *paratuberculosis* from sheep. J Clin Microbiol 1999;37:1077–1083.

25. Whittington RJ, Fell S, Walker D, et al. Use of pooled fecal culture for sensitive and economic detection of *Mycobacterium avium* subsp. *paratuberculosis* infection in flocks of sheep. J Clin Microbiol 2000;38:2550–2556.

26. Kawaji S, Begg DJ, Plain KM, et al. A longitudinal study to evaluate the diagnostic potential of a direct faecal quantitative PCR test for Johne's disease in sheep. Vet Microbiol 2011;148:35–44.

27. Kovich DA, Wells SJ, Friendshuh K. Evaluation of the voluntary Johne's disease herd status program as a source of replacement cattle. J Dairy Sci 2006;89:3466–3470.

28. Goeldner D, Patton E, Wheeler R, et al. Handbook for Veterinarians and Beef Producers: A Guide to Johne's Disease Risk Assessments and Management Plans for Beef Herds, 4th ed. US Animal Health Association Johne's Committee; 2011. Available at: http://www.johnesdisease.org/. Accessed April 16, 2012.

29. Goeldner D, Patton E, Wheeler R, et al. Handbook for Veterinarians and Dairy Producers: A Guide to Johne's Disease Risk Assessments and Management Plans for Beef Herds, 4th ed. US Animal Health Association Johne's Committee; 2011. Available at: http://www.johnesdisease.org/. Accessed April 16, 2012.

30. Garry F. Control of paratuberculosis in dairy herds. Vet Clin North Am Food Anim Pract 2011;27:599–607.

31. Windsor PA, Whittington RJ. Evidence for age susceptibility of cattle to Johne's disease. Vet J 2010;184:37–44.

32. Mitchell RM, Medley GF, Collins MT, et al. A meta-analysis of the effect of dose and age at exposure on shedding of *Mycobacterium avium* subspecies *paratuberculosis* (MAP) in experimentally infected calves and cows. Epidemiol Infect 2012;140:231–246.

33. Whittington RJ, Windsor PA. *In utero* infection of cattle with *Mycobacterium avium* subsp. *paratuberculosis*: A critical review and meta-analysis. Vet J 2009;179:60–69.

34. Eisenberg SW, Nielen M, Koets AP. Within-farm transmission of bovine paratuberculosis: Recent developments. Vet Q 2012;32:31–35.

35. Dore E, Pare J, Cote G, et al. Risk factors associated with transmission of *Mycobacterium avium* subsp. *paratuberculosis* to calves within dairy herd: A systematic review. J Vet Intern Med 2012;26:32–45.

36. Elizondo-Salazar JA, Heinrichs AJ. Review: Heat treating bovine colostrum. Profess Anim Sci 2008;24:530–538.

37. Pithua P, Godden SM, Wells SJ, et al. Efficacy of feeding plasma-derived commercial colostrum replacer for the prevention of transmission of *Mycobacterium avium* subsp *paratuberculosis* in Holstein calves. J Am Vet Med Assoc 2009;234(9):1167–1176.

38. Stabel JR. On-farm batch pasteurization destroys *Mycobacterium paratuberculosis* in waste milk. J DairySci 2001;84: 24–527.

39. Stabel JR, Hurd S, Calvente L, et al. Destruction of *Mycobacterium paratuberculosis*, *Salmonella* spp., and *Mycoplasma* spp. in raw milk by a commercial on-farm high-temperature, short-time pasteurizer. J Dairy Sci 2004;87:2177–2183.

40. Bolton MW, Pillars RB, Kaneene JB, et al. Detection of *Mycobacterium avium* subsp. *paratuberculosis* in naturally exposed dairy heifers and associated risk factors. J Dairy Sci 2011;94:4669–4675.

41. Bastida F, Juste RA. Paratuberculosis control: A review with a focus on vaccination. J Immune Based Ther Vaccines 2011;9:8.

42. Santema W, Rutten V, Koets A. Bovine paratuberculosis: Recent advances in vaccine development. Vet Q 2011;31:183–191.

43. Reddacliff L, Eppleston J, Windsor P, et al. Efficacy of a killed vaccine for the control of paratuberculosis in Australian sheep flocks. Vet Microbiol 2006;115:77–90.

44. Koets AP, Adugna G, Janss LLG, et al. Genetic variation of susceptibility to *Mycobacterium avium* subsp. *paratuberculosis* infection in dairy cattle. J Dairy Sci 2000;83:2702–2708.

45. Gonda MG, Chang YM, Shook GE, et al. Genetic variation of *Mycobacterium avium* ssp. *paratuberculosis* infection in US Holsteins. J Dairy Sci 2006;89:1804–1812.

46. Kirkpatrick BW, Shook GE. Genetic susceptibility to paratuberculosis. Vet Clin North Am Food Anim Pract 2011;27:559–571, vi.

47. Krishnan MY, Manning EJB, Collins MT. Comparison of three methods for susceptibility testing of *Mycobacterium avium* subsp. *paratuberculosis* to 11 antimicrobial drugs. J Antimicrob Chemother 2009;64:310–316.

48. Brumbaugh GW, Simpson RB, Edwards JF, et al. Susceptibility of *Mycobacterium avium* subsp. *paratuberculosis* to monensin sodium or tilmicosin phosphate *in vitro* and resulting infectivity in a murine model. Can J Vet Res 2004;68:175–181.

49. Baldwin EW. Isoniazid therapy in two cases of Johne's disease. Vet Med Small Anim Clin 1976;71:1359–1362.

50. Rankin JD. Isoniazid: Its effects on *Mycobacterium johnei in vitro* and its failure to cure clinical Johne's disease in cattle. Vet Rec 1953;65:649–651.

51. Kleeberg H, Worthington RW. A modern approach to the control of bovine tuberculosis. J S Afr Vet Med Assoc 1963;3:382–390.

52. Gilmour NJL. Studies on the effect of the Rimino Phenazine B663 (630320) on *Mycobacterium johnei*. Br Vet J 1966;122: 517–521.

53. Merkal RS, Larsen AB. Clofazimine treatment of cows naturally infected with *Mycobacterium paratuberculosis*. Am J Vet Res 1973;34:27–28.

54. Whitlock RH, Divers T, Palmer J, et al. Johne's disease: A case study of clofazamine therapy in a dairy cow. In: Proc of the International Colloquium on Paratuberculosis Research, Ames, IA. 1983:231–237.

55. Mondald D, Sinha RP, Gupta MK. Effects of combination therapy in *Mycobacterium paratuberculosis* infected rabbits. Ind J Exp Biol 1994;32:318–323.

56. Slocombe RF. Combined streptomycin-isoniazid rifampin therapy in the treatment of Johne's disease in a goat. Can Vet J 1982;23:160–163.

57. Jernigan AD, St. Jean G, Rings DM, et al. Rifampin pharmacokinetics in adult sheep. Am J Vet Res 1991;52: 1626–1629.

58. Sweeney RW, Divers TJ, Benson C, et al. Pharamacokinetics of rifampin in calves and adult sheep. J Vet Pharmacol Ther 1988;11:413–416.

59. Senturk S, Mecitoglu Z, Ulgen M, et al. Effect of levamisole on faecal levels of acid-fast organisms in cows with paratuberculosis. Vet Rec 2009;165:118–119.

60. Fecteau ME, Fyock TL, McAdams SC, et al. Evaluation of the *in vitro* activity of gallium nitrate against *Mycobacterium avium* subsp. *paratuberculosis*. Am J Vet Res 2011a;72:1243–1246.

61. Fecteau ME, Whitlock RH, Fyock TL, et al. Antimicrobial activity of gallium nitrate against *Mycobacterium avium* subsp. *paratuberculosis* in neonatal calves. J Vet Intern Med 2011b;25:1152–1155.

62. Click RE. A 60-day probiotic protocol with Dietzia subsp. C79793-74 prevents development of Johne's disease parameters after *in utero* and/or neonatal MAP infection. Virulence 2011a;2:337–347.

63. Click RE. Successful treatment of asymptomatic or clinically terminal bovine *Mycobacterium avium* subspecies *paratuberculosis* infection (Johne's disease) with the bacterium Dietzia used as a probiotic alone or in combination with dexamethasone: Adaption to chronic human diarrheal diseases. Virulence 2011b;2:131–143.

64. Click RE, Van Kampen CL. Assessment of Dietzia subsp. C79793-74 for treatment of cattle with evidence of paratuberculosis. Virulence 2010;1:145–155.

65. Brumbaugh GW, Edwards JF, Roussel AJ, et al. Effect of monensin sodium on histological lesions of naturally occurring bovine paratuberculosis. J Comp Pathol 2000;123:22–28.

66. Whitlock RH, Sweeney RW, Fyock T, et al. Johne's disease: The effect of feeding monensin to reduce the bioburden of *Mycobacterium avium* subsp. *paratuberculosis* in neonatal

calves. In: Proceedings of the Am Assoc Bov Pract, Salt Lake City, UT, 2005, 191–192.

67. Hendrick SH, Duffield TF, Leslie KE, et al. Monensin might protect Ontario, Canada dairy cows from paratuberculosis milk-ELISA positivity. Prev Vet Med 2006a;76:237–248.

68. Hendrick SH, Kelton DF, Leslie KE, et al. Efficacy of monensin sodium for the reduction of fecal shedding of *Mycobacterium avium* subsp. *paratuberculosis* in infected dairy cattle. Prev Vet Med 2006b;75:206–220.

69. Stevenson K. Comparative differences between strains of *Mycobacterium avium* subsp. *paratuberculosis*. In: Behr MA, Collins DM, eds. Paratuberculosis: Organism, Disease, Control. Oxfordshire, UK: CABI;2010:126–137.

70. Eltholth MM, Marsh VR, Van Winden S, et al. Contamination of food products with *Mycobacterium avium paratuberculosis*: A systematic review. J Appl Microbiol 2009;107: 1061–1071.

71. Gill CO, Saucier L, Meadus WJ. *Mycobacterium avium* subsp. *paratuberculosis* in dairy products, meat, and drinking water. J Food Prot 2011;74:480–499.

72. Grant IR. *Mycobacterium avium* subsp. *paratuberculosis* in animal-derived foods and the environment. In: Behr MA, Collins DM, eds. Paratuberculosis – Organism, Disease, Control. Oxfordshire, UK: CABI; 2010:29–39.

73. Sweeney RW. Pathogenesis of paratuberculosis. Vet Clin N Am Food Anim Pract 2011;27:537–546.

74. Brady C, O'Grady D, O'Meara F, et al. Relationships between clinical signs, pathological changes and tissue distribution of *Mycobacterium avium* subspecies *paratuberculosis* in 21 cows from herds affected by Johne's disease. Vet Rec 2008;162:147–152.

75. Beumer A, King D, Donohue M, et al. Detection of *Mycobacterium avium* subsp. *paratuberculosis* in drinking water and biofilms by quantitative PCR. Appl Env Microbiol 2010;76:7367–7370.

76. Ayele WY, Svastova P, Roubal P, et al. *Mycobacterium avium* subspecies *paratuberculosis* cultured from locally and commercially pasteurized cow's milk in the Czech Republic. Appl Env Microbiol 2005;71:1210–1214.

77. Ellingson JLE, Anderson JL, Koziczkowski JJ, et al. Detection of viable *Mycobacterium avium* subsp. *paratuberculosis* in retail pasteurized whole milk by two culture methods and PCR. J Food Prot 2005;67:966–972.

78. Grant IR, Williams AG, Rowe MT, et al. Efficacy of various pasteurization time-temperature conditions in combination with homogenization on inactivation of *Mycobacterium avium* subsp. *paratuberculosis* in milk. Appl Env Microbiol 2005;71: 2853–2861.

79. Lamont EA, Bannantine JP, Armien A, et al. Identification and characterization of a spore-like morphotype in chronically starved *Mycobacterium avium* subsp. *paratuberculosis* cultures. PLoS ONE 2012;7:e30648.

80. Schurr E, Gros P. A common genetic fingerprint in leprosy and Crohn's disease? New Engl J Med 2009;361:2666–2668.

81. Abubakar I, Myhill D, Aliyu SH, et al. Detection of *Mycobacterium avium* subspecies *paratuberculosis* from patients with Crohn's disease using nucleic acid-based techniques: A systematic review and meta-analysis. Inflamm Bowel Dis 2008;14:401–410.

82. Feller M, Huwiler K, Stephan R, et al. *Mycobacterium avium* subspecies *paratuberculosis* and Crohn's disease: A systematic review and meta-analysis. Lancet Infect Dis 2007;7:607–613.

83. Waddell LA, Rajic A, Sargent J, et al. The zoonotic potential of *Mycobacterium avium* spp. *paratuberculosis*: A systematic review. Can J Pub Health 2008;99:145–155.

84. Behr MA, Hanley J. Antimycobacterial therapy for Crohn's disease: A reanalysis. Lancet Infect Dis 2008;8:344.

85. Griffith DE. Therapy of nontuberculous mycobacterial disease. Curr Opin Infect Dis 2007;20:198–203.
